# Oral Abstracts for the 17th Asia Pacific Heart Rhythm Society (APHRS) Scientific Sessions

**DOI:** 10.1002/joa3.70003

**Published:** 2025-03-14

## 1 LEFT ATRIAL FUNCTION AND RESERVOIR STRAIN: IMPLICATIONS FOR DISEASE PROGRESSION IN ATRIAL FIBRILLATION

### 1.1 **MOHAMED ABBAS**, JONATHAN ARIYARATNAM, JENELLE DZIANO, JACKSON HOWIE, SHAUN EVANS, MOHANARAJ JAYAKUMAR, JOHN FITZGERALD, MELISSA MIDDELDROP, ADRIAN ELLIOTT, PRASH SANDERS

#### 1.1.1 Royal Adelaide Hospital, Adelaide, Australia

**Introduction:** Atrial fibrillation (AF) and heart failure with preserved ejection fraction (HFpEF) often overlap and are characterized by progressive alterations in left atrial (LA) structure and function. LA reservoir strain (LASr) serves as a marker of atrial mechanical function and remodeling in patients with AF and has been proposed to predict outcome. This study aims to examine the predictive role of LASr in AF and its potential as a marker for disease progression.

**Methods:** We prospectively recruited patients with paroxysmal or persistent AF and preserved left ventricular (LV) ejection fraction scheduled for catheter ablation. Baseline two‐ dimensional echocardiography was performed prior to ablation to quantify LA volume and reservoir strain. Intra‐ procedural hemodynamic measurements were obtained at baseline and with direct LA infusion to classify patients into clinical HFpEF (baseline mean LA pressure >15 mmHg), pre‐clinical HFpEF (baseline mean LA pressure <15 mmHg, increasing to >15 mmHg post‐fluid challenge), or no HFpEF (mean LA pressure <15 mmHg). Intra‐procedural transoesophageal echocardiogram was also performed to assess LA dimensions in response to fluid challenge. LA compliance was calculated as ΔLA dimensions/ΔLA pressure.

**Results:** Of the 125 patients (mean age 65 ± 11 years; 73% male) enrolled, those with persistent AF (50.4%) exhibited reduced LASr compared to those with paroxysmal AF (p<0.001). Lower LASr correlated with older age (r=0.48, p<0.001), female sex (p=0.028), and presence of HFpEF (p=0.005). Reduced LASr was associated with longer diagnosis to ablation time (r=0.19, p=0.002) and Individuals with prior AF ablation showed diminished LASr compared to de‐novo cases (p<0.01). Furthermore, higher LASr was associated with greater LA compliance (r=0.24, p<0.001).

**Conclusions:** Echocardiographic assessment of LA reservoir strain holds promise as a surrogate for disease progression in patients with AF. This non‐invasive method may aid in predicting treatment response in future interventional AF studies.

## 2 ELECTROPHYSIOLOGICAL CHARACTERISTICS AND ROLE OF THE EPICARDIAL SEPTO‐PULMONARY BUNDLE IN LEFT ATRIAL TACHYARRHYTHMIA

### 2.1 **RAKESH AGARWAL** ^1^, ANH HONG NGUYEN^1^, FADHLY SYAH AMRI^2^, RAJIV MAHAJAN^1^

#### 2.1.1 ^1^University of Adelaide and the Lyell McEwin Hospital, Adelaide, Australia,^2^Abbott Medical, Adelaide, Australia

**Introduction:** Endo‐epicardial connections of the Septo‐Pulmonary Bundle (SPB) can lead to persistent pulmonary vein or posterior wall connections despite endocardial block after atrial fibrillation (AF) ablation. Strategies for delineating SPB and its electro‐anatomical characteristics are poorly undefined.

**Methods:** 15 patients with SPB‐mediated posterior wall activation were identified. The epi‐endo connections from SPB into the posterior wall were confirmed if the following criteria were met:Double potentials and lack of capture (10A, 1ms) along the entire single‐ringEarliest activation within the single ring >5 mm away from the ablation line and later activation near the linePacing inside the ring showed the earliest activation anterior to RSPV away from the single ring.Electro‐anatomic data were collected and analyzed.

**Results:** 15 patients (8 Male and 7 Female) with a mean age of 60.3 (±13.3) years were followed up for a median of 5 months (IQR 1‐12 months). Distal connections of SPB were unifocal in 2, multifocal in 11 and diffuse in 2 participants. The proximal insertion of SPB was mapped in 3 participants and was pseudo focal anterior to RSPV. The mean conduction velocity across the epicardial connections of the SPB was 0.56±0.31 m/sec after ablation as compared to the pre‐ablation endocardial velocity of 2.29±0.91m/s. SPB was found to be a critical isthmus for atrial macro entrant tachycardia. The successful ablation strategy for SPB activation of posterior wall was ablation of the earliest activity (n=13) or consolidation the roof line (n‐2)at suspected site of SPB crossover.

**Conclusions:** SPB can be a cause of persistent left atrial activation after PVI in 30% cases and serve as critical isthmus for macro re‐entry due to slowed conduction. High density mapping and delineation of endocardial connections of SPB can achieve durable SRI.

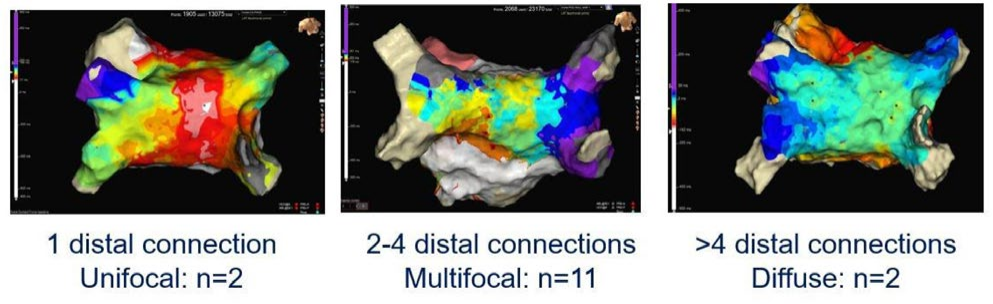

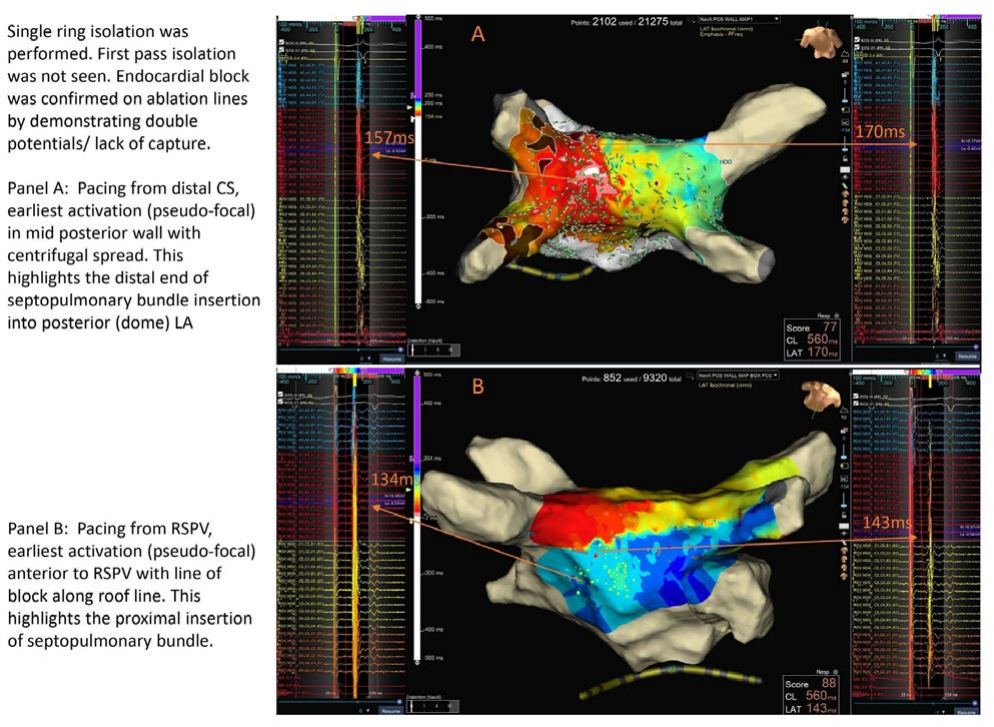

## 3 POLYGENIC RISK‐BASED PREDICTION OF HEART FAILURE IN YOUNG ATRIAL FIBRILLATION: AN ANALYSIS FROM UK BIOBANK

### 3.1 **HYO‐JEONG AHN** ^1^, TAE‐MIN RHEE^2^, EUE‐KEUN CHOI^1^, KYUNG‐YEON LEE^1^, JUNGMIN CHOI^1^, JAE‐HYUN KIM^1^, SEOKMOON HAN^1^, SOONIL KWON^3^, SO‐RYOUNG LEE^1^, SEIL OH^1^, GREGORY Y. H. LIP^4^

#### 3.1.1 ^1^Seoul National University Hospital, Seoul, Korea, Republic of,^2^Seoul National University Hospital Healthcare System Gangnam Center, Seoul, Korea, Republic of,^3^Seoul National University Boramae Medical Center, Seoul, Korea, Republic of,^4^Liverpool Centre for Cardiovascular Science at University of Liverpool, Liverpool, United Kingdom

**Introduction:** Heart failure (HF) is the most concerning morbidity in atrial fibrillation (AF) through mutual influence on poor prognosis. The polygenic risk score (PRS) has recently been proposed to improve the risk prediction for cardiovascular disease. However, the additive predictive role of PRS for HF in AF who inherently carry a high risk of HF is unknown.

**Methods:** From the UK biobank, we identified 21,167 Caucasian participants with newly diagnosed AF without a prior history of HF. The PRS for HF was constructed using genetic instruments from previous genome‐wide association studies. The primary outcome was the occurrence of incident HF. The prediction of incident HF was evaluated using the tertile categorization of PRS for HF (low vs. moderate‐high PRS) across the entire AF cohort, as well as within age subgroups (young AF, <60 years; old AF, ≥60 years, respectively).

**Results:** The mean age was 69.0 years in the total population (55.2 years in age <60 years; 70.7 years in age ≥60 years group). During a median follow‐up of 3.8 years, the incidence rate (1000‐patient year) of HF was 29.9. In the total population, AF patients with moderate‐high PRS for HF presented a higher risk of HF than those with low PRS for HF [adjusted hazard ratio (HR) with 95% confidence interval (CI), 1.18 (1.05‐1.32), p=0.005]. The higher risk of HF in the moderate‐high PRS group was particularly accentuated in young AF, showing a greater increment of HRs: adjusted HR, 2.14 in young AF, and 1.13 in old AF, p‐for‐interaction=0.015. In young AF, the onset of incident HF was earlier in those with the moderate‐high PRS group (the median time from AF diagnosis to incident HF, 4.2 years in low PRS vs. 1.5 years in the moderate‐high PRS group, p=0.001). The prediction of HF was significantly improved by adding PRS to the clinical risk factors for HF, especially in young AF patients, with a net reclassification improvement of 29.7% (p=0.003).

**Conclusions:** PRS for HF can significantly improve the prediction of incident HF in patients with AF, especially in the young population, providing a clinical utility of an individualized approach to integrated management of AF.

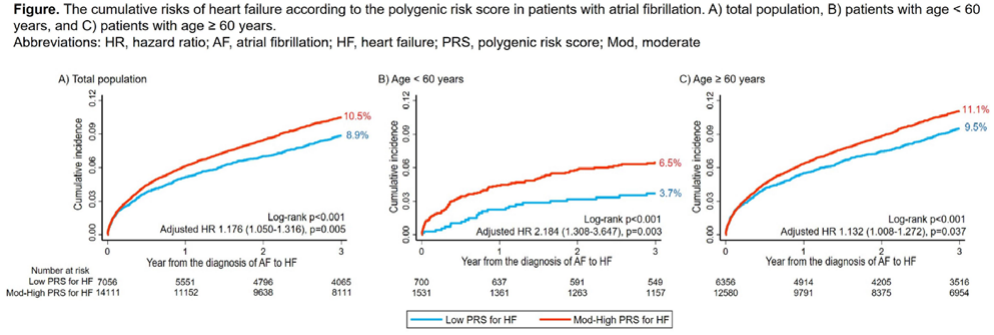

## 4 THE INCIDENCE, PREVALENCE, RISK FACTORS, MORBIDITY AND MORTALITY ASSOCIATED WITH ATRIAL FIBRILLATION IN DEVELOPING VERSUS DEVELOPED COUNTRIES: A SURVEY OF 204 COUNTRIES FROM 1990‐2019

### 4.1 **MISHKA AMARASEKARA** ^1^, MADHUKA WIJAYARATHNE^1^, THALYS RODRIGUES^1^, GEOFFREY WONG^1^, RAJIV MAHAJAN^2,3^, OMAR FAROUQUE^1^, PRASHANTHAN SANDERS^4,3^, HAN LIM^1,5^

#### 4.1.1 ^1^Austin Health, Melbourne, Australia,^2^Lyell McEwin Hospital, Adelaide, Australia,^3^University of Adelaide, Adelaide, Australia,^4^Centre for Heart Rhythm Disorders, Royal Adelaide Hospital, Adelaide, Australia,^5^The University of Melbourne, Melbourne, Australia

**Introduction:** Atrial fibrillation (AF) remains the most prevalent cardiac arrythmia worldwide. Literature remains limited on differences between developing and developed countries. We aimed to explore the burden, risk factor profiles and temporal changes between developing and developed countries in regard to its impact on morbidity, mortality and incidence of ischaemic stroke.

**Methods:** Data was extracted from the Institute for Health Metrics and Evaluation query tool from 204 countries (1990‐2019) and divided according to socio‐economic indices (SDIs). Age‐standardised rates (ASR) of incidence, prevalence, disability‐adjusted life‐years (DALYs) and mortality of AF were calculated.

**Results:**

Incidence and Prevalence of AF

From 1990‐2019, the highest AF incidence and prevalence were within the higher SDI countries.

Risk factors

Lower SDI countries noted an increase of 18% in hypertension and 109% in obesity attributable AF DALYs within the last 30 years.Incidence rates of rheumatic heart disease were 10x greater in low SDI countries and remained a major contributor to AF‐related mortality and DALYs in such countries over the 30 years.

DALYs, mortality and ischaemic stroke incidence

Over the 30 years, DALYs decreased by 10.4% and attributable mortality by 1.9% in higher SDI countries. Conversely, low‐middle SDI countries showed an increase of 8.1% in DALYs and 14.2% in mortality. High SDI countries showed a 35.6% decrease in incidence of ischaemic stroke whereas middle SDI countries showed an increase of 11.1%.

**Conclusions:** Although AF remained most prevalent within higher SDI countries, incidence of ischaemic stroke, DALYs and attributable mortality have decreased over a 30‐year period. However, in developing countries there were an alarming increase in lifestyle related AF risk factors, DALYs, attributable mortality and associated incidence of ischaemic stroke over the same period. This highlights the urgent need for targeted prevention and management measures for AF in low‐middle SDI countries.

## 5 CORONARY SINUS ISOLATION IN ADDITION TO PULMONARY VEIN ISOLATION AND ROOFLINE ABLATION FOR PATIENTS WITH PAROXYSMAL AND PERSISTENT ATRIAL FIBRILLATION: A RANDOMIZED CONTROLLED TRIAL

### 5.1 **JONATHAN ARIYARATNAM** ^1^, ANTHONY BROOKS^2^, MELISSA MIDDELDORP^1^, KADHIM KADHIM^1^, PRASH SANDERS^1^

#### 5.1.1 ^1^University of Adelaide, Adelaide, Australia,^2^Microport, Adelaide, Australia

**Introduction:** Clinically significant arrhythmias commonly arise from the coronary sinus and previous studies have suggested that ablation of the CS can prolong atrial fibrillation (AF) cycle length or even terminate AF. However, the role of de novo CS isolation in the treatment of paroxysmal or persistent atrial fibrillation has yet to be established. This randomized controlled trial sought to investigate the impact of coronary sinus ablation in addition to traditional ablation targets on AF recurrence in patients undergoing AF ablation.

**Methods:** Participants were eligible for inclusion if they had symptomatic paroxysmal or persistent AF despite the use of antiarrhythmic medications and were scheduled for first‐time AF ablation. Participants were randomized to undergo either PVI, roofline ablation and CS isolation (Group 1) or PVI and roofline ablation alone (Group 2). The primary outcome was single procedure drug free (SP‐DF) atrial arrhythmia‐free survival at 2 years. Multi‐procedure drug assisted (MP‐DA) atrial arrhythmia‐free survival was assessed over a 5‐year follow‐up period.

**Results:** Between 2008 and 2013, 100 patients were included in the study; 48 were randomized to Group 1 and 52 were randomized to Group 2. Baseline characteristics were well‐matched between groups. Complete acute PVI and roofline block was achieved in 100% of patients in Group 1 and 98% of patients in Group 2. Acute CS isolation was successful in 94% of patients in Group 1. SP‐DF success at 2 years was 62% in Group 1 and 63% in Group 2; SP‐DF survival was not different between groups (Fig A; Log‐Rank p=0.908). MP‐DA success at 5 years was 92% in Group 1 and 90% in Group 2; MP‐DA survival also was not different between groups at 5 years (Fig B; Log‐Rank p=0.801).

**Conclusions:** The adjunctive ablation of the coronary sinus in paroxysmal or persistent AF does not achieve longer atrial arrhythmia‐free survival time compared to PVI and roofline ablation alone.

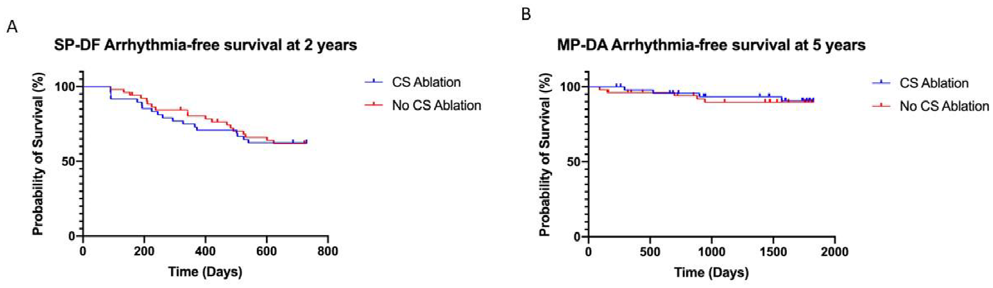

## 6 INVASIVE HAEMODYNAMIC MEASUREMENTS TO ASSESS THE INFLUENCE OF HFPEF ON SYMPTOMS OF EXERTIONAL DYSPNOEA AND PALPITATIONS IN ATRIAL FIBRILLATION

### 6.1 **JONATHAN ARIYARATNAM**, MOHAMED ABBAS, MELISSA MIDDELDORP, JANELLE D'ZIANO, JACKSON HOWIE, PRASH SANDERS, ADRIAN ELLIOTT

#### 6.1.1 University of Adelaide, Adelaide, Australia

**Introduction:** Exertional dyspnoea and palpitations are common symptoms amongst patients with atrial fibrillation (AF). We sought to determine whether these symptoms are attributable to heart failure with preserved ejection fraction, which commonly coexists in patients with AF.

**Methods:** Consecutive patients with symptomatic AF and preserved left ventricular ejection fraction awaiting AF ablation were recruited. The presence and severity of exertional dyspnoea and palpitations was assessed using the AF Symptom Severity (AFSS) questionnaire, in which patients rate these symptoms on a scare of 0‐5. The presence of HFpEF was assessed invasively at AF ablation; HFpEF was diagnosed when mean LA pressure was greater than 15mmHg at baseline or after infusion of 500mls saline. Other hallmarks of HFpEF assessed were exercise capacity (VO_2Peak_) using cardiopulmonary exercise testing and LA and LV strain using transthoracic echocardiography. AFSS was reassessed 12‐months following ablation.

**Results:** Amongst 107 patients with symptomatic AF, exertional dyspnoea was present in 95 (88.8%) whilst palpitations were reported in 82 (76.6%) participants. Exertional dyspnoea was associated increased risk of HFpEF (71 [78.0%,] vs 3 [37.5%], p<0.01) raised mLAP (14.2±4.5 vs 11.4±3.6mmHg, p=0.04), reduced VO_2Peak_ (22.6±4.9 vs 25.3±3.4ml/kg/min, p=0.03), reduced LA reservoir strain (19.3±8.9 vs 29.3±10.4, p=0.01) and reduced LV strain (15.4±3.6 vs 17.8±2.8%, p=0.05). On the other hand, palpitations were not associated with HFpEF diagnosis (61 [77.2%] vs 13 [56.5%], p=0.09), mLAP (14.9±4.1 vs 12.7±4.8, p=0.08), VO_2Peak_ (22.7±5.0 vs 23.4±4.2%, p=0.52)_,_ LA strain (20.6±9.8 vs 19.6±8.8%, p=0.63) or LV strain (15.5±3.6 vs 15.9±3.9, p=0.71). Ongoing exertional dyspnoea post‐ablation were more common amongst patients with HFpEF compared to patients without HFpEF (79 vs 50%, p=0.05).

**Conclusions:** Exertional dyspnoea is associated with invasive and non‐invasive hallmarks of HFpEF whereas palpitations are not. Ongoing exertional dyspnoea after ablation is more likely to occur in patients with coexistent HFpEF.

Chair

**F. Bahlke**

German Heart Center Munich, Munich, Germany

## 7 LEFT‐TO‐RIGHT ATRIOVENTRICULAR ACTIVATION DELAY, A NOVEL MEASURE OF AV‐CONDUCTION PREDICTING CLINICAL OUTCOMES IN NON‐LBBB PATIENTS

### 7.1 **ANIKA BEIERLE** ^1^, WOJCIECH ZAREBA^2^, AUGE RICHARD^1^, SPENCER ROSERO^2^, SCOTT MCNITT^2^, MARTIN STOCKBURGER^1^, VALENTINA KUTYIFA^2^

#### 7.1.1 ^1^Havelland Kliniken, Academic Teaching Hospital of Charité, Nauen, Germany,^2^University of Rochester Medical Center, Clinical Cardiovascular Research Center, Rochester, NY

**Introduction:** PR‐interval reflects AV‐timing but it does not well characterize adverse hemodynamics. The DEEPER‐PR mechanistic study identified novel measures of AV‐conduction correlating with Doppler‐derived cardiac performance but the clinical significance of these parameters is unknown.

**Methods:** We analyzed time intervals from 12‐lead ECGs in 535 non‐LBBB patients enrolled in MADIT‐CRT. The earliest onset of atrial activation, zero crossing of the P wave in lead V1, earliest onset of the QRS complex, and time interval to the first peak of the R wave in V1 and V6 were determined. Endpoints included HF or death and the occurrence of device‐treated VA events. Association between novel measures of AV‐conduction and clinical outcomes in ICD patients (n=209) and CRT‐D (n=326) vs. ICD benefit were assessed using Kaplan‐Meier and multivariable Cox regression analyses.

**Results:** We identified quintile 5 of P0PV1≥201 ms (n=159, 30%) as the strongest risk predictor in ICD patients, over PR‐interval, with more than 3‐fold increase in the risk of HF/death events (p<0.001) but not for the occurrence of device‐treated VA events (p=0.856). In this group, CRT‐D was associated with a 66% lower risk of HF/Death (95% CI: 0.22‐0.68, p=0.001) vs. an ICD. By contrast, in patients with a P0PV1<201ms, CRT‐D vs. an ICD was associated with a 64% increased risk of HF/death (95% CI: 1.12‐2.55, p=0.012), with significant bidirectional interaction (p‐value<0.001). In addition Kaplan‐Meier analyses showed that in patients with a P0PV1<201ms, the 4‐year cumulative probability of VT/VF was 35% with CRT‐D and 36% with an ICD (p=0.497). In patients with longer P0PV1, we also found similar VT/VF rates in patients with CRT‐D and in those with an ICD (30% in both groups, p=0.692).

**Conclusions:** We propose a novel variable, left‐to‐right atrioventricular activation delay (P0PV1) to identify high‐risk ICD patients and benefit vs. harm from CRT‐D in patients with non‐LBBB. CRT‐D treatment according to P0PV1 does not modify the arrhythmogenic substrate in patients with non‐LBBB. Prospective studies are warranted to confirm P0PV1 as a predictor of CRT‐D response.

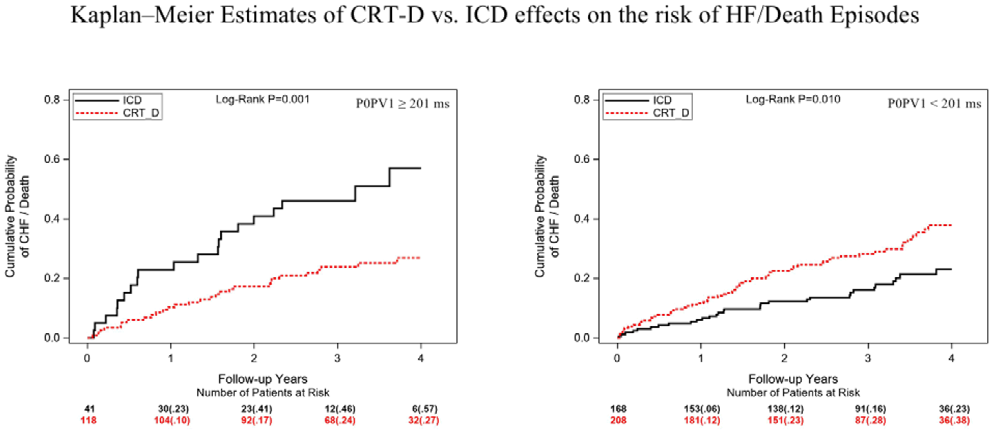

## 8 IDIOPATHIC RIGHT VENTRICULAR ARRHYTHMIAS NOT ARISING FROM THE OUTFLOW TRACT: PREVALENCE, ELECTROCARDIOGRAPHIC CHARACTERISTICS, AND OUTCOME OF CATHETER ABLATION

### 8.1 **LORI BELL** ^1,2^, NATASHA JONES‐LEWIS^1,2^, LUKAH Q TUAN^1,2,3^, ADRIANA TOKICH^1,2,3^, LARA G. B. HEDLEY^1,2,3^, ANUGRAH NAIR^1,2,3^, JENISH SHROFF^1,2,3^, RAJEEV K PATHAK^1,2,3,4^

#### 8.1.1 ^1^Canberra Heart Rhythm, Canberra, Australia,^2^Canberra Heart Rhythm Foundation, Canberra, Australia,^3^Australian National University, Canberra, Australia,^4^University of Canberra, Canberra, Australia

**Introduction:** Most idiopathic right ventricular (RV) ventricular arrhythmias (VAs) originate from the outflow tract. Data on VA from the lower body of the RV are limited. We aim to describe to the idiopathic VAs detailing the prevalence and characteristics of VT arising from the body of the RV.

**Methods:** Of 206 pts who underwent VA catheter ablations from 2019‐2024, 30pts (14.6%) had PVCs mapped to RV non‐outflow tract. Clinical and ECG characteristics, cardiac function and results of radiofrequency (RF) ablation were analyzed.

**Results:** Of RV non‐outflow tract patients, 17 (56.6%) had PVCs mapped within 2 cm of the tricuspid valve annulus (TVA), Among the VAs from the TVA, 4 (23.5%) originated from the free wall and 9 (52%) from the septum (Parahisian). 11 (65%) patients had VA, 6 (54%) from the basal and 5 (45%) from the apical RV segments. Clinical PVC had left bundle branch morphology in all cases. V3 transition seen in all PVCs from Parahisian region and RV septum. 66% of moderator band PVCs transitioned in V4, 33% showing no transition. Mean R wave amplitude is highest in PH in lead I, RV septal in lead II and MB in lead III. Mean R wave amplitude in precordial leads is highest in moderator band PVCs and lowest in RV septal. Mean QRS duration is longest in MB PVCs in all leads excluding lead II, in which RV septal is longer. 2 PH PVCs demonstrate S wave, none seen in other locations. Myocardial scar was demonstrated on MRI in 4 PH pts (23.5%), 1 RV septal (11%) and not seen in MB cohort. Left ventricular ejection fraction (LVEF) at baseline is low in 17% and normal in 83% of RV PH pts (mean LVEF 51±12.1); low in 33% and normal in 66% of RV septal (mean LVEF 50±12.6), and normal EF seen in all MB pts (mean LVEF 63±0.8). Elimination of PVC seen in 88% of PH pts, 63% of RV septal and 75% of MB. Freedom from VA at latest follow‐up (48±8 months) is seen in 100% of RV septal and MB pts, and 87% of PH.

**Conclusions:** Catheter ablation of VAs from RV non‐outflow tract are a low‐risk and effective treatment strategy.

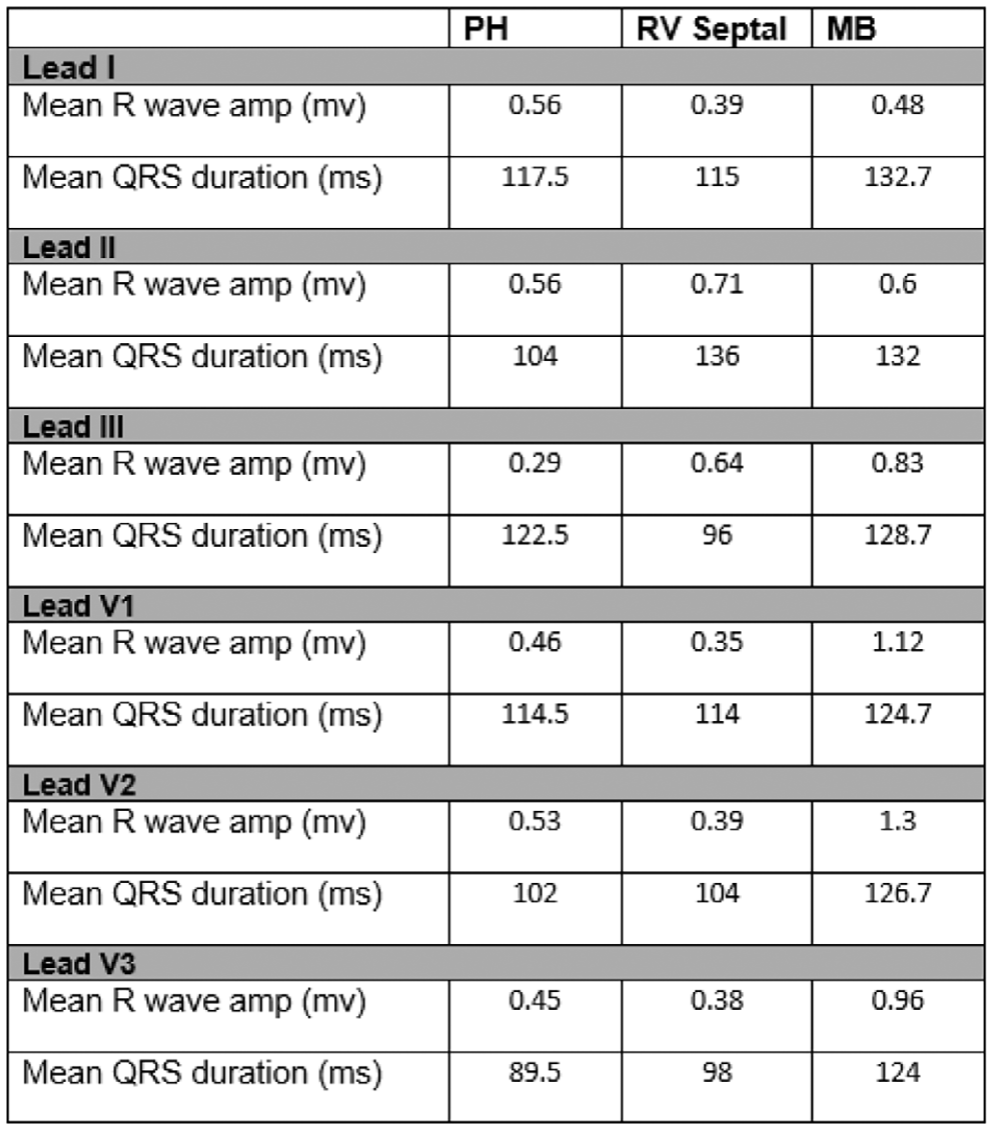

## 9 LEARNING CURVES AMONG PERMANENT LEFT BUNDLE AREA PACING (LBAP) VIA STYLET DRIVEN LEAD (SDL) VERSUS LUMEN LESS LEADS ‐ A MULTI‐CENTRE RETROSPECTIVE STUDY

### 9.1 SOUVIK JANA^1^, SAROJ KR CHOWDHURY^1^, AYAN KAR^1^, SAYED RANA^1^, ADITYA VERMA^2^, ATAHAR ALI^3^, TUSHAR ASIF ZAMAN^3^, POPPY BALA^3^, **DEBABRATA BERA** ^1^

#### 9.1.1 ^1^RTIICS, Kolkata, India,^2^Manipal Hospitals, Kolkata, India,^3^Evercare Hospitals, Dhaka, Bangladesh

**Introduction:** Left bundle area pacing has a steady learning curve. We compared the 2 types of leads regarding their technical challenges and success rates.

**Methods:** This is a retrospective study was carried out in 3 Tertiary Care Centre by 6 operators with SDL Abbott Tendril and Lumen less lead (LLL) 3830 Medtronic leads . LBAP was defined as with qR in V1 along with one of the definitive evidence of left bundle capture. Left posterior fascicular pacing ‐ when LAFB pattern was noted in addition to established LBAP criteria.

**Results:** Total 73 consecutive patients were included among which 48 cases underwent successful CSP (M/F=28/20, Mean age 67 yrs, Mean EF 51±9%). Among 40 cases of LLL, 18 cases (45%) were successful to achieve CSP. The main reason for failure was unable to penetrate the septum even with rapid torque. Among those 18 cases the mean fluoroscopy time was 27±10 minutes. Among the Abbott SDL leads 30/33 cases (91%) were successful. The fluoroscopy time was 13±5 minutes (p<0.05). Among successful cases, median no of attempts to penetrate the septum were 5 in LLL vs. 1 in SDL (p< 0.01). Among the successful LLL cases 15/18 (83.3%) achieved main LBAP whereas among the Abbott SDL cases 22/30(73%) were left posterior fascicular pacing (LPFP) possibly due to the difference in the shape and reach of the sheaths. No major complication. The capture threshold and sensing amplitudes were similar among both groups at implant and at follow‐up of 16±12 months. There was similar proportion of cases (6% each) where loss in conduction system capture were noted on follow up. No late perforation into left ventricle.

**Conclusions:** SDL has much shorter learning curve, lesser technical challenge, lower fluoroscopy time and higher success rate. SDL shall be the preferred choice for beginners. LLL has better reach to main LB area.

Chair

**T. Bunch**;

University of Utah Hospital, Salt Lake City, UT

## 10 EVOLUTION OF SUBSTRATE FOR VENTRICULAR ARRHYTHMIAS EARLY POST‐INFARCTION: INSIGHTS FROM A PORCINE ISCHEMIA‐REPERFUSION MODEL

### 10.1 ASHWIN BHASKARAN

#### 10.1.1 Westmead Hospital, Westmead, Australia

**Introduction:** The evolution of myocardial scar and its arrhythmogenic potential post‐infarct is incompletely understood. Our objective was to investigate scar and border zone (BZ) channels evolution in an animal ischemia‐reperfusion injury model using late gadolinium enhancement cardiac magnetic resonance (LGE‐CMR).

**Methods:** Five swine underwent 90‐minute balloon occlusion of the mid‐left anterior descending artery, followed by LGE‐CMR at day (d) 3, d30 and d58 post‐infarct. Invasive electroanatomic mapping (EAM) was performed at 2 months. Topographical reconstructions of LGE‐CMR were analyzed for left ventricular core and BZ scar, BZ channel geometry and complexity, including transmurality, orientation and number of entrances/exits.

**Results:** LVEF reduced from 48%±1.8% to 41.3%±2.3% post‐infarct. Total scar mass reduced over time (*P*=0.008), including BZ (*P*=0.002) and core scar (*P*=0.05). 72 BZ channels were analyzed across all animals and timepoints. Channel length (*P*=0.05) and complexity (*P*=0.02) reduced progressively from d3 to d58. However, at d58, 64% of channels were newly formed and 36% were mid‐myocardial. Conserved channels were initially longer and more complex. All LGE‐CMR channels co‐localized to regions of maximal decrement on EAM, with significantly greater decrement (115±31msec vs 83±29msec, *P*<0.001) and uncovering of split potentials (24.8% vs 2.6%, *P*<0.001) within channels. Three of five animals had inducible VT and tended to have more channels with greater mid‐myocardial involvement and functional decrement than those without VT.

**Conclusions:** BZ channels form early post‐infarct, demonstrate evolutionary complexity and functional conduction slowing on EAM, highlighting their arrhythmogenic potential. Some channels regress in complexity and length, but new channels form at 2 months’ post‐infarct, which may be mid‐myocardial, reflecting an evolving, three‐dimensional substrate for VT. LGE‐CMR may help identify BZ channels that may support VT early post‐infarct and lead to sudden death.

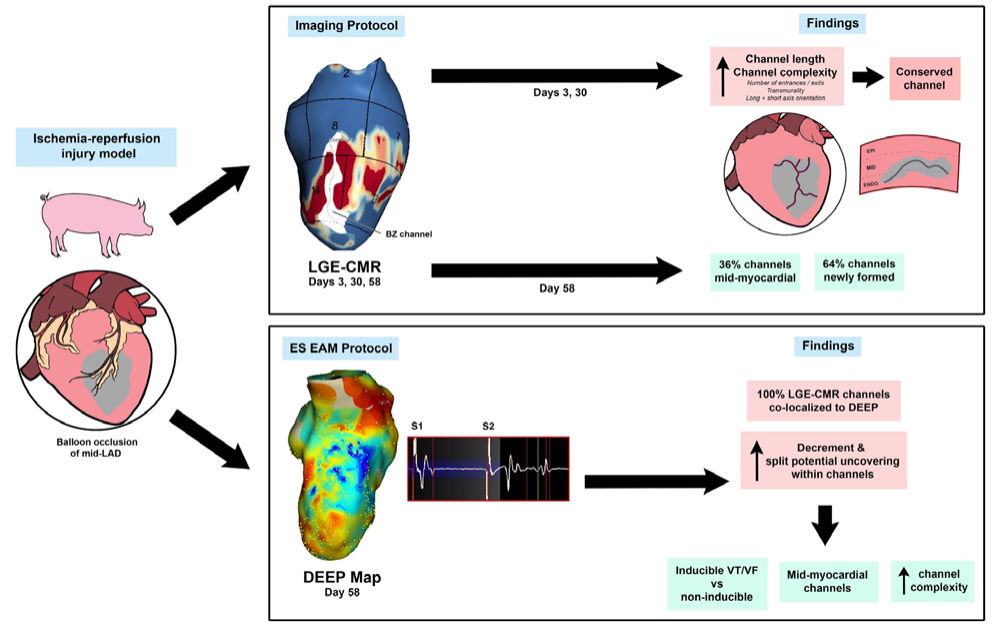

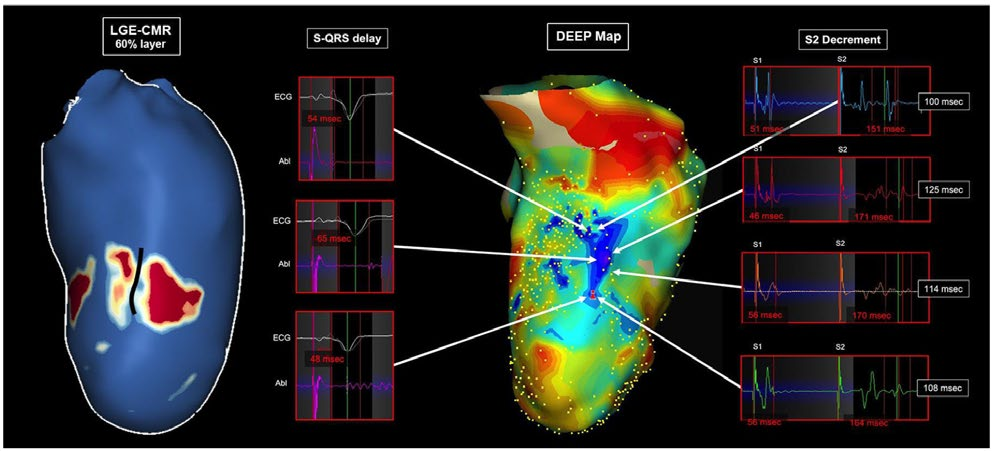

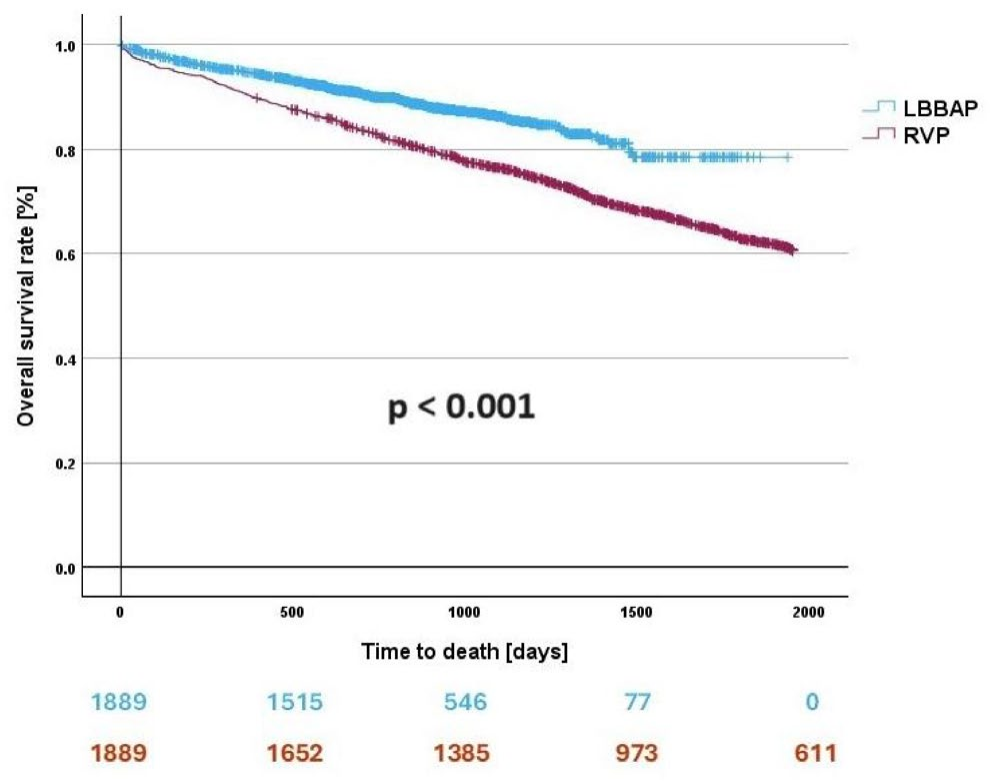

## 11 ALL‐CAUSE MORTALITY WITH LEFT BUNDLE BRANCH PACING VERSUS RIGHT VENTRICULAR PACING IN PATIENTS WITH ATRIOVENTRICULAR BLOCK (MELOS RELOADED)

### 11.1 **HARAN BURRI** ^1^, GRZEGORZ KIEłBASA^2^, OSCAR CANO^3^, KAROL CURILA^4^, FRANCESCO ZANON^5^, CATALIN PESTREA^6^, JAN DE POOTER^7^, KEVIN VERNOOY^8^, NARD RADEMAKERS^9^, DAVID ZIZEK^10^, DOMENICO GRIECO^11^, WIM HUYBRECHTS^12^, PHILIPP KRISAI^13^, ZACHARY WHINNETT^14^, MAREK JASTRZęBSKI^15^

#### 11.1.1 ^1^University Hospital of Geneva, Geneva, Switzerland,^2^First Department of Cardiology, Krakow, Poland,^3^Hospital Universitari i Politècnic la Fe, Valencia, Spain,^4^Charles University of Prague, Prague, Czech Republic,^5^Santa Maria della Misericordia General Hospital, Rovigo, Italy,^6^Brasov County Clinical Emergency Hospital, Brasov, Romania,^7^University Hospital Ghent, Ghent, Belgium,^8^Maastricht University. Medical Center,, Maastricht, Netherlands,^9^Catharina Ziekenhuis, Eindhoven, Netherlands,^10^University Medical Centre Ljubljana, Ljubljana, Slovenia,^11^Policlinico Casilino, Rome, Italy,^12^University Hospital of Antwerp, Antwerp, Belgium,^13^University Hospital of Basel, Basel, Switzerland,^14^Imperial College London, London, United Kingdom,^15^First Department of Cardiology and Electrocardiology, Jagiellonia University, Krakow, Poland

**Introduction:** Left bundle branch area pacing (LBBAP) is becoming an increasingly popular method of permanent pacing. However, robust data on long‐term outcomes, particularly in comparison to right ventricular pacing (RVP), are lacking.

**Methods:** This was a retrospective observational study based on mostly prospectively maintained local registries. Consecutive patients with atrioventricular block, left ventricular ejection fraction (LVEF) > 40% and ventricular pacing > 40% who received a LBBAP pacemaker were included. Patients who received RVP pacemakers, implanted in the same centres but before the era of LBBAP, served as the control group. The primary endpoint was all‐cause death and the secondary combined endpoint was all‐cause death, hospitalisation for heart failure, or upgrade to cardiac resynchronisation therapy.

**Results:** A total of 3778 patients receiving LBBAP or RVP (1:1 ratio) were studied (mean age 76±13 years, 48% males, LVEF 58 ±7%). Median follow‐up was 801 days (1062 ‐ 568 days) for the LBBAP group and 1506 days (1950 ‐ 905 days) for the RVP group. There were 216 (11.4%) deaths in the LBBAP group and 663 (35.1%) deaths in the RVP group. After 4 years of follow‐up, the Kaplan‐Meier curve (Figure 1) showed an absolute difference in survival of 12.3%. LBBAP was the strongest independent predictor of better survival (Table 1) with a hazard ratio of 0.49 (p < 0.001). The combined secondary endpoint was achieved by 274 (14.5%) in the LBBAP group and 756 (40%) in the RVP group. After 4 years of follow‐up, the Kaplan‐Meier curves for the combined secondary endpoint were separated by an absolute difference of 13.2%. No difference was observed in standard pacing parameters and the overall incidence of acute and long‐term complications between LBBAP and RVP.

**Conclusions:** This is the first large study to provide data showing that routine use of LBBAP in patients with atrioventricular block without indications for CRT is associated with significantly lower long‐term mortality compared to RVP. This strengthens the use of LBBAP in this population while awaiting the results of ongoing randomised trials.

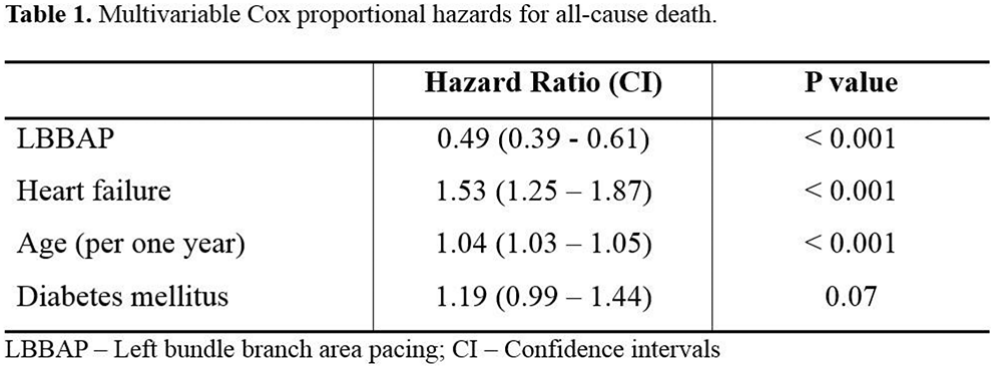

## 12 THE INHIBITORY EFFECT OF FLUOXETINE ON GAIN‐OF‐FUNCTION *KCND3* VARIANT

### 12.1 **TSERENLKHAM BYAMBAJAV** ^1^, KOICHIRO TAKAYAMA^1^, DIMITAR ZANKOV^1^, MINORU HORIE^2^, SEIKO OHNO^1^

#### 12.1.1 ^1^National Cerebral and Cardiovascular Center, Suita, Japan,^2^Shiga University of Medical Science, Otsu, Japan

**Introduction:** Kv4.3 encoded by *KCND3* is an α‐subunit of the I_to_ channel and expressed in both brain and heart. We have reported a gain‐of‐function (GOF) *KCND3* variant, p.G306A, which was identified in a young patient with early repolarization syndrome and refractory epilepsy. The variant increased peak current and slowed inactivation in I_to_. Recently, sudden unexpected death in epilepsy (SUDEP) has been noticed because it is the most frequent cause of death in patients with epilepsy. Therefore, the elucidation of its pathogenesis and the development of safe and effective therapy have been required. In our patient, quinidine was effective to prevent his symptoms, and it normalized GOF effect of the I_to_ channel with Kv4.3‐G306A. We extend pharmacological investigation to selective serotonin reuptake inhibitor, fluoxetine which suppress I_to_.

**Methods:** We aimed to elucidate the pharmacological effect of fluoxetine to Kv4.3‐G306A. Plasmids with Kv4.3 wild type (WT) or G306A were transiently transfected to Chinese Hamster Ovary cells, and I_to_ were recorded using whole‐cell patch‐clamp method at 37^0^C degrees. Fluoxetine loading at 1‐20 μM was applied externally.

**Results:** The peak current densities of WT and G306A at +50 mV were 135.61±9.81 and 331.03±77.11 pA/pF, respectively, and they were decreased to 109.5±9.22 and 186.45±39.75 pA/pF by application of 20 μM fluoxetine (Figure). Although the inactivation in G306A was significantly slower than WT, they were almost normalized after fluoxetine application (Figure and Table). The effect of inhibition by fluoxetine was concentration dependent for both peak current densities and time constants.

**Conclusions:** The GOF effect of I_to_ channel by a *KCND3* variant was reversed by fluoxetine. The variant carrying patient might have a risk of SUDEP, and fluoxetine can be a therapeutic option to prevent sudden cardiac death due to SUDEP.

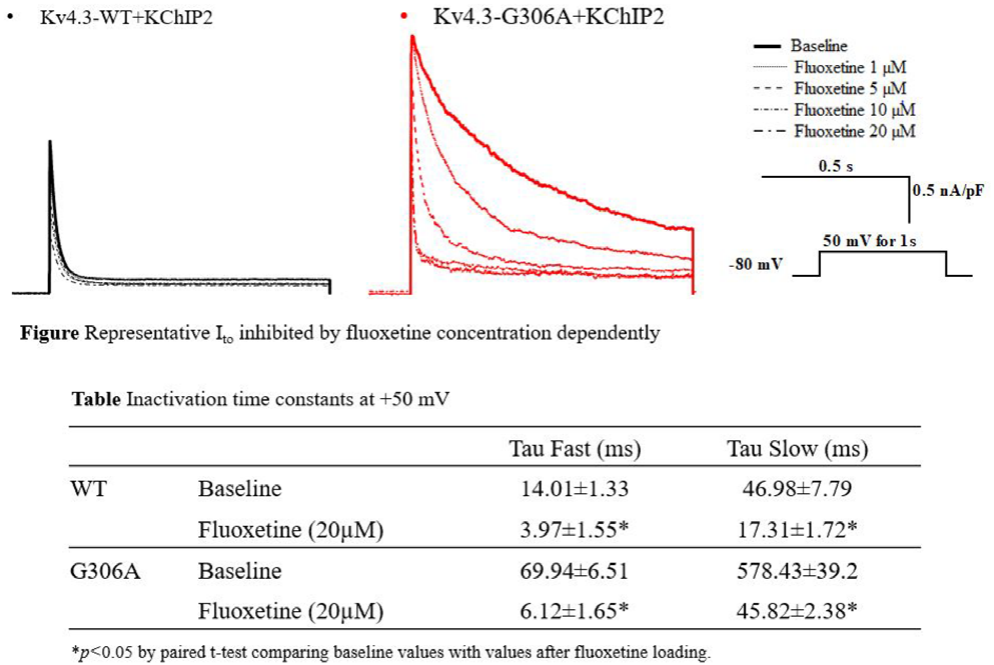

Chair

**M.‐J. Cha**

Seoul National University Hospital, Korea, Republic of

## 13 COMPARATIVE SAFETY PROFILE OF DIRECT ORAL ANTICOAGULANTS VERSUS WARFARIN IN NON‐VALVULAR ATRIAL FIBRILLATION: A META‐ANALYSIS OF RANDOMIZED CONTROLLED TRIALS AND SUBGROUP ANALYSIS OF BLEEDING ADVERSE EVENTS

### 13.1 ALVIAN YAPANTO^1^, KHAIRANI AYU LESTARI^1^, **AGUNG FABIAN CHANDRANEGARA** ^2^, ZWASTA PRIBADI MAHARDIKA^3^, WENING SARI^4^, RIFQATUSSAADAH RIFQATUSSAADAH^5^

#### 13.1.1 ^1^Faculty of Medicine YARSI University, Jakarta, Indonesia,^2^Indonesian Heart Rhythm Society, Jakarta, Indonesia,^3^Department of Medical Education, Faculty of Medicine YARSI University, Jakarta, Indonesia,^4^Department of Pharmacology, Faculty of Medicine YARSI University, Jakarta, Indonesia,^5^Department of Public Health, Faculty of Medicine YARSI University, Jakarta, Indonesia

**Introduction:** Anticoagulant therapy is crucial for managing NVAF to prevent thromboembolic events caused by irregular heart contractions. This meta‐analysis evaluates the bleeding risks of DOACs versus warfarin in NVAF patients, including a subgroup analysis of intracranial and extracranial bleeding risks associated with different DOACs.

**Methods:** Following PRISMA guidelines, we systematically reviewed literature from 2008 to 2023, extracting data from PubMed, Google Scholar, and the Cochrane Library using specific MeSH terms. Only studies comparing the bleeding risks between DOACs and warfarin in humans were included. A fixed effects model evaluated pooled hazard ratios, while I^2^ statistics assessed study heterogeneity. Study quality and potential biases were evaluated using the Cochrane Risk of Bias 2.0 tool.

**Results:** The analysis included 13 RCTs with total 175,199 patients. Results showed DOACs generally had a lower risk of major bleeding compared to warfarin, with significant reductions in extracranial bleeding (HR 0.82; 95% CI 0.78‐0.87; p=<0.005; Figure 1A) and intracranial bleeding (HR 0.64; 95% CI 0.61‐0.67; p= <0.005; Figure 1B). In both subgroup analyses, apixaban consistently showed the lowest risk among all of DOACs, with (HR: 0.42; 95% CI 0.39‐0.45; p= 0.40; Figure 1A) for extracranial bleeding and (HR: 0.69; 95% CI 0.63‐0.76; p= 0.68 Figure 1B) for extracranial bleeding, distinguishing it as the most favourable DOAC in terms of bleeding risk reduction.

**Conclusions:** In this meta‐analysis of patient with NVAF, here were significant differences between DOACs and warfarin in the incidence of major bleeding outcomes. DOACs generally exhibited a lower risk of both intracranial and extracranial bleeding compared to warfarin. Apixaban showed the most favourable profile with the lowest risk for both types of bleeding events.

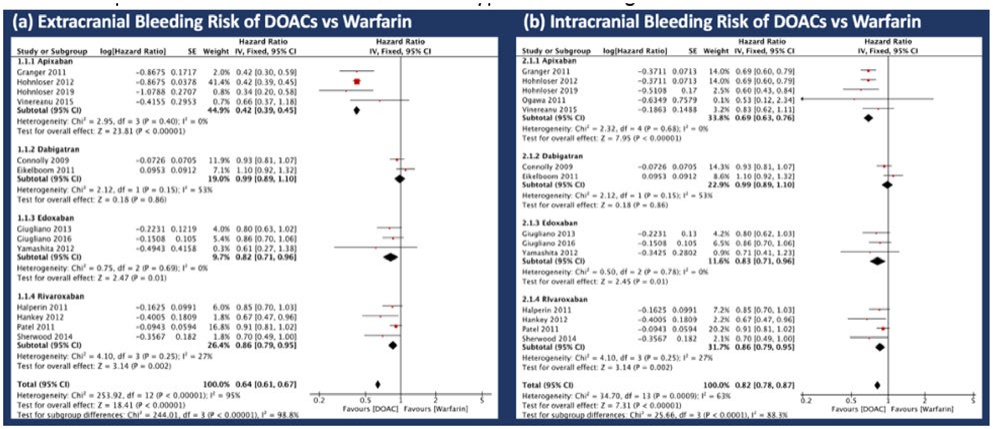

Chair

**K. Chandrasekaran**

Prashanth Hospital, India

## 14 MOLECULAR MECHANISMS OF ATRIOVENTRICULAR BLOCK DUE TO A LMNA MUTATION (R225X)

### 14.1 **MIHAIL CHELU**, TINGTING LI, XIAOLEI WANG, JIA SONG, LUGE LI, YUE YUAN, NA LI

#### 14.1.1 Baylor College of Medicine, Houston, TX

**Introduction:** Atrioventricular block (AVB) is a conduction system disorder and when severe results in pacemaker implant. LMNA mutations, affecting the nuclear envelope proteins lamin A and C, have been linked to AVB in patients, yet the mechanistic connection remains largely unexplored. A heterozygous nonsense mutation (c.C673T, p.R225X) in *LMNA* identified in a kindred was associated with progressive AVB and atrial arrhythmias, followed by ventricular dysrhythmias, left ventricular enlargement, and systolic dysfunction. We aimed to uncover the mechanisms by which the LMNA.pR225X mutation promotes AVB.

**Methods:** LMNA‐R225X knock‐in mice were generated using CRISPR/Cas9 technology. The cardiac phenotype was comprehensively characterized by surface ECG, telemetry ECG, programmed electrical stimulation, and echocardiography. Histology, immunostaining, Ca^2+^ imaging, and RNA‐seq were performed to elucidate the mechanistic underpinnings of AVB.

**Results:** The homozygous LMNA‐R225X mice displayed bradycardia and first‐degree AVB prior to death at the age of 2 weeks. Similar to patients, R225X‐heterozygous mice exhibited age‐dependent progressive AVB, ranging from 1^st^ to 3^rd^ degree, and increased susceptibility to pacing‐induced atrial fibrillation before 12‐month‐old, prior to the development cardiomyopathy at nearly 2‐year‐old, compared to age‐ and sex‐matched wildtype littermates. Histological studies showed increased fibrosis within the atrioventricular node (AVN) and nuclear morphological changes in AVN pacemaker cells of R225X‐heterozygous mice. Ca^2+^ imaging studies revealed a reduced spontaneous firing rate of the isolated AVN pacemaker cells in R225X‐heterozygous mice. Bulk RNA‐seq of the dissected AVN tissue revealed a downregulation of mRNA levels for several ion channels including *Kcnn3* (encoding SK3 channel).

**Conclusions:** The LMNA‐R225X mutation leads to progressive AVB prior to cardiomyopathy, primarily associated with increased fibrosis within the AVN and decreased SK3 channel expression in AVN pacemaker cells. This results in disrupted electrical conduction pathways and diminished pacemaker cell automaticity in the AVN.

## 15 ARTIFICIAL INTELLIGENCE‐ENHANCED ANALYSIS OF ENDOCARDIAL SIGNALS FOR PREDICTION OF ATRIAL FIBRILLATION DRIVER IN ATRIAL FIBRILLATION

### 15.1 **CHIA‐HSIN CHIANG** ^1^, CHIH‐MIN LIU^1^, YENN‐JIANG LIN^1^, MEN‐TZUNG LO^2^, SHIH‐ANN CHEN^3^

#### 15.1.1 ^1^Taipei Veterans General Hospital, Taipei City, Taiwan,^2^National Central University, Taoyuan City, Taiwan,^3^Taichung Veterans General Hospital, Taichung City, Taiwan

**Introduction:** Effective termination of persistent atrial fibrillation (AF) during ablation enhances patient outcomes. This study aimed to develop an AI model to precisely predict AF termination sites, improving ablation efficacy.

**Methods:** We retrospectively enrolled 110 patients with persistent and long‐lasting AF for pulmonary vein isolation and substrate ablation, building the DeePRISM AI model. This model integrates deep learning with PRISM (Morphological Repetitiveness by Periodicity and Similarity) analysis (Figure A). In phase I, we applied a cellular automaton technique to simulate the electrical wave propagation of AF driver, which was then detected by DeePRISM. In phase II study, we employed a prospective, independent test set comprising 37 persistent AF patients, using DeePRISM for external validation.

**Results:** In the initial phase, the DeePRISM model demonstrated an area under the curve (AUC) of 0.98, with sensitivities, specificities, and accuracies recorded at 96.45%, 90.69%, and 91.14% respectively, utilizing a cut‐off value of 0.6 (Figure B). Subsequent results from phase II showed an AUC of 0.87, alongside sensitivities, specificities, and accuracies of 74.3%, 95.4%, and 95.4% respectively. The model facilitated successful acute termination of AF in 40.5% of the cases. At a mean follow‐up period of 27.3 ± 5.5 months, Kaplan‐Meier analysis indicated that patients undergoing DeePRISM‐guided ablation exhibited significantly higher rates of arrhythmia‐free survival post‐procedure compared to the control group (70.3% vs. 35.1%, p = 0.035) (Figure C).

**Conclusions:** Utilizing the DeePRISM model enhances real‐time, automated waveform analysis during ablation, improving outcomes by accurately identifying AF termination sites and increasing arrhythmia‐free survival in persistent atrial fibrillation cases.

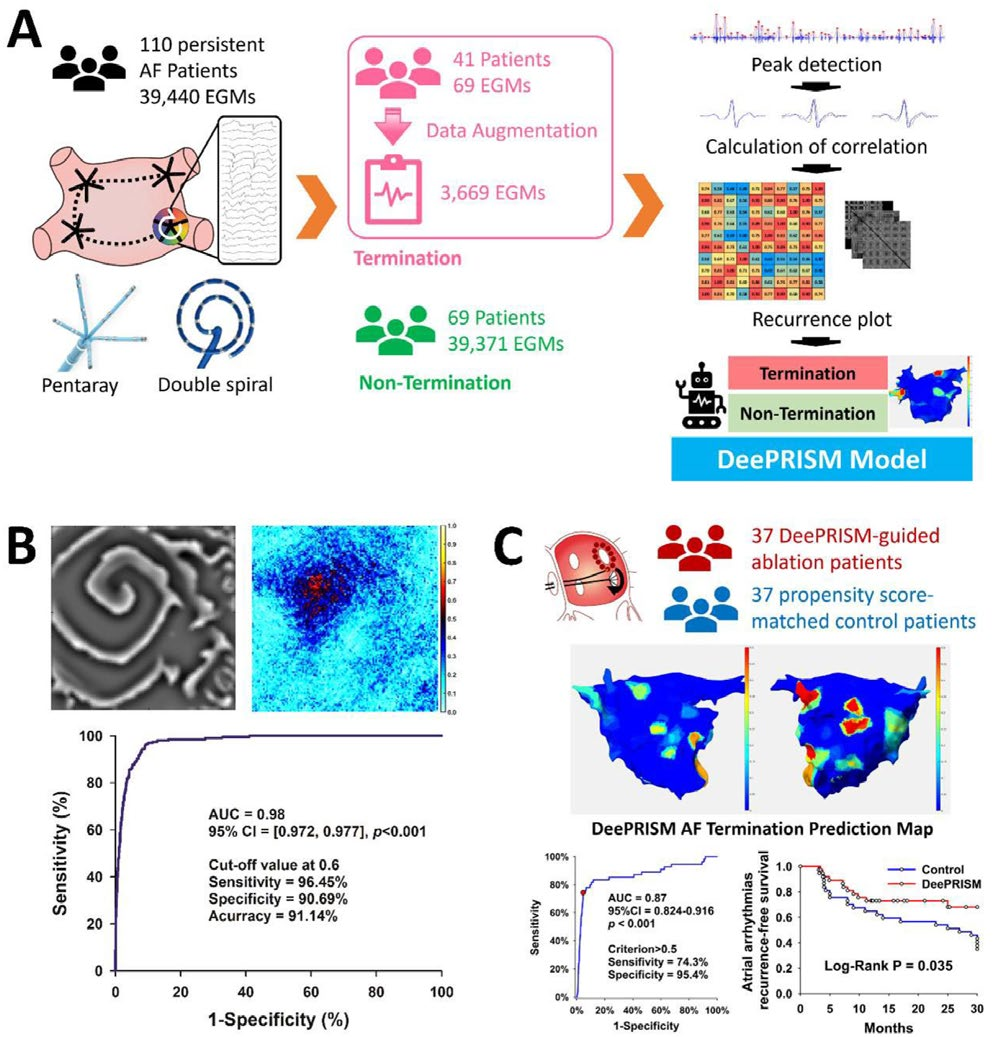

## 16 PREDICTORS OF DISEASE SEVERITY IN CHILDREN WITH CATECHOLAMINERGIC POLYMORPHIC VENTRICULAR TACHYCARDIA: A MULTICENTER COHORT STUDY IN KOREA

### 16.1 **MIN‐JUNG CHO** ^1^, MI KYOUNG SONG^2^, SO YUN JUN^2^, HYE WON KWON^2^, YOUNG HYE RYU^2^, SEUNG MIN BAEK^2^, JUNGHYE KWON^3^, AH YOUNG KIM^4^, JA KYOUNG YOON^4^, CHANG SIN KIM^4^, MI JIN KIM^5^, JAE SUK BAEK^5^, JI EUN BAN^6^, HEE JOUNG CHOI^7^, INSU CHOI^8^, JIHYE YOU^9^, JOUNG HEE BYUN^10^, HEIRIM LEE^11^, JAE YOON NA^12^, JUE SEOUNG LEE^13^, JOOWON LEE^14^, LUCY YOUNGMIN EUN^15^, YOUNG‐TAE LIM^16^, EUN JUNG BAE^2^, JUNE HUH^3^

#### 16.1.1 ^1^Gyungsang National University Changwon Hospital, Changwon, Korea, Republic of,^2^Department of Pediatrics, Seoul National University College of Medicine, Seoul National University Children's Hospital, Seoul, Korea, Republic of,^3^Department of Pediatrics, Heart vascular stroke institute, Samsung Medical Center, Sungkyunkwan University School of Medicine, Seoul, Korea, Republic of,^4^Division of Pediatric Cardiology, Department of Pediatrics, Severance Cardiovascular Hospital, Yonsei University College of Medicine, Seoul, Korea, Republic of,^5^Department of Pediatrics, University of Ulsan College of Medicine, Seoul, Korea, Republic of,^6^Department of Pediatrics, Sejong General Hospital, Bucheon, Gyeonggi‐do, Korea, Republic of,^7^Department of Pediatrics, Keimyung University School of Medicine, Daegu, Korea, Republic of,^8^Department of Pediatrics, Chonnam National University Medical School, Gwangju, Korea, Republic of,^9^Deptartment of Pediatrics, Jeonbuk National University Medical School, Jeonju, Korea, Republic of,^10^Department of Pediatrics, Pusan National University Yangsan Hospital, Pusan National University School of Medicine, Yangsan, Korea, Republic of,^11^Department of Pediatrics, Pusan National University Hospital, Busan, Korea, Republic of,^12^Departments of Pediatrics, Hanyang University College of Medicine, Seoul, Korea, Republic of,^13^Department of Pediatrics, Korea University College of Medicine and Korea University Medical Center, Seoul, Korea, Republic of,^14^Departments of Pediatrics, Seoul National University Bundang Hospital, Bundang, Korea, Republic of,^15^Department of Pediatrics, Gangnam Severance Hospital, Yonsei University College of Medicine, Seoul, Korea, Republic of,^16^Department of Pediatrics, Kyungpook National University Hospital, Daegu, Korea, Republic of

**Introduction:** Children with catecholaminergic polymorphic ventricular tachycardia (CPVT) are at risk for adverse arrhythmic events causing sudden death, and a risk stratification tool remains ill defined. The purpose of this study was to evaluate predictors of disease severity in children with CPVT.

**Methods:** The study population was derived from the national multicenter cohort of inherited arrhythmia syndromes, consisting 16 pediatric cardiology centers in our country. Demographics, therapy, and outcomes were compared between patients with and without cardiac arrest at diagnosis. Also, predictors of arrhythmic events during treatment were evaluated using Cox regression models.

**Results:** Among the 350 patients with inherited arrhythmia syndrome, a total of 40 patients who were diagnosed with CPVT at ≤18 years old were included (10±3.3; 3‐17 years). Twelve patients (30%) presented with cardiac arrest; age of diagnosis (11.7±4 vs 9.3±2.7 years; p=0.035), age at symptom onset (10.3±4.3 vs 7.1±2.2 years; p=0.052), and baseline QTc (444±31 vs 416±25 ms; p=0.006) showed significant difference compared with patients without cardiac arrest at diagnosis. During a mean follow‐up of 79 months (0‐293 months), 18 (45%) occurred significant arrhythmic event (AE), defined as aborted cardiac arrest, documented ventricular tachyarrhythmias, appropriate implantable cardioverter‐defibrillator shocks, or sudden cardiac death. Induction of ventricular tachyarrhythmias during recovery of exercise test was an independent predictor of time to first AE after the initiation of therapy (p=0.015; hazard ratio=5.713). The 1‐, 3‐ and 5‐year event‐free survival rates for patients having ventricular tachyarrhythmias during recovery of exercise test were 71%, 57%, and 28%, respectively, and for patients without induced ventricular tachyarrhythmias during the period were 87%, 78%, and 72%, respectively.

**Conclusions:** Induction of ventricular tachyarrhythmias during recovery of exercise test independently predicted an earlier onset of AE. A larger sample size would enable a comprehensive investigation of this findings.

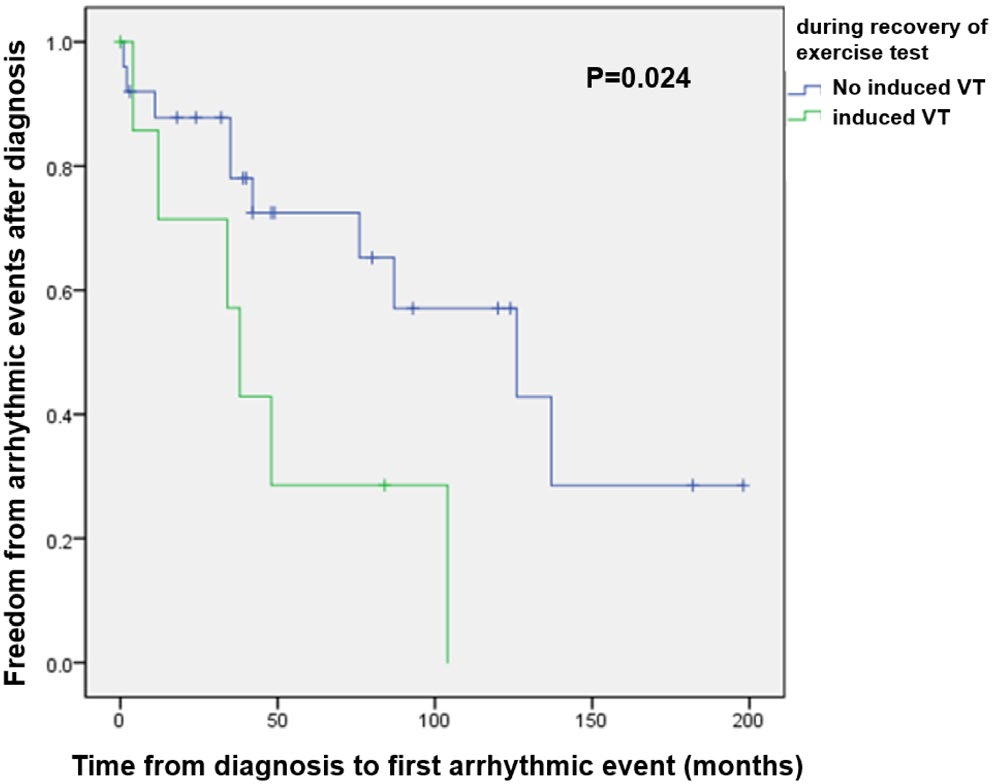

## 17 PROGNOSTIC VALUES OF EXTRA‐PULMONARY VEIN TRIGGERS AFTER PULMONARY VEIN ISOLATION IN PATIENTS WITH ATRIAL FIBRILLATION

### 17.1 **SEUNGHOON CHO**, HANJIN PARK, OH‐SEOK KWON, DAEHOON KIM, HEE TAE YU, TAE‐HOON KIM, JAE‐SUN UHM, BOYOUNG JOUNG, MOON‐HYOUNG LEE, HUI‐NAM PAK

#### 17.1.1 Severance Cardiovascular Hospital, Seoul, Korea, Republic of

**Introduction:** Extra‐pulmonary vein triggers (ExPVT) have been recognized as a significant predictor of clinical recurrence of atrial fibrillation (AF) following catheter ablation (AFCA). This study comprehensively analyzes the clinical characteristics and prognostic value of ExPVT.

**Methods:** We included 3,203 non‐valvular AF patients (men 74.5%, 59.7 ± 11.0 years old) who underwent de novo AFCA and consistent isoproterenol provocation tests after pulmonary vein isolation (PVI). We assessed the ExPVT‐associated clinical factors and their sensitivity and specificity on 2‐year AF recurrence. Subgroup analyses were conducted to examine the ExPVT‐location‐dependent prognostic value.

**Results:** ExPVT was identified in 13.4% of patients, associated with older age, female gender, more extended AF diagnosis to ablation time, higher CHA2DS2‐VASc scores, larger left atrial (LA) volumes, smaller atrial epicardial adipose tissue (EAT) volumes, and lower mean LA voltages. Notably, atrial EAT (OR 0.99 [0.98‐1.00]) and mean LA voltage (OR 0.32 [0.20‐0.52]) were independently associated with ExPVT. Diagnostic accuracy of ExPVT for 2‐year AF recurrence after AFCA shows a sensitivity of 33.6% and specificity of 77.6%. Depending on ExPVT locations, 2‐year AF recurrence rates were diverse (multiple/unmappable triggers 25.5%‐year, HR 3.06 [2.31‐4.05]; Septal trigger 15.3%‐year, HR 1.43 [1.00‐2.06]; LA trigger 15.6%‐year, HR1.42 [1.00‐2.01], and RA trigger 11.3%‐year, HR 1.10 [0.74‐1.63]). During 22.0 [IQR 10.0‐23.0] months of follow‐up, patients with multiple/unmappable triggers showed worst rhythm outcomes than others (Log‐rank p<0.001). Patients with ExPVT but no AF recurrence within 2 years were independently associated with paroxysmal AF (OR 1.63 [1.04‐2.56]), LA volumes (OR 0.98 [0.96‐1.00]), and male (OR 0.51 [0.28‐0.93]).

**Conclusions:** ExPVT after PVI has a significant prognostic value for 2‐year AF recurrence with high specificity, associated with smaller atrial EAT volume and lower LA voltage. The patients with multiple or unmappable ExPVTs showed the worst rhythm outcome than those with mappable triggers.

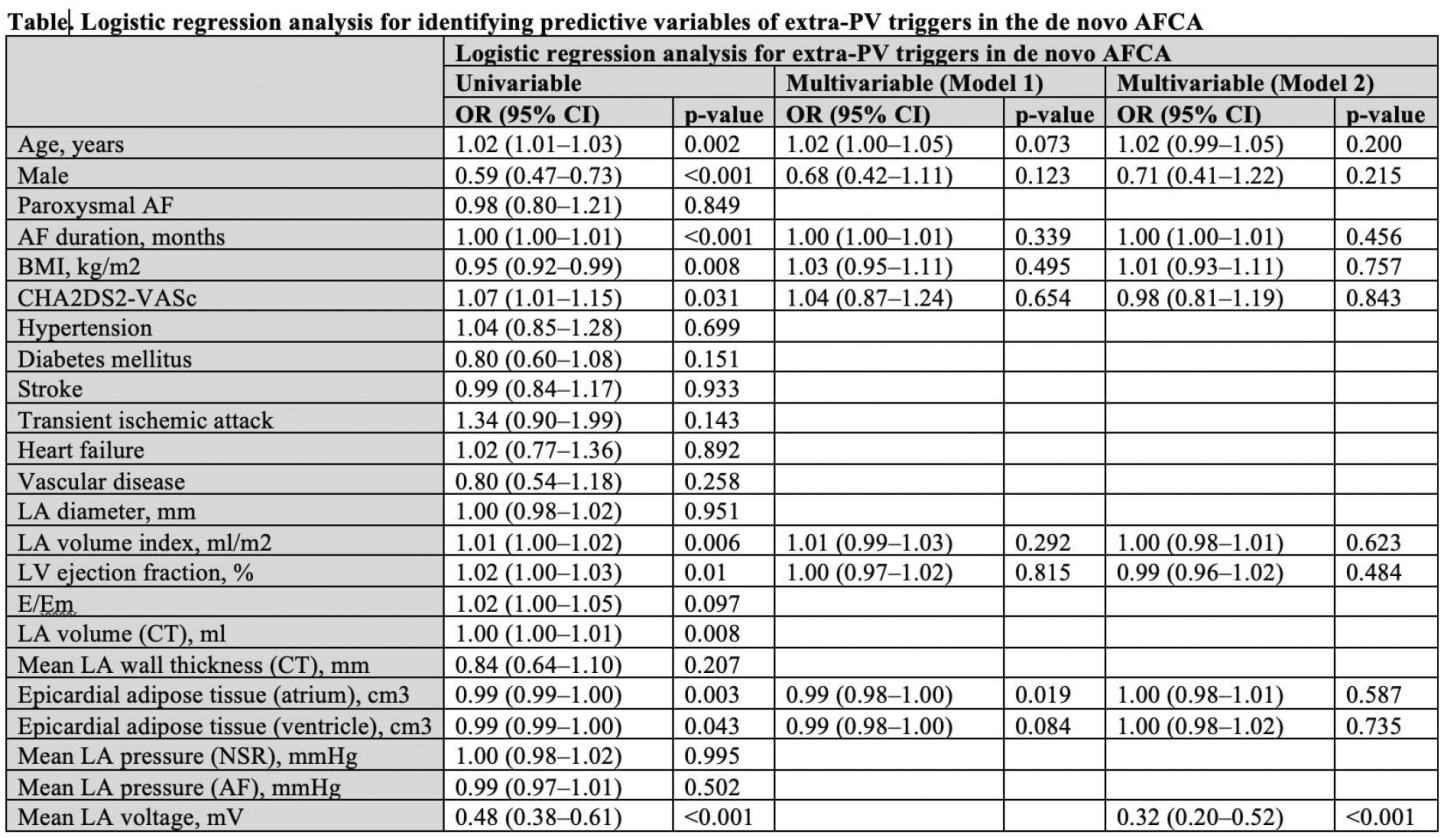

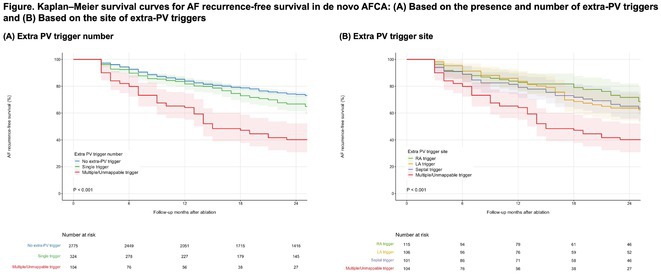

Chair

**P. Chockalingam**

Cardiac Wellness Institute, Chennai, India

## 18 PERIOPERATIVE FACTOR XA INHIBITOR DISCONTINUATION IN PATIENTS WITH ATRIAL FIBRILLATION UNDERGOING MINOR RISK INTERVENTION: THE RESULT OF PERIXA STUDY

### 18.1 **EUE‐KEUN CHOI** ^1^, SO‐RYOUNG LEE^1^, KYUNG‐YEON LEE^1^, JONG‐SUNG PARK^2^, YOUNG SOO LEE^3^, YONG SEOG OH^4^, SANG‐JIN HAN^5^, JUNE NAMGUNG^6^, JI HYUN LEE^7^, WOO‐HYUN LIM^8^, MIN SOO AHN^9^, SOONIL KWON^8^, HYO‐JEONG AHN^1^, SEIL OH^1^, GREGORY Y. H. LIP^10^

#### 18.1.1 ^1^Seoul National University Hospital, Seoul, Korea, Republic of,^2^Dong‐A University Hospital, Seoul, Korea, Republic of,^3^Daegu Catholic Univ. Medical Center, Seoul, Korea, Republic of,^4^The Catholic University Of Korea, Seoul St. Mary’S Hospital, Seoul, Korea, Republic of,^5^Hallym Univ. Medical Center, Seoul, Korea, Republic of,^6^Inje University Ilsan Paik Hospital, Seoul, Korea, Republic of,^7^Seoul National University Bundang Hospital, Seoul, Korea, Republic of,^8^Smg–Snu Boramae Medical Center, Seoul, Korea, Republic of,^9^Wonju Severance Christian Hospital, Seoul, Korea, Republic of,^10^University of Liverpool and Liverpool Heart & Chest Hospital, Seoul, Korea, Republic of

**Introduction:** Although guidelines recommend against discontinuing direct oral anticoagulants for minor risk interventions, concerns about bleeding events create a gap between guidelines and practice. This study aimed to explore the safety and efficacy of short‐term perioperative discontinuation of factor Xa inhibitors.

**Methods:** This multicenter, prospective, single‐arm registry study included patients with AF who were on factor Xa inhibitors and were scheduled for minor risk interventions. The last dose of DOACs was recommended to be taken 24 hours before the procedures and restarted the next day (**Figure 1A**). The primary safety outcome was major bleeding, and the primary efficacy outcome was a composite of stroke, transient ischemic attack, systemic embolism, and myocardial infarction 30 days after the procedure.

**Results:** The study included 1902 patients (mean age 70.4 years; 60% men; mean CHA2DS2‐VASc score 2.8; mean HAS‐BLED score 1.6). Among the minor risk interventions, 50% were endoscopies, 43% dental procedures, and 6% ophthalmic procedures (**Figure 1B**). Patients were on apixaban (48.4%), edoxaban (32.4%), or rivaroxaban (19.2%). The adherence rate to the Factor Xa interruption and resumption protocol was 84.9%. The 30‐day post‐procedural event rates for primary safety and efficacy outcomes were 0.1% and 0%, respectively (**Figure 1C**), consistent across procedure categories and Factor Xa inhibitors (**Figures 1D** and **1E**). Minor bleeding was significantly higher in the dental procedure group (4.2%) compared to endoscopy (0.7%) and ophthalmic procedures (0%), p<0.001 (**Figure 1F**). Practitioner surveys had a 60.9% return rate, with 88.1% reporting manageable bleeding, 11.5% reporting difficult but controllable bleeding, and 0.3% requiring special procedures for bleeding control.

**Conclusions:** In this study, patients with AF who were anticoagulated with factor Xa inhibitors and underwent minor risk interventions demonstrated low rates of major bleeding and thromboembolism when managed with the standardized perioperative strategy outlined in the PERIXa protocol.

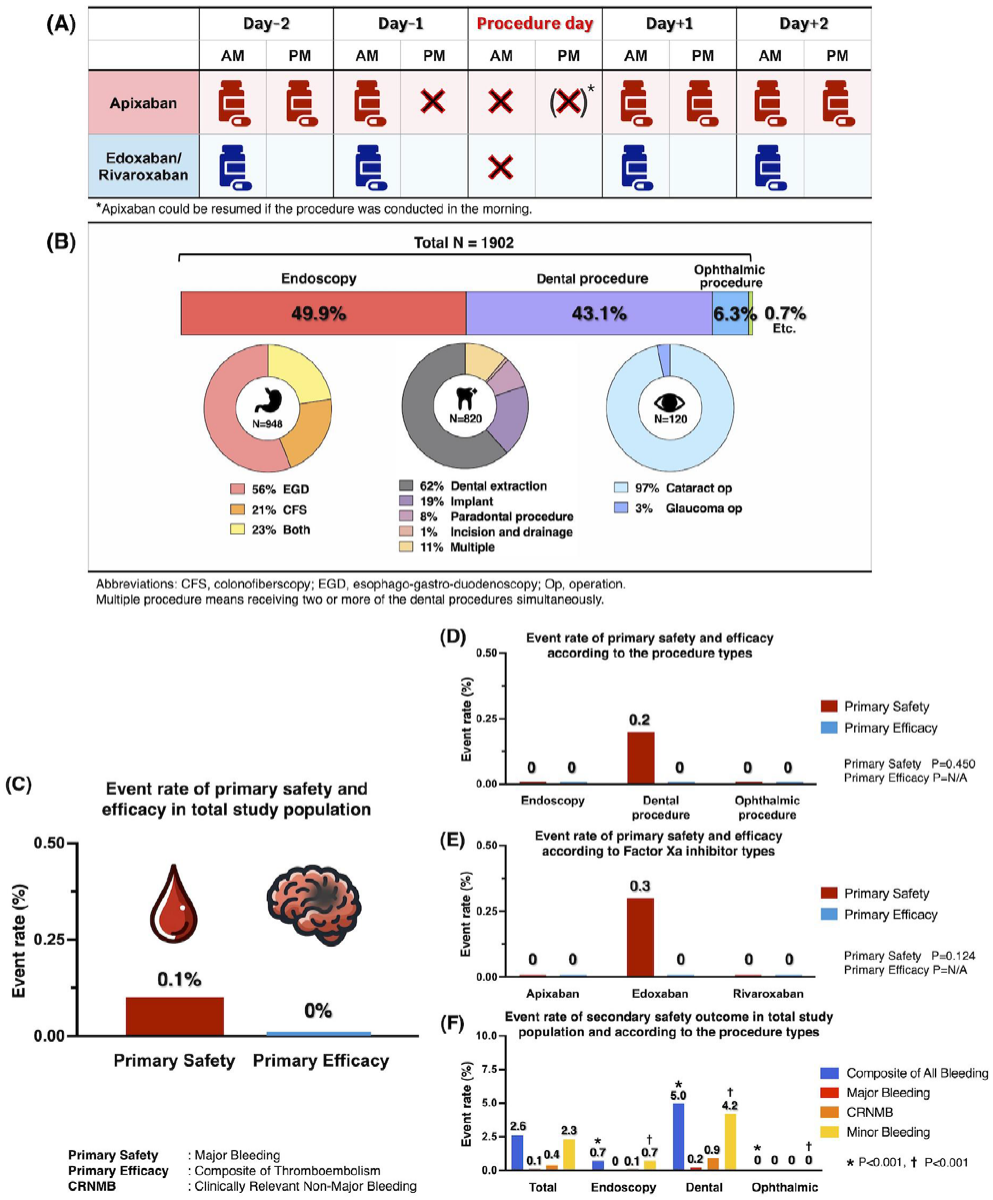

## 19 A REAL‐WORLD, PROSPECTIVE, OBSERVATIONAL STUDY OF RIVAROXABAN ON PREVENTION OF STROKE AND NON‐CENTRAL NERVOUS SYSTEMIC EMBOLISM IN RENALLY IMPAIRED KOREAN PATIENTS WITH NON‐VALVULAR ATRIAL FIBRILLATION: XARENAL

### 19.1 **JINA CHOI** ^1^, IL‐YOUNG OH^1^, CHANG HOON LEE^2^, EUE‐KEUN CHOI^3^, HONG EUY LIM^4^, YONG‐SEOG OH^5^, JONG‐IL CHOI^6^, MIN‐SOO AHN^7^, JU YOUN KIM^8^, NAM‐HO KIM^9^, NAMSIK YOON^10^, MARTIN SANDMANN^11^, KEE‐JOON CHOI^12^

#### 19.1.1 ^1^Seoul National University Bundang Hospital, Seongnam‐si, Korea, Republic of,^2^Veterans Health Service Medical Center, Seoul, Korea, Republic of,^3^Seoul National University Hospital, Seoul, Korea, Republic of,^4^Hallym University Medical Center, Anyang, Korea, Republic of,^5^Seoul St. Mary's Hospital, The Catholic University of Korea, Seoul, Korea, Republic of,^6^Korea University Medical Center, Seoul, Korea, Republic of,^7^Yonsei University Wonju College of Medicine, Wonju, Korea, Republic of,^8^Heart Vascular Stroke Institute, Samsung Medical Center, Sungkyunkwan University, Seoul, Korea, Republic of,^9^Wonkwang University Hospital, Wonkwang University, Iksan, Korea, Republic of,^10^Chonnam National University, Gwangju, Korea, Republic of,^11^ClinStat GmbH, Hürth, Germany,^12^Asan Medical Center, University of Ulsan, Seoul, Korea, Republic of

**Introduction:** A variety of real‐world studies on rivaroxaban have been conducted among patients with nonvalvular atrial fibrillation (NVAF); however, data regarding its safety for patients with renal impairment remain limited. XARENAL, a real‐world investigation, was designed to prospectively examine the safety of rivaroxaban in patients with NVAF with renal impairment, specifically those with creatinine clearance (CrCl) levels between 15 and 49 mL/min.

**Methods:** XARENAL is a single‐arm observational cohort study focusing on patients with NVAF and renal impairment. Patients were monitored approximately every three months for a year, or until 30 days after any early termination of the study. The primary endpoint was the incidence of major bleeding events. Secondary outcomes included all adverse events, symptomatic thromboembolic events, duration of treatment, and changes in renal function from the baseline.

**Results:** XARENAL recruited 888 participants across 29 different study locations. Of these, 713 (80.3%) exhibited moderate renal impairment (CrCl, 30‐49 mL/min), and 175 (19.7%) had severe renal impairment (CrCl, 15‐29 mL/min), with an average estimated glomerular filtration rate (eGFR) of 45.2 ± 13.0 mL/min/1.73 m2. The average risk scores for CHA2DS2‐VASc and HAS‐BLED were 3.3 ± 1.4 and 1.7 ± 0.9, respectively. The study observed a major bleeding incidence rate of 5.6% (6.2 events per 100 patient‐years), with fatal bleeding occurring in 0.5% of cases (0.5 events per 100 patient‐years). The average annual change in eGFR was a shift of 2.22 ± 26.47 mL/min/1.73 m2.

**Conclusions:** This study found no meaningful differences in major bleeding events or changes in renal function compared to previous studies of Rivaroxaban‐treated NVAF patients with moderate‐to‐severe renal impairment. These outcomes are deemed acceptable in clinical practice.

## 20 THE EFFICACY AND SAFETY OF NOAC IN VERY ELDERLY ATRIAL FIBRILLATION PATIENTS; DATA FROM THE KOREAN NATIONAL HEALTH INSURANCE COHORT REGISTRY

### 20.1 **SEONG HUAN CHOI** ^1^, YONG‐SOO BAEK^1^, YEONG CHAN LEE^2^

#### 20.1.1 ^1^Inha university, Incheon, Korea, Korea, Republic of,^2^Sungkyunkwan University School of Medicine, Seoul, Korea, Republic of

**Introduction:** We investigated the clinical benefit of anticoagulation with NOAC in very elderly AF patients through national healthcare insurance registry

**Methods:** Clinical data of 862,935 patients who were diagnosed with AF from 2015 to 2020 were collected for analysis. Patients under the age of 85, prior history of ICH, GI bleeding and prior prescription days of aspirin, warfarin and NOAC exceeding 90 along with follow up period less than 90 days were excluded.

**Results:** A total of 10,625 patients were eligible for analysis. Patients with OAC (HR: 0.60, 95% CI 0.53‐0.69), p<0.001) showed higher efficacy regarding systemic embolism compared to aspirin (HR: 0.84, 95% CI 0.74‐0.95, p=0.008) and no treatment group. Individual comparison of NOAC and aspirin via propensity score matching showed that patients with NOAC (HR: 0.71, 95% CI 0.61‐0.85), p<0.001) showed higher event free survival regarding systemic embolism compared to aspirin. Bleeding risk was also higher for NOAC (HR: 1.28, 95% CI 1.07‐1.56, p=0.006) group but did not result in commensurate increase in mortality (HR:0.60, 95% CI 0.45‐0.81, p<0.001)

**Conclusions:** Anticoagulation with NOAC in very elderly patient showed higher event free survival regarding systemic embolism. Despite having higher event rate of bleeding, eventual death did not show any association with NOAC.

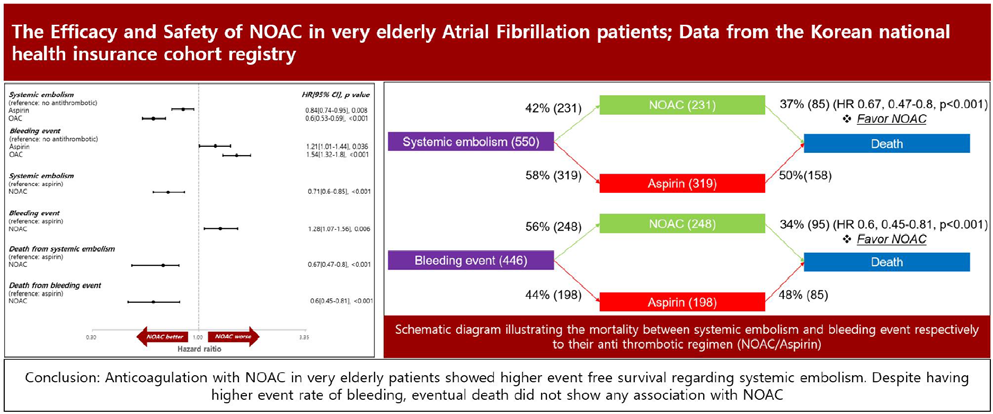

## 21 STRATEGY OPTIMIZATION FOR THE COMBINED PROCEDURE OF LEFT ATRIAL APPENDAGE OCCLUSION PLUS CATHETER ABLATION IN PATIENTS WITH ATRIAL FIBRILLATION (COMBINATION): A MULTI‐CENTER, RANDOMIZED STUDY

### 21.1 XIANFENG DU, **HUIMIN CHU**

#### 21.1.1 The First Affiliated Hospital of Ningbo University, Ningbo, China

**Introduction:** The long‐term efficacy and safety of the atrial fibrillation (AF) combined procedure have been established. However, the optimal combining strategy has not been well elucidated. We aimed to determine the impact of different combining strategies on long‐term clinical outcomes.

**Methods:** AF patients referred for the combined procedure were randomly assigned to the ablation‐first or occlusion‐first groups at a 1:1 ratio. Long‐term outcomes of LAAC with a Watchman device and AF catheter ablation were evaluated.

**Results:** A total of 194 randomized patients from 14 centers were analyzed. Baseline characteristics were comparable between groups. All procedures were accomplished with acute successful LAAC and restoration of sinus rhythm. Incidences of periprocedural complications were similar. Higher incidences of chronic peri‐device leak (PDL, 15.5% vs 5.2%, *P*=0.031) and device‐related thrombus (DRT, 8.2% vs 1.0%, *P*=0.035) were observed in the ablation‐first group. The event‐free survival rate of the primary endpoint (composite of thromboembolic events including stroke/transient ischemic attack, DRT, clinically‐relevant bleeding, cardiovascular rehospitalization/death) was significantly higher in the occlusion‐first group (83.5% vs 71.1%, log‐rank *P*=0.036, HR 0.53, 95%CI 0.29‐0.95) during a median 2.5‐year follow‐up. Patients with male gender and higher CHA_2_DS_2_‐VASc score were at lower risk in the subgroup analysis. Long‐term freedoms from AF (77.3% vs 63.5%, log‐rank *P*=0.039) and atrial tachyarrhythmias (70.1% vs 55.7%, log‐rank *P*=0.044) were higher in the occlusion‐first group, respectively.

**Conclusions:** The occlusion‐first approach is recommended for patients with atrial fibrillation in combined procedures with catheter ablation and left atrial appendage closure with a plug‐like device implanted due to the superior long‐term clinical benefits.

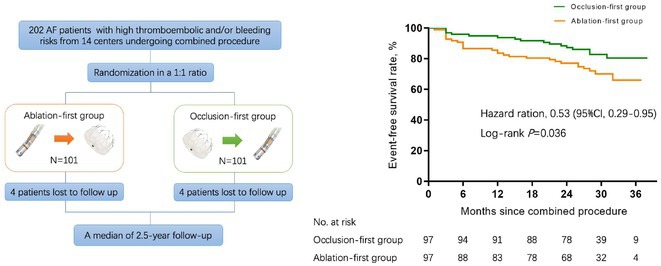

## 22 COMPARATIVE ANALYSIS OF ATRIAL FIBRILLATION CATHETER ABLATION IN ADULT CONGENITAL HEART DISEASE: PROCEDURAL INSIGHTS AND CLINICAL OUTCOMES

### 22.1 **CHIEH‐MAO CHUANG** ^1^, PI‐CHANG LEE^1^, WEN‐PO FAN^2^, YING‐HSUAN PENG^3^, I‐HSIN TAI^4^, YUN‐CHING FU^1^, YU‐CHENG HSIEH^5^, CHENG‐HUNG LI^5^, MING‐JEN KUO^5^, SHANG‐JU WU^5^, SHIH‐ANN CHEN^5^

#### 22.1.1 ^1^Division of Pediatric Cardiology, Pediatric Medical Center, Taichung Veterans General Hospital, Taichung City, Taiwan,^2^Division of Pediatric Cardiology, Department of Pediatrics, Taipei Veterans General Hospital, Taipei City, Taiwan,^3^Division of Pediatric Cardiology, Department of Pediatrics, Chung Shan Medical University Hospital, Taichung City, Taiwan,^4^China Medical University Children's Hospital, Taichung City, Taiwan,^5^Cardiovascular Center, Taichung Veterans General Hospital, Taichung City, Taiwan

**Introduction:** Atrial fibrillation (AF) represents the most prevalent arrhythmia in adult congenital heart disease (ACHD). While catheter ablation is an established rhythm control technique for AF in non‐ACHD patients, performing AF ablation in ACHD presents unique technical challenges due to complex anatomy and diffuse surgical scarring.

**Methods:** To present clinical outcomes of AF ablation in ACHD based on a single operator's experience.

**Results:** The study included 91 patients: 34 in the ACHD group and 57 in the non‐ACHD group. Demographic data were comparable between groups, except for a higher prevalence of heart failure (64.7% vs. 42.1%, p = 0.037) and pulmonary hypertension (32.4% vs. 3.5%, p < 0.001) in the ACHD group. Congenital heart disease (CHD) severity was mild in 70.6%, moderate in 23.5%, and severe in 5.9% of cases. In the ACHD group, 41.1% had paroxysmal AF, 17.7% had persistent AF, and 41.2% had long‐standing persistent AF (LsPAF), with a similar distribution in the non‐ACHD group. The ACHD group employed more radiofrequency (RF) needles and fewer BRK needles for transseptal puncture. Ablation strategies differed only in more cavotricuspid isthmus linear ablation (91.2% vs. 71.9%, p = 0.03) for ACHD. Procedure time was longer in the ACHD group (304.5 vs. 283 minutes, p = 0.03). Despite the complex anatomy in the ACHD group, the ratio of nonfluoroscopic ablation was similar between groups (58.8% vs. 63.2%). Recurrence rates (38.2% vs. 28.1%), 1‐year complete freedom from AF or atrial flutter (AFL) (65.2% vs. 75%), and 3‐year AF or AFL‐free survival (48.3% vs. 67.5%) were comparable between the two groups. ACHD patients exhibited less pulmonary vein stenosis (0 vs. 12.3%, p = 0.04).

**Conclusions:** AF ablation is effective and safe in patients with ACHD compared to those without ACHD.

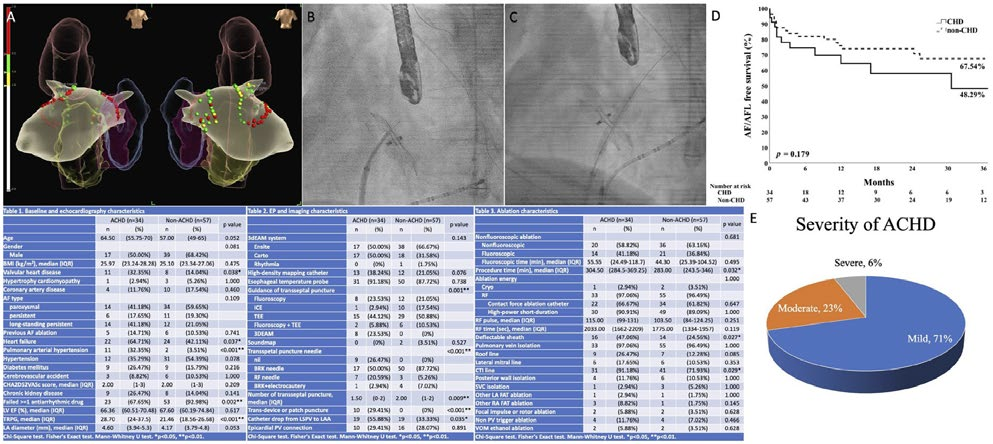

## 23 THE EXTRAVASCULAR ICD SAFELY PROVIDES EFFECTIVE ATP AND DEFIBRILLATION THROUGH LONGER‐TERM FOLLOW‐UP: FINAL PIVOTAL STUDY RESULTS

### 23.1 **IAN CROZIER** ^1^, FRANCIS MURGATROYD^2^, JAIMIE MANLUCU^3^, LUCAS VA BOERSMA^4^, BRADLEY P KNIGHT^5^, CHRISTOPHE LECLERCQ^6^, NICOLAS CLÉMENTY^7^, ANISH AMIN^8^, BÉLA PETER MERKELY^9^, ULRIKA MARIA BIRGERSDOTTER‐GREEN^10^, JOSEPH YAT SUN CHAN^11^, MAURO BIFFI^12^, REINOUD ELWIN KNOPS^13^, CHRISTOPHER WIGGENHORN^14^, PAUL FRIEDMAN^15^

#### 23.1.1 ^1^Christchurch Hospital, Christchurch, New Zealand,^2^King's College Hospital, London, United Kingdom,^3^London Health Sciences Centre, London, ON, Canada,^4^Cardiology Departments of St. Antonius Hospital Nieuwegein and Amsterdam University Medical Center, Amsterdam, Netherlands,^5^Northwestern University, Chicago, IL,^6^CHU de Rennes ‐ Hôpital Pontchaillou, Rennes, France,^7^Clinique du Millénaire, Montpellier, France,^8^Riverside Methodist Hospital, Columbus, OH,^9^Heart and Vascular Center, Semmelweis University, Budapest, Hungary,^10^University of California San Diego, San Diego, CA,^11^Prince of Wales Hospital, Chinese University of Hong Kong, Hong Kong, Hong Kong,^12^Policlinico Sant' Orsola‐Malpighi, Bologna, Italy,^13^Amsterdam University Medical Centers, Amsterdam, Netherlands,^14^Medtronic Inc, Mounds View, MN,^15^Mayo Clinic, Rochester, MN

**Introduction:** By using a substernal lead, the extravascular implantable defibrillator (EV‐ICD) provides the therapeutic benefits of transvenous ICDs, including effective pacing and defibrillation, without the complications associated with intravascular leads. The global Pivotal Study has shown that the EV‐ICD is safe and effective through 6 and 18 months of follow‐up, but longer‐term experience has yet to be reported. The objective is to assess the performance and safety of the EV‐ICD through final follow‐up.

**Methods:** The EV‐ICD Pivotal Study was a prospective, global, single‐arm, premarket clinical study. Patients who met guidelines for a class I or IIa single‐chamber ICD were enrolled. Freedom from major system‐ and/or procedure‐related complications was assessed at 3 years. The rate of appropriate therapy, inappropriate therapy, and antitachycardia pacing (ATP) success were also assessed. Cumulative rates were calculated using the Kaplan‐Meier (KM) method.

**Results:** A total of 316 patients underwent an implant attempt [74.7% male/25.3% female, 53.8±13.1 years old, 81.6% primary prevention, LVEF 38.9%±15.4%, and NYHA Class I (23.7%) or II/III (65.5%)]. Of the 299 patients with a successful implant (mean follow‐up 30.6±8.5 months), 24 experienced 82 spontaneous arrhythmic episodes that were appropriately treated with either ATP (38), shock (34), or both (10) (Figure) for a 3‐year KM‐estimated appropriate therapy rate of 9.2%. The ATP success rate through final follow‐up was 77.1% (37 of 48 episodes). ATP usage increased from discharge to 2 years, and a small proportion (2.8%) of patients reported having ATP programmed “off” due to pacing sensation at 2 years. The inappropriate shock rate at 1 year was 9.8%. Freedom from system‐ or procedure‐related major complications was 89.0% at 3 years (Figure), which is in‐line with other systems.

**Conclusions:** The EV‐ICD is safe and effective during longer‐term follow up, with a low rate of complications, high rate of ATP success at 77%, and consistently effective shocks when required.

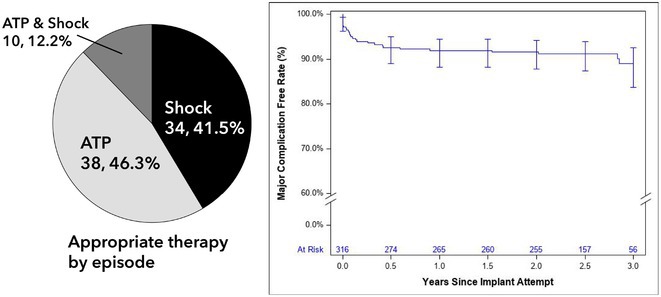

## 24 PULSED RADIOFREQUENCY OF STELLATE GANGLION: A NOVEL MINI‐INVASIVE THERAPY FOR REFRACTORY VENTRICULAR ARRHYTHMIAS

### 24.1 **CHANG CUI**, ZHOU XU, SHU YANG, MINGLONG CHEN

#### 24.1.1 The First Affiliated Hospital of Nanjing Medical University, Nanjing, China

**Introduction:** Previous study showed that stellate ganglion (SG) blockade was effective for the treatment of refractory ventricular arrhythmias (VA). However, long‐term modulation of SG with mini‐invasive strategy is still warranted. Pulsed radiofrequency (PRF) of SG was applied to treat the chronic facial pain, while its effect on the VAs has not been reported. This study investigates the effect of SG PRF in myocardial infarction (MI) induced VA animal models and assesses its long‐term efficacy in patients with refractory electrical storms (ES).

**Methods:** 48 Sprague‐Dawley rats were randomly divided into control, MI, MI + PRF groups. To evaluate the acute and chronic effects of SG PRF, heart rate variability and serum concentrations of angiotensin II and noradrenaline were obtained before PRF, immediately after the PRF, after 1 week after the PRF. Peripheral sympathetic neural activity was assessed by measuring left stellate ganglion neural activity at each time point. At 2 weeks after the PRF, echocardiography, electrocardiographic recording and programmed electrophysiological stimulation (EPS) were used to test the vulnerability of VAs. Finally, hearts and SGs were extracted for histopathological and bulk RNA‐seq analysis, respectively. In addition, the efficacy of SG PRF was evaluated in 4 patients with refractory ES.

**Results:** In animal experiments, PRF significantly decreased the serum angiotensin II and noradrenaline concentration, and LF/HF power at 1 week. Moreover, MI induced SG activity was significantly attenuated by the PRF at 1 week. Compared with the MI group, the LVEF was significantly increased and EPS induced VAs were significantly decreased in the MI + PRF group at 2 weeks after the PRF. In addition, PRF resulted in a significant reduction of TH positive cells and expressions of inflammatory cytokines in the SG. In the clinical pilot study, 4 patients suppressed ES after SG PRF therapy; however, 1 patient had a recurrence of VAs at 3 months after PRF.

**Conclusions:** SG PRF reduced sympathetic activity by attenuating the inflammatory response, which may be a novel mini‐invasive adjunctive therapy to control refractory VAs.

## 25 UNDERSTANDING HOW PHOSPHORYLATION AND REDOX MODIFICATIONS REGULATE CARDIAC RYANODINE RECEPTOR‐TYPE 2 ACTIVITY TO PRODUCE AN ARRHYTHMOGENIC PHENOTYPE IN ADVANCED HEART FAILURE

### 25.1 **ALEXANDER DASHWOOD** ^1^, NICOLE BEARD^2^, HARIS HAQQANI^1^, YEE WENG WONG^1^, MELANIE SPRATT^3^, ELIZABETH CHEESMAN^1^, PETER MOLENAAR^1^

#### 25.1.1 ^1^The University of Queensland, Brisbane, Australia,^2^University of Canberra, Canberra, Australia,^3^Queensland University of Technology, Brisbane, Australia

**Introduction:** In heart failure (HF), oxidative and phosphorylative stress can cause post‐translational modifications of cardiac ryanodine receptor type 2 (RyR2), contributing to ventricular arrhythmias through aberrant diastolic calcium cycling.

**Methods:** Using ventricular trabeculae from patients undergoing orthotopic cardiac transplantation, our study examined the effects of oxidative (50μM H_2_O_2_) and phosphorylative (10μM (‐)‐noradrenaline) stress on diastolic calcium cycling and ventricular arrhythmogenesis, and whether carvedilol could reverse these effects. Ventricular trabeculae were subjected to in‐vitro arrhythmia protocols with or without oxidative and phosphorylative stress. Subsequently, tissues were analysed for aberrant RyR2 modifications and RyR2 channel activity. The same experiments were repeated in the presence of absence of carvedilol.

**Results:** Seventeen patients donated their hearts for the study. Both oxidative and phosphorylative stress were necessary to produce aberrant diastolic RyR2 calcium release (Fig 1A), which was associated to the highest number of observed spontaneous beats (Fig 1B). Pre incubation with carvedilol normalised RyR2 activity in diastolic calcium conditions under stress (Fig 1C) and prevented spontaneous contractions (Fig 1D).

**Conclusions:** Adrenergic receptor mediated phosphorylation, combined with oxidative stress, has synergistic adverse effects on diastolic calcium cycling through harmful modifications of RyR2 in failing human hearts. Carvedilol, with its broad adrenoceptor blockade and potent antioxidant properties, consistently reduced spontaneous contractions and normalised RyR2 activity, underscoring its therapeutic potential in HF management. These findings highlight the significance of stress‐induced RyR2 modifications in ventricular arrhythmogenicity and may represent novel therapeutic targets.

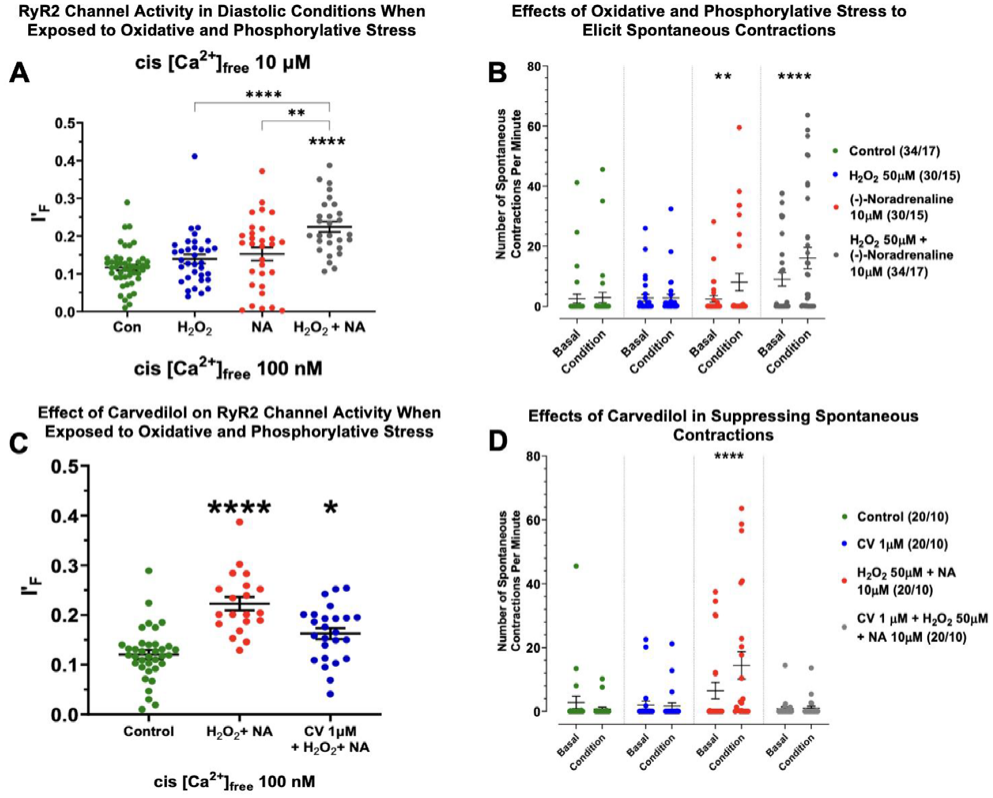

## 26 WHOLE HEART HISTOLOGICAL AND ELECTROANATOMIC ASSESSMENT OF POST INFARCTION CMR SCAR AND CONDUCTING CHANNELS

### 26.1 **KASUN DE SILVA** ^1^, TIMOTHY CAMPBELL^1^, RICHARD BENNETT^1^, ROBERT ANDERSON^2^, CHRIS DAVEY^1^, ALEXANDRA O'DONOHUE^3^, AARON SCHINDELER^3^, SAMUAL TURNBULL^1^, ASHWIN BHASKARAN^1^, YASUHITO KOTAKE^1^, CHI‐JEN HSU^1^, JAMES CHONG^1^, EDDY KIZANA^1^, SAURABH KUMAR^1^

#### 26.1.1 ^1^Westmead Hospital, Sydney, Australia,^2^Royal Melbourne Hospital, Melbourne, Australia,^3^The Childrens Hospital at Westmead, Sydney, Australia

**Introduction:** Cardiac magnetic resonance imaging (CMR) defined ventricular scar and anatomical conduction channels (CMR‐CCs) offer promise in delineating ventricular tachycardia substrate. No studies have validated channels with co‐registered histology nor have they ascertained the histological characteristics of deceleration zones (DZs) within these channels. We aimed to validate CMR scar and CMR‐CCs with whole‐heart histology and electroanatomic mapping (EAM) in a post‐infarction model.

**Methods:** 5 sheep underwent anteroseptal infarction. CMR (116 ± 20 days post‐infarct) was post‐processed using ADAS 3D, varying pixel intensity thresholds (5545, 6040, 6535 and 7030). DZs were identified by EAM (129 ± 12 days post‐infarct). Explanted hearts were sectioned and stained with Picrosirius red and whole‐heart histopathological shells generated. Scar topography as well as percentage fibrosis, adiposity and remaining “viable myocardium” within 3mm histological biopsies and within CMR‐CCs were determined.

**Results:** Using the standard 6040 thresholding, CMR had 83.8% accuracy for identifying histological scar in the endocardium (kappa 0.666) and 61.4% in the epicardium (kappa 0.276). Thirty‐seven CMR‐CCs were identified by varying thresholding; 23 (62%) were unique. DZs co‐localised to 19/23 (83%) of CMR‐CCs. Twenty (87%) CMR‐CCs were histologically confirmed. Within‐channel histological fibrosis did not differ by presence of DZs (P=0.873). Within‐channel histological adiposity was significantly higher at sites with vs without DZs (24.1% vs. 8.9%, P=0.002).

**Conclusions:** Post‐processed CMR derived scar and channels were validated by histology and EAM. Regions of CMR‐CCs at sites of DZs had higher adiposity but similar fibrosis than regions without DZs suggesting that lipomatous metaplasia may contribute to arrhythmogenicity of post‐infarction scar.

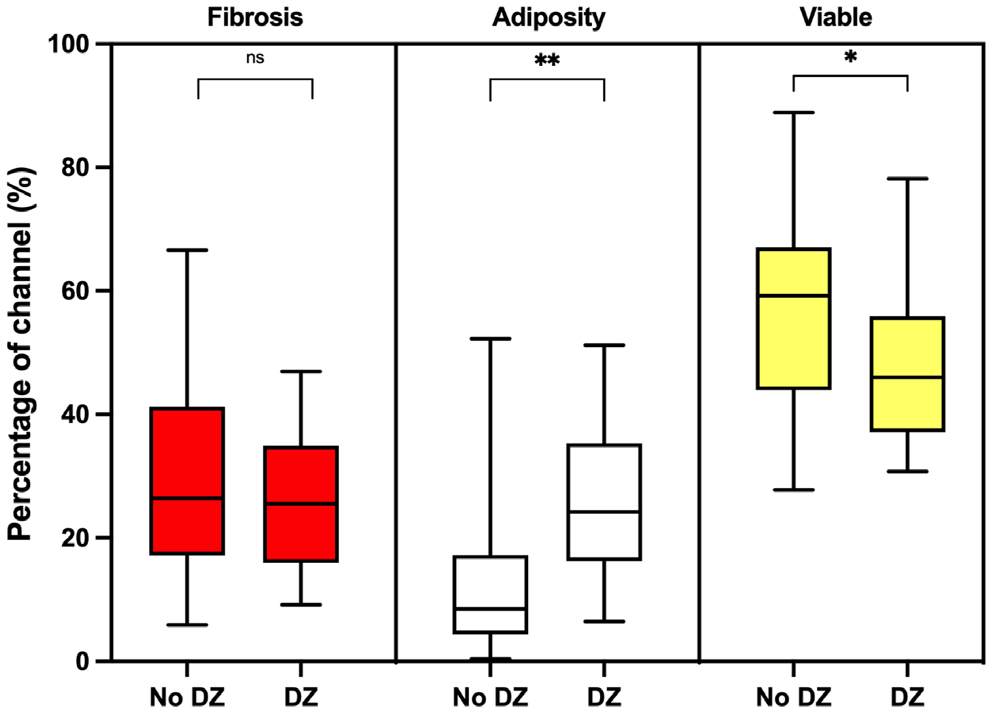

## 27 HEMODYNAMIC CHARACTERIZATION AND ANATOMICAL RECONSTRUCTION OF BACHMANN’S BUNDLE IN A VOLUME OVERLOADED IN VIVO SWINE MODEL AND CONTRAST‐ENHANCED MICRO‐CT

### 27.1 **NEAL DUONG**, PAUL IAIZZO

#### 27.1.1 University of Minnesota, Minneapolis, MN

**Introduction:** The Bachmann's bundle (BB), the predominant conduction pathway within the atria, is crucial in coordinating atrial activation and ensuring synchronous contraction. Increased filling pressure and valve regurgitation can prompt atrial adaptations. Traditional right atrial appendage (RAA) pacing can lead to P‐wave lengthening, pacemaking‐induced arrhythmias, and the risk of perforation. Atrial conduction system pacing at BB has been associated with reduced atrial fibrillation incidence, lower risk of perforation, and potential hemodynamic benefits. Despite this, hemodynamic effects and structural characteristics of the aligned myocytes within BB remain elusive.

**Methods:** 3 swine were subjected to volume overload via intravenous infusion of 3 liters of 0.9% saline solution. A CD Laycom catheter was inserted retrograde into the left ventricle to measure pressure and volume. Medtronic 3830 pacing leads were placed in the RAA and BB. Rates were alternated between the two sites at 110, 120, and 130 bpm, with the acquisition of pressure‐volume loops. Anatomies were formalin‐fixed and treated with a 7.5% iodine solution to delineate contractile myocardium. The specimens underwent scanning using an NSI x3000 microCT machine and visualized within the eFX software.

**Results:** Our findings revealed atrial synchrony with BB pacing compared to RAA pacing. BB pacing demonstrated a reduction in average P‐wave duration by 30 ms, accompanied by increased stroke volume across pacing speeds (23%, 17%, and 15% at 110, 120, and 130 bpm, respectively). Micro‐CT scans depicted representative orientations of the Bachmann's Bundle.

**Conclusions:** Through this study, we have achieved a comprehensive hemodynamic characterization and anatomical reconstruction of Bachmann's bundle in an in vivo swine model subjected to volume overload. The hemodynamic implications of BB pacing relative to RAA pacing underscore the potential of BB pacing as an alternative site for atrial pacing. This understanding of atrial physiology may inform clinical interventions aimed at optimizing cardiac pacing strategies.

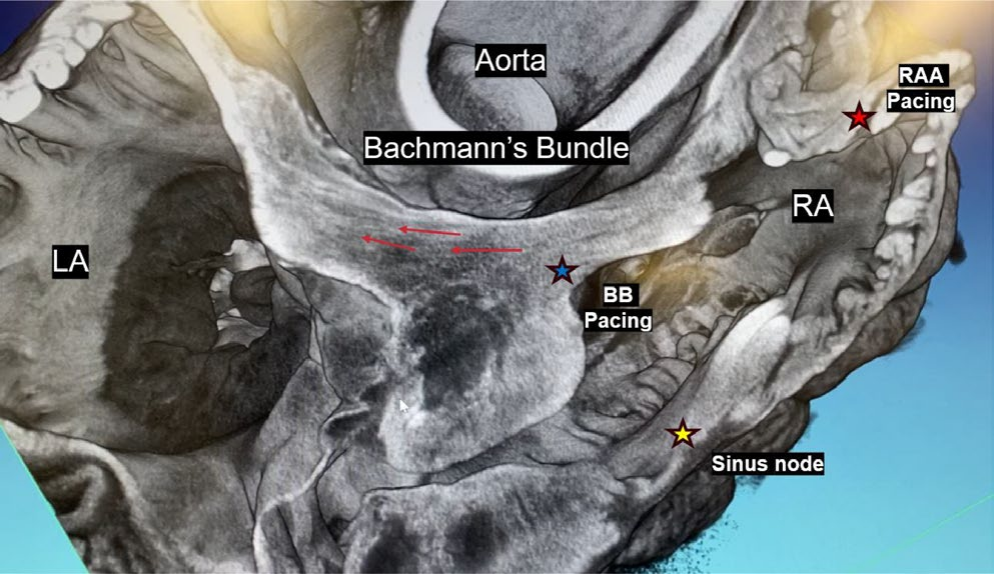

Chair

**A. Elliott**

University of Adelaide, Australia

## 28 ELECTROGRAM‐DERIVED CURRENT OF INJURY IN ATRIAL LEADLESS PACEMAKERS PREDICT CHRONIC PACING CAPTURE THRESHOLDS

### 28.1 **DEREK EXNER** ^1^, ERIC JOHNSON^2^, VIVEK REDDY^3^, JAMES IP^4^, RAHUL DOSHI^5^, PASCAL DEFAYE^6^, ROBERT CANBY^7^, MARIA GRAZIA BONGIORNI^8^, MORIO SHODA^9^, GERHARD HINDRICKS^10^, CHRISTIE HUFF^2^, LEYLA SABET^2^, KENNETH BRUHNS^2^, REINOUD KNOPS^11^

#### 28.1.1 ^1^Foothills Medical Center, Calgary, AB, Canada,^2^Abbott Medical, Sylmar, CA,^3^Icahn School of Medicine at Mount Sinai, New York, NY,^4^Weill Cornell Medicine/ New York Presbyterian Hospital, New York, NY,^5^HonorHealth Cardiac Arrhythmia Group, Scottsdale, AZ,^6^CHU Grenoble Alpes, Grenoble, France,^7^Texas Cardiac Arrhythmia Institute, Austin, TX,^8^San Rossore Private Hospital and Medical Center, Pisa, Italy,^9^Tokyo Women's Medical University, Tokyo, Japan,^10^German Heart Center of the Charite, Berlin, Germany,^11^Amsterdam UMC, Amsterdam, Netherlands

**Introduction:** Current of injury (COI) has been used in transvenous lead implants to identify robust tissue fixation and settlement of acute pacing capture thresholds (PCT). Mapping and tether capability in the novel atrial leadless pacemaker (aLP) may allow acute evaluation of COI to further characterize underlying myocardial tissue and optimize aLP implant site selection. This study investigated the intraprocedural COI amplitude from atrial electrograms (EGMs) and their correlation with chronic PCT.

**Methods:** Retrospective analyses were performed on atrial implant and follow‐up data from the Aveir DR i2i Study (NCT#:05252702). Patients with atrial electrograms (EGM) during implant (mapping, tether, and post‐release), and PCT at 3 months were included. EGMs were extracted from device session records, and COI amplitude was identified using a custom algorithm in MATLAB (R2021b). Variables are reported as mean±SD. Student's t‐test or ANOVA was used to determine differences in means.

**Results:** 61 patients were included. COI amplitude at mapping, tether, and release were 2.9±1.9 mV, 3.1±2.0 mV, and 2.4±1.3mV, respectively. Tether COI amplitude was significantly higher than release (p=0.03). In univariate linear regression, COI in tether and release were significant predictors of 3‐month PCT (p=0.02, p<0.01, respectively). In multivariate linear regression, release COI remained a significant predictor of 3‐month PCT (p=0.02). Tether COI ≤2.25mV significantly predicted higher PCT at 3‐months (1.1±.9V) vs. tether COI >2.25mV (0.7±0.7V, p<0.05) Similarly, lower COI at release (COI ≤2.25mV) resulted in higher PCT at 3‐months (1.1±1.0V) vs. release COI >2.25mV (0.5±0.2V, p<0.01).

**Conclusions:** Intraprocedural COI during aLP implant was correlated with chronic capture thresholds. Atrial tissue responsive to an aLP device, with increased COI amplitude during the implant, may indicate favorable implant locations resulting in lower PCT. Further research will be critical in identifying optimal COI targets and throughout the implant process, in addition to considering additional implant characteristics such as impedance.

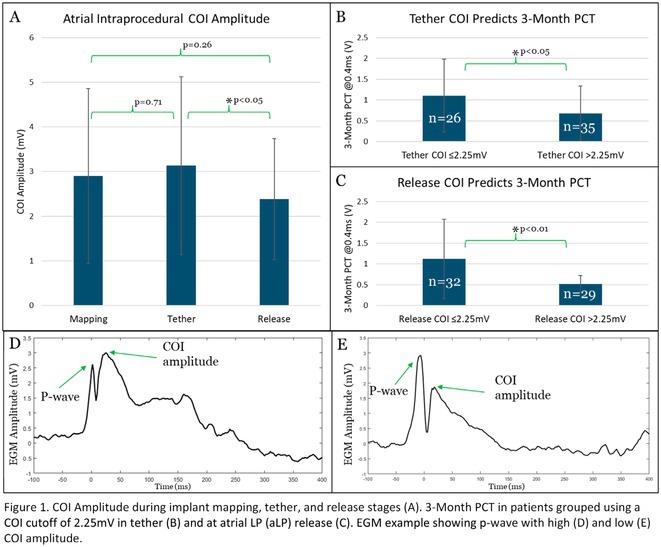

Chair

**A. Ghani**

Malaysia

## 29 VARIATION IN PACEMAKER BATTERY LONGEVITY

### 29.1 **JAMES FREEMAN** ^1^, MICHAEL TORRE^2^, PRASHANTHAN SANDERS^3^, NIRAJ VARMA^4^, TINA BAYKANER^5^, THOMAS DEERING^6^, ANDREA RUSSO^7^, YUE ZHANG^8^, BENJAMIN STEINBERG^9^

#### 29.1.1 ^1^Yale School of Medicine, New Haven, CT,^2^University of Utah, Salt Lake City, UT,^3^University of Adelaide, Adelaide, Australia,^4^Cleveland Clinic, Cleveland, OH,^5^Stanford University School of Medicine, Stanford, CA,^6^Piedmont Heart Institute, Atlanta, GA,^7^Cooper Medical School of Rowan University, Camden, NJ,^8^University of Utah School of Medicine, Salt Lake City, UT,^9^Denver Health and University of Colorado, Denver, CO

**Introduction:** Pacemaker battery longevity impacts the need for generator replacement, which is associated with a substantial risk of complications, and may be an important distinguishing characteristic among devices. We compare the battery longevity and factors associated with battery longevity across different pacemaker types and manufacturers.

**Methods:** We evaluated the battery longevity between implant and observed or device estimated time of replacement index (RI). We compared Kaplan‐Meier (K‐M) survival curves, and device parameters, including mean lifetime lead outputs, pulse widths, pacing percentages, lifetime shocks delivered, and shocks aborted across manufacturers. Finally, we performed Cox proportional hazard regression to measure battery longevity by device type adjusting for device manufacturer and parameters.

**Results:** We evaluated 7190 single‐chamber, 42731 dual‐chamber, and 3429 biventricular devices. Each additional lead resulted in approximately 2‐3 years of lost battery longevity compared with a single‐chamber pacemaker. Among devices triggering replacement, Biotronik had longest median battery longevity for single chamber pacemakers (Biotronik [BIO] 3717 days, Medtronic [MDT] 3582, St Jude/Abbot [STJ] 3559, BSX 3180; p<0.001); MDT for dual chamber (MDT 3840 days; BIO 3788, STJ 3475, and BSX 3137; p<0.001); and MDT for biventricular pacemakers (MDT 2820 days, BSX 2389, STJ 2389, BIO 2313; p<0.001). Figure shows all devices and manufacturers with censoring at loss to follow‐up. Device parameters, including lead output amplitude, pulse width, impedance, and pacing percentage varied substantially across manufacturers. With inclusion of all devices and after adjustment for mean lifetime lead outputs, pulse widths, and pacing percentages, Biotronik had longest single‐ and dual‐chamber longevity, and BSX had the longest biventricular battery (p<0.001 for each across group).

**Conclusions:** Pacemaker battery longevity varies by device type and by manufacturer, and may be influenced by programming and/or pacing demands. Further analyses are needed to understand how to prospectively optimize longevity for these devices.

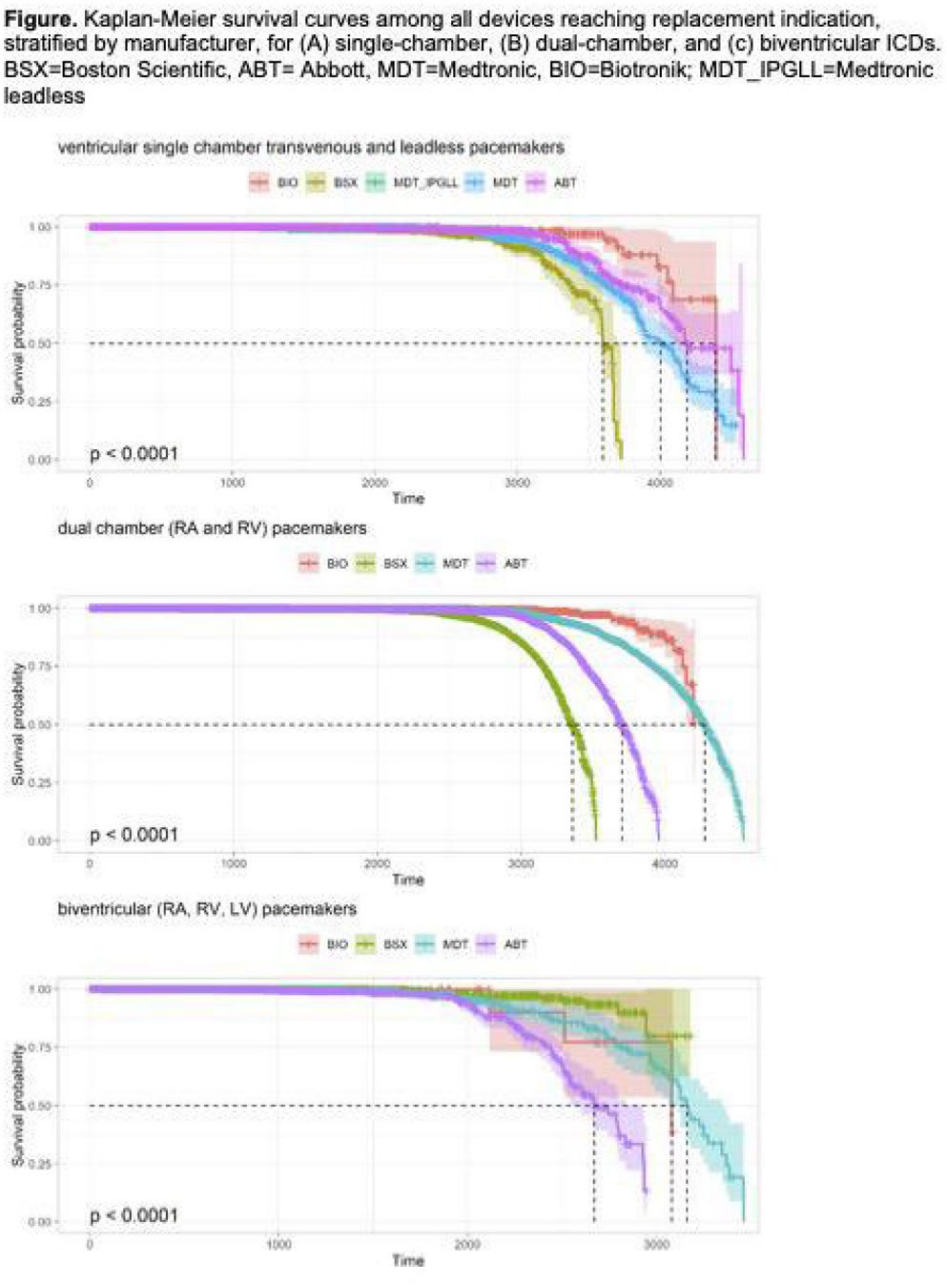

## 30 BIPOLAR RADIOFREQUENCY ABLATION OF REFRACTORY VENTRICULAR ARRHYTHMIAS: RESULTS FROM A NETWORK SUPPORTED BY EHRA SCIENTIFIC INITIATIVES COMMITTEE

### 30.1 **PIOTR FUTYMA** ^1^, STEFANO BORDIGNON^2^, LUKASZ ZAREBSKI^1^, ARIAN SULTAN^3^, GURAM IMNADZE^4^, MARIA KOUSTA^5^, SVEN KNECHT^6^, NIKOLA PAVLOVIC^7^, PETR PEICHL^8^, EVGENY LYAN^9^, THOMAS KUEFFER^10^, DANIEL SCHERR^11^, PAWEL MOSKAL^12^, GABRIEL CISMARU^13^, BOR ANTOLIč^14^, BORIS SCHMIDT^2^, JAKOB LÜKER^3^, PHILIPP SOMMER^4^, CHRISTIAN STICHERLING^6^, VERA MASLOVA^9^, TOBIAS REICHLIN^10^, ANDRES ENRIQUEZ^15^, HELMUT PÜRERFELLNER^5^, JONAS WÖRMANN^3^, JULIAN KR CHUN^2^

#### 30.1.1 ^1^St. Joseph's Heart Rhythm Center, Rzeszów, Poland,^2^Cardioangiologisches Centrum Bethanien, Frankfurt, Germany,^3^Heart Center at the University of Cologne, Cologne, Germany,^4^Herz‐ und Diabeteszentrum NRW, Bad Oeynhausen, Germany,^5^Ordensklinikum Linz Elisabethinen, Linz, Austria,^6^University Hospital Basel, Basel, Switzerland,^7^University Hospital Dubrava, Zagreb, Croatia,^8^Institute for Clinical and Experimental Medicine, Prague, Czech Republic,^9^Cardiovascular Center, University Clinic Schleswig‐Holstein, Kiel, Germany,^10^Inselspital, Bern University Hospital, University of Bern, Bern, Switzerland,^11^Department of Medicine, Medical University of Graz, Graz, Austria,^12^1st Department of Cardiology, Interventional Electrocardiology, and Hypertension, Jagiellonian University Medical College, Krakow, Poland,^13^University of Medicine and Pharmacy of Cluj Napoca, Rzeszów, Romania,^14^University Medical Centre of Ljubljana, Ljubljana, Slovenia,^15^Clinical Electrophysiology, Hospital of the University of Pennsylvania, Philadelphia, PA

**Introduction:** Advanced ablation strategies are needed to effectively treat ventricular tachycardia (VT) and premature ventricular contractions (PVC) refractory to standard unipolar ablation (UA). Bipolar ablation (BA) has emerged as a treatment option for refractory VT, PVC or complex ventricular substrate in general.

**Methods:** The study group consists of consecutive patients undergoing BA at 14 European centers for recurring VT/PVC after at least one standard UA. A second ablation catheter was used instead of a dispersive patch and positioned at the opposite site of the ablation target.

**Results:** Between March 2021 and July 2024 eighty‐five consecutive patients underwent a total of 87 BA procedures (69 males, age 62±13, number of prior UA 1.6±1.2; range 1‐7). The main indication for ablation was recurrence of frequent PVC (n=54), VT (n=17), electrical storm (n=12) or PVC‐triggered ventricular fibrillation (n=2). In seventy‐three procedures, a combined UA+BA approach was used. Procedural time was 161±75min, BA time was 421± 292s (power 33±8W) and UA time was 828±721s (power 44±8W). Elimination of clinical VT/PVC was achieved in 65 (76%) patients, while significant suppression of VT/PVC was observed in further 8 (10%) patients. In 12 patients (14%) no effect on VT/PVC was observed. Concomitant alcohol ablation was performed in three patients. There were 3 (4%) major complications: coronary artery injury, anticipated AV block and arteriovenous fistula. Follow‐up lasted 7±8 months. Two patients with VT storm and advanced‐stage heart failure died within the first week after BA. Fifteen (56%) out of remaining 27 VT patients remained VT‐free during follow‐up, including 5 on antiarrhythmic drugs (AAD). ≥80% PVC burden reduction was achieved in 43 (77%) out of 56 PVC patients, including 5 on AAD.

**Conclusions:** BA is currently adapted as an advanced ablation strategy in the setting of refractory and recurrent PVC and in patients with complex VT substrate. This real‐world registry data indicates that BA of refractory VT/PVC is feasible, safe and effective in majority of patients with recurrent ventricular arrhythmias.

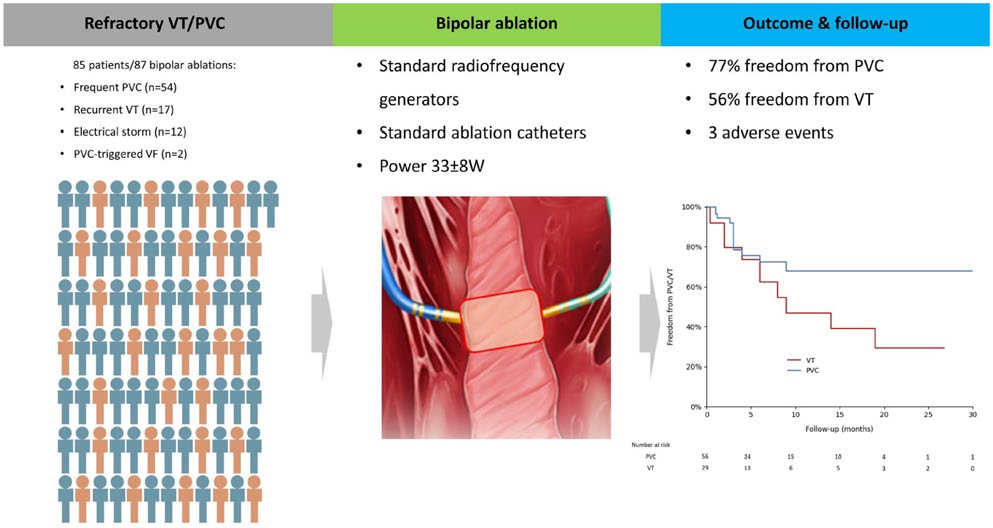

## 31 THE DEVELOPMENT, VALIDATION, AND COMPARISON OF MACHINE LEARNING ALGORITHMS TO PREDICT COMPLICATIONS ASSOCIATED WITH ATRIAL FIBRILLATION ABLATION

### 31.1 **MOHAMED GAD** ^1^, MATTHEW SEGAR^1^, JERIN GEORGE^1^, HANNAH SMATI^1^, AJIT KODURI^1^, IRAKLI GIORGBERIDZE^1^, HAMID AFSHAR^1^, JOANNA ESTHER MOLINA RAZAVI^1^, ABDI RASEKH^1^, PAUL SCHURMANN^2^, MOHAMMAD SAEED^1^, MIHAIL G. CHELU^1^, MEHDI RAZAVI^1^

#### 31.1.1 ^1^Baylor College of Medicine, HOUSTON, TX,^2^Houston Methodist Hospital, HOUSTON, TX

**Introduction:** Periprocedural complications following ablation can be associated with serious mortality and morbidity. In this study, we aimed to develop, validate, and evaluate the performance of machine learning (ML) models to aid in predicting periprocedural complications.

**Methods:** Patients diagnosed with AFib who underwent catheter ablation were identified in the Nationwide Readmission Database (NRD) from 2016 to 2019. The primary outcome was in‐hospital mortality following AFib ablation. The secondary outcomes were 1) acute peri‐procedural stroke, 2) the development of tamponade or effusion requiring pericardiocentesis, and 3) vascular and bleeding complications. The identified patients were split randomly into a training dataset (70%) and a validation and testing dataset (30%). Scikit‐learn's Python library was used. Difference algorithms were compared, including K nearest neighbors, Logistic regression, Stochastic Gradient Descent, Naive Bayes, Decision Tree Classifier, Random Forest, and Gradient Boosting Classifier, and the best performing algorithm was used. The tuning parameters were as follows: number of trees 200, maximum depth 10, and criterion "gini." Model performance was evaluated using AUC, accuracy, precision, and specificity. The analysis was conducted using Python 3.6.9.

**Results:** A total of 112,000 patient records were identified. For in‐hospital mortality, the model had an AUC of 0.901, accuracy of 0.989, precision of 0.900, and specificity of 0.999 (Figure 01‐A). For stroke, the model had an AUC of 0.741, accuracy of 0.991, precision of 0.599, and specificity of 0.999 (Figure 01‐B). For tamponade, the model had an AUC of 0.812, accuracy of 0.980, precision of 0.999, and specificity of 0.999 (Figure 01‐C). For vascular and bleeding complications, the model had an AUC of 0.691, accuracy of 0.864, precision of 0.846, and specificity of 0.999 (Figure 01‐D).

**Conclusions:** Our study highlights that using ML models based on EMR to predict adverse events following catheter ablation for atrial fibrillation is feasible with high success in predicting outcomes.

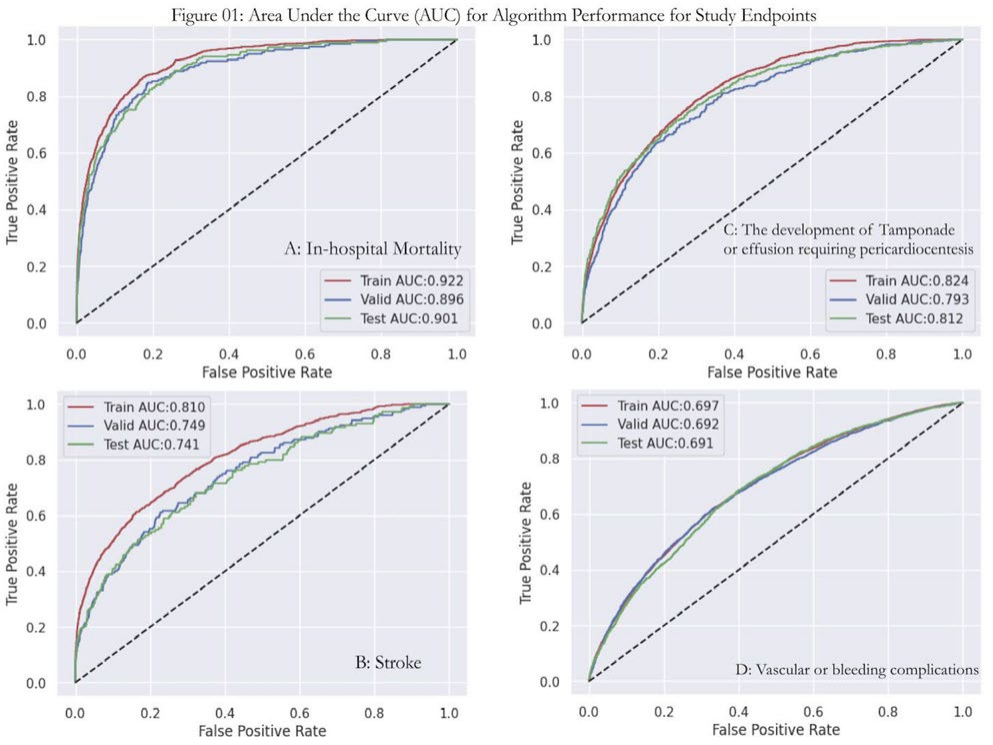

## 32 FIRST‐IN‐HUMAN SERIES OF PERMANENT LEFT BUNDLE BRANCH AREA DEFIBRILLATOR IMPLANTATION‐SAFETY, FEASIBILITY AND SHORT‐TERM FOLLOW‐UP

### 32.1 **ANINDYA GHOSH**, ULHAS M PANDURANGI

#### 32.1.1 Madras Medical Mission, Chennai, India

**Introduction:** Use of Left bundle branch area pacing (LBBAP) leads as part of DF1‐ LB optimized‐ICDs where they serve the pace‐sense function has recently gained popularity with proven efficacy. However, the same renders the device MRI incompatible because of redundant lead port. Defibrillator lead at the LBBA preserves MRI compatibility of the device and has previously been tested as proof‐of‐concept with a single case report of a permanent implant.

**Methods:** We prospectively attempted implantation of a 7F Durata single coil lead at the LBBA using a compatible fixed‐curve non‐deflectable CPS locator delivery sheath (Figure 1) using standard criteria and methods. All parameters including sensing and defibrillation with DFT testing were noted. The study also includes 1‐ month follow‐up evaluation.

**Results:** A total of 10 patients (M‐80%, LVEF‐ 33%, IHD in 4, Primary prevention indication in 6) underwent an attempt at LBBA defibrillator implantation. LBBAP pacing was successfully achieved in 8/10 with permanent implant possible in 7/10. DFT testing was done in all and when induced, sensing was adequate in 100%. Baseline clinical demographic and procedural characteristics are shown in Figure 1. Fluoroscopic image during RV angiogram performed using 6F pigtail catheter from the groin shows depth of the implanted lead in Figure 1

**Conclusions:** Implantation of a 7F defibrillator lead at LBBA was safe and feasible with favorable 1 month‐follow‐up status. Further data at longer follow‐up intervals is awaited.

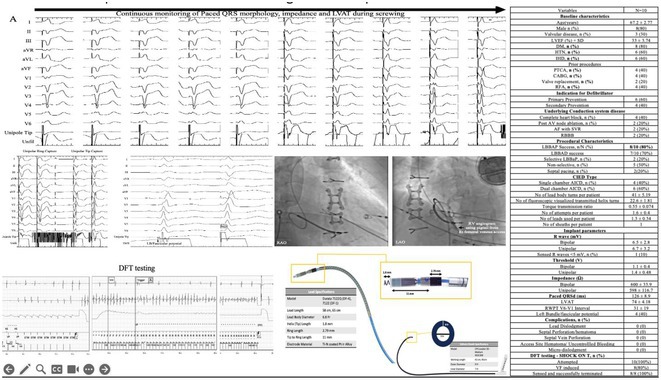

Chair

**K. Gwynne**

## 33 FIRST‐IN‐HUMAN EXPERIENCE FROM THE RESET‐AF TRIAL: THE 6 AND 12‐MONTH OUTCOMES OF PVI USING A NOVEL SINGLE‐SHOT PULSED FIELD ABLATION SYSTEM

### 33.1 **PAUL GOULD** ^1^, STUART THOMAS^2^, WAHEED AHMAD^1^, PIERRE QIAN^2^, ARASH ARYANA^3^

#### 33.1.1 ^1^Princess Alexandra Hospital, Brisbane, Australia,^2^Westmead Hospital, Sydney, Australia,^3^Mercy General Hospital, Sacramento, CA

**Introduction:** The *R* apid On *E* ‐ *S* hot *E* lectroporation *T* rial for *A* trial *F* ibrillation (RESET‐AF) is a first in human study to evaluate the efficacy and safety of using a novel, single‐shot Pulse Field Ablation (PFA) system to treat AF.

**Methods:** RESET‐AF is a prospective, non‐randomized trial at 2 centers including 36 patients with paroxysmal (n=22) or persistent (n=14) AF. PFA was given to each pulmonary vein (PV), (1 to 2 treatments) without repositioning using a bipolar, biphasic electric fields (>2kV) and QRS‐gated PFA system through a spiral, 8.4‐Fr, 16‐electrode mapping/ablation catheter (ElePulse, CRC EP) to isolate PV (PVI). 3D mapping validated acute PVI. Additional applications were allowed to achieve PVI. Patients were grouped in 3 cohorts (high (1), low (2) and medium (3) dose). All patients underwent re‐mapping at 3 months and patients with AF within the 3‐month blanking period could have radio‐frequency re‐ablation. The primary efficacy and safety outcomes included acute PVI, PVI durability at 3 months, freedom from AF at 6 and 12 months, and safety at 7 and 30 days.

**Results:** Acute PVI was achieved in 100% using 2.1 ± 0.6 applications/PV (procedure: 128 ± 36 min, ablation: 6.1 ± 1.8 min, LA dwell: 63 ± 31 min, fluoroscopy: 23 ± 20 min). There was no PV stenosis, TIA, phrenic nerve/esophageal injury, perforation/tamponade, stroke or death. One patient with preexisting chronic kidney disease developed acute kidney injury that fully resolved. PVI durability at 3 months was confirmed in 92%, 55% and 94% of the PVs in cohorts 1, 2 and 3, respectively. Freedom from recurrent AF at 6 months was 97% (35/36) based on serial ECG and Holter. 19 patients reached the 1‐year follow‐up timeline. 18 (95%) were free from AF. Regardless of dose, 100% of patients that had all their PVs isolated at 3 months remained free from AF at 6 and at 12 months

**Conclusions:** The RESET‐AF trial achieved all safely and 6 month efficacy end‐points in 36 patients and is trending very well for 12 month efficacy with a 97% and 95% freedom from AF at 6 and 12 months . This was associated with a 94% PVI durability validated at a 3 month repeat mapping study in the optimal treatment cohort

## 34 MULTI‐CENTER REAL‐WORLD EXPERIENCE WITH THE FIRST DUAL‐CHAMBER LEADLESS PACEMAKER SYSTEM

### 34.1 **CYRUS HADADI** ^1^, KEN W. LEE^2^, MONICA LO^3^, MAYER RASHTIAN^4^, ATHANASIOS THOMAIDES^1^, NEBU ALEXANDER^1^, ZAYD ELDADAH^1^, NIMA BADIE^5^, DEVI G. NAIR^6^

#### 34.1.1 ^1^MedStar Heart & Vascular Institute, MedStar Washington Hospital Center, Washington, DC,^2^Health First Medical Group, Melbourne, FL,^3^Arkansas Heart Hospital, Little Rock, AR,^4^Huntington Hospital, Pasadena, CA,^5^Abbott, Sunnyvale, CA,^6^St. Bernards Healthcare, Jonesboro, AR

**Introduction:** The Aveir™ DR pacemaker (Abbott) is a recently released, dual‐chamber leadless pacemaker (LP) system. Aveir DR includes right atrial and right ventricular helix‐fixation LPs (ALP, VLP), with beat‐to‐beat atrioventricular synchrony via wireless communication between the two devices. The initial, multi‐center commercial implant experience of the Aveir DR dual‐chamber LP system has yet to be evaluated.

**Methods:** Patients to be implanted with a de novo Aveir DR in the US after commercial release were consecutively included in this study. Procedural characteristics were evaluated, and electrical parameters were measured during pre‐fixation mapping, post‐fixation tether mode, after LP release, and prior to patient discharge. Any acute procedure‐ or device‐related complications within 30 days were noted.

**Results:** Patients were implanted with Aveir DR per standard practice (N=114 from 5 centers; 72±10 years; 62% male; 65% sinus node dysfunction, 35% AV block). ALPs were implanted predominantly in the base of the right atrial appendage (84%), VLPs in the right ventricular septum (92%). Pre‐fixation mapping allowed repositioning to be avoided in 87% of ALPs and 85% of VLPs. The total procedure duration was 71±25 min, from initial incision to final suture. In both LPs, capture thresholds improved from tether to release and again by discharge, and sensed amplitudes improved from release to discharge (see figure). Acute complications were noted in 1.8% of patients (1 ALP and 1 VLP dislodgement within 6 months of commercial release), with no perforations.

**Conclusions:** The initial real‐world experience of the helix‐fixation, dual‐chamber LP system demonstrated safe and efficient implantation with clinically acceptable electrical metrics and minimal acute complications, both occurring early after commercial release.

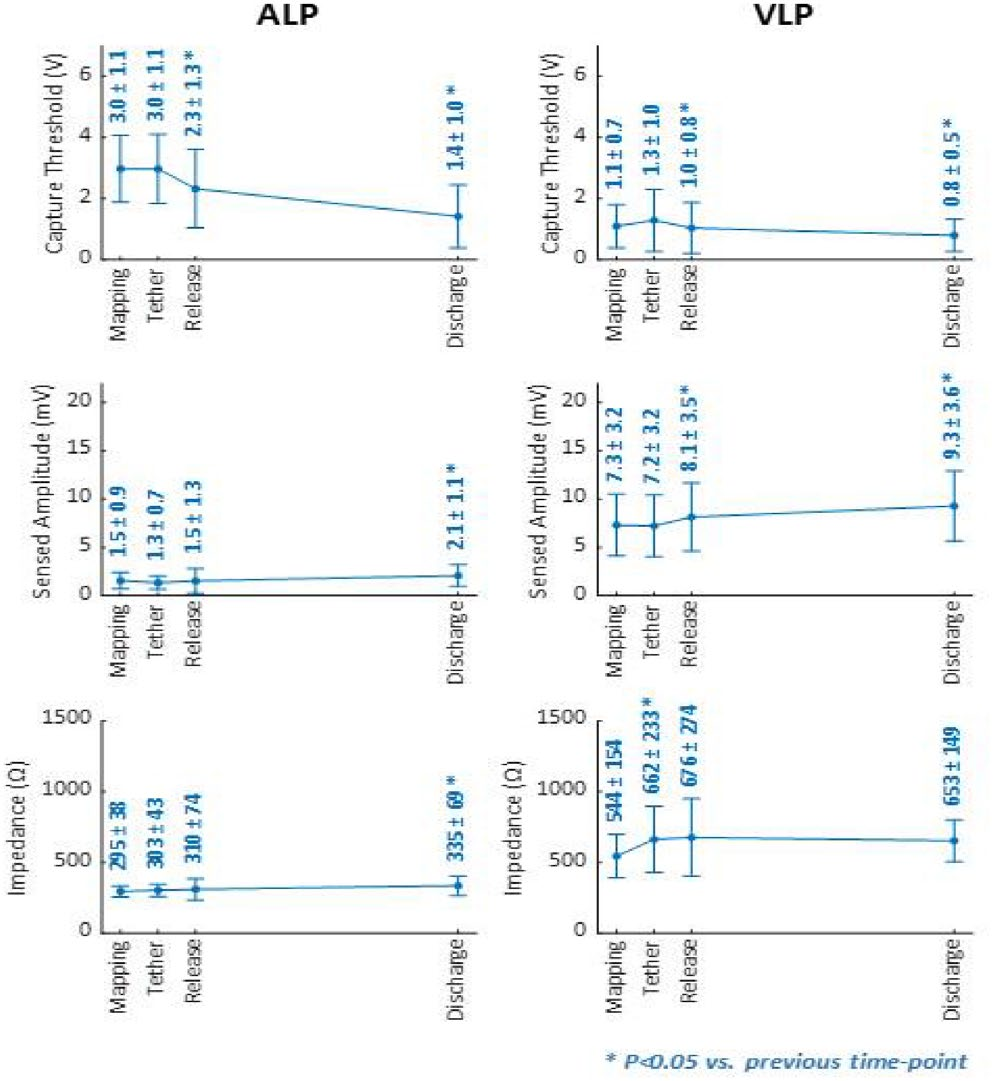

## 35 FILTERED IMPEDANCE DROP ESTIMATES LESION SIZE AND CONDUCTION GAPS OF PULMONARY VEIN ISOLATION IN ATRIAL FIBRILLATION

### 35.1 **MASAHIDE HARADA**, YUJI MOTOIKE, YOSHIHIRO NOMURA, ASUKA NISHIMURA, KOKI MATSUO, HIDEO IZAWA

#### 35.1.1 Fujita Health University, Toyoake, Japan

**Introduction:** Generator impedance drop is utilized to estimate lesion formation during radiofrequency (RF) application. However, the impedance signals usually oscillate due to cardiac beating and respiration, reducing the accurate estimation of lesion formation. The latest 3‐dimensional mapping system provides a novel module (EnSite X^TM^ Aid Module, Abbott) to eliminate the oscillation artifact by filtering original impedance signals (Averaged Impedance Drop, AID, Figure A), which may improve the accuracy. However, there is no parameters to estimate lesion size in EnSite X+TactiFlex^TM^ based‐PVI. We examined whether AID estimates lesion size and conduction gaps (CGs) of PVI in atrial fibrillation (AF) patients.

**Methods:** PVI was performed in 13 AF patients. Lesions were created using standard‐power long‐duration ablation (SPLD, 30‐40W, 20‐30sec, target contact force 10‐20g) or high‐power short‐duration ablation (HPSD, 45‐50W, 10‐15 sec, target contact force 10g). Input and output parameters in all RF points (AutoMarks) were measured and compared between points with and without CG after first‐pass ablation.

**Results:** 1108 AutoMark points from 26 pulmonary veins were evaluated; CGs were observed in 32 points (CG[+]) whereas not in 1076 points (CG[‐]). In input/output RF parameters, CG[+] showed significantly lower RF energy (J, p=0.005), smaller generator impedance drop (Ω, p=0.044), absolute AID (Ω, p<0.001), and percentage AID (%, p<0.0001) than CG[‐]; percentage AID (%AID) showed the best statistical significance. Percentage of decrease of bipolar voltage amplitude after RF application were linearly correlated with %AID (R2=0.79, p<0.05). In ROC curve analysis, the best cut‐off value of %AID to estimate CG was 9.05% (Figure C). In ex‐vivo experiments, %AID was linearly correlated with lesion size (R2=0.71, p<0.05). %AID‐guided PVI (≥9%) could successfully achieve first pass isolation without CGs.

**Conclusions:** %AID would estimate lesion size and CG in PVI and may improve the usability of impedance drop as an endpoint for RF application. %AID‐guided ablation (≥9%) may ameliorate the acute success rate of PVI in AF. ablation.

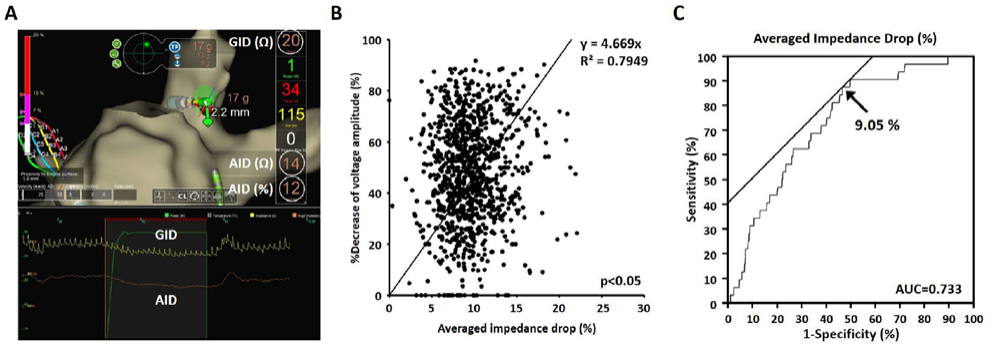

## 36 STEREOTACTIC ABLATIVE RADIOSURGERY OF RECURRENT VENTRICULAR TACHYCARDIA IN STRUCTURAL HEART DISEASE‐ A PRELIMINARY RESULTS OF STAR VT RANDOMIZED TRIAL

### 36.1 **JANA HASKOVA** ^1,2^, DAN WICHTERLE^1^, PETR PEICHL^1^, LUKAS KNYBEL^3^, RADEK NEUWIRTH^4,5^, OTAKAR JIRAVSKY^4^, MAREK SRAMKO^1^, JAKUB CVEK^3^, JOSEF KAUTZNER^1^

#### 36.1.1 ^1^IKEM, Institute for Clinical and Experimental Medicine, Prague, Czech Republic,^2^Palacký University Medical School, Olomouc, Czech Republic,^3^Department of Oncology, University Hospital Ostrava, Ostrava, Czech Republic,^4^Podlesí Hospital Trinec, Trinec, Czech Republic,^5^Department of Cardiology, Masaryk University Medical School, Brno, Czech Republic

**Introduction:** The study was designed to compare the efficacy of stereotactic arrhythmia radiotherapy (STAR) and repeated radiofrequency catheter ablation (RFCA) for drug‐refractory ventricular tachycardia (VT) in patients with structural heart disease (SHD) who already underwent at least one failed RFCA in the expert centre (STAR VT trial; NCT04612140).

**Methods:** Between June 2020 and January 2024, patients were recruited in 2 centres and randomized to STAR or repeated RFCA in a 1:1 fashion by the covariate‐adaptive algorithm. During the STAR (CyberKnife, Accuray), a dose of 25 Gy was delivered to cover at least 95 % of the planned target volume which was delineated by a co‐registration of electroanatomical map of the arrhythmogenic substrate (CARTO 3, Biosense Webster) with the planning computed tomography scan. Repeated RFCA was performed according to corresponding guidelines and included epicardial ablation if applicable. Recurrence of sustained VT and repeated ablation for VT were study endpoints.

**Results:** A total of 22 patients (77 % males, aged 67±11 years, 27 % ischemic cardiomyopathy, LVEF 29±6 %, 3.1±1.3 previous RFCA) were enrolled (11 in each arm) and followed for 18±12 months. Patients after STAR compared to RFCA had non‐significantly (P = 0.12) higher risk of VT recurrence and significantly (P <0.01) higher risk of repeated ablation for VT (Figure). There were 8 and 2 crossovers from the STAR and RFCA arm, respectively. In addition, patients after STAR vs. RFCA required non‐significantly (P = 0.20) more repeated ablation procedures (1.1±1.3 vs. 0.7±1.3, respectively).

**Conclusions:** The study suggests that patients with the recurrence of SHDrelated VT after several RFCA procedures still benefit more from repeated RFCA compared to STAR

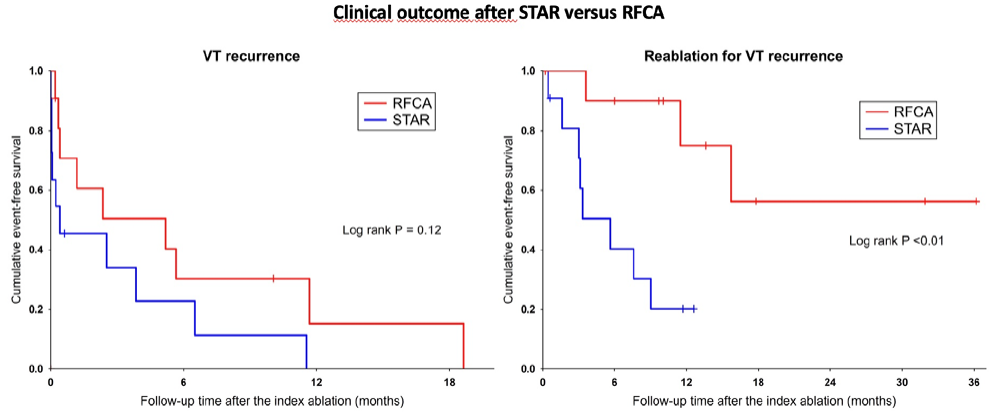

## 37 CONTINUOUS HIGH POWER SHORT DURATION ABLATION OF PULMONARY VEIN VIA FIXED TIME AND APPROPRIATE CONTACTFORCE RANGE

### 37.1 **WANG HONGTAO**, LI WENNA, SUN YANG, FAN BOYUAN

#### 37.1.1 The Second Affiliated Hospital of Xi’an JiaoTong University, xi'an, China

**Introduction:** Interrupted high power short duration (HPSD) guided by ablation index (AI) was widely used in pulmonary isolation. However, continuous HPSD strategy seemed more effective, and the clinical outcome was still unclear.This study was therefore designed to investigate the efficiency and safety of continuous HPSD ablation.

**Methods:** A total of 300 patients were randomly assigned to two different groups for treatment. Group I underwent the traditional interrupted HPSD protocol, with a AI value of 400,500,450 at posterior wall,anterior wall and other sites respectively at a power of 50W;while Group II received treatment using the continuous HPSD protocol followed by an fixed ablation time of 10sec(7‐14g),15sec(12‐20g),12sec(9‐17g) at the same sites above respectively.

**Results:** In Group II,AI ranged from 380‐420,480‐520,430‐470 respectively that approximate to that of Group I. But the average ablation time was 24 minutes, which was significantly shorter than the 37.5 minute duration in Group I (*P*<0.001). The total procedure time was also shorter in the continuous group, taking only 130 minutes compared to 146 minutes in the interrupted group. Additionally, the fluoroscopy time and radiation dose were shorter and lower in Group II (14.5 minutes vs. 17 minutes, *P*<0.001; 12mGy vs. 13mGy, *P*=0.004).Atrial arrhythmia recurrence appeared more frequent in Group I (25.3% vs. 14%, *P* =0.014).

**Conclusions:** Continuous HPSD strategy with a suitable contactforce range and fixed time for each point is superior to the traditional interrupted HPSD ablation in shortening ablation time and decreasing recurrence rate, allowing quicker procedures with lower fluoroscopy dose.

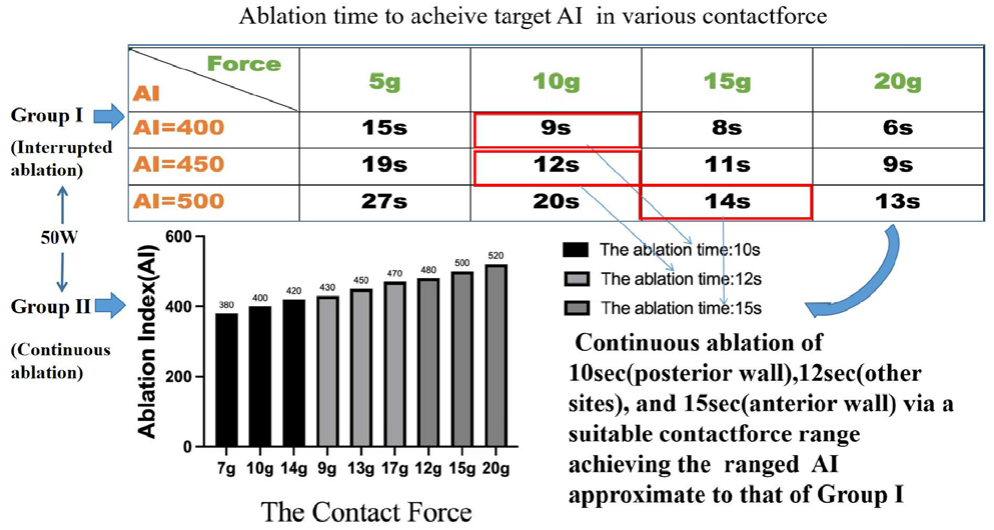

## 38 THE PREVALENCE OF HEART FAILURE WITH PRESERVED EJECTION FRACTION AND LEFT ATRIAL REMODELLING IN EMBOLIC STROKE

### 38.1 **JACKSON HOWIE** ^1,2,3^, JOHN FITZGERALD^1,2,3,4^, JENELLE DZIANO^1,2,3^, JONATHAN ARIYARATNAM^1,2,3,4^, ELNAZ SHAHMOHAMADI^1,2,3,4^, MOHANARAJ JAYAKUMAR^1,2,3,4^, MELISSA MIDDELDORP^1,2^, PRASHANTHAN SANDERS^1,2,3,4^, ADRIAN ELLIOTT^1,2,3^

#### 38.1.1 ^1^University of Adelaide, Adelaide, Australia,^2^Centre for Heart Rhythm Disorders, Adelaide, Australia,^3^SAHMRI, Adelaide, Australia,^4^Royal Adelaide Hospital, Adelaide, Australia

**Introduction:** Atrial fibrillation (AF) is a common cause of stroke and is strongly associated with the development of heart failure with preserved ejection fraction (HFpEF). Left atrial disease is commonly considered as central to the pathophysiology of these conditions. This study aims to explore the prevalence of HFpEF and left atrial remodelling amongst patients with embolic stroke.

**Methods:** Patients aged 18 ‐ 85 years with an embolic stroke or an transient ischemic attack were prospectively recruited. Assessment of HFpEF was performed according to the HFA‐PEFF diagnostic algorithm, including echocardiographic measures of left ventricular (LV) filling pressure, left atrial (LA) volume, LV size and function, and NT‐proBNP. Exercise intolerance was objectively measured with cardiopulmonary exercise testing. Quality of life and physical activity were recorded using the EQ‐5D and IPAQ.

**Results:** In total, 140 patients (61% male, mean age 74 ± 8.6 years) were included. Forty‐one (29%) met the criteria for HFpEF based on an HFA‐PEFF score of >5. Of those, HFpEF patients had enlarged LA volumes (p<0.001), reduced septal e’ (p=0.001), reduced lateral e’ (p=0.017), increased LV end‐diastolic diameter (P=0.025), and elevated NT‐proBNP (p<0.001). There was no between group difference in VO2peak (p=0.761), EQ‐5D (p=0.862), IPAQ (p=0.786) and comorbidities.

**Conclusions:** We show that 29% of patients meet objective evidence of HFpEF based on echocardiography and biomarker assessment. Patients with HFpEF have greater LA volume, reduced LV filling pressure and increased LV size. These findings show that HFpEF and LA remodelling is prevalent in patients following embolic stroke, despite the absence of AF.

Chair

**Y.‐F. Hu**

Taiwan

## 39 EFFECTIVENESS OF LEFT BUNDLE BRANCH AREA PACING FOR CARDIAC RESYNCHRONIZATION THERAPY IN PATIENTS WITH PACEMAKER UPGRADE: A HEAD‐TO‐HEAD COMPARISON WITH BIVENTRICULAR PACING

### 39.1 **HAO HUANG**, WEI HUA

#### 39.1.1 Fuwai Hospital, National Center For Cardiovascular Diseases, Peking Union Medical College, Beijing, China

**Introduction:** Left bundle branch area pacing (LBBAP) has developed as a strategy for cardiac resynchronization therapy (CRT) upgrade. We aimed to investigate the potential nuance and influencing factors in treatment effects between LBBAP and traditional biventricular pacing (BiVP) among these population.

**Methods:** Consecutive patients for CRT upgrade were retrospectively enrolled from 2011‐2023 in a tertiary hospital. Clinical symptoms, electrocardiographic and echocardiographic response, and follow‐up cardiovascular events were compared between LBBAP and BiVP groups.

**Results:** Totally, 90% patients had ventricular pacing rhythm with an average baseline QRS duration (QRSd) of 184ms. 50% had both clinical and echocardiographic response. Compared with BiVP, LBBAP had similar improvements in left ventricular ejection fraction (LVEF), left ventricular end‐diastolic dimension (LVEDD), and NYHA class, while achieved narrower QRSd (147.9±23.3 ms vs. 158.3±24.8 ms; P=0.048). Higher QRSd reduction was associated with better LVEF improvement in LBBAP group (P<0.001), but not in BiVP group (P=0.221). LBBAP yielded a better LVEF improvement than BiVP only when QRSd reduction was more than 29ms. Corrected QRSd, LVEF improvement, reserved kidney function, and spironolactone use were independently associated with lower mortality. After 1:1 propensity score matching for follow‐up time between two groups, LBBAP also had similar rates of all‐cause mortality and heart failure hospitalization.

**Conclusions:** LBBAP is a feasible alternative to BiVP for resynchronization in patients with indication of pacemaker upgrade, which exhibits superiority over BiVP in echocardiographic response only when QRS duration was largely shortened. Narrower paced QRSd, better LVEF improvement, higher estimated glomerular filtration rate (eGFR), and spironolactone use were independent protective factors of survival.

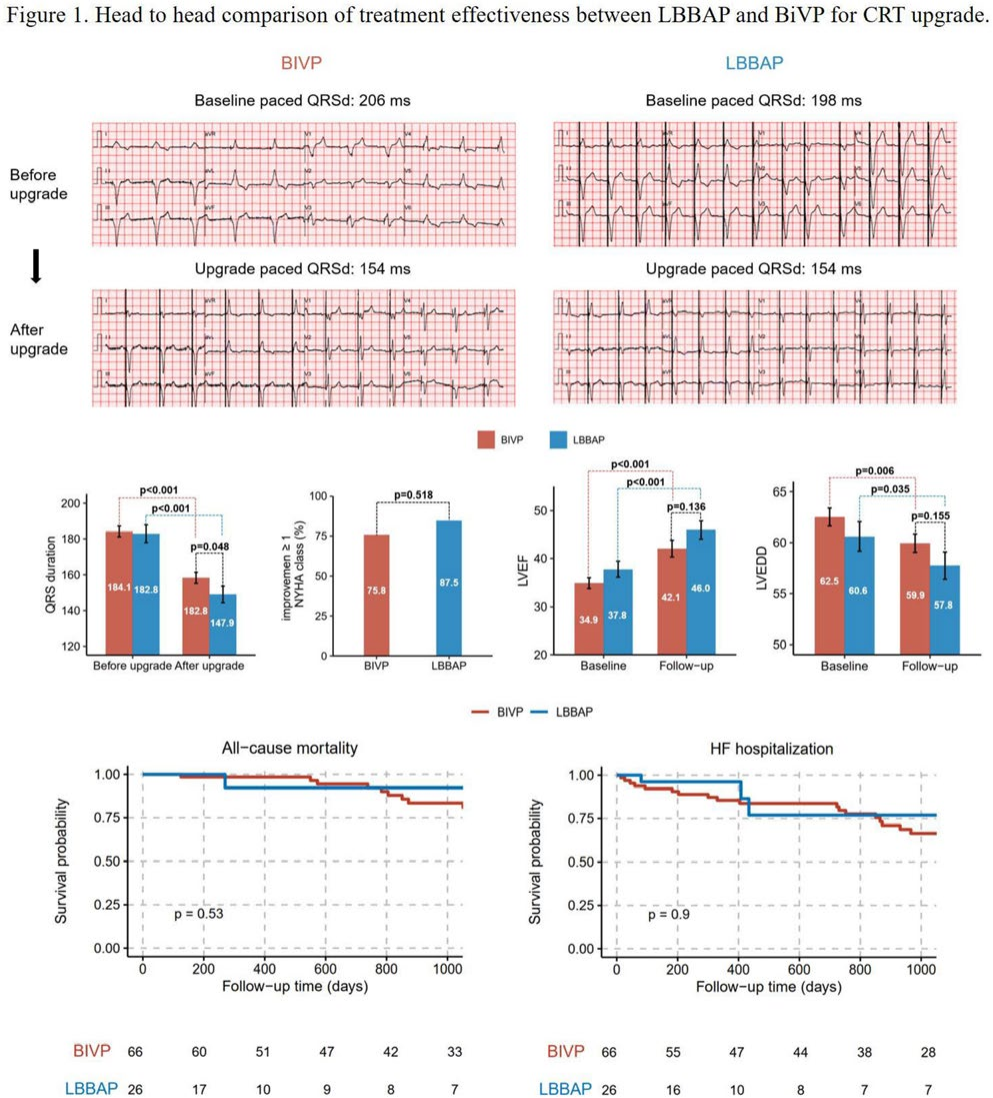

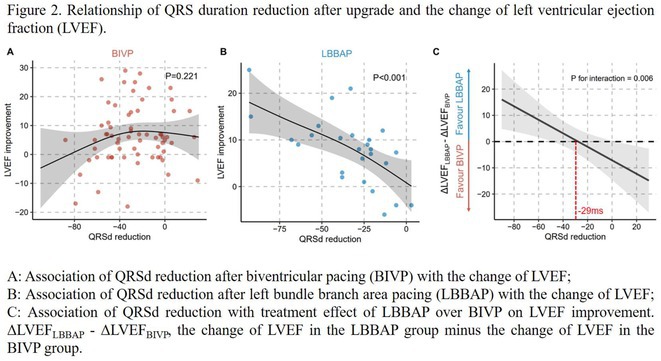

## 40 LONG‐TIME OUTCOMES AND SAFETY OF HIS‐PURKINJE CONDUCTION SYSTEM PACING: A MULTICENTER OBSERVATIONAL STUDY

### 40.1 **WEIJIAN HUANG** ^1^, JIANGANG ZOU^2^, BINNI CAI^3^, LAN SU^1^, WEI HUA^4^

#### 40.1.1 ^1^The first affiliated hospital of Wenzhou medical university, Wenzhou, China,^2^First Affiliated Hospital, Nanjing Medical University, Nanjing, China,^3^Xiamen Cardiovascular Hospital of Xiamen University, Xiamen, China,^4^National Clinical Research Center of Cardiovascular Diseases, Fuwai Hospital, Beijing, China

**Introduction:** This study aimed to evaluate the long‐term outcomes and lead performances of conduction system pacing (CSP) in a prospective, multicenter, collaborative study.

**Methods:** This study prospectively enrolled 2,060 pacemaker patients who received HBP met strict threshold criteria and LBBP from January 2019 to December 2021 in 4 centers. Pacing parameters and echocardiographic data were assessed at implant and during follow‐up. Clinical outcomes such as heart failure hospitalizations (HFH) and all‐cause mortality were assessed.

**Results:** Among 2,060 patients (mean age 69±13 years, 44% female), 448 received HBP and 1612 received LBBAP (1384 LBBP, 228 LVSP). The mean follow‐up time was 39.14±13.29 months (Q1‐Q3: 30‐51months). In HBP groups, the His capture threshold was 0.67±0.50 mV/0.5ms at implant and increased to 0.89±0.62 mV/0.5 ms during last follow‐up, with 6.03% had an increasement of > 1V/0.5ms; while in LBBP group, the capture threshold increased from 0.52±0.33 mV/0.5 ms to 0.73±0.39 mV/0.5, with an lower risk of an threshold increasement of > 1V/0.5ms (2.82%). In those with narrow QRS duration, the LVEF remained stable during follow‐up (62.0±13.35% vs 61.4 ± 9.8%, p=0.12), while improved significantly in patients with LBBB (40.5±16.0% to 56.3±12.3%, P<0.001). HFH and all‐cause mortality were observed in 5.73% and 5.58% of patients, respectively.

**Conclusions:** This large multicenter study demonstrates that HBP met strict threshold criteria and LBBP were both effective and safe during long‐term follow‐up. Furthermore, LBBP exhibited a lower and more stable lead performance.

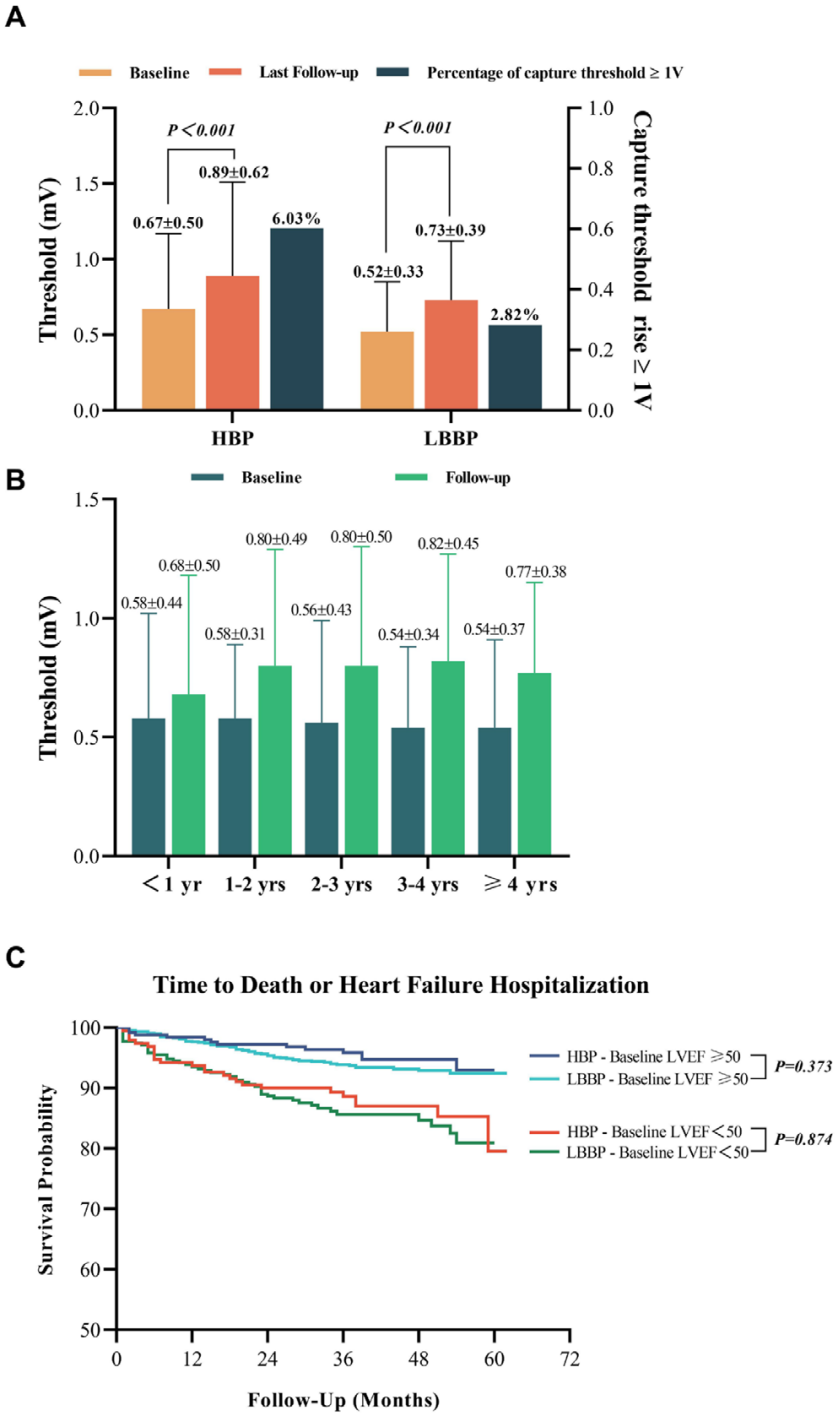

## 41 NOGO B ENHANCES THE INFLAMMATORY MICROENVIRONMENT OF EPICARDIAL ADIPOSE TISSUE AND INDUCES ATRIAL FIBRILLATION BY PROMOTING PREADIPOCYTE INTO WHITE‐LIKE ADIPOCYTE THROUGH THE AKT2‐GSK3β‐CATENIN PATHWAY

### 41.1 QIN HUIYUAN

#### 41.1.1 Nanjing Medical University, Nanjing, China

**Introduction:** Epicardial adipose tissue (EAT) is involved in atrial fibrillation (AF) progression, with increased white adipocytes correlating with atrial fibrosis. However, the precise mechanisms remain unknown.

**Methods:** EAT samples from 26 AF patients and 24 sinus rhythm individuals underwent metabolomics and proteomics analysis. A lentiviral vector overexpressing NogoB was used to generate 3T3‐L1‐NogoB‐OE cells for in vitro studies. Western blotting, qRT‐PCR, and ELISA assessed lipid differentiation and inflammation. RNA‐seq and western blot examined NogoB's role in differentiation pathways. Optical mapping described electrical changes in cardiomyocytes. In vivo, AAV8‐mediated NogoB overexpression in CS‐CREM mice was used to observe AF and fibrosis. SKL2001's efficacy in mitigating atrial arrhythmia was evaluated.

**Results:** In a cohort of 26 individuals with atrial fibrillation (AF) and 24 with sinus rhythm, demographic characteristics like age, gender, hypertension, and diabetes were balanced. AF patients had significantly larger epicardial adipose tissue and adipocytes (*p*=0.004, 0.001). Metabolomic profiling showed distinct metabolic features in AF patients, including reduced anti‐inflammatory lipids (LPC 16:0, LPC 18:1) and increased lipid storage molecule Phosphatidylglyceride 16. Proteomics linked NogoB with these changes, influencing cell differentiation via the AKT2/GSKβ/catenin pathway.Co‐culture experiments involving 3T3‐L1‐NogoB overexpressing (OE) cells with cardiomyocytes and cardiac fibroblasts demonstrated elongated action potentials and enhanced fibroblast activity, evidenced by increased expression of αSMA, collagen I, and TGF‐β. In animal models, NogoB overexpression via AAV8 increased AF incidence and fibrosis, but β‐catenin agonist SKL2001 reduced these effects, suggesting a potential therapeutic approach.

**Conclusions:** NogoB exacerbates EAT inflammation and promotes AF by inducing preadipocyte differentiation into white‐like adipocytes via the AKT2‐GSK3β‐Catenin pathway.

## 42 REDUCTION IN P‐WAVE DURATION AND P/PR RATIO FOLLOWING RF ABLATION FOR ATRIAL FIBRILLATION IS ASSOCIATED WITH FREEDOM FROM REOCCURRENCE

### 42.1 **USMAN HUSSAIN** ^1^, MARIA KLESEWETTER^1^, ANDRE TAYLOR^1^, SAMUEL GEORGE^1^, RISHI ADA^1^, MOEEN ABEDIN^1^, CLAUDIA LUCUS^2^, ZAINUL ABEDIN^1^

#### 42.1.1 ^1^Paul Foster School of Medicine at El Paso, El Paso, TX,^2^University Medical Center of El Paso, El Paso, TX

**Introduction:** Isolating large segments of atrial myocardium reduces the surface area of the electrically active left atrium and therefore reduces the P‐wave duration. Successful pulmonary vein and antrum isolation will result in a reduction in P wave duration as a result of a reduction in conduction time. This in turn should result in a reduction of the reoccurrence of atrial fibrillation during follow‐up. Although the P‐wave conduction time reduction has been demonstrated in previous studies, the reduction in P /PR ratio and follow‐up for one year to ascertain for reoccurrence of atrial fibrillation has not been previously demonstrated.

**Methods:** Sixty‐six patients underwent radiofrequency ablation for atrial fibrillation. Post‐procedure electrocardiogram, performed within 24 hours, was analyzed for P‐wave duration and P/PR ratio (Figure 1).

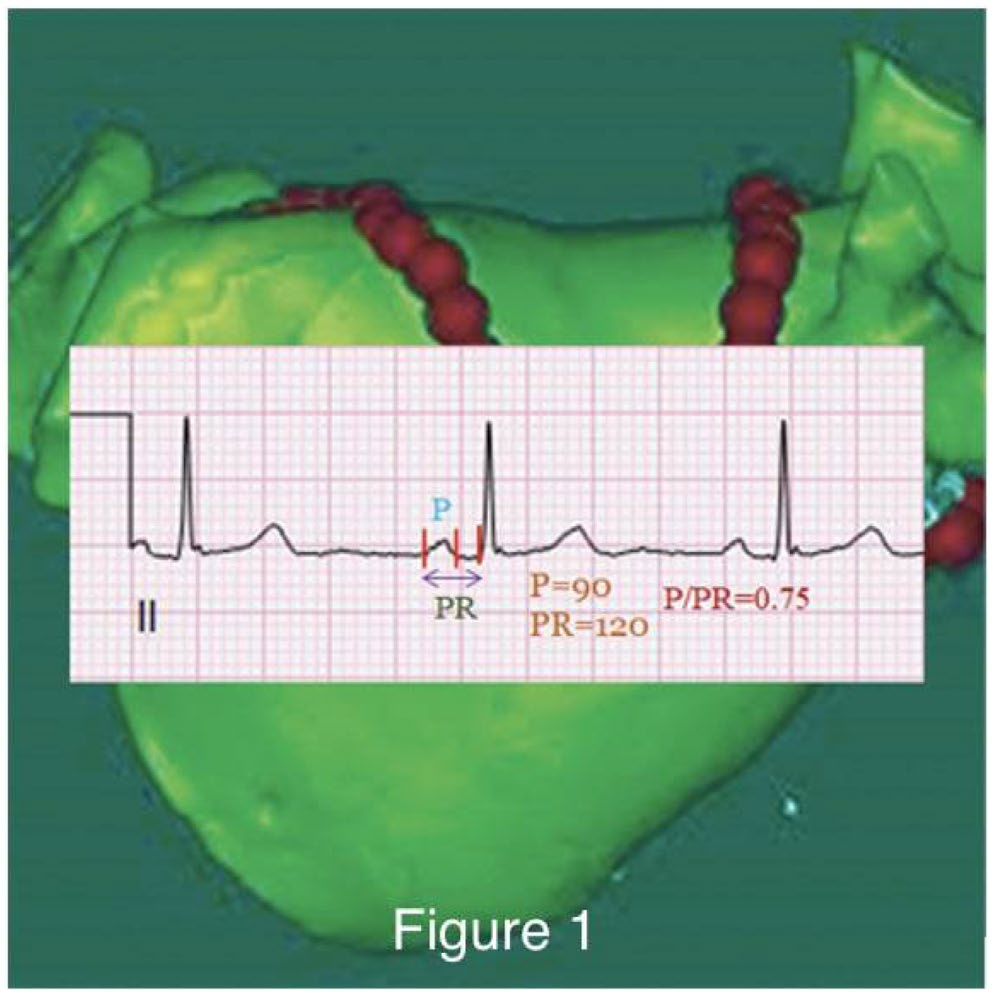

Patients in whom subsequent electrocardiograms at one‐year follow‐up were available were included in the study. Thirty‐two patients had follow‐up electrocardiograms available for analysis with twenty‐three being male.

**Results:** Twenty‐two patients in whom there was a reduction in the P‐wave duration following radiofrequency ablation did not have a recurrence of atrial fibrillation at one year (Sensitivity 95.6%) (Table 1).

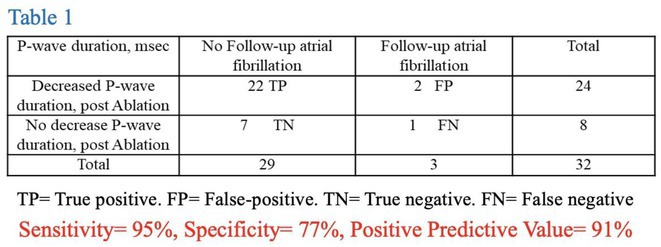

**Conclusions:** Reduction in the P‐wave duration and P/PR ratio in an immediate postoperative electrocardiogram, suggests a long‐term (one year) freedom from reoccurrence of atrial fibrillation following the successful pulmonary vein isolation ablation.

## 43 PREDICTING ATRIAL FIBRILLATION RECURRENCE AFTER CATHETER ABLATION USING SPATIAL VARIABILITY IN LEFT ATRIAL ACTION POTENTIAL DURATION RESTITUTION: FROM A DIGITAL TWIN STUDY

### 43.1 **TAEHYUN HWANG** ^1^, OH‐SEOK KWON^1^, DAEHOON KIM^1^, BYOUNGHYUN LIM^1^, MOON‐HYUN KIM^2^, JE‐WOOK PARK^2^, HEE TAE YU^1^, TAE‐HOON KIM^1^, JAE‐SUN UHM^1^, BOYOUNG JOUNG^1^, MOON‐HYOUNG LEE^1^, HUI‐NAM PAK^1^

#### 43.1.1 ^1^Yonsei University College of Medicine, Seoul, Korea, Republic of,^2^Yongin Severance Hospital, Yongin, Korea, Republic of

**Introduction:** We have used digital twin technology to perform extra pulmonary vein (PV) ablation in patients with non‐paroxysmal atrial fibrillation (AF). We reported that high dominant frequency (DF) site ablation improved rhythm outcomes, but high maximal slope of action potential restitution curve (Smax) site ablation did not. This study aimed to delve into the influence of spatial heterogeneity in Smax on the outcomes of AF ablation.

**Methods:** This retrospective analysis, conducted at a single center, involved 116 patients with persistent AF who underwent atrial fibrillation catheter ablation (AFCA). The left atrium was segmented into 16 regions, and associations among mean Smax, voltage, DF, and WT values in each segment were explored. The coefficient of variation (COV) was calculated for each parameter to assess the relationship between value variability and spatial heterogeneity. Patients were stratified into high‐ and low‐variability groups based on the median values, and post‐AFCA outcomes were compared using Cox regression analysis.

**Results:** Smax demonstrated negative correlations with both mean voltage (r=‐0.48) and mean DF (r=‐0.46). Conversely, COV‐Smax showed positive correlations with mean voltage (r=0.43) and mean DF (r=0.31) and a negative correlation with mean Smax (r=‐0.43). When stratified by the median COV‐Smax value (60%), the group with low COV‐Smax had significantly higher recurrence rates compared to the high COV‐Smax group (log‐rank p=0.014, HR 2.26, 95% CI 1.16‐4.40). Even after adjusting for age, sex, and body mass index (BMI), both COV‐Smax values (HR 0.97, 95% CI 0.95‐0.99) and the low COV‐Smax group (HR 2.60, 95% CI 1.31‐5.19) independently predicted poor outcomes following AFCA.

**Conclusions:** Our findings indicated that lower spatial Smax heterogeneity, measured using digital twin technology, was associated with poorer outcomes post‐AFCA among the patients with non‐paroxysmal AF.

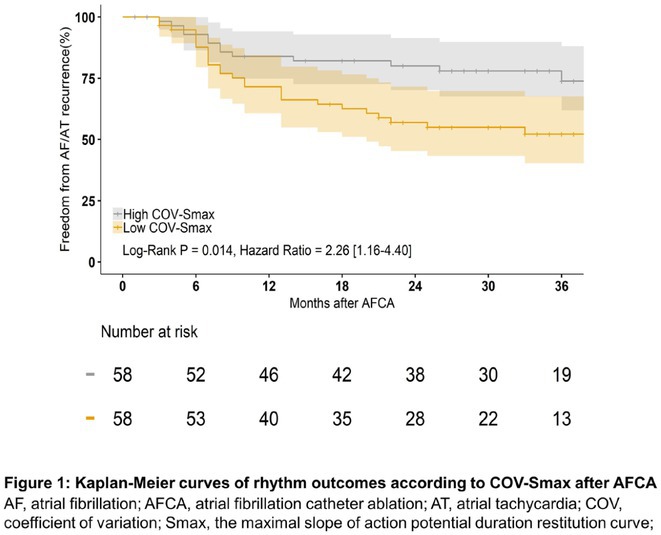

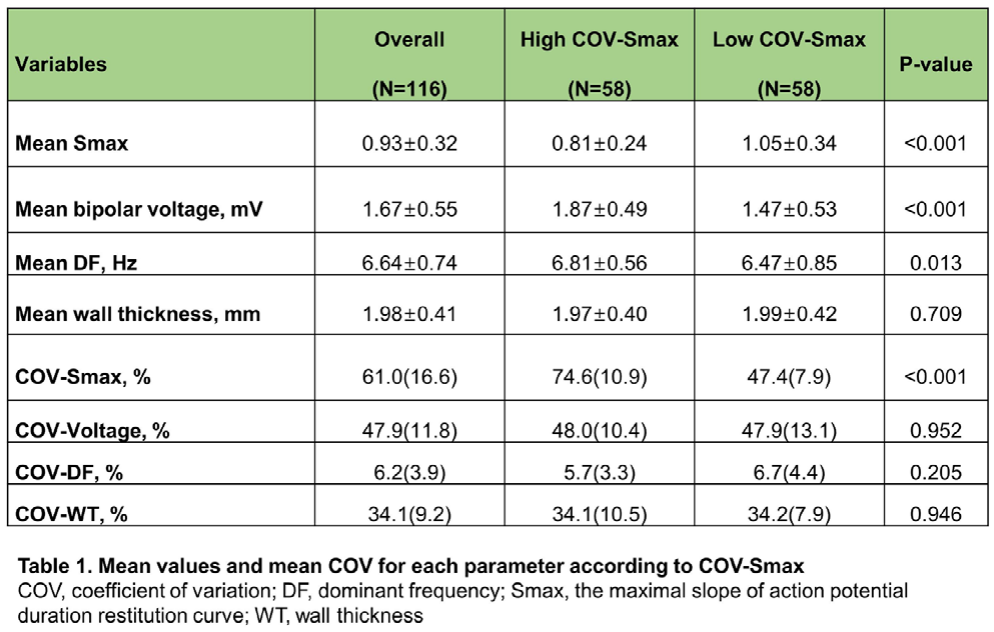

## 44 NON‐INVASIVE ASSESSMENT OF HYDROQUINIDINE EFFECT IN BRUGADA SYNDROME (THE QUIET BRS STUDY)

### 44.1 **JULIA ISBISTER** ^1,2^, MARINA STROCCHI^3,4^, MATTHEW RIEDY^5^, LAURA YEATES^1,6,7,2^, BELINDA GRAY^1,2^, EMMA S SINGER^2,6,8^, RICHARD BAGNALL^2,6,8^, JODIE INGLES^7,1^, HARIHARAN RAJU^9^, CHRISTOPHER SEMSARIAN^1,6,2^, STEVEN NIEDERER^3,4,10^, RAYMOND SY^1,2^

#### 44.1.1 ^1^Royal Prince Alfred Hospital, Sydney, Australia,^2^Faculty of Medicine and Heath, The University of Sydney, Sydney, Australia,^3^National Heart and Lung Institute, Imperial College London, London, United Kingdom,^4^School of Biomedical Engineering and Imaging Sciences, King's College London, London, United Kingdom,^5^Medtronic Australasia, Melbourne, Australia,^6^Agnes Ginges Centre for Molecular Cardiology at Centenary Institute, Sydney, Australia,^7^Genomics and Inherited Disease Program, Garvan Institute of Medical Research and University of New South Wales, Sydney, Australia,^8^Bioinformatics and Molecular Genetics Group at Centenary Institute, Sydney, Australia,^9^Faculty of Medicine, Health and Human Sciences, Macquarie University, Sydney, Australia,^10^The Alan Turing Institute, London, United Kingdom

**Introduction:** Hydroquinidine reduces arrhythmic events in patients with Brugada syndrome (BrS). The mechanism by which it exerts antiarrhythmic benefit and it's electrophysiological effects on BrS substrate remains incompletely understood. The aim of this study was to determine the effect of Hydroquinidine on ventricular depolarisation and repolarisation in patients with BrS in vivo using non‐invasive parameters including electrocardiographic imaging (ECGi).

**Methods:** Twelve patients with BrS underwent electrocardiogram (standard, high‐lead and signal averaged) and ECGi at baseline and “on‐treatment” with hydroquinidine 300mg BD. Activation time (AT), repolarisation time (RT) and activation‐recovery intervals (ARI) were computed for the ventricles and the right ventricular outflow tract (RVOT). Serum Hydroquinidine levels were determined, and adverse drug events captured through medication survey.

**Results:** Hydroquinidine increased RT (301.1 ± 24.1ms vs 348.8 ± 28.3ms, p <0.001), repolarisation gradients (1.1 ± 0.4 ms/mm vs 1.6 ± 0.4 ms/mm, p<0.001) and ARI (241.3 ± 18.1 ms vs 284.8 ± 21.5 ms, p<0.001) in the right RVOT, with a greater change in the RVOT compared to the rest of the ventricles. Mean AT in the RVOT did not change significantly on‐treatment with Hydroquinidine (59.4 ± 13.5ms vs 63.8 ± 19.2ms, p=0.066). Hydroquinidine levels did not correlate with electrophysiological changes or occurrence of adverse drug reactions. One patient developed frequent runs of non‐sustained ventricular tachycardia on‐treatment with Hydroquinidine which resolved following cessation of the drug.

**Conclusions:** Hydroquinidine primarily affects ventricular repolarisation and action potential duration (indicated by ARI) in patients with BrS and demonstrates regional variation with changes greatest in the RVOT.

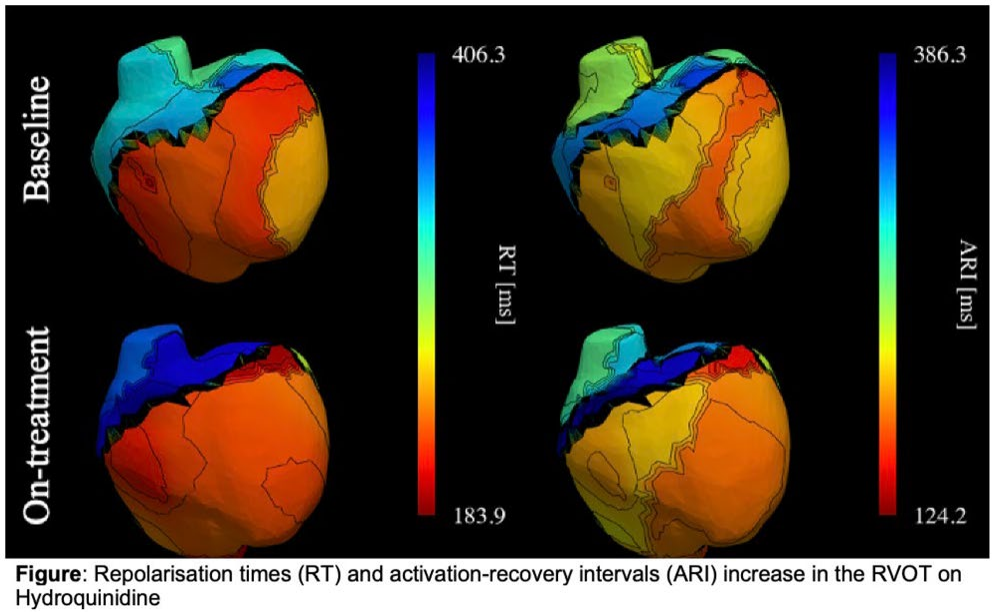

## 45 VERY LONG‐TERM RECURRENCE OF AF AFTER CATHETER ABLATION IS ASSOCIATED WITH DIFFERENTIAL REMODELLING OF THE LA AND LAA: POTENTIAL IMPLICATIONS TO LAA ARRHYTHMOGENESIS AND STROKE RISK

### 45.1 **MOHANARAJ JAYAKUMAR**, ANAND THIYAGARAJAH, SURAYA HANI KAMSANI, SHAUN EVANS, JOHN FITZGERALD, MEHRDAD EMAMI, PRASHANTHAN SANDERS

#### 45.1.1 Royal Adelaide Hospital, Adelaide, Australia

**Introduction:** Patient and arrhythmia characteristics are important determinants of AF recurrence after catheter ablation of atrial fibrillation (AF). Left atrial (LA) remodelling during AF determines the outcome of AF ablation, with a reduction in LA size post ablation being associated with improved outcomes. The extent of LA appendage (LAA) remodelling and its role in recurrence of AF following catheter ablation has not well documented.

**Methods:** This was a retrospective observational study of cases undertaken at 3 associated hospitals. We compared pre‐ and post‐ablation LA and LAA volumes in patients who had very late recurrence after initially successful ablation. The LAA and LA volumes were obtained from pre‐procedural CT scans of the patients who underwent a repeat ablation. A three‐dimensional image of LA and LAA were constructed using Ensite™ Mapping System. The consequential LA and LAA volumes were measured using the reconstructed 3D maps. We included only those who had good quality CT scans with LA and LAA contrast filling on both occasions.

**Results:** From a total of 59 consecutive patients underwent repeat catheter ablation very late after initially successful AF ablation, we included 20 patients (60% male) with the mean age of 70.8±10.5 years who had good quality CT scans with adequately filled LAA in both CT scans. The mean time difference between the first and second ablations were 11.7±2.9 years. The mean left atrial volume decreased from 100.4±25.5 cm^3^ before the index ablation to 94.7±26.8 cm^3^ before the repeat ablation (P Value=0.51). The left atrial appendage size has increased significantly from 8.5±3.8 cm^3^ at the index ablation to 12.5±6.7 cm^3^ before the repeat ablation (P Value=0.0004).

**Conclusions:** There is evidence of differential remodelling of the LA and LAA chronically after catheter ablation of AF. This remodelling is associated with significant LAA volume expansion. These features may potentially contribute to LAA arrhythmogenesis and also thrombogenesis and warrants further evaluation.

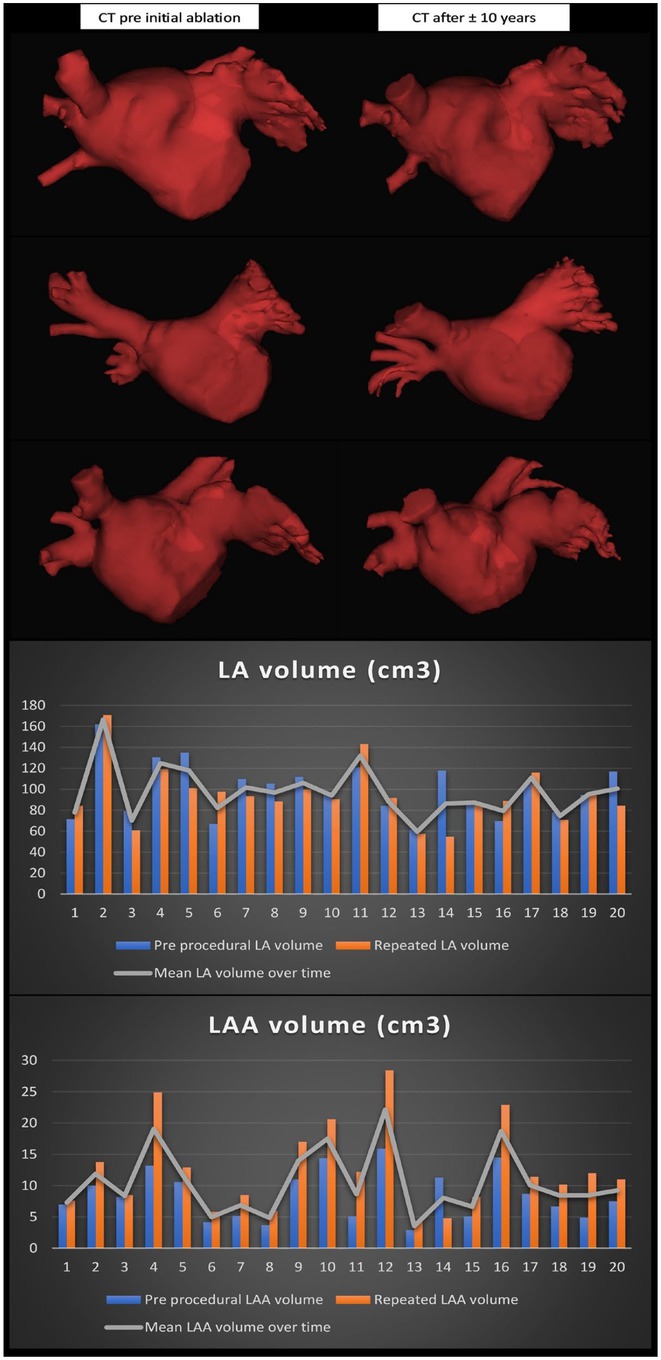

## 46 STEREOTACTIC RADIOTHERAPY ABLATION FOR PULMONARY VEIN ISOLATION: AN ANIMAL STUDY

### 46.1 **ZIHAN JIANG**, AIKAI ZHANG, LEI DING, LIJIE MI, YUANDONG LIU, MIN TANG

#### 46.1.1 Arrhythmia Center, State Key Laboratory of Cardiovascular Disease, Fuwai Hospital, National Center for Cardiovascular Diseases, Chinese Academy of Medical Sciences and Peking Union Medical College, Beijing, China

**Introduction:** The use of stereotactic radiotherapy ablation (SBRT) for refractory ventricular tachycardia is gaining popularity due to its non‐invasiveness and speed. Can SBRT be used for pulmonary vein isolation? Safety and effectiveness of current practices are limited. Animal experiments were designed to assess SBRT effects on pulmonary veins and nearby organs.

**Methods:** Adult canines were used as the experimental subjects. The right main pulmonary vein was set as the clinical target volume (CTV). The planning target volume (PTV) was defined by adding 0‐3 mm to the CTV, excluding the overlap area between the esophagus and main bronchus. The total prescribed dose of the PTV was 20 Gy. SBRT was performed using TrueBeam^TM^. After 3 months, electroanatomic mapping of the left atrium was performed. The canines were then sacrificed and the left atrium, pulmonary veins, surrounding esophagus, phrenic nerve, and lungs were examined microscopically. All tissues were stained with HE, eosin, and Masson's trichrome stain.

**Results:** One canine has completed the study by now. The canine was maintained in good health during breeding and showed no clinical symptoms. Electroanatomical mapping was performed three months after SBRT, and neither electrical conduction block nor voltage abnormalities were found in the left atrium and pulmonary veins. Pathology of the right upper pulmonary vein showed a transmural injury in the subendocardium. The pathology of the right lower pulmonary vein was normal, but inflammatory cell infiltration and fibrous hyperplasia were observed in the adjacent lung tissues. Pathology of the left upper pulmonary vein showed subepicardial hemorrhage without myocardial or endocardial damage. Pathology of the left lower pulmonary vein showed endocardial and subendocardial myocardial damage (Figure). The structures of the esophagus and the phrenic nerve were intact, and no pathological damage was observed.

**Conclusions:** SBRT at 20 Gy may not be sufficient to cause transmural damage to pulmonary vein tissue and cannot effectively implement electrical isolation. At the same time, this dose is safe for the esophagus and the phrenic nerve.

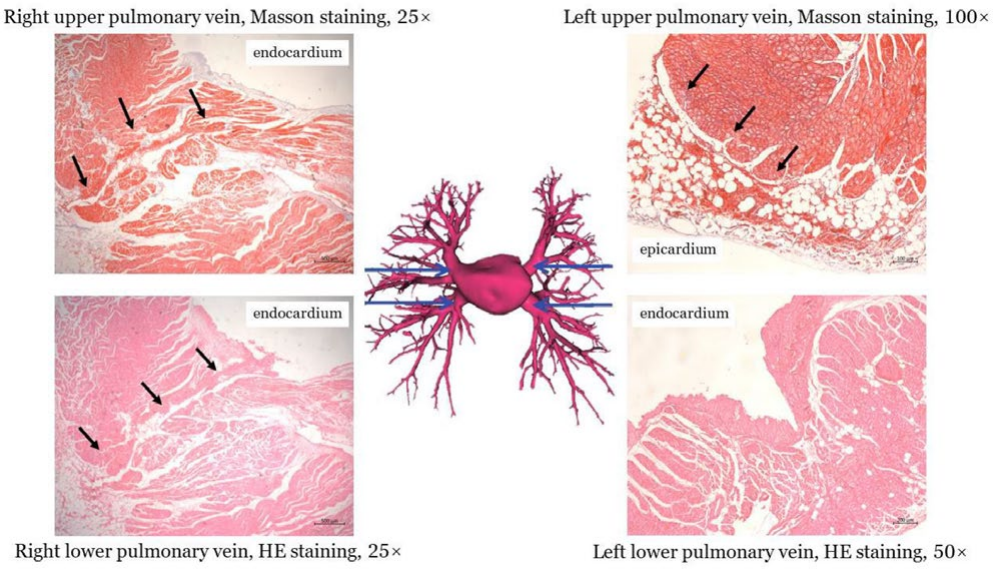

Chair

**B. Joung**

Yonsei University Severance Hospital, Korea, Republic of

## 47 DEVELOPMENT AND VALIDATION OF A MULTIVARIABLE PREDICTION MODEL TO ESTIMATE THE PROBABILITY OF MODERATE‐TO‐SEVERE SLEEP‐DISORDERED BREATHING IN PATIENTS WITH ATRIAL FIBRILLATION: MOODS‐AF

### 47.1 **KADHIM KADHIM** ^1^, ADRIAN ELLIOTT^2^, MELISSA E MIDDELDORP^2^, CHRISHAN NALLIAH^3^, R DOUG MCEVOY^4^, NICHOLAS ANTIC^4^, RAJEEV PATHAK^5^, MEHRDAD EMAMI^2^, DENNIS LAU^2^, JONATHAN KALMAN^3^, DOMINIK LINZ^6^, PRASH SANDERS^2^

#### 47.1.1 ^1^Freeman Hospital, Newcastle upon Tyne, United Kingdom,^2^Centre for Heart Rhythm Disorders, Adelaide, Australia,^3^Royal Melbourne Hospital, Melbourne, Australia,^4^Adelaide Institute for Sleep Health (AISH), Adelaide, Australia,^5^Canberra Heart Rhythm, Canberra, Australia,^6^Maastricht University Medical Centre and Cardiovascular Research Institute, Maastricht, Netherlands

**Introduction:** Sleep‐disordered breathing (SDB) is common in atrial fibrillation (AF) patients and negatively impact prognosis. Tools for AF patient selection for SDB testing are lacking. We aimed to develop and validate a prediction tool to detect AF patients with moderate‐to‐severe SDB.

**Methods:** Prospectively‐collected data on 442 consecutive ambulatory AF patients undergoing polysomnography were used as the derivation sample, and performance was externally validated on a test cohort of 409 patients. Significant SDB was defined as an apnoea‐hypopnoea‐index ≥15/hr. Multivariable logistic regression was used to construct a prediction model and calculate individual SDB probabilities.

**Results:** Significant SDB was present in 34% and 54% of patients in the derivation and validation cohorts respectively. The prediction model comprised age, sex, body mass index (BMI), diabetes and previous stroke or transient ischaemic attack (TIA). Following calibration, the model had a good discrimination ability for significant SDB on external validation (C‐statistic [95% confidence interval] = 0.75 [0.71‐0.80]). A simplified composite score (MOODS, range 0‐8) comprised Male sex (1 point), Overweight (BMI 25‐29.9, 1 point) or Obesity (BMI≥30, 3 points), Diabetes (2 points) and Stroke/TIA (2 points) had good discrimination on external validation (C‐statistic = 0.73 [0.68‐0.77]). As a rule‐out or a rule‐in test, a MOODS score of ≤1 had a 100% sensitivity and score of ≥5 had a 96% specificity for detecting significant SDB, respectively.

**Conclusions:** The MOODS score provides an individualised and accurate probability of significant SDB in AF patients. MOODS has the potential to aid clinical decision making and allow efficient resource allocation.

## 48 DIFFERENT EFFECT OF DEFIBRILLATOR SHOCKS ON THE MYOCARDIAL DAMAGE BETWEEN SUBCUTANEOUS AND TRANSVENOUS IMPLANTABLE CARDIOVERTER‐DEFIBRILLATORS: ASSOCIATION BETWEEN TOTAL SHOCK JOULES AND MYOCARDIAL DAMAGE

### 48.1 **TAKAHIDE KADOSAKA** ^1^, MASAYA WATANABE^2^, SHOTA SAITO^1^, KOTARO NISHINO^1^, JIRO KOYA^1^, DAISHIRO TATSUTA^1^, MOTOKI NAKAO^1^, TARO TEMMA^1^, TOSHIHISA ANZAI^1^

#### 48.1.1 ^1^Hokkaido University, Sapporo, Japan,^2^Caress Sapporo Hokko Memorial Hosipital, Sapporo, Japan

**Introduction:** We previously reported that an accumulation of total shock energy was associated with a higher mortality in patients with a transvenous implantable cardioverter‐defibrillator (TV‐ICD). An experimental swine study reported that TV‐ICD shock was associated with elevated cardiac biomarkers whereas the subcutaneous ICD (S‐ICD) shock caused less myocardial damage than TV‐ICD. However, the difference in the influence of shock therapies between via TV‐ICD and S‐ICD has not been adequately examined in patients. We aimed to investigate the influence of shocks on myocardium in patients with TV‐ICD and S‐ICD.

**Methods:** We enrolled consecutive patients who received a defibrillator or transvenous pacemaker (TV‐PM) implantation between November 2020 and November 2023. A total of 72 patients were included and divided into three groups: patients with TV‐ICD with defibrillation testing (DFT) (TV‐DFT, 27 patients), those with TV‐ICD or TV‐PM without DFT (TV‐no DFT, 23 patients) and those with S‐ICD (22 patients). All patients with S‐ICD received a DFT. Serum troponin‐I level was measured as a surrogate of myocardial damage.

**Results:** Serum troponin‐I levels were significantly elevated in 4‐8 hours after implantation compared to baseline in TV‐DFT and TV‐no DFT but not in S‐ICD group. Delta troponin‐I (the difference between baseline and 4‐8 hours after implantation) was significantly increased in TV‐DFT group compared with TV‐no DFT (181.6 ± 198.6 pg/ml vs 72.6 ± 57.0 pg/ml, *p* = 0.009) and S‐ICD groups (vs ‐ 2.6 ± 10.9 pg/ml, *p* < 0.001). In patients with TV‐ICD (44 patients), delta troponin‐I was significantly correlated with total shock joules (r = 0.571, p < 0.001). In the multivariable linear regression analysis, a value of total shock joule was independently associated with delta troponin‐I (*p* < 0.001).

**Conclusions:** The elevation of serum troponin‐I was associated with the TV‐ICD shock, dependent on the total shock joule, but not with S‐ICD shock. Our results suggest that the S‐ICD shock is less harmful to the myocardium than TV‐ICD shock.

## 49 VOLUMETRIC LESION ANALYSIS AND VALIDATION OF VARIOUS BIPOLAR CONFIGURATIONS IN RADIO FREQUENCY ABLATION OF VENTRICULAR MYOCARDIUM IN A BOVINE MODEL

### 49.1 SAIKIRAN KAKARLA

#### 49.1.1 Sree Chitra TIrunal Institute for Medical Sciences and Technology, Thiruvananthapuram, India

**Introduction:** The bipolar radiofrequency ablation(B‐RFA) strategy was increasingly used to target deep intramural re‐entrant foci responsible for the arrhythmia not ablated by conventional unipolar RFA / sequential unipolar RFA. Lesional characteristics of various bipolar configurations were largely unknown.

**Objective:** To investigate the lesional geometry in relation to various factors to determine the most effective ablation strategy that minimises steam pops and achieves transmurality. To assess the tissue temperatures at the indifferent electrode.

**Methods:** A custom‐made validated ex‐vivo bipolar ablation model was used to assess lesion formation. The myocardial sample was placed between two ablation catheters in four different orientations. Lesions were created using different power (30 W, 40 W, 50 W) and time settings(30, 40 and 50 seconds) with different catheter orientations. Data was analysed using binary logistic regression and multiple linear regression.

**Results:** Among 107 lesions, The volume of the active catheter lesion (266 +/‐ 137 mm^3) significantly differed from the passive counterparts (130 +/‐ 91.8 mm^3) (p < 0.001), and the temperatures at the passive end were lower than at the active electrode (p = 0.004). Higher power and longer duration application led to more frequent steam pops (p < 0.001), while true parallel configuration resulted in fewer steam pops (p < 0.001).

**Conclusions:** A custom model without ground electrode temperature monitoring is cost‐effective. The safest strategy is a true parallel configuration with an inter‐electrode distance of at least 15mm and a power of 30W to 40W, which generates lower steam pops and better transmurality.

## 50 ARE STYLET‐DRIVEN LEADS A BETTER OPTION THAN LUMENLESS PACING LEADS? A META‐ANALYSIS COMPARING SAFETY AND EFFICACY IN PATIENTS RECEIVING LEFT BUNDLE BRANCH PACING

### 50.1 **WILLIAM KAMARULLAH**, GIKY KARWIKY, RAYMOND PRANATA, CHAERUL ACHMAD, MOHAMMAD IQBAL

#### 50.1.1 Department of Cardiology and Vascular Medicine, Faculty of Medicine, Padjadjaran University, Hasan Sadikin General Hospital, Bandung, Indonesia

**Introduction:** Left bundle branch pacing (LBBP) has gained significant momentum recently due to its shorter learning curve and more stable pacing parameters than His‐bundle pacing (HBP). While early experience with lumenless lead (LLL) for LBBP is extensively utilized, data on efficacy and safety of stylet‐driven lead (SDL) against LLL are constrained. We aimed to compare the performance of SDL during LBBP in comparison to LLL.

**Methods:** Systematic literature search was conducted using PubMed, Europe PMC, and ScienceDirect for studies that compared the outcomes of SDL during LBBP compared to LLL. Study outcomes included periprocedural parameters, pacing metrics, and complications.

**Results:** A total of 6 studies involving 3991 participants were included. LBBP procedural success was comparable between groups. Compared to LLL, SDL appeared to result in shortened procedure (MD: ‐11.50 (‐22.35, ‐0.65); P=0.04; I^2^=77%) and fluoroscopy (MD: ‐2.56 (‐4.84, ‐0.28); P=0.03; I^2^=89%) time, along with increased capture threshold (MD: 0.13 (0.05, 0.20); P<0.001; I^2^=70%) and reduced lead impedance (MD: ‐84.44 (‐126.32, ‐42.56); P<0.001; I^2^=78%) at implantation. Nonetheless, electrocardiogram (paced QRS) and pacemaker parameters (R‐wave amplitude, capture threshold, lead impedance) remained comparable between both groups at follow‐up. Lead dislodgement and lead‐related complications (except septal perforation) occurred mostly in SDL group. No statistical differences were found in life‐threatening complications after pacing.

**Conclusions:** Current evidence suggests that SDL is a viable alternative to LLL, with comparable pacing metrics and implant success rates. A specific learning curve should be considered for SDL, yet additional long‐term, randomized trials are still warranted to corroborate these findings.

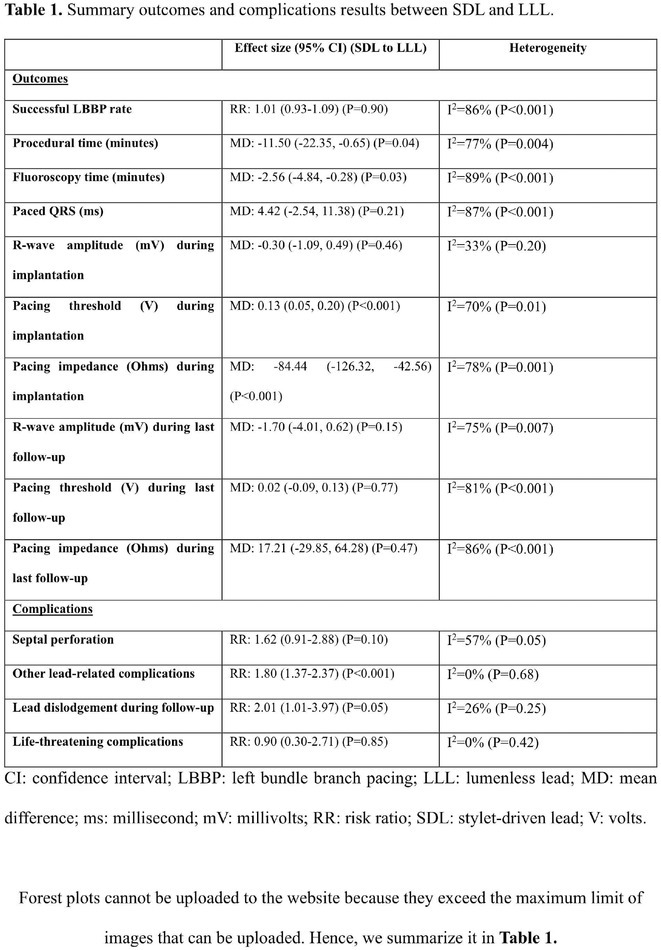

## 51 EXOSOMAL LONG NON‐CODING RNA H19 AS A BIOMARKER AND THERAPEUTIC TARGET FOR ATRIAL FIBRILLATION

### 51.1 **JI‐YOUNG KANG**, DASOM MUN, MALGEUM PARK, GYEONGSEO YOO, BOYOUNG JOUNG

#### 51.1.1 Yonsei University College of Medicine, Seoul, Korea, Republic of

**Introduction:** Exosomes are nanosized membranous vesicles that can mediate intercellular communication by transmitting various forms of RNAs. In recent years, exosomal long non‐coding RNAs (lncRNAs) have been reported to exhibit different expression profiles and play important roles in the development and progression of many diseases. However, there are still few reports on their role in atrial fibrillation (AF). Therefore, the aim of this study was to explore AF‐related exosomal lncRNAs, thereby providing an ideal theragnostic target for AF.

**Methods:** Serum exosomes from controls and patients with persistent AF were isolated and characterized by TEM, NTA, and western blot. In addition, AF‐related pathophysiological roles of exosomal lncRNAs were investigated in angiotensin II (Ang II)‐treated human induced pluripotent stem cell‐derived atrial cardiomyocytes (iPSC‐atrial CMs) and mice.

**Results:** After RNA‐sequencing analysis, we identified 27 differentially expressed lncRNAs (i.e., 4 upregulated and 23 downregulated lncRNAs with a |fold change| ≥ 2 and *p* < 0.05) in serum exosomes from patients with persistent AF compared with the controls. Among them, qRT‐PCR reveled that lncRNA H19 was consistently downregulated in serum exosomes and clinical tissues of patients with persistent AF. Interestingly, the levels of exosomal lncRNA H19 in serum exhibited significant diagnostic validity for AF. In addition, lncRNA H19 was involved in the pathophysiological mechanisms of AF. In Ang II‐treated iPSC‐atrial CMs, overexpression of lncRNA H19 significantly attenuated Ang II‐induced increases in levels of hypertrophic markers (ANP, BNP, and β‐MHC), cell surface area, and inflammation (*p* < 0.05). Similarly, a series of *in vivo* experiments showed that cardiac‐specific overexpression of lncRNA H19 improved abnormal cardiac structure and function. Mechanistically, we found that lncRNA H19 regulated the expression of PTEN, which promotes the atrial remodeling and consequent AF, by sponging miR‐141‐3p and miR‐200a‐3p.

**Conclusions:** This study suggests that exosomal lncRNA H19 could serve as a new potential biomarker and therapeutic target for AF.

## 52 LEFT BUNDLE BRANCH AREA PACING IN PATIENTS WITH HISTORY OF MYOCARDIAL INFARCTION AND PACING INDICATION

### 52.1 **VIKAS KATARIA**, MOHIT BHAGWATI, PRITAM KITEY, AMITABH YADUVANSHI

#### 52.1.1 Holy Family Hospital, New Delhi, India

**Introduction:** Left bundle branch area pacing (LBBAP) has emerged as a popular technique for conduction system pacing. Feasibility and success rates to achieve selective LB pacing in patients with past history of myocardial infarction (MI) is not known.

**Methods:** We studied the procedural success of LBBAP for patients with documented past history of MI and pacing indication. All patients undergoing LBBAP between 1^st^ January 2023 to 31^st^ December 2023 fulfilling inclusion criteria were enrolled. Procedural success was defined as a) Selective LBB caputure: short and consistent Stim‐LVAT at high and low pacing output and an isoelectric interval between the pacing artifact and the ventricular electrogram b) Acceptable pacing threshold (<1.5V @ 0.5ms) and sensing parameters (R wave >5mV).

**Results:** Of all the patients undergoing LBBAP, twenty two fulfilled the inclusion criteria. Baseline and procedural characteristics of these patients are given in the table below.

Previous treatment records and/or angiograms of all the patients were reviewed. All of them had past history of MI; 13 patients had inferior wall MI, 2 patients had lateral wall MI and 7 patients had anterior wall MI. Of those 7 patients who had anterior wall MI, 6 patients had proximal inter‐ventricular septal (IVS) scar as confirmed by ECG (qRBBB) and Echocardiogram. Cardiac MRI data was available for four of these patients which showed transmural scar involving >80% of the septum. Selective LB capture could not be achieved in 6 patients who had proximal septal scar, whereas it could be successfully achieved in all the other 16 patients without the septal scar. Pacing threshold was much higher and R wave sensing was much lower in patients with the proximal septal scar than those without it Threshold 1.95±0.26 V @0.4ms Vs 0.78±0.16 V @0.4ms; R wave: 5.9±0.58 mV Vs 16.1±3.16)

**Conclusions:** LBBAP is feasible and successful (as defined above) in post‐MI patients except those who have proximal ventricular septal scar. Pre‐procedural knowledge about the presence of septal scar by ECG/ECHO/CMRI may be helpful in selecting the patients suitable for LBBAP.

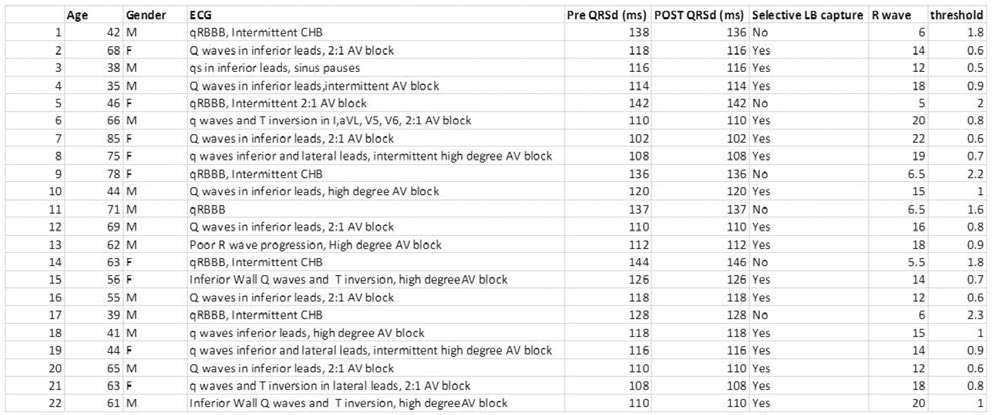

Chair

**E. Kizana**

Sydney, Australia

## 53 OPERATOR LEARNING CURVE WITH A NOVEL DUAL‐ENERGY LATTICE‐TIP ABLATION SYSTEM

### 53.1 **ERICH KIEHL** ^1^, STAVROS MOUNTANTONAKIS^2^, MOUSSA MANSOUR^3^, DEVI NAIR^4^, DINESH SHARMA^5^, TYLER TAIGEN^6^, PETR NEUZIL^7^, JOSEF KAUTZNER^8^, JOSE OSORIO^9^, ANDREA NATALE^10^, JOHN HUMMEL^11^, ANISH AMIN^12^, USMAN SIDDIQUI^13^, DORON HARLEV^14^, PAUL HULTZ^14^, SOFI ROSEN^14^, BIRCE ONAL^14^, KHALDOUN TARAKJI^14^, VIVEK REDDY^15^, ELAD ANTER^16^

#### 53.1.1 ^1^Sentara, Newport News, VA,^2^Northwell, New Hyde Park, NY,^3^Massachusetts General Hospital, Boston, MA,^4^St. Bernards Medical Center & Arrhythmia Research Group, Jonesboro, AR,^5^NCH Rooney Heart Institute, Naples, FL,^6^Cleveland Clinic, Cleveland, OH,^7^Na Homolce Hospital, Prague, Czech Republic,^8^IKEM Prague, Prague, Czech Republic,^9^HCA Florida Miami, Miami, FL,^10^Texas Cardiac Arrhythmia Institute, Austin, TX,^11^Ohio State Univ Div of Cardio, Columbus, OH,^12^Riverside Methodist Hospital, Upper Arlington, OH,^13^Florida Cardiology, Orlando, FL,^14^Medtronic, Minneapolis, MN,^15^Helmsley Electrophysiology Center, Mount Sinai Fuster Heart Hospital, New York, NY,^16^Shamir Medical Center, Be'er Ya'Akov, Israel

**Introduction:** The SPHERE Per‐AF trial randomized patients with persistent atrial fibrillation (AF) to ablation with a wide‐footprint, lattice‐tip, pulsed field (PF) and radiofrequency (RF) mapping and ablation catheter vs a conventional RF catheter. The investigational arm met its primary non‐inferiority efficacy and safety endpoints with efficacy of 73.8% in the investigational vs 65.8% in the control arm. While operators were highly experienced with the control, the majority were new to the investigational system and therefore each site treated 0‐2 roll‐in patients. The objective of this analysis was to assess the learning curve using this novel system and its impact on efficacy and efficiency outcomes.

**Methods:** Patients were grouped based on the sequential cases performed by an operator during the trial. Roll‐in patients were included, but patients treated by operators who performed ≤2 procedures using Sphere‐9 were excluded. The composite effectiveness endpoint was freedom from acute procedural failure, repeat ablation at any time, or arrhythmia recurrence, drug initiation/escalation, or cardioversion each at 1 year excluding a 3‐month blanking period. Efficiency endpoints included energy application time, time between first and last lesion, and skin‐to‐skin procedural time.

**Results:** The total cohort included 443 patients (235 investigational, 208 control). In the investigational arm, a decrease in skin‐to‐skin time was noted from operators’ first to second patients (119 vs 101 min) and time between first and last lesion (56 min vs 48 min) with minimal change in energy application time (6.6 min vs 6.1 min). Efficiency outcomes remained similar thereafter with increasing operator experience. The primary effectiveness endpoint improved with increased operator volume. For the first five patients treated by operators, primary effectiveness was 65% (74/114), for patients 6‐10: 75% (33/44), and for patients >10: 80% (60/75).

**Conclusions:** Efficacy of a novel dual‐energy, lattice‐tip ablation system was more pronounced compared to conventional RF after a short operator learning curve, with persistent procedural efficiency observed after 2 cases performed.

## 54 ELECTROCARDIOGRAPHIC AGING AND SEX MISCLASSFICATION DERIVED FROM ARTIFICIAL INTELLIGENCE IS ASSOCIATED WITH THE RISK OF ATRIAL FIBRILLATION

### 54.1 **DAEHOON KIM**, OH‐SEOK KWON, HANJIN PARK, JE‐WOOK PARK, HEE TAE YU, TAE‐HOON KIM, JAE‐SUN UHM, BOYOUNG JOUNG, MOON‐HYOUNG LEE, HUI‐NAM PAK

#### 54.1.1 Yonsei University College of Medicine, Seoul, Korea, Republic of

**Introduction:** The application of artificial intelligence (AI) algorithms to 12‐lead electrocardiogram (ECG) provides promising age and sex prediction methods. We investigated whether the gap between the AI‐predicted age from ECG (AI‐ECG age) and chronological age or the misclassification of sex can predict an incidence of atrial fibrillation (AF).

**Methods:** We validated a pre‐trained residual network (ResNet)‐based model for age prediction on four independent multinational datasets (total ECG number=414,804); CODE‐15% (*r*=0.83), PTB‐XL (*r*=0.74), UK Biobank (*r*=0.53), and SaMi‐Trop (*r*=0.60). We developed an AI‐ECG gender prediction model using the MIMIC4 cohort and validated it on two independent and multinational datasets sourced from UK (AUC 0.92) and Brazil (AUC 0.89). After the validation, the AI ECG age and AI ECG‐classified sex was estimated in the Severance Hospital dataset (n=558,149). We categorized the AI‐ECG age gap into two groups: aged ECG (≥10 years) and normal ECG age (<10 years) groups based on the mean absolute ECG age gap error and ECG‐gender discrepancy was defined as having ECG‐gender probability of ≥50% for the opposite sex.

**Results:** During a mean follow‐up of 5.5 (standard deviation [SD] 5.2) years, 9,417 (1.7%) had incident AF (mean age 50.7 [SD 15.7] years; 55.3% females). Compared with normal ECG age, aged‐ECG was associated with a higher risk of AF incidence (hazard ratio [HR] 1.80, 95% confidence interval [CI] 1.73‐1.88). ECG‐gender discrepancy was associated with a HR of 1.46 (95% CI 1.38‐1.54) for incident AF, compared with correctly classified ECG‐gender. The association of ECG‐gender discrepancy with an increased risk of AF was consistently observed among both men (HR 1.32, 95% CI 1.19‐1.45) and women (HR 1.48, 95% CI 1.38‐1.59). When incorporating AI prediction of age and gender, individuals with aged ECG and ECG‐gender discrepancy had a 2.49‐fold increased risk of AF (95% CI 2.31‐2.68), compared with those with normal ECG age and correctly classified gender.

**Conclusions:** AI‐derived ECG‐aging and ECG‐gender discrepancy was associated with a higher risk of AF incidence.

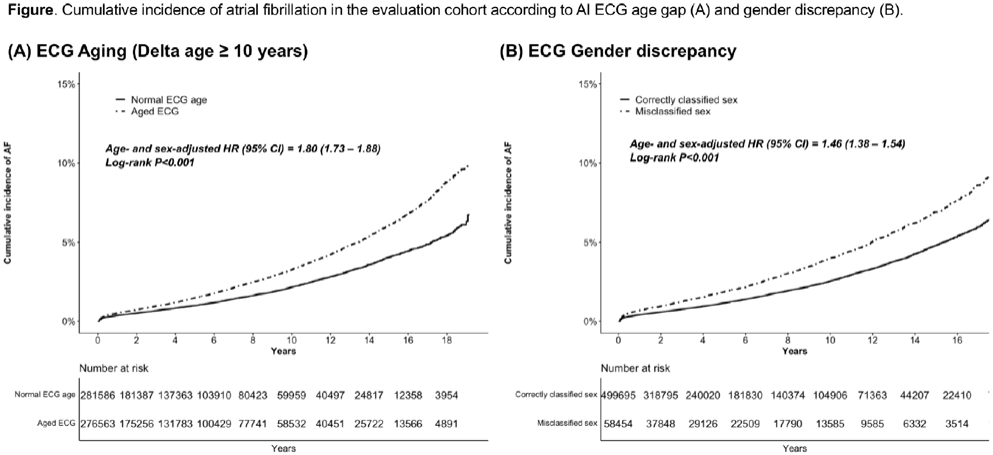

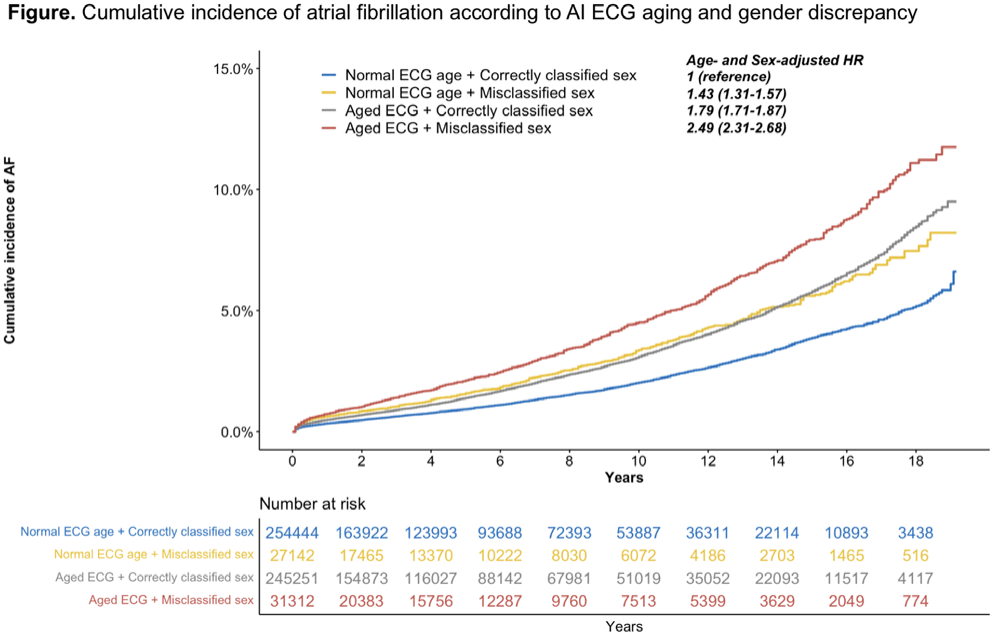

## 55 USING COMPUTED TOMOGRAM ATRIAL MYOCARDIAL THICKNESS MAPS IN CRYOBALLOON PULMONARY VEIN ISOLATION: THE UTMOST‐AF2 RANDOMIZED CLINICAL TRIAL

### 55.1 **DAEHOON KIM**, OH SEOK KWON, TAEHYUN HWANG, HEE TAE YU, TAE‐HOON KIM, JAE‐SUN UHM, BOYOUNG JOUNG, MOON‐HYOUNG LEE, HUI‐NAM PAK

#### 55.1.1 Yonsei University College of Medicine, Seoul, Korea, Republic of

**Introduction:** Durable pulmonary vein (PV) isolation is a crucial determinant of successful atrial fibrillation (AF) ablation. It is uncertain whether adjusting the duration of ablation based on atrial wall thickness provides extra benefits. We studied the safety and effectiveness of tailored cryoballoon PV isolation (Cryo‐PVI) based on left atrial (LA) wall thickness (LAWT) for patients with paroxysmal AF.

**Methods:** 279 patients with paroxysmal AF refractory to anti‐arrhythmic drug (AAD) therapy were randomized 1:1 to either LAWT‐guided Cryo‐PVI (LAWT, n=135) and conventional Cryo‐PVI (control, n=142). Conventional Cryo‐PVI was performed using a 28‐mm cryoballoon with a recommended application time of 240 s per ablation. Cryoballoon application time in the LAWT group was titrated (additional application for 120 s at PVs, where more than 25% of the circumference includes segments with a LAWT exceeding 2.5 mm and reduced baseline application for 180 s at PVs where more than 75% of the circumference includes segments with a LAWT of less than 1.5 mm) according to the computed tomogram‐myocardial thickness color map. The primary endpoint was freedom from any documented atrial arrhythmia of more than 30 seconds without antiarrhythmic medication, after a single ablation procedure.

**Results:** Median age was 62 years (32.3% women). During a median follow‐up of 18 (IQR 14‐23) months, 101 patients (74.8%) of the LAWT group were free from recurrent atrial arrhythmia without antiarrhythmic medication, compared with 91 (64.1%) of the control group (HR 0.64, 95% CI 0.42‐0.99; log‐rank P=0.043). No differences were observed between the LAWT and control groups in major complication rate (3.0% vs. 1.4%, respectively; p=0.647). The total procedure time was extended in the LAWT group than in the control group (median 68 vs. 61 minutes, p=0.001).

**Conclusions:** Among patients with paroxysmal AF receiving Cryo‐PVI, the LAWT‐guided energy titration strategy improved freedom from atrial arrhythmia without antiarrhythmic medication, compared with conventional strategy.

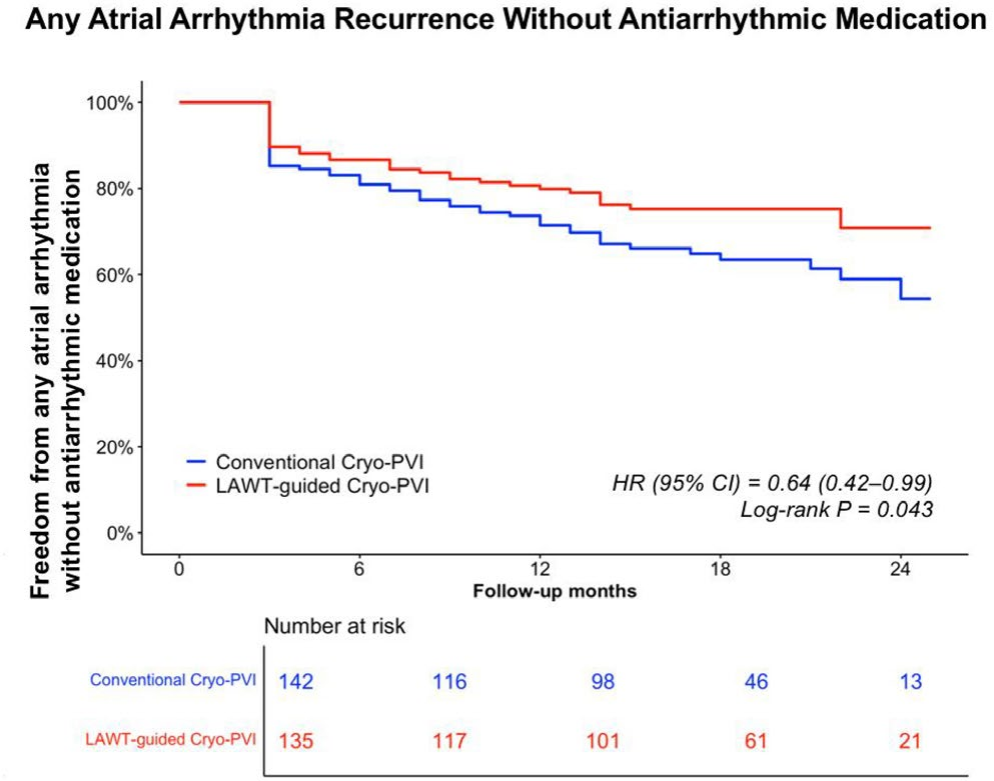

## 56 IMPACT OF ATRIAL FUNCTIONAL SUBSTRATE IN PATIENTS WITH ATRIAL FIBRILLATION

### 56.1 **YASUHITO KOTAKE** ^1^, SHUNSUKE KAWATANI^1^, TAKUYA TOMOMORI^1^, AIKO TAKAMI^2^, MASARU KATO^1^, KAZUHIRO YAMAMOTO^1^

#### 56.1.1 ^1^Tottori university, Yonago, Japan,^2^Tottori Prefectural Central Hospital, Yonago, Japan

**Introduction:** Decremental evoked potential (DEEP) mapping is one of the functional substrate mapping methods mainly used in the field of ventricular arrhythmia. We hypothesize that atrial functional substrate in patients with atrial fibrillation (AF) reflects one aspect of atrial electrophysiological remodeling and potential ablation targeting.

**Methods:** Consecutive patients referred for AF ablation were prospectively included. DEEP mapping was performed in left atrial roof and posterior wall from two different pacing locations (endocardially from the left atrial appendage, epicardially from the proximal coronary sinus). The electrograms (EGMs) during S1 600 ms drive and after an extra‐stimulus (S2 at +10 ms above atrial refractoriness) were studied at each location and assessed for decremental properties.

**Results:** Sixty‐four patients were included and 45% had persistent AF. Extra‐stimulus EGMs were individually analysed and measured. Fifty‐five percent of patients showed DEEP properties at left atrial roof and/or posterior wall. DEEPs were more prevalent in patients with long duration of AF (persistent AF 57% vs paroxysmal AF 43%, p=0.04). Similarly, DEEPs were more prevalent in patients with longer time from initial diagnosis to ablation procedure (161 [130‐364] days vs 134 [101‐206] days, p=0.03). The patients with DEEP properties have more early recurrence of AF after ablation procedure than those without DEEP properties (p=0.001).

**Conclusions:** This study showed AF patients with atrial DEEP properties is characterized by a longer duration of AF. These patients tended to have more early recurrence of AF after ablation procedure. Atrial DEEP might reflect atrial electrophysiological remodeling and be correlated with recurrence rate after ablation.

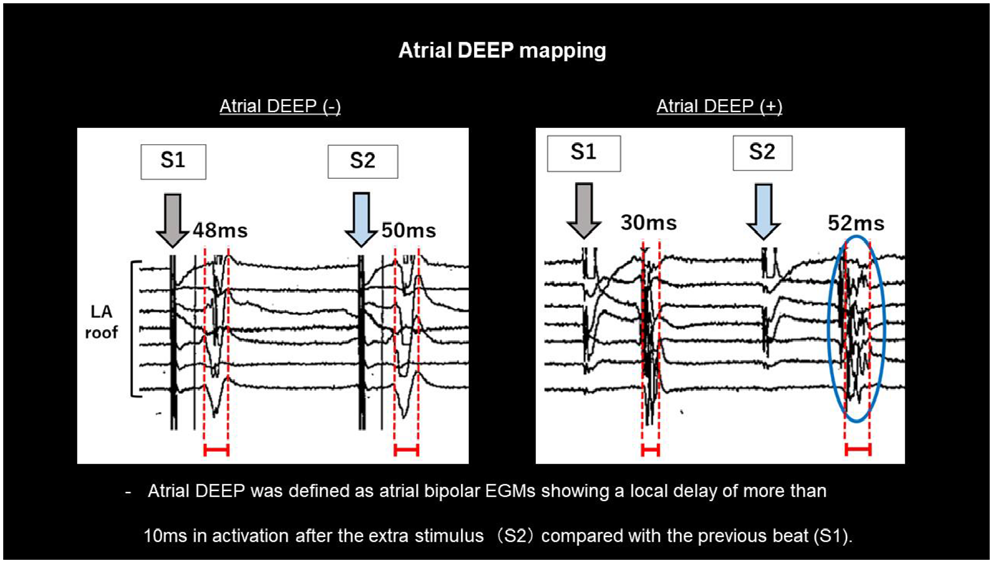

Chair

**C. Leclercq**

University Hospital of Rennes, France

## 57 CRYOBALLOON OR RADIOFREQUENCY ABLATION IN PATIENTS WITH PERSISTENT ATRIAL FIBRILLATION: FIRST RESULTS FROM THE FIRE AND ICE II PILOT STUDY

### 57.1 **KARL HEINZ KUCK** ^1^, STEPHAN WILLEMS^2^, KYOUNG‐RYUL JULIAN CHUN^3^, THOMAS ARENTZ^4^, DANIEL BECKER^5^, ROLAND TILZ^1^, SERGE BOVEDA^6^

#### 57.1.1 ^1^Universitätsklinikum Schleswig‐Holstein, Lübeck, Germany,^2^Asklepios Klinik St. Georg Hamburg, Hamburg, Germany,^3^Cardioangiologisches Centrum Bethanien, Frankfurt, Germany,^4^University Heart Center Freiburg, Bad Krozingen, Germany,^5^Medtronic GmbH, Meerbusch, Germany,^6^Clinique Pasteur, Toulouse, France

**Introduction:** Pulmonary vein isolation (PVI) is the cornerstone of catheter ablation for atrial fibrillation (AF), and time‐to‐AF recurrence is a widely accepted endpoint. However, AF burden (AFB) may be a more relevant clinical parameter. This study evaluated AFB before and after treatment with PVI only using cryoballoon‐ (Cryo) or radiofrequency‐ (RF) ablation in persistent AF.

**Methods:** Patients were randomized 1:1 to Cryo or RF. Included were patients with symptomatic persistent AF (≤12 months) and antiarrhythmic medication (AAD) failure at 5 sites. An implantable loop recorder was inserted ≥1 month before ablation to measure AFB (hours/day). Patients were followed ≥1 year. The primary efficacy endpoint was clinical treatment failure (TF) (a composite of any documented atrial arrhythmia ≥30 sec, AF hospitalization, cardioversion, repeat ablation, use of AAD) after a 90‐day blanking period. The primary safety endpoint was device‐ or procedure‐related serious adverse events. Further endpoints were AFB, scar area assessed via MRI and detections of AF sources by non‐invasive body surface electrocardiographic imaging before and after ablation.

**Results:** In total, 61 patients were randomized (29 to Cryo). Enrollment was on median 67 (27‐146) days after persistent AF diagnosis with 65±11 years of age, 74% male, with a LVEF of 58±9%. AFB in the Cryo arm was reduced from a median of 23.5 (16.9‐23.7) to 0.2 (0.0‐3.2) hours/day and in the RF arm from 23.6 (20.9‐23.7) to 0.5 (0.1‐4.1); comparison using a mixed regression model revealed no difference (p=0.62). Primary analysis comparing TF shows no difference between arms (HR: 1.10 [0.64‐1.91]; p=0.73). Cumulative incidence estimates at 12 months for TF were 79% for Cryo and 81% for RF. In total, two primary safety endpoints were reported, a dyspnea after initial RF ablation and a death 32 days after RF repeat ablation.

**Conclusions:** PVI only ablation in persistent AF shows similar safety and efficacy outcomes for Cryo and RF ablation. Evaluation of AF burden reduction reveals treatment benefits (in both arms) that were hidden by standard time‐to‐treatment failure analysis.

## 58 EXTENDED PERIOD OUTCOMES OF POSTERIOR BOX ISOLATION IN FOUR RANDOMIZED ATRIAL FIBRILLATION CATHETER ABLATION TRIALS

### 58.1 **SANG JUN LEE**, HEE TAE YU, SUNG HWA CHOI, DAEHOON KIM, TAEHOON KIM, JAE‐SUN UHM, BOYOUNG JOUNG, MOON‐HYOUNG LEE, HUI‐NAM PAK

#### 58.1.1 Yonsei University, College of Medicine, Seoul, Korea, Republic of

**Introduction:** Catheter‐based electrical posterior box isolation (POBI) and circumferential pulmonary vein isolation (CPVI) do not improve the rhythmic outcomes of atrial fibrillation catheter ablation (AFCA) in previous studies with 12‐24 months of follow‐up. We analyzed the long‐term rhythm outcomes of our four previously‐conducted randomized controlled trials (RCTs) comparing CPVI alone versus CPVI plus additional POBI using the intention‐to‐treat principle.

**Methods:** We analyzed 575 AF patients included in our four previous RCTs. We compared clinical recurrence defined as recurrent atrial arrhythmia after the index procedure. In patients underwent a repeat procedure due to recurrence after the index procedure, the mechanism of recurrence was analyzed.

**Results:** After a median follow‐up of 48 months, there were no significant differences in the clinical recurrence or major adverse cardiac events between the CPVI alone and CPVI plus POBI groups. The procedure time was significantly longer, and the atrial tachycardia (AT) recurrence rate was higher in the CPVI plus POBI group. In the patients who experienced clinical recurrence, there were no significant differences in the rates of cardioversion or need for repeat procedures between the groups. In patients underwent a repeat procedure due to recurrence after the index procedure (N=64), the PV reconnection rate did not differ, but reentrant AT was more common in the CPVI plus POBI group, while extra‐PV triggers were more common in the CPVI alone group.

**Conclusions:** The addition of POBI to CPVI did not improve the long‐term rhythm outcomes in patients undergoing AFCA.

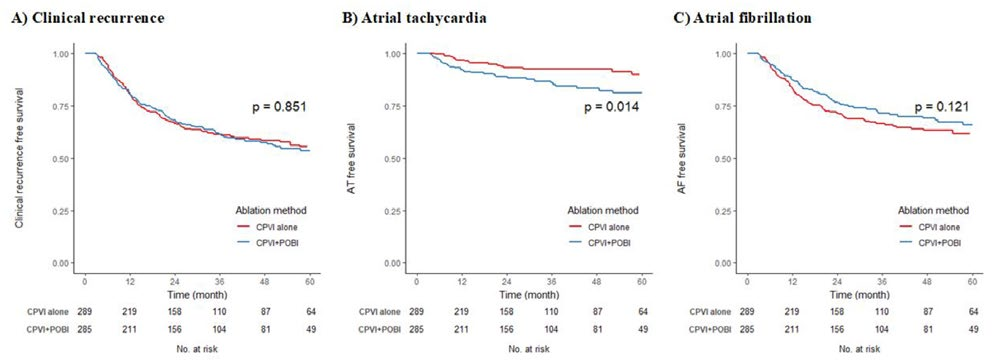

## 59 POLYGENIC RISK SCORE FOR PREDICTION OF ATRIAL FIBRILLATION ACCORDING TO PRESENCE OF THE HYPERTROPHIC CARDIOMYOPATHY

### 59.1 **YOUNG SHIN LEE** ^1,2^, PIL‐SUNG YANG^3^, EUNSUN JANG^1^, DAEHOON KIM^1^, HEE TAE YU^1^, TAE‐HOON KIM^1^, JAE‐SUN UHM^1^, JUNG‐HOON SUNG^3^, HUI‐NAM PAK^1^, MOON‐HYOUNG LEE^1^, JIN‐BAE KIM^2^, BOYOUNG JOUNG^1^

#### 59.1.1 ^1^Severance Cardiovascular Hospital, Yonsei University College of Medicine, Seoul, Korea, Republic of,^2^Kyung Hee University Hospital, Seoul, Korea, Republic of,^3^CHA Bundang Medical Center, CHA University, Seongnam, Korea, Republic of

**Introduction:** The significance of genetic predisposition in predicting atrial fibrillation (AF) is well recognized, yet its role in hypertrophic cardiomyopathy (HCM) patients remains less clear. In this study, we aimed to elucidate the impact of genetic risk on the development of AF in both HCM and non‐HCM participants.

**Methods:** This retrospective analysis examined 1,180 HCM participants (mean age 61.1±7.1, 63.0% male) and 476,238 non‐HCM participants (mean age 57.0±8.1, 45.3% male) from the UK Biobank. Participants were stratified based on their validated polygenic risk score (PRS) for AF: the bottom 10% constituted the low‐risk genetic group, the top 10% comprised the high‐risk genetic group, and the remainder fell into the intermediate‐risk genetic group. We assessed the incidence of AF and major adverse cardiovascular events (MACE) and analyzed predictors, including genetic risk.

**Results:** During the follow‐up period of 11.6 years, while 34.2% individuals developed AF in HCM group, 5.4% did in non‐HCM group. The age‐ and sex‐adjusted AF incidence rates were 2.4, 3.6, and 5.4 per 100 person‐years for the low‐, intermediate‐, and high‐risk genetic group with HCM, and 0.2, 0.5, and 1.0 for the low, intermediate, and high‐risk genetic group in non‐HCM participants, respectively. Genetic risk emerged as a significant predictor of AF in both HCM (HR, 2.51; 95% CI, 1.59‐3.98) and non‐HCM participants (HR, 4.77; 95% CI, 4.47‐5.09) (*P*=0.008 for interaction). In addition to genetic risk, age, diabetes mellitus, and ischemic stroke were identified as significant predictors of AF in participants with HCM. Moreover, high genetic risk was significantly associated with the risk of MACE.

**Conclusions:** In conclusion, our study indicates associations between genetic predisposition and AF occurrence in both HCM and non‐HCM participants.

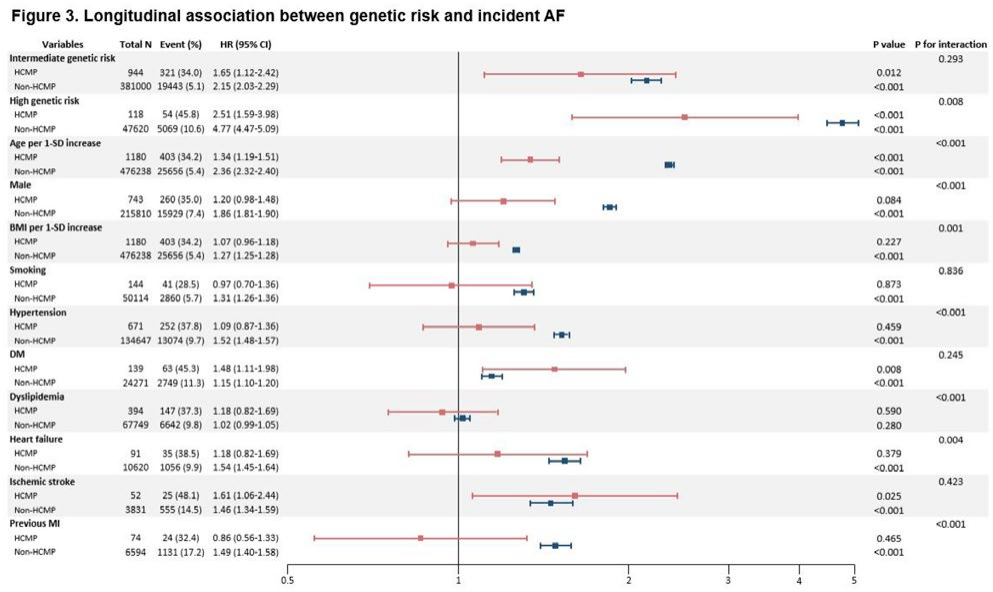

ENHANCING HEALTHCARE ECONOMICS: THE COST‐EFFECTIVENESS OF GENETIC TESTING FOR CONGENITAL LONG QT SYNDROME IN THE THAI POPULATION

**SARIN LEKCHUENSAKUL** ^1,2^, ANKAVIPAR SAPRUNGRUANG^1,2^, SUPALUCK KANJANAUTHAI^3^, APICHAI KHONGPHATTHANAYOTHIN^1,2,4^;

^1^Center of Excellence in Arrhythmia Research Chulalongkorn University, Department of Medicine and Division of Pediatric Cardiology, Department of Pediatrics, Chulalongkorn University, Bangkok, Thailand,^2^Cardiac Center, King Chulalongkorn Memorial Hospital, Bangkok, Thailand,^3^Division of Pediatric Cardiology, Department of Pediatrics, Siriraj Hospital, Bangkok, Thailand,^4^Bangkok General Hospital, Bangkok, Thailand.

**Introduction:** While genetic testing allows for more accurate identification and treatment of the patient and affected family members with congenital long QT syndrome (LQTS), it is associated with increased initial costs. This study explores the cost‐effectiveness of widespread genetic testing for patients diagnosed with LQTS in Thailand.

**Methods:** The Markov Decision Model was used to compare death, quality of life, and cost between 3 treatment strategies of LQTS families once the proband is clinically diagnosed: watchful waiting without treatment, empiric beta‐blocker therapy, and genetic testing (whole exome sequencing for proband and Sanger sequencing of the familial variant for first‐degree relatives to prioritize usage of beta blocker). We assumed family members were identified at 10 years old and are followed for 60 years. Adjustments of assumption were made with available data for Thai population. The risk of sudden death was estimated to be reduced by 50% with beta blockers and 90% with ICD. We assumed quality‐adjusted life‐year were 0.96 for beta blockers, 0.94 for ICD, and 1.0 for no treatment. The cost‐effectiveness of each strategy was calculated as cost per QALY adjusted to current value in Thai Baht.

**Results:** Although the empiric beta‐blocker strategy yielded the lowest LQTS‐related mortality among others, it led to the lowest quality of life. The genetic testing strategy yielded a higher life expectancy than the watchful waiting strategy, with an incremental cost estimation of 30,000 Thai Baht per family member compared to the watchful waiting strategy. In this model, the cost‐effectiveness of genetic testing was estimated to be approximately 60,000 Baht per life‐year saved and 140,000 Thai Baht per QALY, which proved to be economically efficient according to Thailand's National List of Essential Medicines Committee in 2013 (less than 160,000 Thai Baht per QALY for each medical technology is considered cost‐effective).

**Conclusions:** Offering genetic testing for LQTS in a proband, followed by a cascade screening in first‐degree relatives upon a positive result, is likely cost‐effective for the Thai population.

## 60 COMPARISON OF CARDIONEUROABLATION VERSUS PACEMAKER THERAPY IN PATIENTS WITH FUNCTIONAL BRADYCARDIA

### 60.1 **LAI KUAN LEONG**, KENG TAT KOH

#### 60.1.1 Sarawak Heart Centre, Kota Samarahan, Malaysia

**Introduction:** The epicardial autonomic ganglia of the heart regulates our cardiac physiological function. An increase in parasympathetic overactivity may negatively affect the myocardium and cause vagal bradycardia. Patients with symptomatic functional bradycardia are at risk of injury and experience poor quality of life if left untreated. Pacemaker therapy and cardioneuroablation are effective treatments for functional bradycardia. There is no study comparing efficacy and safety of cardioneuroablation versus pacemaker therapy. Therefore, we conducted this study to examine the efficacy and safety of cardioneuroablation and pacemaker therapy in patients with functional bradycardia.

**Methods:** Patients included had functional bradycardia and underwent cardioneuroablation or pacemaker implantation. Primary composite endpoint of the study includes time to first syncope recurrence, complications related to pacemaker therapy and complications related to cardioneuroablation therapy.

**Results:** We included 12 patients (pacemaker [n5], cardioneuroablation [n7]). The mean number of syncopal events pre‐cardioneuroablation was 3.2 (±1.6) whereby 85.7% patients sustained a traumatic fall. The mean number of syncopal events pre‐pacemaker implantation was 2.4 (±1.7) whereby 80% patients sustained a traumatic fall. During follow‐up, one post‐ablation patient still had symptomatic sinus bradycardia which required DCPPM+ CSP implantation. One pacemaker implantation patient still had syncope one year later and received cardioneuroablation.

**Conclusions:** Cardioneuroablation and pacemaker therapy appear to be a safe and effective treatment option for patients with functional bradycardia.

## 61 CARDIOVASCULAR MORTALITY IN PATIENTS WITH ATRIAL FIBRILLATION AND PHYSICAL ACTIVITY INTENSITY: DELINEATING THE THRESHOLD OF VIGOROUS ACTIVITY

### 61.1 **LO CHIEH LING** ^1^, YENN‐JIANG LIN^1^, YUN‐YU CHEN^1^, YU‐CHENG HSIEH^2^, KUO‐LIONG CHIEN^3^, CHING‐HENG LIN^2^, LI‐WEI LO^1^, SHIH‐LIN CHANG^1^, YU‐FENG HU^1^, FA‐PO CHUNG^1^, TA‐CHUAN TUAN^1^, TZE‐FAN CHAO^1^, JO‐NAN LIAO^1^, TING‐YUNG CHANG^1^, CHIN‐YUN LIN^1^, LING KUO^1^, CHIH‐MIN LIU^1^, SHIN‐HUEI LIU^1^, CHENG‐I WU^1^, YU‐SHAN HUANG^1^, SHIH‐ANN CHEN^1,2^

#### 61.1.1 ^1^Taipei Veterans General Hospital, Taipei City, Taiwan,^2^Taichung Veterans General Hospital, Taichung City, Taiwan,^3^National Taiwan University, Taipei City, Taiwan

**Introduction:** There is a correlation between light‐to‐moderate physical activity and reduced atrial fibrillation (AF) incidence and cardiovascular risk. Vigorous activities reduce the benefits and increase the risk of AF. The exact threshold at which physical activity increases the risk of cardiovascular death (CVD) in patients with AF remains to be determined.

**Methods:** This study analyzed data from 23856 individuals from the Taiwan Biobank (2017). Physical activity levels were quantified in the metabolic equivalent task (MET) hours/week and categorized into four groups based on 20 MET‐hour increments. The study employed Kaplan‐Meier survival analysis and an adjusted generalized additive model (GAM) to assess the impact of physical activity on CVD risk (adjusting for age, sex, diabetes, hypertension, chronic obstructive pulmonary disease, and chronic kidney disease), with follow‐up data from 2017 to 2022.

**Results:** Over a median 5.51‐year follow‐up, 238 AF cases (cumulative rate of AF: 0.998%) were identified (48.7% female, mean age 61.7 years). The linear model indicated a lower CVD risk in participants engaging in moderate physical activity (20‐40 MET‐hours/week; adjusted hazard ratio [HR]: 0.38, 95% CI: 0.16‐0.95) compared to the reference group (0‐20 MET‐hours/week). The GAM model demonstrated a significant increase in CVD risk for activities exceeding 40 MET‐hours/week after multivariable adjustment.

**Conclusions:** Physical activity levels and CVD risk in AF patients are correlated in a J‐shaped manner. The CVD risk of AF decreased with mild to moderate physical activity. For AF patients, it establishes a critical threshold of 40 MET‐hours/week, beyond which there is an increased risk of CVD, thus clarifying safe physical activity limits.

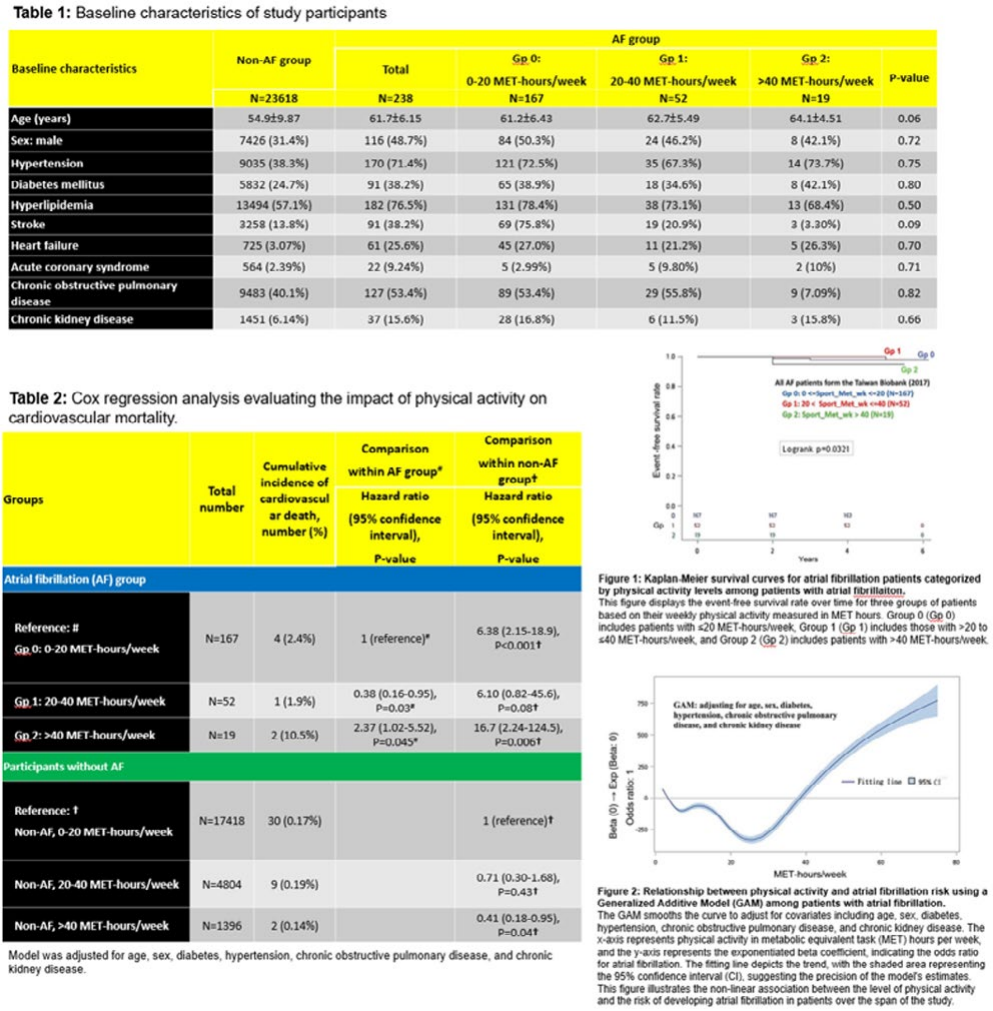

Chair

**T. Liu**

Tianjin Institute of Cardiology, Second Hospital of Tianjin Medical University, China

## 62 COMPARISON OF LEFT BUNDLE BRANCH PACING AND BIVENTRICULAR PACING IN THE TREATMENT OF CHRONIC SYSTOLIC HEART FAILURE WITH LEFT BUNDLE BRANCH BLOCK: A MULTICENTER, PROSPECTIVE, RANDOMIZED, CONTROLLED TRIAL

### 62.1 XUEYING CHEN^1^, **XI LIU** ^1^, WEIWEI ZHANG^2^, YIXIU LIANG^1^, LEI ZHANG^1^, WEI WANG^1^, JIN BAI^1^, JINGFENG WANG^1^, SHENGMEI QIN^1^, RUOGU LI^2^, YANGANG SU^1^

#### 62.1.1 ^1^Department of Cardiology, Zhongshan Hospital of Fudan University, Shanghai Institute of Cardiovascular Diseases, National Clinical Research Center for Interventional Medicine, Shanghai, China,^2^Department of Cardiology, Shanghai Chest Hospital, School of Medicine, Shanghai Jiao Tong University, Shanghai, China

**Introduction:** Left bundle branch pacing (LBBP) has emerged as a potential alternative to biventricular pacing (BVP) in heart failure (HF) patients with left bundle branch block (LBBB). This study is the largest randomized study comparing LBBP and BVP.

**Methods:** This is a multicenter, prospective, randomized, controlled trial. Patients with left ventricular ejection fraction (LVEF) ≤ 35% and LBBB from three centers were randomized 1:1 to LBBP or BVP group. Here, we present the preliminary results comparing LBBP and BVP in electrocardiographic parameters, echocardiographic measurements and N‐terminal pro‐B‐type natriuretic peptide (NT‐proBNP) during the initial 6‐month follow‐up.

**Results:** Two hundred patients were randomized from October 2020 to March 2022. The success rate was 98% in the LBBP group and 94% in the BVP group (P = 0.28). Crossovers occurred in 2% of LBBP and 6% of BVP. All patients completed 6‐month follow‐up. Intention‐to‐treat analysis showed the paced QRS duration in the LBBP group was significantly shorter than those at baseline (120.6 ± 18.1 ms vs. 169.8 ± 19.0 ms, P < 0.01) and in the BVP group (137.4 ± 15.8 ms, P < 0.01). Echocardiographic measurements were comparable at baseline, while LBBP achieved greater LVEF (45.0 ± 9.6 % vs. 39.2 ± 7.4 %, P < 0.01), LVESD (44.1 ± 10.6 mm vs. 50.1 ± 9.9 mm, P < 0.01) and LVEDD (57.2 ± 8.6 mm vs. 61.5 ± 8.0 mm, P < 0.01) than BVP at 6‐month follow‐up. There was no significant difference in response rate (improvement in LVEF ≥ 5%) between LBBP and BVP groups (86.0 % vs. 81.0 %, P = 0.34) while super‐response rate (improvement in LVEF ≥ 15% or LVEF ≥ 50%) was significantly higher in the LBBP group compared to the BVP group (55.0 % vs. 36.0 %, P < 0.01) at 6‐month follow‐up. LBBP also showed greater NT‐proBNP than BVP (1184.9 ± 1482.0 pg/mL vs. 1501.2 ± 1339.4 pg/mL, P < 0.01) at 6‐month follow‐up.

**Conclusions:** LBBP demonstrated greater electrical, mechanical and hematological improvements than BVP in HF patients with LBBB, and is potentially an alternative to BVP. Date of clinical endpoints with long follow‐up period is forthcoming.

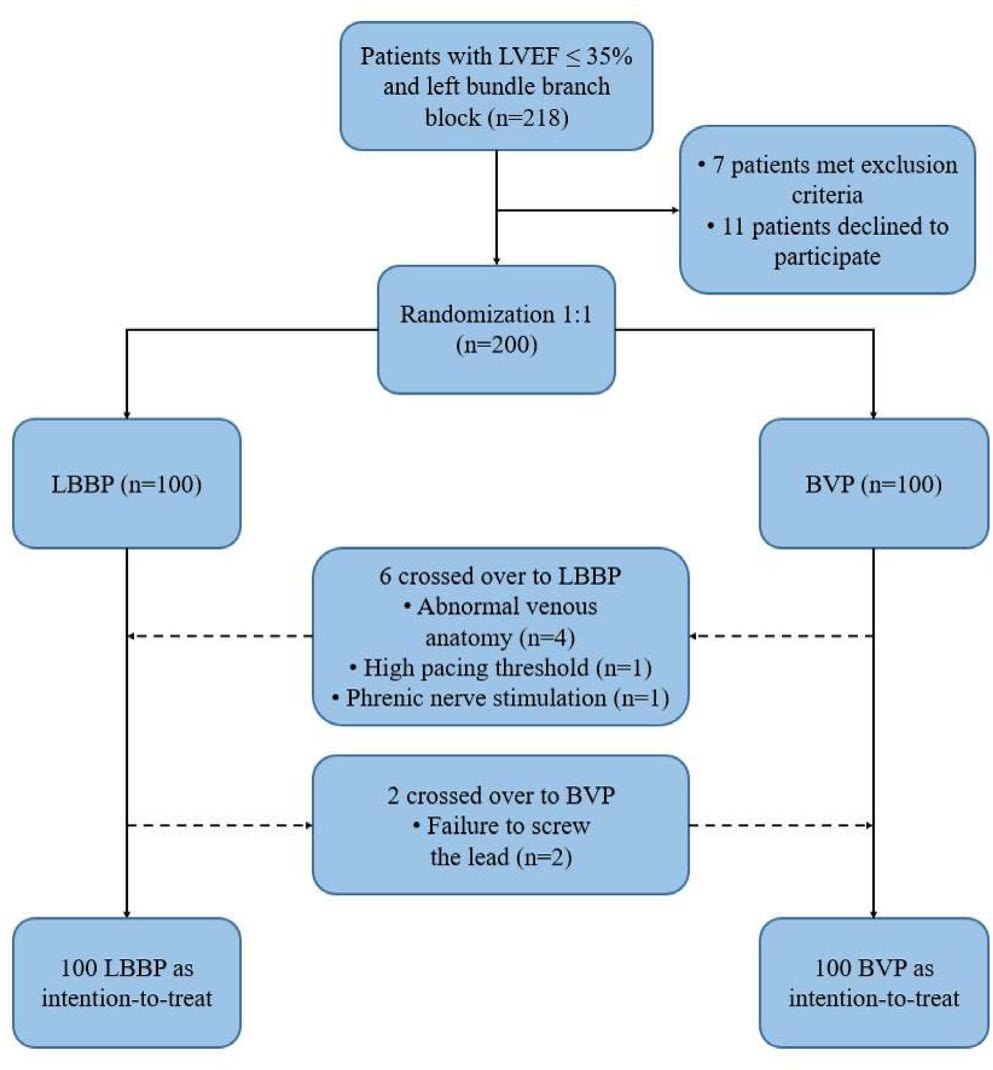

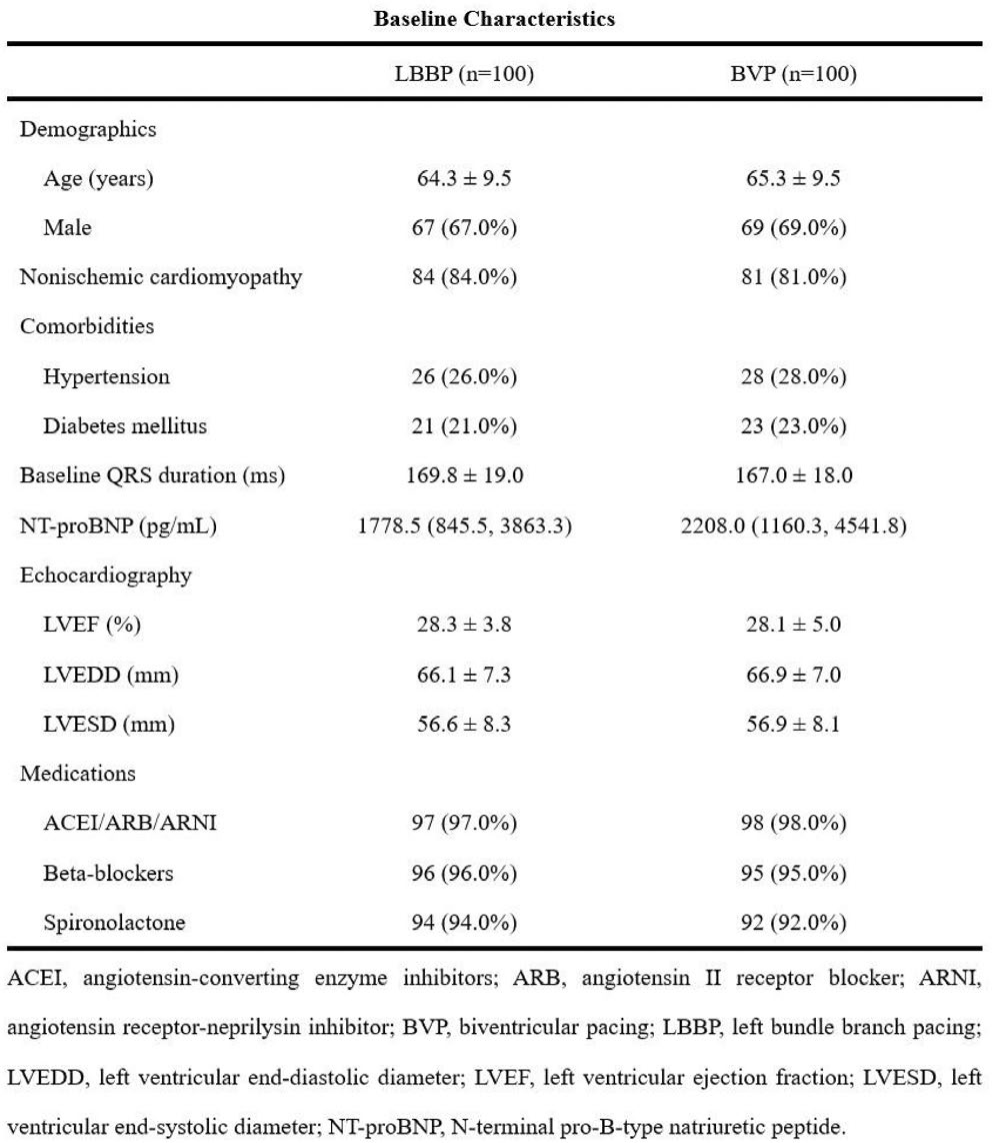

## 63 ‘LIFE’S ESSENTIAL 8’ CARDIOVASCULAR HEALTH AND RISK OF INCIDENT ARRHYTHMIAS: A PROSPECTIVE COHORT STUDY

### 63.1 **TIANXIN LONG**, SIJING CHENG, HAO HUANG, BINGQI FU, WEI HUA, YONGJIAN WU

#### 63.1.1 Fuwai hospital, Chinese Academy of Medical Sciences, Beijing, China

**Introduction:** Cardiovascular health (CVH) was proposed to improve cardiovascular risk factors and was updated recently. However, association between CVH with Life's Essential 8 (LE8) approach and arrhythmia risk is still unknown.

**Methods:** A total of 111,239 participants from UK biobank who were free cardiovascular diseases and arrhythmia were included. CVH score (range from 0 to 100) was assessed using LE8 metrics and categorized into low, moderate and high, according to American Heart Association definitions. Cox proportional‐hazards and restricted cubic spline models were used to assess the associations of CVH with the risk of cardiac arrhythmia.

**Results:** During a median follow‐up of 11 years, we identified 5560 cases (4.99%) of atrial fibrillation (AF), 722 cases (0.65%) of ventricular arrhythmias and 2234 cases (2.01%) of bradyarrhythmia. High CVH was significantly associated with lower risk of AF (HR: 0.63; 95%CI: 0.56‐0.71), ventricular arrhythmias (HR:0.42, 95%CI: 0.31‐0.58) and bradyarrhythmia (HR:0.62, 95%CI: 0.52‐0.75) and a nonlinear dose‐response relationship was observed, after adjustment for potential confounders. Every standard deviation increment of LE8 was associated with a 13%, 22% and 15% lower risk of incident AF, ventricular arrhythmias and bradyarrhythmia, respectively. Additionally, no significant interaction was found between LE8 score and AF genetic risks. Stratified analysis showed a consistent association between CVH and risk of incident AF across different AF polygenic risk score (PRS) levels. Compared with participants with high PRS and low CVH, participants with low PRS and high CVH experienced the lowest risk (HR: 0.21; 95% CI: 0.17, 0.27) of incident AF.

**Conclusions:** Our findings suggest that maintaining optimal CVH should be recommended as a preventive strategy for arrhythmias.

## 64 LONG‐TERM OUTCOMES OF LEADLESS PERMANENT PACING: A SINGLE CENTRE AUSTRALIAN EXPERIENCE

### 64.1 **EHSAN MAHMOODI**, XIANG WEN LEE, BLAKE FREEMAN, MEGHAN WEBSTER, JOHN BETTS, HARIS HAQQANI, RUSSELL DENMAN

#### 64.1.1 The Prince Charles Hospital, Brisbane, Australia

**Introduction:** Leadless pacemakers (LP) have emerged as an alternative to conventional transvenous pacemakers in patients with bradyarrhythmias, mitigating lead and pocket related complications associated with transvenous pacemakers.

**Methods:** We report the long‐term clinical outcomes of consecutive patients who underwent LP implantation from 2016 to 2024 at a single tertiary centre.

**Results:** 179 patients (median age 82, 62.5% male) underwent LP implantation, with 162 single chamber (Micra VR) and 17 dual chamber (Micra AV) LPs. 158 patients (88.3%) had chronic atrial fibrillation, 109 patients (61.6%) had heart failure, and the median left ventricular ejection fraction was 55% (50‐62%). 55 patients (30%) had previous transvenous pacemakers requiring device extraction mainly due to lead or pocket related infection. 97.2% of the procedures were performed with conscious sedation and the median procedure time was 36 minutes (30‐50). Intraprocedural acute dislodgement occurred in one patient with successful snaring and reimplantation of LP. The median R wave at implant was 10.9 mV (6.9‐14.5), median capture threshold at 0.24 ms was 0.5 V (0.4‐0.8) and the median impedance was 740 ohms (650‐880). The median length of hospital stay was 1 day (1‐3), and 5 patients (2.8%) developed complications during admission including 3 patients with small groin haematoma, 1 patient with acute battery depletion, and 1 patient with high capture threshold on day 1 post implant, with successful device reimplantation in both patients. The median R wave at the last follow up was 13.8 mV (9.4‐19), median capture threshold at 0.24 ms was 0.5 V (0.375‐0.625), and the median impedance was 560 ohms (490‐670). During a median follow‐up of 2.3 years (0.3‐4.6), 2 patients (1.1%) required device upgrade to transvenous system, and 52 patients (29%) died, none of which were due to device related complications.

**Conclusions:** Leadless pacemakers can be implanted safely and effectively with good electrical performance and low risk of long‐term complications. Further studies are required to assess the long‐term performance and complications of LP.

## 65 ABNORMAL PET‐CT SCAN FINDINGS IN PATIENTS≤60 YEARS OF AGE PRESENTING WITH AV BLOCK

### 65.1 **ANAND MANICKAVASAGAM**, JOHN ROSHAN, SIRISH CHANDRA SRINATH, DAVID CHASE

#### 65.1.1 Christian Medical College, Ranipet, India

**Introduction:** AV block in the age group ≤ 60 years may be idiopathic or an initial manifestation of systemic diseases such as sarcoidosis. This study aims to assess the prevalence of abnormal FDG‐PET scan in patients ≤ 60 years presenting with unexplained AV Block undergoing FDG‐PET scan to rule out sarcoidosis.

**Methods:** Retrospective study. Patients undergoing pacemaker implantation between 1^st^ January 2022 to 30^th^ June 2023 in a tertiary care hospital in south India were considered. Inclusion criteria: 1. Patients who underwent pacemaker implantation for AV block. 2. Age ≤ 60 years. 3. Normal LV systolic function. 4.No other obvious cause for AV Block. 5.Underwent FDG‐PET sarcoidosis protocol.

**Results:** 80 patients underwent pacemaker implantation for AV block with no obvious cause and of these 19 patients underwent PET‐CT scan for sarcoidosis. 8 patients had abnormal PET CT scan. Of these 6 patients had myocardial uptake and 2 patients had uptake in the lung and lymph node without myocardial uptake. The clinical characteristics were similar between the PET positive and PET negative group (Table). Based on the PET scan uptake, three patients had mediastinal lymph node biopsy and it showed granulomatous inflammation. Four patients were treated with immunosuppression for cardiac sarcoidosis based on FDG‐PET abnormality and of these four patients, three patients had improvement in AV conduction.

**Conclusions:** 8 (42%) patients presenting with unexplained AV block in young had abnormal FDG‐PET scan and 4 (21.5%) patients were started on immunosuppression for probable cardiac sarcoidosis. Cardiac Sarcoidosis should be suspected in all patients ≤ 60 years with AV block with no known cause even though there are no features of sarcoidosis on echocardiogram.

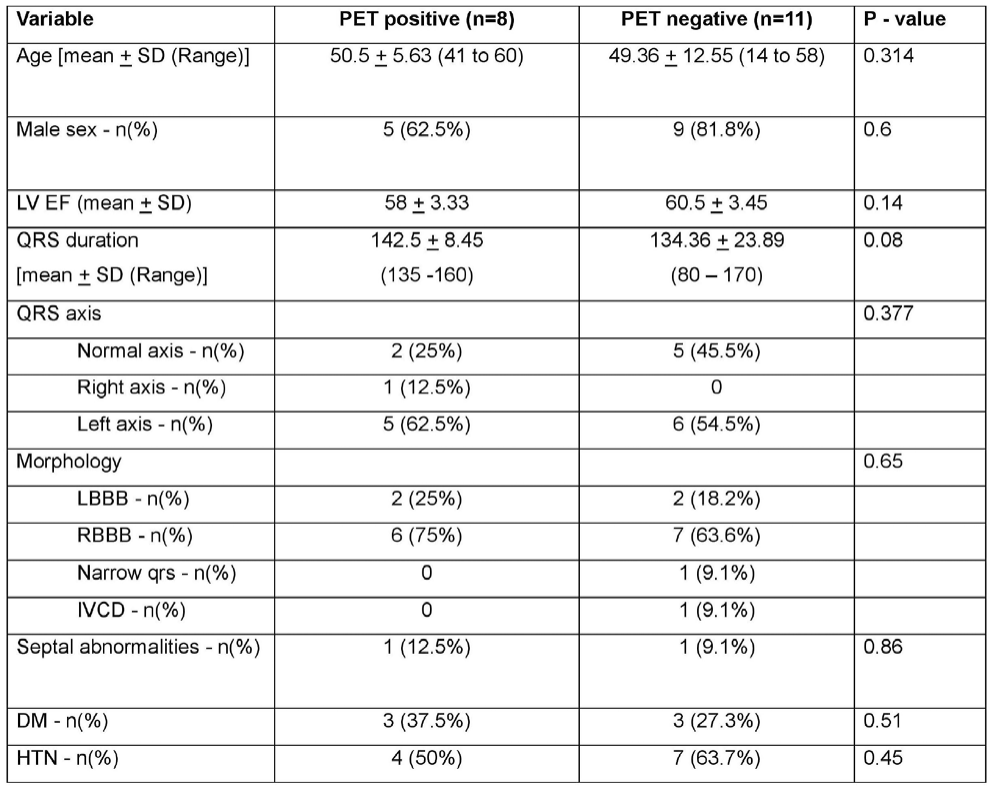

Chair

**L. Marcantoni**

Rovigo General Hospital, Rovigo, Italy

## 66 THE “420” CANNABIS CELEBRATION AND ATRIAL FIBRILLATION

### 66.1 JEAN JACQUES NOUBIAP, **GREGORY M. MARCUS**

#### 66.1.1 University of California, San Francisco, San Francisco, CA

**Introduction:** Data on the association between cannabis use and incident atrial fibrillation (AF) are conflicting, and no previous study has assessed if cannabis might acutely influence discrete AF episodes. This study aimed to determine whether an increase in cannabis use in the general population is associated with more acute healthcare utilization for AF.

**Methods:** California's Department of Health Care Access and Information databases were used to identify healthcare encounters of adults aged ≥18 years between 2005 and 2019. Diagnoses were identified using ICD‐9 and ICD‐10 codes. April 20^th^ (or “420” a day when cannabis is more commonly consumed) as an instrumental variable event was compared with all other days of the year. The outcomes were the number of emergency department visits or hospital admissions with a diagnosis of AF. Cannabis‐related disorders were assessed as positive control; amphetamine‐related disorders, abdominal hernia, appendicitis, and sarcoma were evaluated as negative controls. Poisson regression models adjusted for day of week, month, and year were performed to estimate the relationship between April 20th and each outcome.

**Results:** Among 27,780,762 patients experiencing 146,506,714 healthcare encounters in 2005‐2019 in California, 420 was associated with an increase in the number of healthcare encounters with a diagnosis of AF (incidence rate ratio [IRR] 1.012, 95% confidence interval [CI] 1.002‐1.022) and for the positive control cannabis‐related disorders (IRR 1.054, 95% CI 1.033‐1.075). No association was observed between 420 and healthcare utilization for the negative controls (amphetamine‐related disorders, abdominal hernia, appendicitis, or sarcoma).

**Conclusions:** These findings suggest that cannabis may trigger acute AF episodes detectable at the level of the general population. This has implications related to the consequences of cannabis consumption and the prevention of AF.

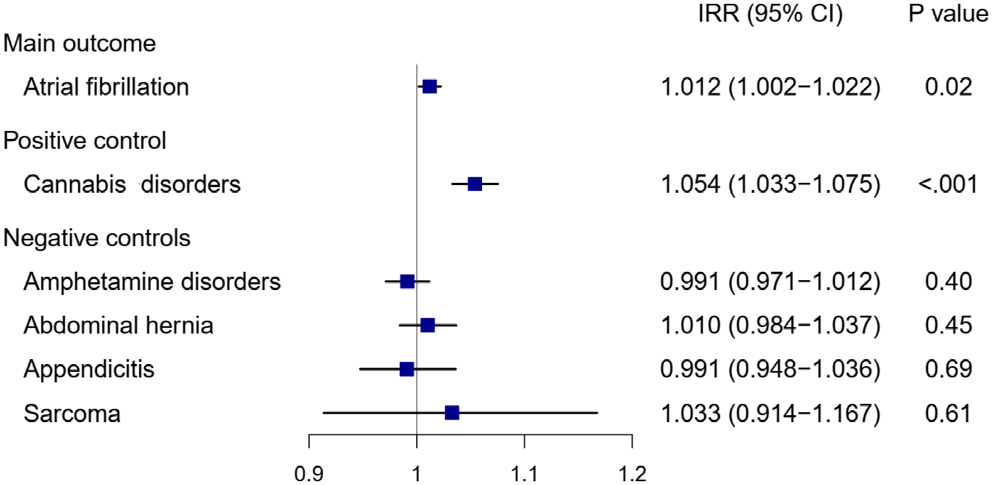

## 67 CLINICAL CHARACTERISTICS AND OUTCOME OF YOUNG PATIENTS WITH ATRIAL FIBRILLATION UNDERGOING CATHETER ABLATION FOR NON‐PULMONARY VEIN TRIGGERS

### 67.1 **HIROHIDE MATSUURA**, ARIHIDE OKAHARA, MASAKI TOKUTOME, SHUNSUKE KAWAI, KIYOHIRO OGAWA, RYUUICHI MATSUKAWA, YASUSHI MUKAI

#### 67.1.1 Japanese Red Cross Fukuoka Hospital, Fukuoka, Japan

**Introduction:** Little is known regarding the clinical characteristics and outcome of young patients with atrial fibrillation (AF) who required catheter ablation (CA) for non‐pulmonary vein triggers (nPV triggers).

**Methods:** From 4/1/2019 to 12/31/2021, 835 AF catheter ablation (AFCA) procedures (769 patients) were performed at our institution, of which 100 patients (104 cases, mean age 67.6 ± 10.9 years, 66.0% male) underwent nPV trigger ablation. The analysis was conducted separately for the younger group (<60 years old, N=20) and the non‐younger group (>60 years old, N=80). The distribution of nPV triggers was classified into left atrial origin, septal origin, right atrial origin, and superior vena cava origin.

**Results:** The percentage of paroxysmal AF and the sites of nPV trigger were comparable between the younger group and the non‐younger group, however the number of nPV triggers was significantly high in the younger group. The Kaplan‐Meier curve showed the significantly lower rate of freedom from atrial tachycardia/AF recurrence rate in the younger group (49.1%) compared to the non‐younger group (64.2%, P<0.05).

**Conclusions:** The younger group that required nPV trigger ablation had a significantly higher recurrence rate of atrial tachycardia and AF than the non‐younger group. There was no difference in the distribution of nPV triggers between the two groups, however there was a significant difference in the number of nPV triggers, which may be related to the recurrence rate.

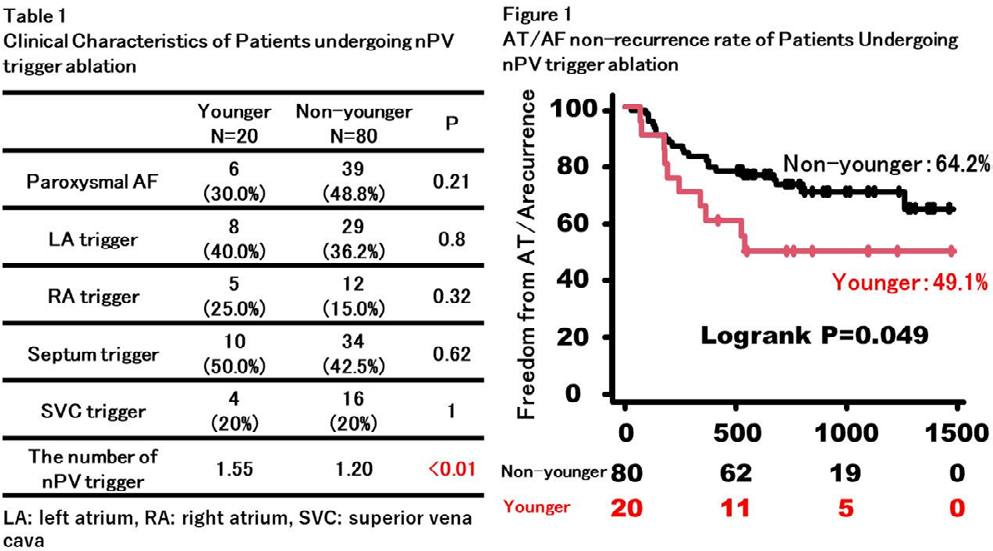

## 68 PEAK FREQUENCY ANNOTATION ALGORITHM FOR REVEALING END‐EPI CONNECTION IN LEFT ATRIAL POSTERIOR WALL ISOLATION

### 68.1 **KENTARO MINAMI**, IKUO ATAGI, KOHKI INOUE, KEITARO IIDA, IKUTA SAITO, TAIKI MASUYAMA, YOSHIYUKI KITAGAWA, HIYORI OYAMA, TOSHIAKI NAKAJIMA, SHIGERU TOYODA

#### 68.1.1 Dokkyo Medical University, Mibu, Shimotsugagun, Tochigi, Japan

**Introduction:** The left atrial posterior wall (LAPW) can be a target for atrial fibrillation (AF) catheter ablation. However, it is sometimes difficult to isolate due to the presence of endocardial‐epicardial connections, such as the sept‐pulmonary bundle. Omnipolar mapping technology (OMT) can automatically annotate the peak frequency (PF) potential associated with acquired intracardiac electrograms. This study aims to verify the PF annotation algorithm's effectiveness in revealing the end‐epi connection in left atrial posterior wall isolation.

**Methods:** We retrospectively studied 177 AF patients who underwent the LAPW electrical isolation after the pulmonary vein isolation. Omipolar mapping was performed after a first‐pass linear ablation along the superior and inferior LAPW.

**Results:** A first‐pass LAPW isolation was achieved in 130 patients (73.4%). Among the 47 patients, the line gaps at the superior or inferior line were observed in 7 patients (end gaps). High‐frequency (HF) sites ( &gt;300 Hz) located more than 5 mm away from the lines in LAPW were found in 40 patients (22.6%). In 40 patients, the RF application to the HF site more than 5 mm away from the line successfully achieved LAPW isolation (end‐epi gaps). The PF was significantly higher at the end‐epi gap site than at the end gap site (median [IQR]; 498.5 [468‐562] vs 202.1[181‐232], *p* &lt;0.001).

**Conclusions:** A PF annotation algorithm accurately identifies areas of the breakout site of the end‐epi connection. The high‐frequency site in the LAPW after a first‐pass liner ablation could be an ablation target site to complete LAPW isolation.

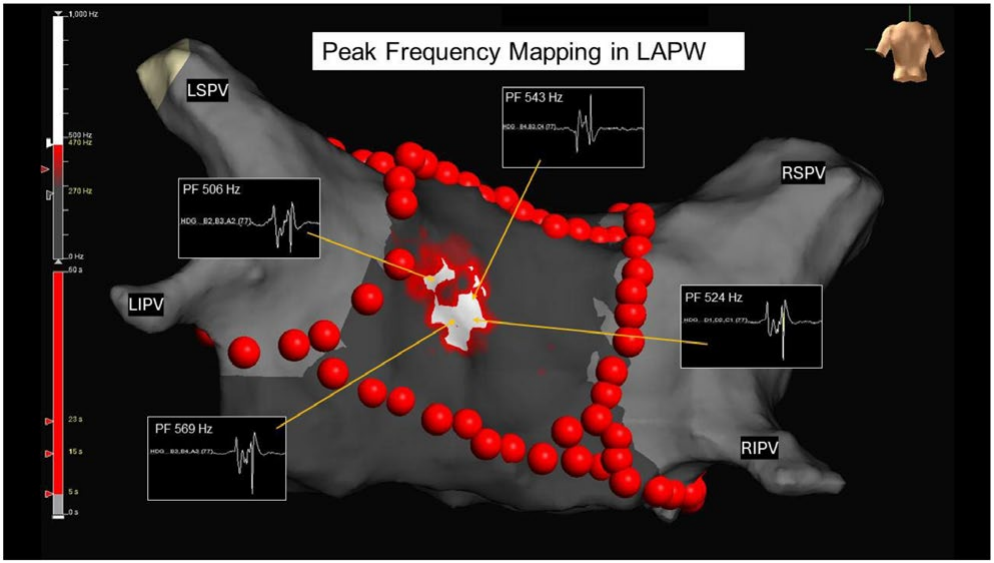

## 69 ELUCIDATING THE IMPACT OF THE KCNH2 G53S PAS DOMAIN VARIANT ON TYPE 2 LONG QT SYNDROME THROUGH HIPSC‐DERIVED CARDIOMYOCYTES

### 69.1 **DASOM MUN** ^1^, JI‐YOUNG KANG^1^, MALGEUM PARK^1^, GYEONGSEO YOO^1^, YOU MI HWANG^2^, BOYOUNG JOUNG^1^

#### 69.1.1 ^1^Yonsei University, Seoul, Korea, Republic of,^2^Catholic University, Suwon, Korea, Republic of

**Introduction:** Long QT syndrome type 2 (LQT2) is a heart disease caused by loss‐of‐function mutation in the KCNH2 gene, resulting in impaired Kv11.1 channel function. The KCNH2 gene encodes the α‐subunit of the rapid‐acting inward rectifying potassium (Ikr) channel. Despite the critical impact of these mutations, a significant gap remains in our understanding of the specific mechanisms, mainly due to the lack of disease‐specific, patient‐derived in vitro models.This study aims to advance the understanding of LQT2 pathophysiology by employing patient‐specific hiPSC‐derived ventricle cardiomyocytes (hiPSC‐vCMs), focusing on the electrophysiological alteration to explore the cellular consequences of the KCNH2 G53 PAS domain variant.

**Methods:** hiPSC‐vCMs were generated from iPSCs of a patient harboring a KCNH2 G53 PAS domain variant and an unrelated healthy control iPSCs using the non‐integrative Sendai virus‐mediated iPSC reprogramming method. Electrophysiological properties of KCNH2 mutation were assessed with field potential, action potential duration (APD) restitution, wavelength (WL) and conduction velocity (CV) restitution using microelectrode arrays (MEA). Additionally, Ca^2+^ dynamics in hiPSC‐vCMs were characterized using Fluo‐4 dye.

**Results:** Following the differentiation of hiPSC‐vCMs from LQT2 patients, Immunocytochemistry confirmed robust expression of cardiomyocyte markers (Nkx2.5 and cTNI). KCNH2‐G53S hiPSC‐vCMs exhibited a lower expression of KCNH2 expression compared to normal cardiomyocytes. These KCNH2‐G53S hiPSC‐vCMs demonstrated prolonged APD90 and abnormal Ca2^+^ handling properties such as increased amplitude and enhanced Ca2+ leak compared to WT hiPSC‐vCMs. Notably, functional assessment of calcium homeostasis revealed that the KCNH2 mutation contributes to atrial arrhythmias by impairing calcium handling genes.

**Conclusions:** This study provides an understanding of electrical remodelling caused by the KCNH2 G53S PAS Domain Variant on LQT2, leading to an understanding of the development of arrhythmias.

## 70 THE EFFICACY OF 2‐WEEK HOLTER MONITORING FOR DETECTING ATRIAL TACHYARRHYTHMIA RECURRENCE AFTER THE INITIAL ABLATION IN PATIENTS WITH ATRIAL FIBRILLATION

### 70.1 **HIROKAZU NAGANAWA**, YUKO UEMURA, RYO YAMAGUCHI, DAISUKE YOSHIMOTO, YUICHIRO SAKAMOTO, TAKAHIKO SUZUKI

#### 70.1.1 Toyohashi Heart Center, Toyohashi, Japan

**Introduction:** Holter monitoring is widely used to detect atrial tachyarrhythmia recurrence and to evaluate long‐term outcomes after catheter ablation (CA) in patients with atrial fibrillation (AF). Patients with AF after CA often have poor subjective symptoms, and recurrence may be underdiagnosed. 2‐week Holter monitoring can be more helpful in detecting atrial tachyarrhythmia recurrence than 24‐hour Holter monitoring. Nonetheless, there is a paucity of current literature discussing strict follow‐up by 2‐week Holter monitoring after CA for patients with AF. Therefore, this study seeks to evaluate the efficacy of 2‐week Holter monitoring for detecting atrial tachyarrhythmia recurrence.

**Methods:** From January 2019 to December 2021 at our center, a total of 755 consecutive AF patients (Paroxysmal:449, Persistent:256, Long‐standing:50) who underwent initial CA of wide‐area pulmonary vein isolation using the CARTO system (Biosense Webster) were enrolled in this study. 2‐week Holter monitoring was conducted 3, 6, 12, 18, and 24 months after CA. 24‐hour Holter monitoring was substituted for the first 24 hours of 2‐week Holter monitoring. Freedom from atrial tachyarrhythmia recurrence was defined as the absence of > 30 seconds of atrial tachyarrhythmia beyond a 3‐month blanking period.

**Results:** Sixty‐nine patients (9.1%) dropped out of the follow‐up schedule. In the remaining 686 patients, atrial tachyarrhythmia recurrence was detected in 173 cases (25.2%) and 46 cases (6.7%) in the 2‐week and 24‐hour Holter monitoring groups, respectively (P < 0.001), during the 2‐year follow‐up period. Holter monitoring‐based analysis revealed that asymptomatic recurrence was significantly higher in the persistent and long‐standing AF group (85.0%, 96/113 records) compared to paroxysmal AF (50.0%, 76/152 records) (P<0.001).

**Conclusions:** 2‐week Holter monitoring was more effective than conventional 24‐hour Holter monitoring for detecting atrial tachyarrhythmia recurrence after CA, especially in the persistent and long‐standing AF group.

## 71 OUTCOMES OF PREMATURE VENTRICULAR COMPLEX ABLATION IN HEART FAILURE WITH MID RANGE EJECTION FRACTION

### 71.1 **ANUGRAH NAIR**, JENISH SHROFF, LUKAH TUAN, LORI BELL, NATASHA JONES‐LEWIS, RAJEEV PATHAK

#### 71.1.1 CANBERRA HEART RHYTHM, CANBERRA, Australia

**Introduction: Objective:** This study aimed to evaluate the clinical outcomes of PVC ablation in HFmrEF patients.

**Methods: Methods:** Of the 148 patients between 2018‐2024, 31 PVC patients with LVEF 41‐50% who underwent ablation were included. Change in PVC burden at 6 weeks and 12 months after ablation, improvement in LVEF at 12 months, and need for redo procedure were analysed.

**Results:** Results: Out of the 31 patients, 6 had Para‐hisian PVCs (19.35%), 5 papillary muscle PVCs (16.12%), 4 Mitral annular PVCs (12.9%), 3 septal PVCs (9.7%), 8 LVOT (25.8%) PVCs and 5 RVOT (16.12%) PVCs. 10 had ischemic cardiomyopathy (32.25%), 16 had non‐ischemic cardiomyopathy (51.6%) and 5(16.12%) had mixed cardiomyopathy. The mean PVC burden reduced over 6‐week and 12‐month follow‐ups [baseline 18.8±8.8% to 1.01±1.4% at 6 weeks and 1.63±3.6% at 12 months respectively(P<0.05)]. The mean LVEF improved over a 12‐month follow‐up period [baseline 41.5±4.8% to 51±8.8% at 12 months (p<0.05)]. Acute success seen in 96.77% of cases and success at 12 months was seen in 87.09%. 13 patients had myocardial scar (41.9%). Patients without a scar had a greater reduction of PVC burden at 12 months (94.66±4.4% vs 86.19±5.6% with scar). 2 patients required a redo procedure.

**Conclusions:** Conclusion: PVC ablation was associated with improved clinical outcomes in HFmrEF patients. The mean PVC burden reduced and mean LVEF improved significantly. Majority had successful suppression of PVC and only minor number of patients required a redo procedure.

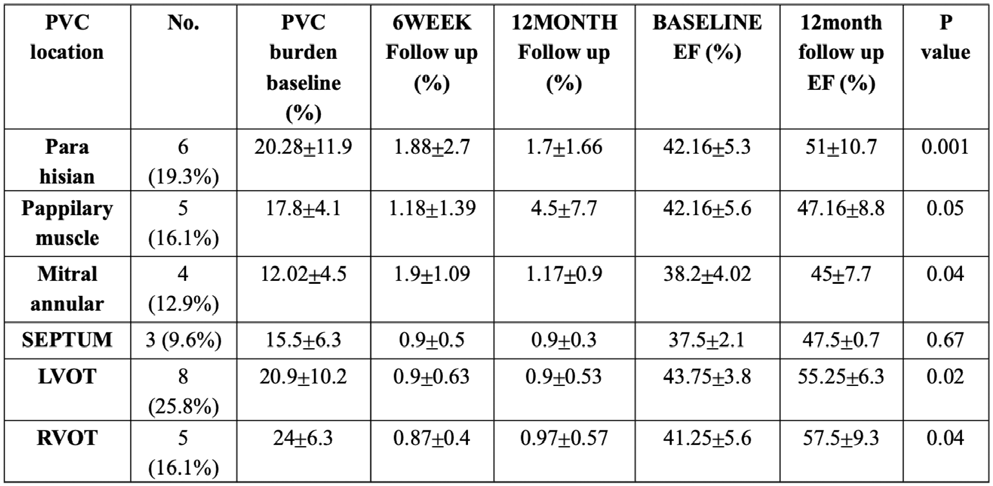

## 72 ATRIAL HELIX‐FIXATION LEADLESS PACEMAKER: INITIAL REAL‐WORLD EXPERIENCE

### 72.1 **DEVI NAIR** ^1^, KEN W LEE^2^, GANESH NAIR^3^, KIROLLOS GABRAH^3^, BRANDON DOTY^3^, NIMA BADIE^4^, KYUNGMOO RYU^4^, CYRUS HADADI^5^

#### 72.1.1 ^1^St. Bernards Medical Center & Arrhythmia Research Group, Jonesboro, AR,^2^Health First Medical Group, Melbourne, FL,^3^Arrhythmia Research Group, Jonesboro, AR,^4^Abbott, Sunnyvale, CA,^5^3.MedStar Heart and Vascular Institute, MedStar Washington Hospital Center,, Washington, DC

**Introduction:** The Aveir™ AR pacemaker (Abbott) is a new single‐chamber leadless pacemaker (LP) designed specifically for the right atrium. The initial commercial implant experience with this atrial LP has yet to be evaluated.

**Methods:** Sinus node dysfunction patients to be implanted with a de novo Aveir AR device in the US after commercial release were consecutively included in this study. Procedural characteristics were evaluated, and electrical parameters were measured during pre‐fixation mapping, post‐fixation tether mode, after LP release, and prior to patient discharge. Any acute procedure‐ or device‐related complications within 30 days were noted.

**Results:** Forty‐two (42) Patients were implanted with Aveir AR per standard practice at 3 centers (73±13 years; 52% male), with ALPs placed predominantly in the base of the right atrial appendage (76%). Pre‐fixation mapping allowed repositioning to be avoided in 83% of patients. The total procedure duration was 38±28 min (range: 15‐130 min), from initial incision to final suture, with a total fluoroscopy duration of 9±9 min (range: 2‐42 min). By patient discharge, pacing capture thresholds and sensed P‐wave amplitudes significantly improved, and impedances remained stable (see figure). No perforations or other acute complications were observed.

**Conclusions:** The initial real‐world experience of the helix‐fixation, single‐chamber, atrial leadless pacemaker demonstrated safe and efficient implantation with clinically viable electrical metrics and no acute complications.

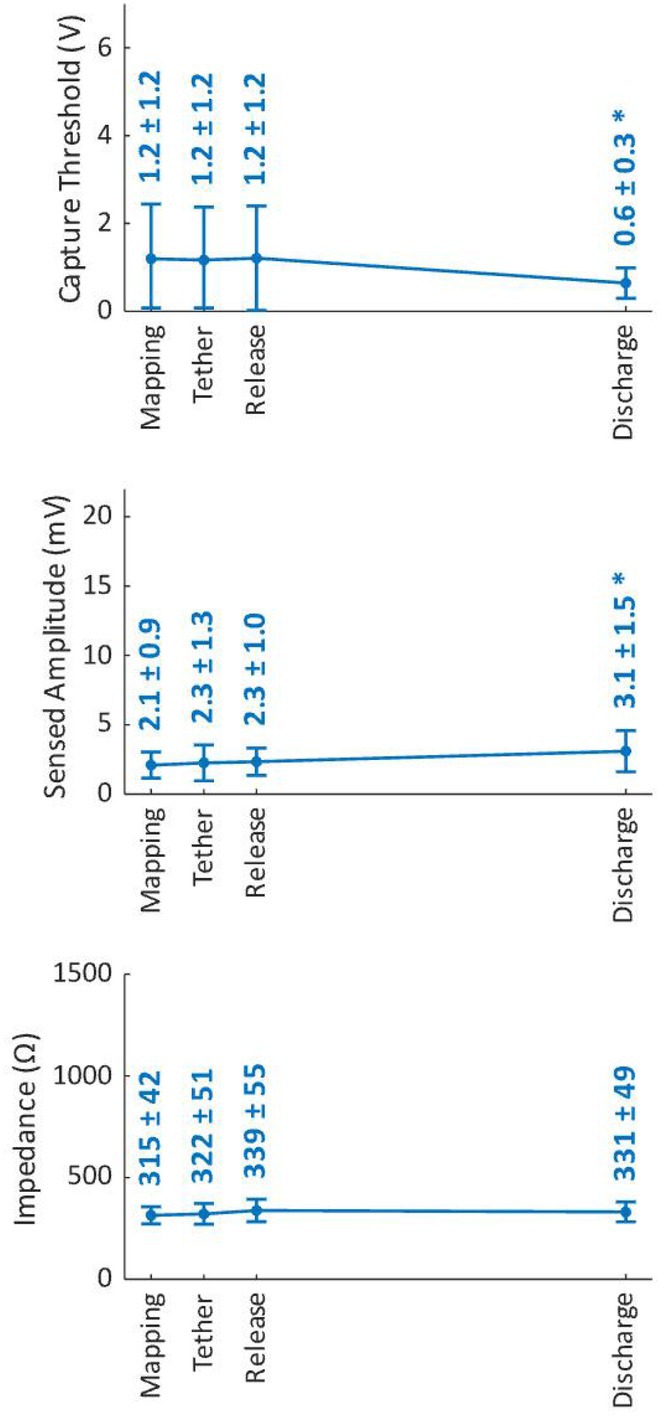

## 73 FIRST INVASIVE REMAPPING TO ASSESS LONG TERM DURABILITY OF PULMONARY VEIN ISOLATION USING A CIRCULAR PULSED FIELD ABLATION CATHETER

### 73.1 **DEVI NAIR** ^1^, GANESH NAIR^2^, KIROLLOS GABRAH^2^, BRANDON DOTY^1^, RACHELLE KAPLON^3^

#### 73.1.1 ^1^St. Bernards Medical Center & Arrhythmia Research Group, Jonesboro, AR,^2^Arrhythmia Research Group, Jonesboro, AR,^3^Medtronic Cardiac Ablation Solutions, Mounds View, MN

**Introduction:** Pulsed field ablation (PFA) is a novel nonthermal ablation modality for treating atrial fibrillation (AF) through irreversible electroporation. PFA causes rapid disappearance of intracardiac electrocardiograms, but chronic lesion formation and durability are less defined across PFA systems. This study evaluates lesion durability during the initial real‐world use of a circular PFA catheter.

**Methods:** Lesion durability was assessed in a consecutive cohort of AF patients undergoing left atrial appendage occlusion (LAAO) ~6‐10 weeks after first index pulmonary vein isolation (PVI) using the PulseSelect™ Pulsed Field Ablation System (Medtronic). Durable PVI was evaluated by confirming entrance and exit block using a commercially available mapping catheter (Octaray / HD Grid ) and electroanatomical mapping system (EAM) (Carto / EnSite X).

**Results:** A total of 25 patients (56% paroxysmal AF, 52% male, 69±9 years, CHA2DS2VASc 4±2) underwent PVI and subsequent invasive remapping. All index ablation procedures were conducted using intracardiac echocardiography and EAM without fluoroscopy. General anesthesia was used in 24/25 patients, and all patients were discharged on the same day. PV anatomical variations treated included right middle (n=5), left middle (n=1), and left common (n=2) PVs; additionally, superior vena cava (SVC) isolation was completed in 9 patients. An average of 55±5 PFA applications were placed during a skin‐to‐skin procedure time of 36±6 minutes. Acute PV isolation was achieved in 100% of patients. Invasive remapping 57±9 days post‐ablation demonstrated durable isolation in 98% of PVs (102/104), and 96% of patients (24/25) had all veins isolated. SVC isolation was durable in 9/9 patients. There were no complications during an average follow‐up of 74±18 days. Atrial arrhythmia recurrence was documented in 2/25 patients 39 and 45 days post‐ablation. Both patients had persistent AF, and all PVs were durably isolated on remapping.

**Conclusions:** Real‐world treatment of paroxysmal and persistent AF with a circular PFA catheter is safe, efficient, and results in durable lesion formation.

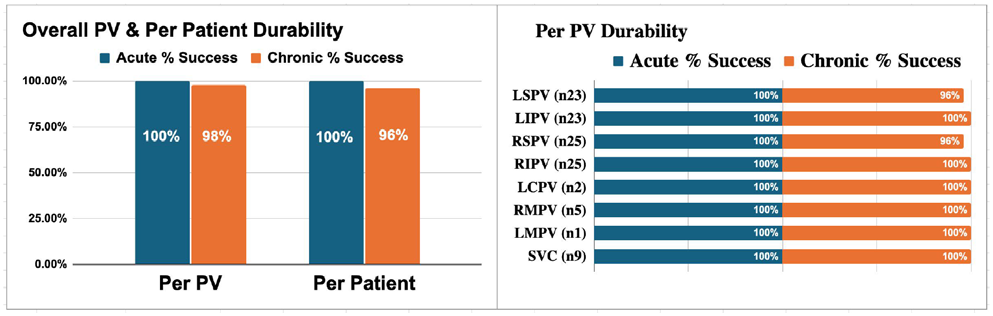

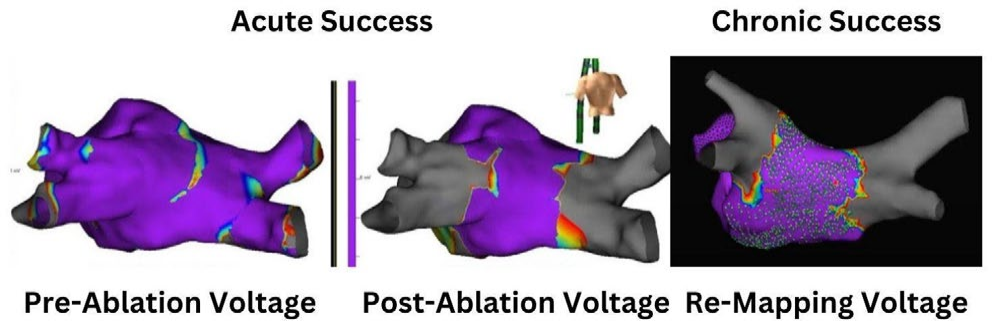

## 74 IMPACT OF 3D MAPPING ON INITIAL OPERATOR EXPERIENCE WITH PFA CATHETERS ‐ ACUTE FINDINGS & LEARNING CURVE

### 74.1 JOHN DAY^1^, HANY DEMO^2^, KEVIN TRULOCK^3^, CHRISTOPHER HEALY^3^, ANIL PUROHIT^4^, CHANUKYA R DAHAGAM^4^, EMILY WENZEL^5^, LAUREN GAETA^6^, PHILLIPP SOMMER^1^, **DEVI NAIR** ^7^

#### 74.1.1 ^1^MountainStar Healthcare, Salt Lake City, UT,^2^Swedish Hospital, Chicago, IL,^3^Community Heart & Vascular, Indianapolis, IN,^4^Community Heart and Vascular, Indianapolis, IN,^5^Abbott, St. Paul, MN,^6^Abbott, St Paul, MN,^7^St. Bernard's Healthcare, Jonesboro, AR

**Introduction:** Pulsed Field Ablation (PFA) technology has had an immediate impact on EP practice. Early experience in European trials has shown promise in safety and efficiency using primarily fluoroscopy‐based workflows. As PFA is available in more markets, workflows using 3‐dimensional mapping (3DM) and ICE have emerged. Patterns of 3DM use and impact of new software for representative PFA catheter shape visualization are not yet well quantified.

**Methods:** Procedural data was collected prospectively in those using commercially available PFA tools (Farawave, BSX (FW) and PulseSelect, MDT) mapped on the EnSite X system (Abbott). Procedures were completed on software in which FW is visualized as a ring and on new software designed to show both catheter shapes ‐ basket and flower (EnSite v3.0.2). Analysis was completed on procedural metrics and learning curves.

**Results:** Acute procedural data from 310 PFA procedures was collected from 33 institutions and 71 EP operators in the US and Europe. Choice of PFA catheter, lesion set, pre‐/post‐mapping and PVI confirmation were at physician discretion. Indication for ablation was *de novo* AF (60% PAF; 40% PsAF); 77.4% *de novo*. Model and voltage map was created with Advisor HD Grid or Circular catheter in 72.3% of patients. Post‐map was created with this catheter in 68.7% of this group, identifying first‐pass isolation in 75.6% of *de novo* patients. This is a higher rate of acute PV gaps than in previously reported studies though experienced users (15+ cases) saw rates of 92% in this cohort. PFA was used to close gaps in 97.4%, 2.6% with RFA. Sub‐analysis of FW cases with new visualization software showed procedure and fluoroscopy time decreased with physician experience (Figure) and 20% of cases were completed with zero fluoroscopy. Physician comfort was surveyed and found to increase from 5.8 to 6.2 (scale 1‐7) when new software was used compared to previous version.

**Conclusions:** 3DM enables may identify gaps in PVI lines acutely, can be used to enable zero‐fluoro procedures, and may reduce physician learning curve with new PFA technologies. Additional analysis should be completed on long‐term impact of use of 3DM in PFA procedures.

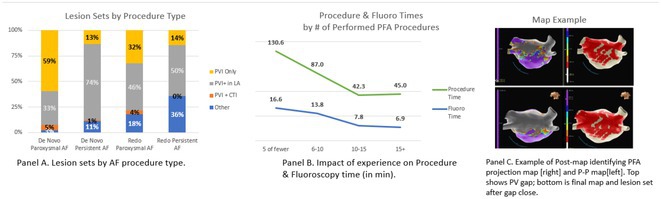

## 75 REDUCTION IN PVC BURDEN IMPROVES LEFT VENTRICULAR EJECTION FRACTION

### 75.1 **ANNA NATHER**, DAVID BEGLEY, CALIRE MARTIN, SHARAD AGARWAL

#### 75.1.1 Royal Papworth Hospital NHS Trust, Cambridge, United Kingdom

**Introduction:** Premature ventricular contraction (PVC) is frequently encountered in routine ECGs which may lead to reduction in ejection fraction (EF). This may be improved or even reversed with a medical or interventional reduction of ectopic burden. The primary objective of this study is to assess the potential effectiveness of cardiac ablation in suppressing PVC and subsequently enhancing the EF and to establish predictive factors of the success of the intervention

**Methods:** We analysed 116 patients who underwent PVC ablation and had LVEF <50%. Demographics, scar on MRI, EF and PVC burden was collected. Holter monitor and echo was assessed before and after ablation. Ablation procedure details were noted and predictors for improvement in LVEF was assessed. A reduction of PVC burden of at least 50% and improvement of LVEF by >5% was considered significant.

**Results:** Demographics are in table 1. It was found that patients participating in this study were predominantly male (66.4%), on average 60.1 (± 13.6) years old, overweight (BMI 29.8 ± 5.4) with no additional heart disease (56%) and no scarring on MRI (55%). The PVC burden before ablation (27.9 ± 12.7) significantly reduced to 8.0 ± 11.2 post‐ablation (p < 0.005). Successful interventions, defined by a reduction exceeding 50%, were observed in 79% of patients. The EF increased from 39.6 ± 8.9 before ablation to 47.5 ± 10.7 post‐ablation (p < 0.005), corresponding to an average increase by 8.4 ± 8.5. A strong correlation emerged between the relative change in PVC burden and LVEF improvement and a linear correlation was seen when the PVC burden was reduced by >75%. Higher BMI, presence of scar on the MRI and increasing age had a negative influence on the relative improvement in EF for reduction in the PVC burden. 35 patients had a F/U of around 90 weeks and 8 patients had recurrence of PVC with reversal of the improvement in EF.

**Conclusions:** In conclusion a relative reduction in PVC burden increases the EF after treatment. Increased BMI, age presence of scar on MRI negatively impacted LVEF recovery. Further research could improve the model that predicts the relative improvement in EF post ablation.

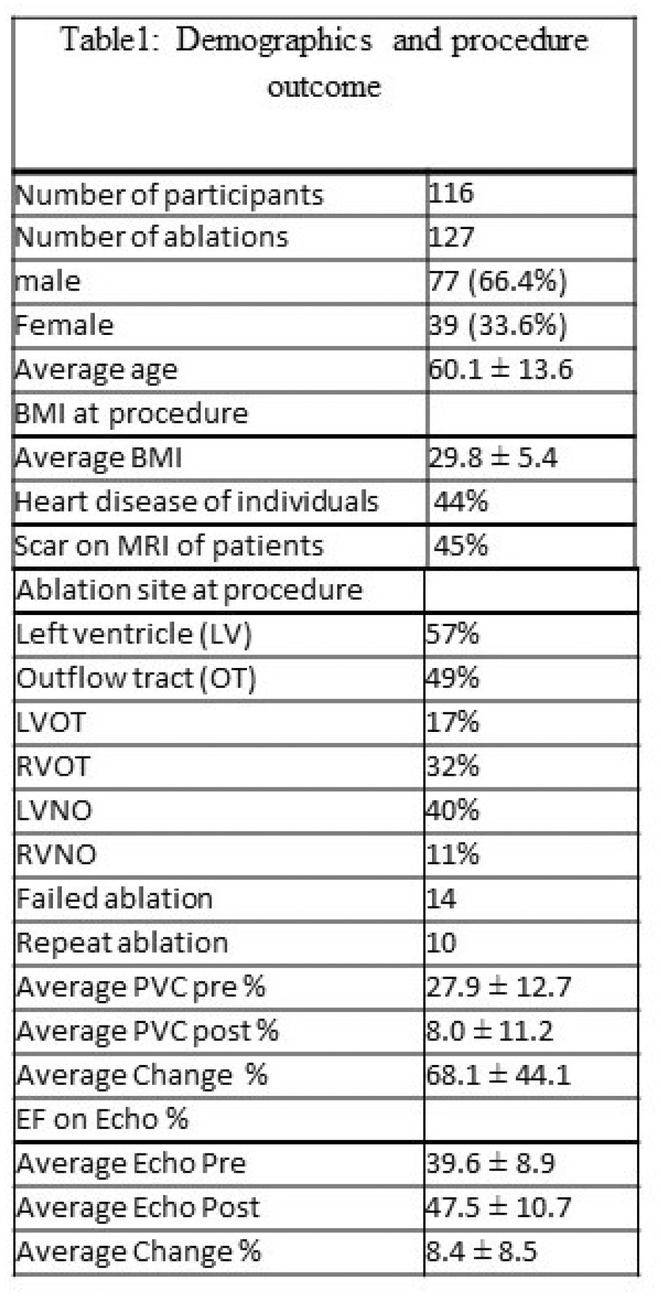

## 76 THE VALUE OF DIFFERENCE BETWEEN S‐WAVE AND R‐WAVE AMPLITUDES IN LEAD V1 AND V2 IN PREDICTING THE ORIGIN OF OUTFLOW TRACT VENTRICULAR ARRHYTHMIAS

### 76.1 **TUAN VIET NGUYEN** ^1^, DINH PHONG PHAN^2^

#### 76.1.1 ^1^Thanh Hoa Province General Hospital, Thanh Hoa, Viet Nam,^2^Vietnam National Heart Institute, Hanoi, Viet Nam

**Introduction:** Orienting the diagnosis of the location of ventricular arrhythmias from the ventricular outflow tract, including the right ventricular outflow tract (RVOT) and left ventricular outflow tract (LVOT), can help electrophysiologists limit the time exposed to X‐rays while also the number of vascular accesses that must be used during the procedure.

**Methods:** The study was conducted on 84 patients with confirmed diagnosis and successful arrhythmia ablation at the right ventricular outflow tract (RVOT group, n = 68) and left ventricular outflow tract (LVOT group, n = 16). The difference in total S‐R wave amplitude at V1 and V2 was measured and calculated on the surface 12‐lead electrocardiogram according to the formula: (V1S + V2S) ‐ (V1R + V2R)

**Results:** The difference between the total amplitute S‐R in lead V1 and V2 in the arrhythmia group originating from the LVOT was lower than the group originating from the RVOT (*p* < 0.001). The Cut‐off value to predict the site of origin (calculated by analyzing the ROC curve) was: 1,604 mV (Sensitivity: 93%, specificity: 84.7%, positive diagnostic value : 85.3%, negative diagnostic value: 91.7%). The area under the curve (AUC) was 0.875 (*p* < 0.001)

**Conclusions:** The difference between the total S‐R amplitude of >1,6 mV is a valued ECG criterion to predict RVOT rather than LVOT origin

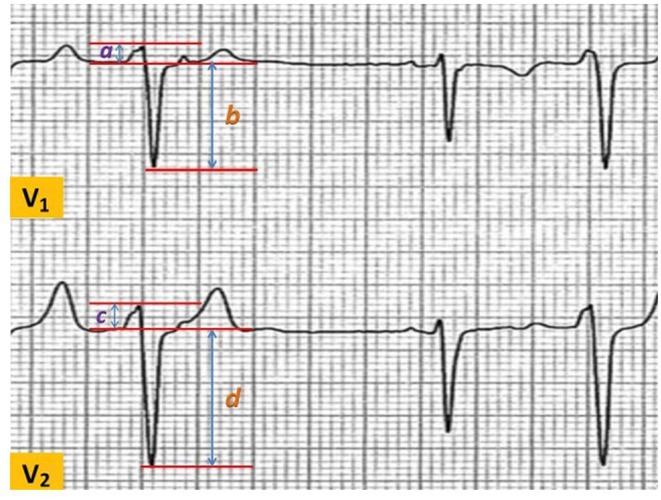

## 77 DOSE RESPONSE ALGORITHM VS WEIGHT BASED HEPARIN DOSING FOR INTRAPROCEDURAL ANTICOAGULATION DURING AF ABLATION

### 77.1 **JONATHAN O'LEARY** ^1,2^, TUPPENCE RICHMAN^3^, PHILLIP CUMPSTON^4,2^, MATTHEW TUNG^3,5^

#### 77.1.1 ^1^The Prince Charles Hospital, Brisbane, Australia,^2^Faculty of Medicine, The University of Queensland, Brisbane, Australia,^3^Sunshine Coast University Hospital and Health Network, Birtinya, Australia,^4^Greenslopes Private Hospital, Greenslopes, Australia,^5^School of Medicine and Dentistry, Griffith University, Birtinya, Australia

**Introduction:** Heparin dosing during atrial fibrillation ablation (AFA) is traditionally guided by weight alone with treatment effect monitored by Activated Clotting Time (ACT). An algorithm was developed utilising sex, weight, height, age, eGFR, platelet count, anticoagulant status and pre‐heparin ACT. The algorithm incorporated estimation of the heparin dose‐response curve and estimated blood volume using Nadler's formula. In our centre, heparin dosing utilised weight alone up until May 2022 when the dosing algorithm was adopted.

**Methods:** A retrospective cohort study was performed at our centre. Baseline patient characteristics, intraprocedural ACTs, and requirement for additional heparin bolus doses were recorded from consecutive AFA procedures prior to and after the change of dosing strategy. Cases with incomplete medical records were excluded. A mixed‐model 2x2 ANOVA was used to analyse the within‐subject variable accuracy of ACTs at two levels (1st and 2nd intraprocedural ACT), and between groups (weight alone based dosing and algorithm based dosing). Accuracy of ACT was defined as difference between target ACT (350) and recorded ACT.

**Results:** 187 consecutive patients who underwent AFA were included in the analysis. A mixed 2x2 ANOVA found a significant effect for method of heparin dosing and measured ACT, F (1, 175) = 13.741, p = <.001, partial η2 = .073 (medium effect size), with algorithm based dosing significantly more accurate (M = 23.22, SD = 18.09) than weight alone‐based heparin dosing (M = 39.51, SD = 35.67). There were no further significant within‐subject, or interaction effects found.

**Conclusions:** During AFA an algorithm incorporating dose response curves performed with higher accuracy achieving a 1^st^ and 2^nd^ ACT closer to target, compared with a traditional weight based dosing strategy in a retrospective comparative cohort.

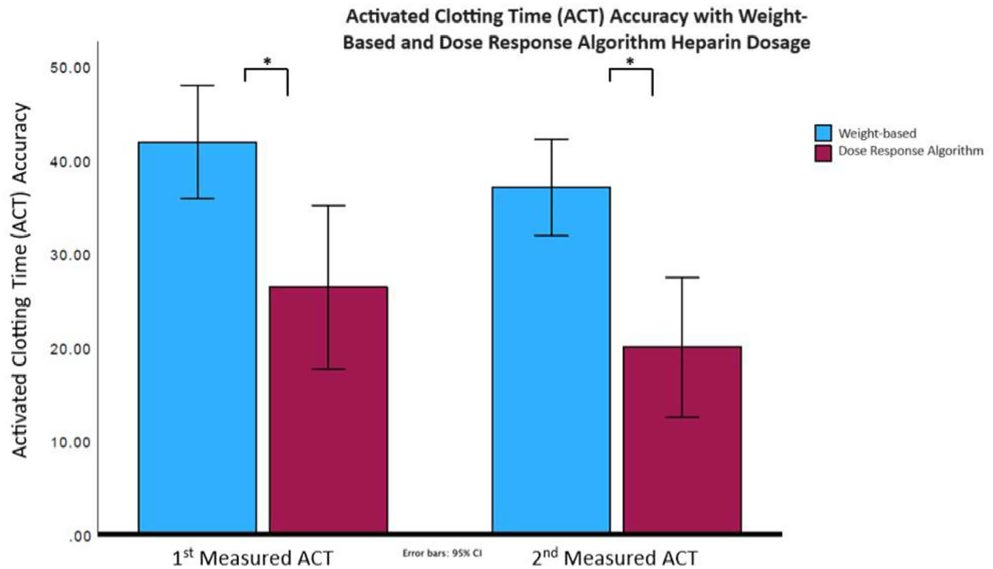

Chair

**U. Pandurangi**

Chair

**R. Rosso**

Tel Aviv Sourasky Medical Center, Tel Aviv, Israel

## 78 RANDOMISED CLINICAL TRIAL OF AGGRESSIVE LIFESTYLE AND RISK FACTOR MANAGEMENT FOR AF ‐ IMPACT ON ARRHYTHMIA BURDEN AND SUBSTRATE

### 78.1 **RAJEEV K. PATHAK** ^1,2^, ADRIAN ELLIOTT^3,4^, MELISSA MIDDLEDORP^3,4^, ABHINAV B. MEHTA^1,2^, DENNIS H. LAU^3^, SCOTT WILLOUGHBY^3^, JONATHAN M. KALMAN^5^, PRASHANTHAN SANDERS^3,4^

#### 78.1.1 ^1^Canberra Heart Rhythm, Canberra, Australia,^2^Australian National University, Canberra, Australia,^3^Centre for Heart Rhythm Disorders, Adelaide, Australia,^4^University of Adelaide, Adelaide, Australia,^5^University of Melbourne, Melbourne, Australia

**Introduction:** Cardiac risk factors (RF) such as hypertension, diabetes mellitus, obesity and sleep apnoea have been associated with structural and electrical remodeling of the atria that forms the substrate leading to the development and progression of atrial fibrillation (AF). We evaluated the impact of aggressive management of RFs on AF burden and the AF substrate.

**Methods:** 50 consecutive AF pts with BMI ≥ 27 kg/m^2^ and ≥1 cardiac risk factor were randomized to either RF management (RFM) or usual care (Control). The RFM group were managed in a physician‐led clinic directed at RF control in accordance with AHA/ACC guidelines with an aim of >10% weight loss. Both the groups were studied with electrophysiological (EP) study, echocardiography, cardiac MRI, serum fibrosis and endothelial function markers at baseline and repeated at > 12 months (or earlier if AF ablation was required). AF symptoms were quantified using the AF Symptom Severity Questionnaire and AF burden using implantable loop recorders.

**Results:** There were no differences in baseline characteristics or follow‐up duration (15±3 months) between the groups (p=NS). RFM resulted in greater reduction in weight (p<0.001), systolic blood pressure (p=0.004), better glycaemic control (p=0.003) and lipid profile (p=0.04), LA size (p=0.003) and total pericardial fat volume (p=0.001). RFM was associated with a significant reduction in AF symptom duration (P=0.002), frequency (P=0.001), severity (P=0.001) and AF burden (P<0.001) (Table). In addition, there was marked reversal in atrial electrical parameters, reduced pro‐inflammatory/fibrotic markers and improved endothelial/platelet function.

**Conclusions:** Aggressive risk factor management is associated with reduction in AF symptom and AF burden. RFM reverses electrical and structural remodeling and results in a significant improvement in endothelial and platelet function underscoring its importance in AF management. (Clinical Trial Registration: ACTRN12613000444785).

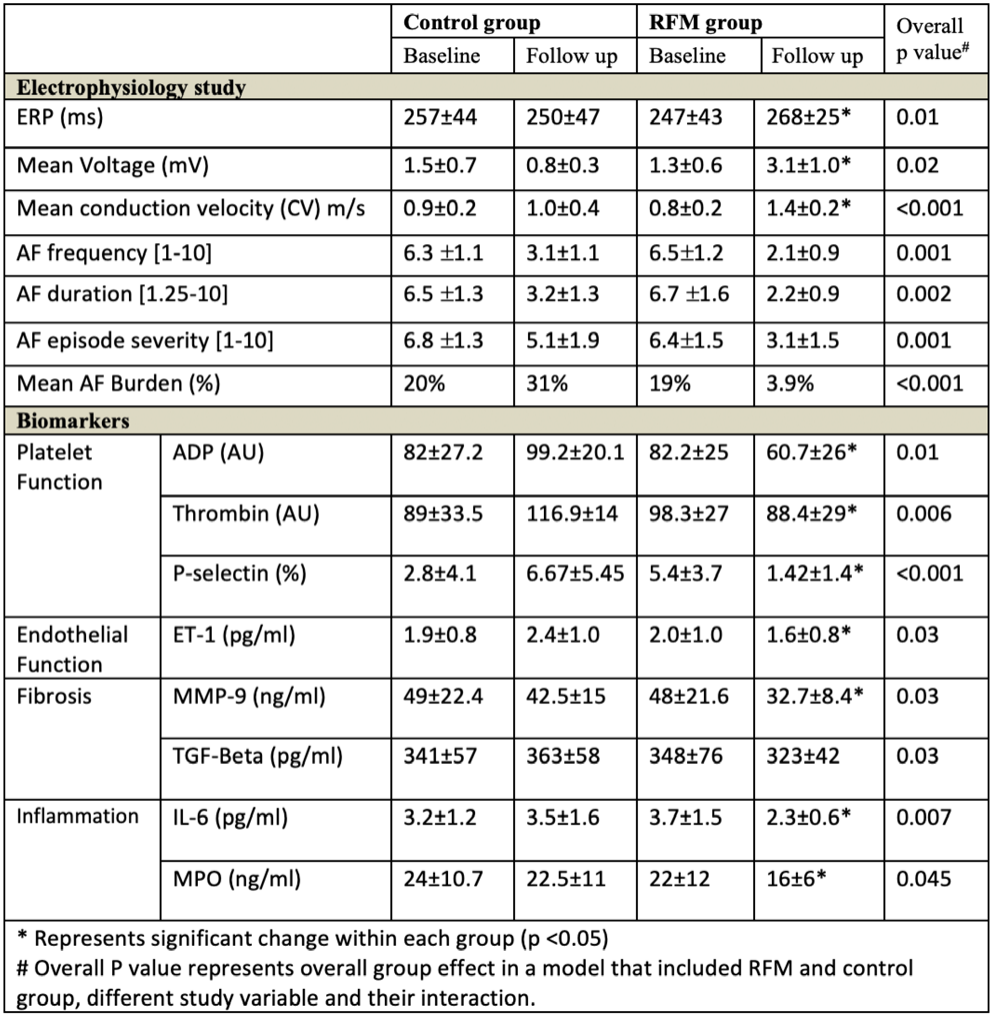

## 79 ELECTROPHYSIOLOGICAL CHARACTERISTICS AND CLINICAL OUTCOMES OF RIGHT VENTRICULAR ACTIVATION DURING LEFT BUNDLE BRANCH AREA PACING (RV‐LBBAP STUDY)

### 79.1 **SHUNMUGA SUNDARAM PONNUSAMY** ^1^, VITHIYA GANESAN^1^, VADIVELU RAMALINGAM^1^, PUGAZHENDHI VIJAYARAMAN^2^

#### 79.1.1 ^1^Velammal Medical college, Madurai, India,^2^Geisinger commonwealth school of medicine, Wilkes Barre, PA

**Introduction:** The mechanism of right ventricular(RV) activation during left bundle branch area pacing(LBBAP) is unknown. The aim of this study was to analyze the electrophysiological characteristics of RV activation during LBBAP and its clinical significance.

**Methods:** This prospective, single‐center study included patients with LBBAP between Nov‐2023 to Jun‐2024. The activation of RV during LBBAP could happen through retrograde conduction into the right bundle(RB) or through basal transeptal conduction(TC). Mapping of the RV was performed to categorize the activation pattern. Different patterns of activation among different baseline QRS documented.

**Results:** A total of 113 patients met inclusion criteria. 23 patients excluded due to uninterpretable electrograms and the remaining 90 included in the study. LBBP in 83 patients and LVSP in 7 patients. Mean age 60.6±13.6 years. Non‐selective to selective transition was noted in 82%(n=74). During Selective capture(n=74), RV was activated through RB in 95.9%(n=71) and through TC in 4.1%(n=3). During non‐selective capture(n=83), right bundle conduction in 86.7%(n=72) and transeptal conduction in 13.3%(n=11). During LVSP, RV was activated through transeptal conduction in all(n=7). Among 85 included patients with programmed pacing output of >2V, 80%(n=72) had RV activation through right bundle(group‐I) and 20%(n=18) had activation through TC(group‐II). Patients in group‐II had prolonged baseline QRS duration(140±25 vs 123±29ms;p=0.03) and worse NYHA functional class(2.5±0.7 vs 2.3±0.4;p=0.05). The clinical outcomes of RB mediated RV activation vs TC mediated RV activation will be presented. Different activation pattern in different baseline QRS complex will be presented

**Conclusions:** Retrograde activation of RB is the predominant mechanism (86.7%) of RV activation during non‐selective LBB capture. Patients with TC mediated RV activation had advanced conduction system disease while retrograde RB conduction is associated with early stage of conduction system disease and better clinical parameters

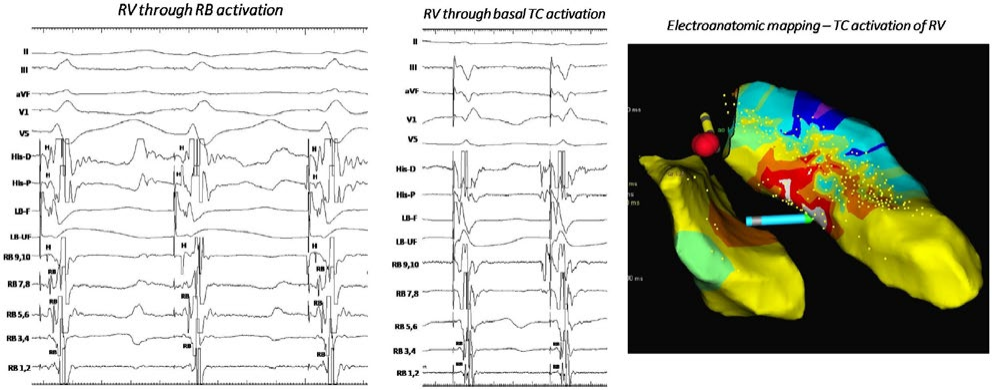

## 80 ETHANOL INFUSION INTO THE VEIN OF MARSHALL REDUCED ATRIAL TACHYARRHYTHMIA RECURRENCE DURING CATHETER ABLATION: A SYSTEMATIC REVIEW AND META‐ANALYSIS

### 80.1 **RAYMOND PRANATA**, WILLIAM KAMARULLAH, GIKY KARWIKY, CHAERUL ACHMAD, MOHAMMAD IQBAL

#### 80.1.1 Department of Cardiology and Vascular Medicine, Faculty of Medicine, Universitas Padjadjaran, Hasan Sadikin General Hospital, Bandung, Indonesia

**Introduction:** Ethanol infusion into the vein of Marshall (EIVoM) may increase mitral isthmus bidirectional block (MIBB) and cause local autonomic denervation that may improve outcome. This meta‐analysis aimed to investigate whether the addition of EIVoM to atrial fibrillation (AF) ablation led to a better outcome.

**Methods:** Systematic literature search was performed using PubMed, SCOPUS, Sciencedirect, and EuropePMC for studies that compared the addition of EIVoM during AF ablation with radiofrequency ablation alone. The primary outcome was atrial tachyarrhythmia (ATa) recurrence, defined as atrial fibrillation/atrial flutter/atrial tachycardia (AF/AFL/AT) after blanking period.

**Results:** There were 2821 patients from 11 studies, and EIVoM was successful in 77% (62‐92%). ATa recurrence was 27% (95%CI 20‐34%) in the EIVoM group and 42% (95%CI 33‐51%) in ablation‐only group. EIVoM reduced ATa recurrence (OR 0.52 [95%CI 0.36, 0.76], p<0.001; I^2^: 76.92). The rate of MIBB was 85% (95%CI 77‐94%) in the EIVoM group and 73% (95%CI 61‐85%) in the ablation‐only group, which was significantly higher (OR 3.87 [95%CI 1.46, 10.28], p<0.001; I^2^: 83.68). Mitral isthmus reconnection rate (OR 0.45 [95%CI 0.15, 1.30], p=0.13; I^2^: 63.4) and redo procedure (OR 0.75 [95%CI 0.53, 1.08), p=0.12; I^2^: 48) were similar, however, a leave‐one‐out sensitivity analysis showed p<0.05 for both. The benefit of EIVoM was not affected by age, LA diameter, and LVEF (p>0.05). Age (p=0.029) and LA diameter (p=0.042) were inversely associated with EIVoM benefits in terms of repeat ablation and mitral isthmus reconnection (age, p=0.003).

**Conclusions:** The addition of EIVoM to ablation increased MIBB and reduced ATa recurrence.

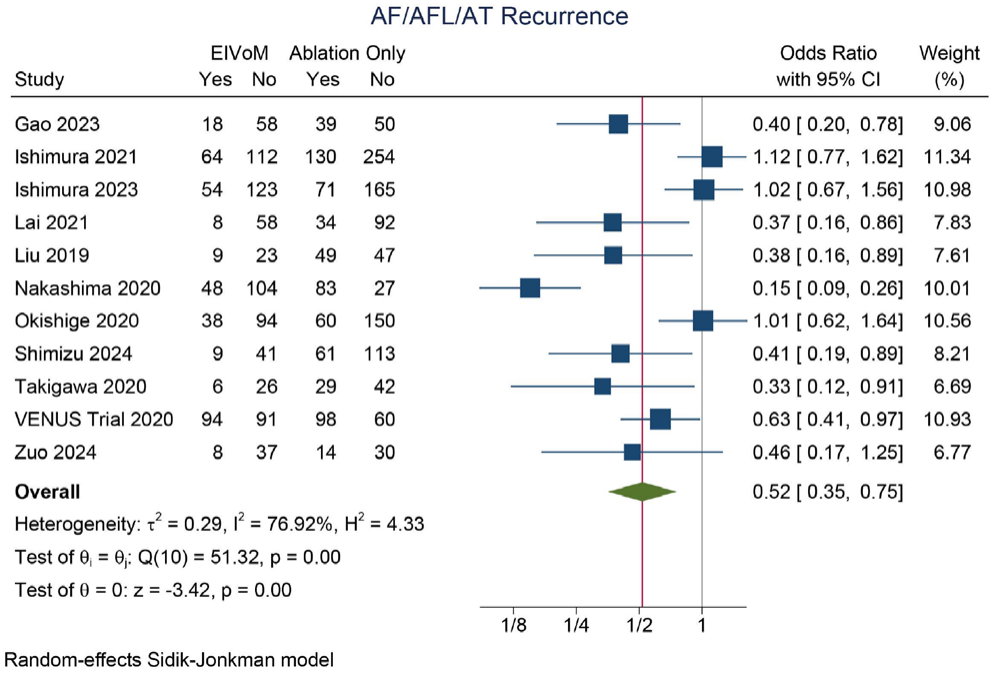

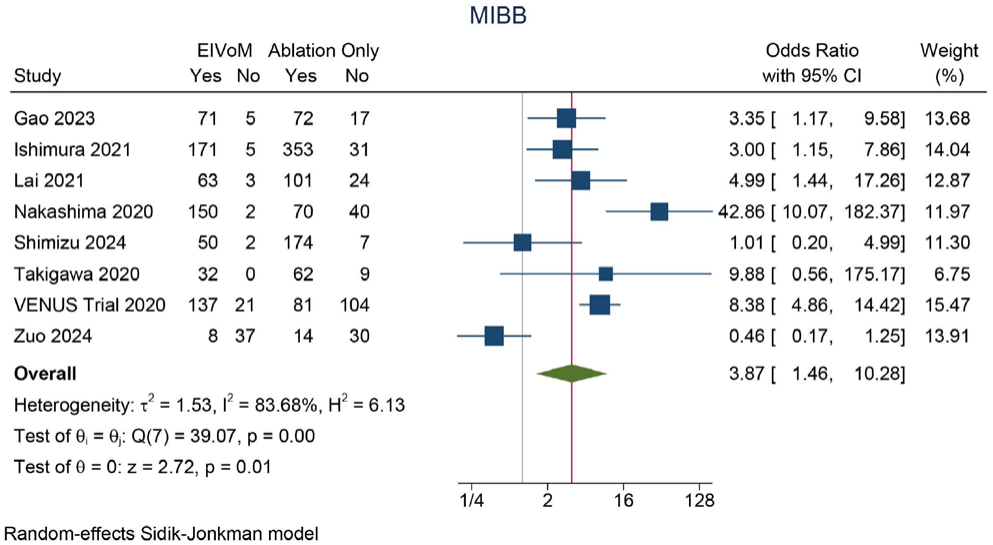

## 81 PULMONARY VEIN ISOLATION DURABILITY WITH A CONFORMABLE SINGLE‐SHOT PULSED FIELD ABLATION CATHETER

### 81.1 **VIVEK REDDY** ^1^, ELAD ANTER^2^, PETR PEICHL^3^, GEDIMINAS RACKAUSKAS^4^, JAN PETRU^5^, MORITOSHI FUNASAKO^5^, JACOB KORUTH^6^, GERMANAS MARINSKIS^4^, MOHIT TURAGAM^6^, AUDRIUS AIDIETIS^4^, JADA SELMA^7^, VOJTECH NEJEDLO^7^, FRED KUEFFER^7^, JOSEF KAUTZNER^3^, ANDREA NATALE^8^, ANDREAS METZNER^9^, PIERRE JAIS^10^, PETR NEUZIL^5^

#### 81.1.1 ^1^Mount Sinai Fuster Heart Hospital, New York, NY,^2^Shamir Medical Center, Tel Aviv, Israel,^3^Institute for Clinical and Experimental Medicine, Prague, Czech Republic,^4^Vilnius University, Vilnius, Lithuania,^5^Homolka Hospital, Prague, Czech Republic,^6^Icahn School of Medicine, Mount Sinai, NY,^7^Medtronic, Inc., Minneapolis, MN,^8^Texas Cardiac Arrhythmia Institute, Austin, TX,^9^University Heart and Vascular Center Hamburg, Hamburg, Germany,^10^University of Bordeaux, Bordeaux, France

**Introduction:** Despite recent advances in catheter ablation technology with pulsed field ablation (PFA), conduction gaps remain a challenge affecting pulmonary vein (PV) isolation (PVI) durability. PVI durability is the main factor in preventing arrythmia recurrence and therefore remap data serves as reasonable surrogate for long‐term efficacy.

**Methods:** In a first‐in‐human study (NCT06026345; NCT05120193), we evaluated the efficiency, safety, and PVI durability of an 8 Fr, single‐shot PFA, all‐in‐one mapping and ablation, conformable catheter (Sphere‐360; Medtronic Inc.) for the treatment of paroxysmal atrial fibrillation (PAF). To depict PV anatomy (Figure 1) in conjunction with the mapping system (Prism‐1; Medtronic Inc.), the over‐the‐wire PFA catheter was maneuvered through an 8.5 Fr steerable sheath into the left atrium. PVI was attained by positioning the catheter into each PV ostium and delivering biphasic pulse trains (2‐4 applications/PV) while moving antral between each application. Based on durability results, the pulse waveform was modified over the trial, resulting in PULSE1, PULSE2, and finally the optimized PULSE3. Optional invasive remapping was performed 75±15 days post ablation. At the time of this interim analysis, 97% of patients had reached 75‐day follow‐up, with 74% completing remap.

**Results:** A total of 100 PAF patients, 58.4±10.6 years old and 60% male, underwent PVI with either PULSE1 (n=30), PULSE2 (n=20) or PULSE3 (n=50) at 3 European centers. Following the simplified workflow, total procedure time was under 1 hour (57.9±20.6 minutes), with minimal fluoroscopy (6.8±5.7 minutes) and using 1 transeptal puncture. There were 0 primary safety events. PVI durability was 78% and 93% on a per‐patient basis and 91% and 99% on a per‐vein basis for the total and PULSE3 cohorts, respectively. Durability remained high even in more difficult PV anatomy (80% in LCPVs (n=10) and 100% in RMPVs (n=6)). Of the 26/292 PVs found to be reconnected in the entire cohort, only 3 (all LSPVs) were in patients treated with the optimized PULSE3 waveform.

**Conclusions:** Treatment of PAF with the conformable single‐shot PFA catheter led to high PVI durability.

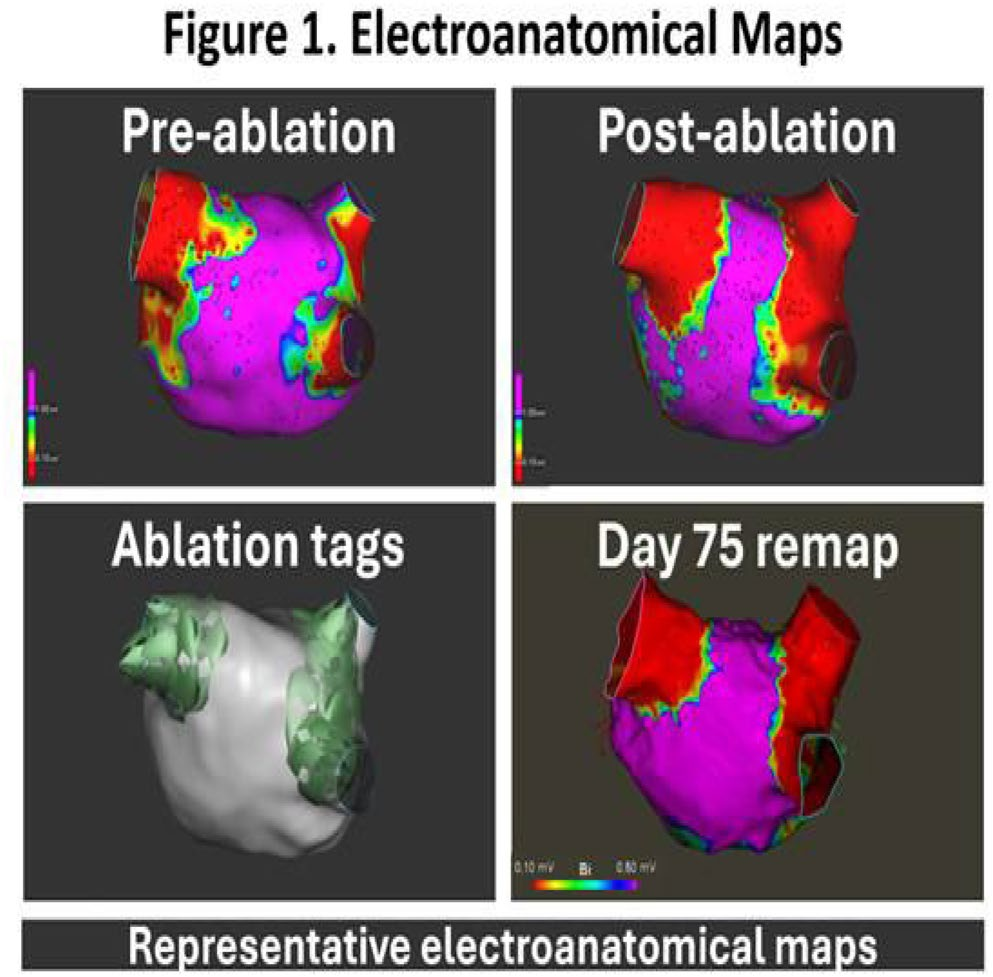

## 82 PULSED FIELD VS RADIOFREQUENCY VS CRYOBALLOON ABLATION FOR ATRIAL FIBRILLATION: A COMPARISON OF POST‐PROCEDURAL TOLERANCE AND SYMPTOMS

### 82.1 **KEENAN SALEH** ^1^, ZAKI AKHTAR^2^, YASEEN MUKADAM^1^, AHMED ABDI^1^, RUI SHI^1^, JAMES BILHAM^1^, WAJID HUSSAIN^1^, DAVID JONES^1^, HASEEB VALLI^1^, SHOUVIK HALDAR^1^, MARK GALLAGHER^2^, ZHONG CHEN^1^, TOM WONG^1^

#### 82.1.1 ^1^Royal Brompton and Harefield Hospitals, Guys' and St Thomas' NHS Trust, London, United Kingdom,^2^St George's University Hospitals NHS Trust, London, United Kingdom

**Introduction:** Pulsed field ablation (PFA) is a novel non‐thermal ablation treatment for atrial fibrillation (AF). In addition to the emerging safety and efficacy data from early adopters of PFA for AF ablation, there is a growing perception that PFA is better tolerated by patients than established ablation modalities. We sought to compare procedure‐related patient symptoms following PFA, radiofrequency (RF) and cryoballoon ablation.

**Methods:** We prospectively enrolled 120 patients (age: 65±10: female 32%) with paroxysmal or early persistent AF from 3 institutions who underwent pulmonary vein isolation (PVI) using PFA, RF or cryoballoon ablation between November 2022 and October 2023, with 40 age‐ and sex‐matched patients per group. Patient symptom scores were collected at 24‐hours and 7‐days post ablation for chest pain, breathlessness, palpitations, sore throat, groin pain and fatigue, each graded using a 5‐point Likert scale. Statistical analysis was performing using Kruskal‐Wallis test and thereafter the Dunn‐Bonferroni post‐hoc test.

**Results:** Patients reported a greater degree of procedure‐related symptoms in the cryoballoom cohort at 24 hours post‐procedure, which mostly improved by day 7 (Figure 1). The median symptom scores were significantly higher (p&lt;0.05) in the cryoballoon cohort than in the PFA and RF cohort for chest pain, breathlessness, palpitations, sore throat and groin pain. There was no significant difference between PFA and RF patients across the symptom domains.

**Conclusions:** Pulmonary vein isolation using PFA or RF is associated with better patient tolerance and fewer post‐procedure symptoms when compared to cryoballoon ablation.

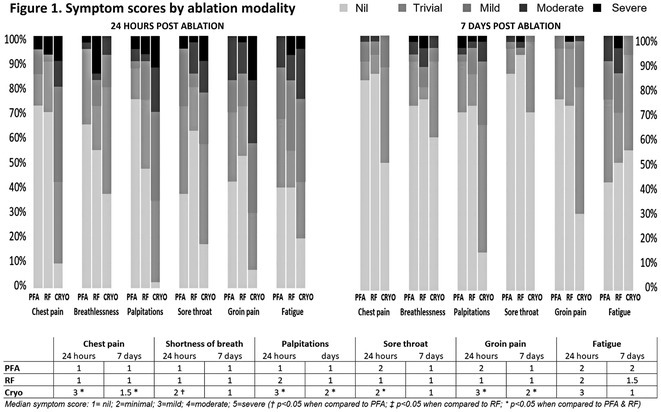

HIGH MODELED 10 YEAR CONDUCTOR RELIABILITY PROJECTED FOR THE NOVEL SMALL‐DIAMETER OMNIASECURE DEFIBRILLATION LEAD

**PRASHANTHAN SANDERS** ^1^, PAMELA MASON^2^, BERT HANSKY^3^, PAOLO DE FILIPPO^4^, MAULLY SHAH^5^, FRANCOIS PHILIPPON^6^, DARIUS SHOLEVAR^7^, TRAVIS RICHARDSON^8^, MICHAEL WEST^9^, BRIAN RAMZA^10^, JAY DINERMAN^11^, ADAM HIMES^12^, JAMES DAWSON^12^, LEAH SEVERSEIKE^12^, AMY THOMPSON^12^, GEORGE CROSSLEY^8^;

^1^University of Adelaide and Royal Adelaide Hospital, Adelaide, Australia,^2^University of Virginia Medical Center, Charlottesville, VA,^3^Städtische Kliniken, Bielefeld, Germany,^4^ASST Papa Giovanni XXIII, Bergamo, Italy,^5^The Children's Hospital of Philadelphia, Philadelphia, PA,^6^Institut Universitaire de Cardiologie et de Pneumologie de Québec, Quebec, QC, Canada,^7^Virtua Cardiology Group, Cherry Hill, NJ,^8^Vanderbilt University Medical Center, Nashville, TN,^9^Presbyterian Heart Group, Albuquerque, NM,^10^Saint Luke's Mid America Heart Institute, Kansas City, MO,^11^Heart Center Research, Huntsville, AL,^12^Medtronic, Inc., Minneapolis, MN.

**Introduction:** Defibrillation leads remain the weak link in ICDs, thus reliable leads are needed. The novel, lumenless, 4.7Fr, catheter‐delivered, OmniaSecure defibrillation lead was designed for reliability and targeted placement, based on the established SelectSecure SureScan MRI Model 3830 lumenless pacing lead that has been commercialized since 2003. The LEADR trial reported efficacy & safety of the OmniaSecure lead through 12 mo. with follow‐up ongoing. Alongside the trial, advanced conductor fatigue reliability modeling has been used to understand long‐term lead durability. This reliability model accurately predicted the 10 year observed durability of Sprint Quattro and Fidelis defibrillation leads.

**Methods:** Fracture‐free survival of the OmniaSecure lead was projected using a validated reliability model incorporating patient and bench data. Patient data were obtained from biplane fluoroscopy images of the lead during cardiac and patient motion to evaluate lead bending curvature in a subset of LEADR trial patients (n=53). Bench tests then reproduced these use conditions utilizing ~4x greater bending curvatures than observed in patients to exaggerate stress on the lead.

**Results:** As previously reported, the worldwide LEADR trial passed its endpoints demonstrating 97.5% overall implant defibrillation efficacy and 97.1% freedom from lead‐related major complications at 12 mo. There have been zero lead fractures of the OmniaSecure lead through 12.7 ± 4.8 mo. Advanced reliability modeling projected a 99.94% fracture‐free survival rate at 12 mo., aligned with the zero fractures observation within the trial. This model has been extended to 10 years and has evaluated both adult (≥22 yrs) and adolescent (≥ 12 & <22 yrs) populations. The projected 10‐year fracture‐free survival rate of the OmniaSecure lead is 98.27% (adult) and 97.91% (adolescent) (Figure).

**Conclusions:** Consistent with early clinical trial experience, modeling projects highly reliable 10‐year performance of the OmniaSecure lead including within the active adolescent population which may benefit from a novel 4.7 Fr defibrillation lead designed for reliability.

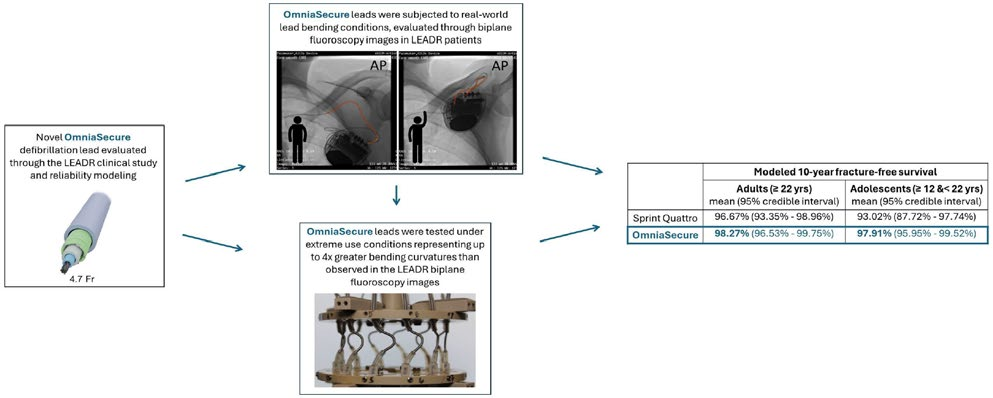

Chair

**U. Schotten**

Maastricht University Medical Centre, Maastricht, Netherlands

## 83 PROCEDURAL AND LONG‐TERM CLINICAL OUTCOMES FOLLOWING LEFT ATRIAL PULSED FIELD ABLATION WITH ZERO FLUOROSCOPY FOR THE TREATMENT OF ATRIAL FIBRILLATION

### 83.1 **WILLIAM H. SAUER** ^1^, DAVID NEWTON^2^, DEVI NAIR^3^, CHRISTOPHER F. LIU^4^, MARK METZL^5^, ANSHUL M. PATEL^6^, LUIGI DI BIASE^7^, PAUL C. ZEI^1^, JOSE OSORIO^8^, MOUSSA MANSOUR^9^, HUGH CALKINS^10^, OUSSAMA WAZNI^11^, VIVEK Y. REDDY^12^, ANDREA NATALE^13,14^

#### 83.1.1 ^1^Cardiac Arrhythmia Service, Brigham and Women's Hospital and Harvard Medical School, Boston, MA,^2^Memorial Health, Savannah, GA,^3^St. Bernards Medical Center & Arrhythmia Research Group, Jonesboro, AR,^4^Weill Cornell Medicine – New York Presbyterian Hospital, New York, NY,^5^Endeavor Health, Glenview, IL,^6^Emory Arrhythmia Center, Emory St. Joseph's Hospital, Atlanta, GA,^7^Cardiac Arrhythmia Center, Division of Cardiology at the Montefiore Medical Center, Albert Einstein College of Medicine, New York, NY,^8^HCA Florida Miami, Miami, FL,^9^Massachusetts General Hospital, Boston, MA,^10^Johns Hopkins Medical Institutions, Baltimore, MD,^11^Cleveland Clinic Foundation, Cleveland, OH,^12^Helmsley Electrophysiology Center, Mount Sinai Fuster Heart Hospital, New York, NY,^13^Texas Cardiac Arrhythmia Research Foundation, Austin, TX,^14^Department of Biomedicine and Prevention, Division of Cardiology, University of Tor Vergata, Rome, Italy

**Introduction:** Zero‐fluoroscopy (ZF) techniques for mapping and ablation have been shown to be safe and effective in pulmonary vein isolation (PVI) procedures using radiofrequency or cryothermal energies. However, limited data are available on use of ZF techniques in pulsed‐field ablation (PFA) procedures. In the admIRE study, which demonstrated the safety and efficacy of a novel variable‐loop circular catheter (VLCC) integrated with an electroanatomical mapping (EAM) system for PFA of atrial fibrillation (AF), several operators used a ZF approach. This subgroup analysis of admIRE evaluated outcomes of PFA using a ZF approach versus low fluoroscopy (LF) and conventional fluoroscopy (CF).

**Methods:** The prospective, multicenter, single‐arm admIRE study enrolled adult subjects with symptomatic drug‐refractory paroxysmal AF (PAF) in the US from April‐November 2022. PVI was achieved using the VLCC with integrated EAM system. For this retrospective analysis, subject and procedural characteristics and clinical outcomes were compared in subgroups based on fluoroscopy time: ZF, LF (<5 min), or CF (>5 min). Significant differences in distribution of subject characteristics and outcome data were tested by Fisher's exact test or Kruskal‐Wallis test.

**Results:** A total of 87 subjects from 7 sites were included in the ZF subgroup, whereas 65 and 210 subjects were identified in the LF and CF subgroups. Baseline characteristics were comparable across subgroups; average age was ~62 years, and most subjects (61‐64%) were male. Mean number of PFA applications and PFA time were lower in the ZF and LF subgroups, with a higher first‐pass isolation rate, compared to the CF subgroup (**Table 1**). Despite these differences, the proportion of subjects with freedom from AF recurrence at 12 months was comparable with ZF (72%), LF (75%), and CF (74%; **Figure 1**). Primary adverse event rates were non‐significantly lower with ZF (1.2%) and LF (1.5%) versus CF (2.9%).

**Conclusions:** In this subanalysis of the admIRE study, the use of ZF and LF catheter ablation techniques was associated with comparable safety and efficacy to CF.

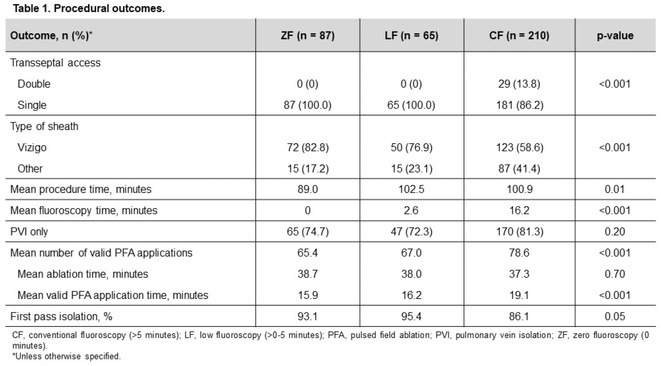

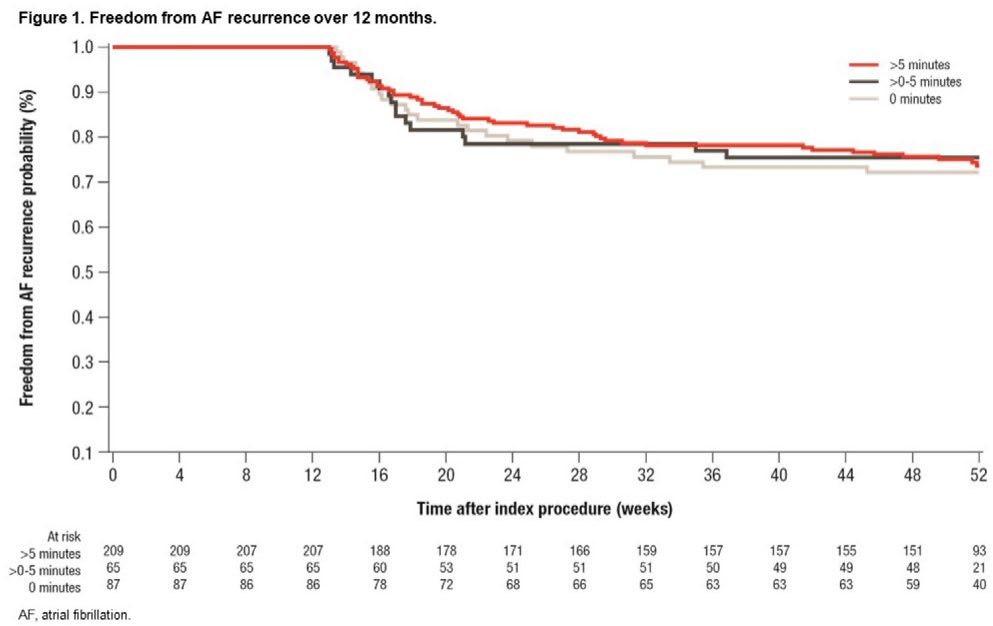

## 84 LEFT ATRIAL STASIS PERSISTS DESPITE LV RECOVERY FOLLOWING CATHETER ABLATION IN AF‐MEDIATED CARDIOMYOPATHY

### 84.1 **LOUISE SEGAN** ^1,2^, JOHN KEARNS^3^, STAVROULA PAPAPOSTOLOU^1^, ROSE CROWLEY^1^, JEREMY WILLIAM^1^, BENEDICT COSTELLO^2^, DAVID CHIENG^1^, ALEKSANDR VOSKOBOINIK^1^, LIANG‐HAN LING^1^, HARIHARAN SUGUMAR^1^, GEOFF LEE^4^, JOSEPH MORTON^4^, ANDREW TAYLOR^1,4^, JONATHAN KALMAN^4^, PETER KISTLER^1^, SANDEEP PRABHU^1^

#### 84.1.1 ^1^The Alfred Hospital, Melbourne, Australia,^2^Baker Heart and Diabetes Institute, Melbourne, Australia,^3^Monash University, Melbourne, Australia,^4^The Royal Melbourne Hospital, Melbourne, Australia

**Introduction:** LV recovery is frequently observed following sinus rhythm restoration in AF‐mediated cardiomyopathy(AFCM). However, the extent and reversibility of left atrial (LA) remodeling is uncertain. We examined left atrial flow haemodynamics in patients with recovered AFCM compared to healthy controls.

**Methods:** We compared LA size (left atrial volume index (LAVI)) and flow haemodynamics between healthy controls and recovered AFCM patients with sinus rhythm maintained >6 months. LA stasis was determined by tracking virtual particles flowing with the 4D‐flow velocity field to assess transit time through the LA with a Residence Time Distribution (RTD) and quantified stasis from the time constant (RTDtc) from the distribution's decay.

**Results:** Individuals with recovered AFCM(N=20) were age‐matched with controls (N=20; mean age 61.3±7.5 vs 59.3±8.0 years in controls,p=0.392) and underwent CMR imaging with 4D‐flow analysis. Markers of LA remodeling and stasis were evaluated (LAVI and RTDtc, respectively). LA RTDtc was prolonged in individuals with recovered AFCM compared to controls (1.4±0.4 vs 1.2±0.2, p=0.016, figure 1a), as was LA size (LAVI 28.2±11.3 vs 40.6±13.0ml/m^2^,p=0.002). RTDtc was strongly correlated with LA size in both groups (R=0.409,p=0.0006, figure 1b). In those with recovered AFCM (mean LVEF 58.2±5.5%, LAVI 40.6±13.0mml/m^2^, CHADS2VASc 2.0 (IQR 1.0‐3.0)), LA enlargement was incrementally associated with greater RTDtc (R = 0.470, p=0.018) and arrhythmia recurrence (R=0.417, p=0.038).

**Conclusions:** Individuals with AFCM exhibit persisting left atrial stasis and enlargement despite LV recovery following sinus rhythm restoration. This may reflect incomplete reversibility of LA remodeling which may have important prognostic implications including the need for long‐term anticoagulation among the AFCM population.

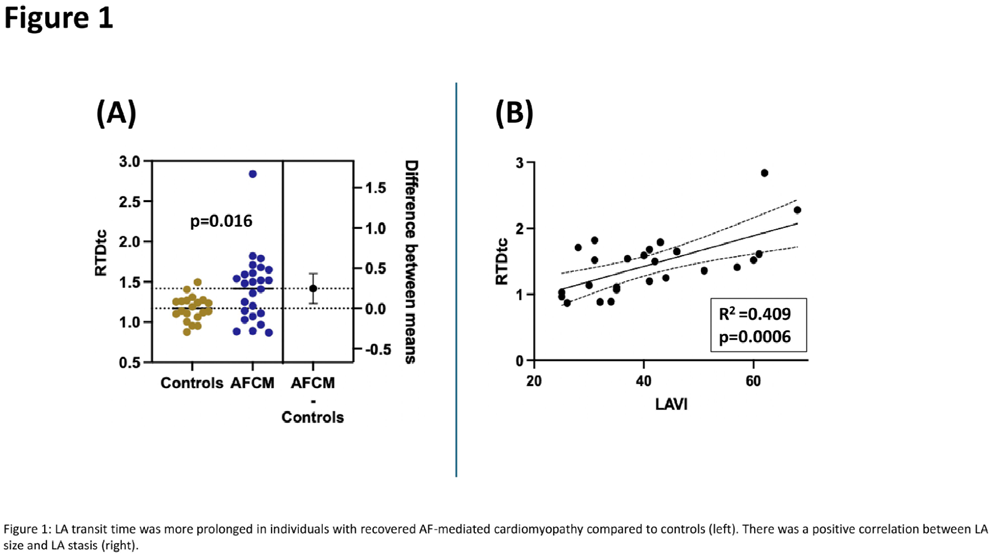

## 85 RELATIONSHIP BETWEEN TITIN AND LIFESTYLE RISK FACTORS ON INCIDENT AF RISK AMONG THE UK BIOBANK

### 85.1 **LOUISE SEGAN** ^1^, WILLIAM HO^2^, SHANE NANAYAKKARA^1^, ROSE CROWLEY^1^, JEREMY WILLIAM^1^, MICHAEL LIM^3^, SANDEEP PRABHU^1^, JONATHAN KALMAN^3^, DOMINIC ABRAMS^4^, FUMIHIKO TAKEUCHI^2^, PETER KISTLER^1^

#### 85.1.1 ^1^The Alfred Hospital, Melbourne, Australia,^2^Baker Heart and Diabetes Institute, Melbourne, Australia,^3^Royal Melbourne Hospital, Melbourne, Australia,^4^Boston Children's Hospital, Boston, MA

**Introduction:** Titin truncating variants (TTNtv) have been associated with heightened arrhythmogenic risk among the dilated cardiomyopathy population. However, it is unclear whether TTNtv could enhance risk stratification in evaluating incident AF risk. We evaluated the interaction between TTNtv and lifestyle AF risk factors on incident AF risk among the UK Biobank.

**Methods:** TTNtv was examined in those with and without incident AF based on ICD‐10 coding (baseline AF was an exclusion). Univariate and multivariable regression examined the association between TTNtv, components of the previously validated HARMS_2_‐AF lifestyle AF risk score, and incident AF risk. We then compared TTNtv prevalence stratified by low (score <5) and high (score >5) HARMS_2_‐AF clinical risk categories.

**Results:** Among 301,865 participants with available whole genome sequencing data (48.0% male, age 57 years (IQR 50‐63), 84.8% caucasian), AF incidence was 6.5% with a median time to AF 8.5 (IQR 5.0‐11.2) years. Prevalence of TTNtv was 0.2% overall and higher among the incident AF group (0.4% vs 0.1%). The AF population were older (63.0 (IQR 59.0‐66.0) vs 57.0 (IQR 49.0‐62.0), with a greater prevalence of hypertension (68.1% vs 26.1%), diabetes (9.3% vs 4.0%), sleep apnea (5.6% vs 1.6%) and obesity (32.7% vs 20.6%). TTNtv was the strongest independent predictor of incident AF risk, conferring a 4‐fold higher risk independent of modifiable AF risk factors (OR 4.15, 95% CI 2.97‐5.50, P<0.001; figure 1a) and was consistent across both sexes (figure 1b). TTNtv was more strongly associated with incident AF risk than AF polygenic risk score (AF PRS: OR 1.67, 95% CI 1.62‐1.74). The TTNtv gene positive individuals who developed AF had a lower median HARMS2‐AF risk score (median 6.0 (IQR 4.2‐8.8) compared to gene negative AF individuals (HARMS2‐AF score 8.0 (IQR 5.0‐10.0).

**Conclusions:** TTNtv status was strongly associated with incident AF risk irrespective of lifestyle risk factors and identified an AF subset with minimal AF risk factors. Further studies incorporating clinical and genetic risk factors are needed to enhance AF risk prediction.

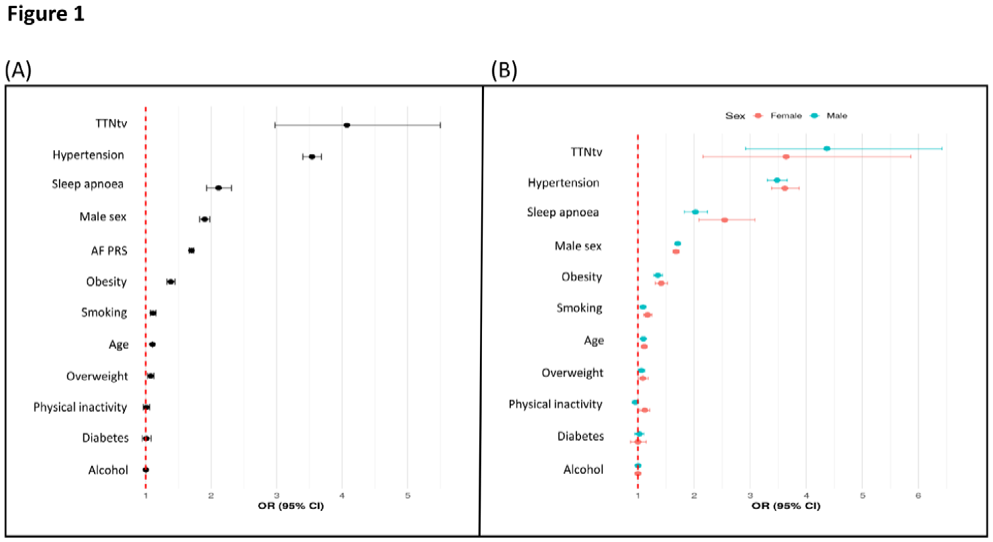

## 86 ROLE OF SEX DIFFERENCES WITH HOME‐BASED STRUCTURED EDUCATION AND LEARNING PROGRAM FOR ATRIAL FIBRILLATION ON HOSPITALISATION AND MORTALITY, HELP‐AF TRIAL

### 86.1 **ELNAZ SHAHMOHAMADI** ^1^, JEROEN M. HENDRIKS^2^, DEBRA ROWETT^3^, PRASHANTHAN SANDERS^1^, MELISSA E. MIDDELDORP^1^

#### 86.1.1 ^1^Centre for Heart Rhythm Disorders, University of Adelaide, Adelaide, Australia,^2^Caring Futures Institute, College of Nursing and Health Sciences, Flinders University, Adelaide, Australia,^3^School of Pharmacy and Medical Sciences, University of South Australia, Adelaide, Australia

**Introduction:** The impact of education on reducing hospitalizations among patients with atrial fibrillation (AF) was promising. It is of interest to see whether this effect is modified by patient sex.

**Methods:** The Home‐based patient‐centered structured Education and Learning Program (HELP‐AF) randomized patients presenting to the emergency department due to atrial fibrillation (AF) into two groups: usual care or the HELP‐AF intervention. The HELP‐AF intervention included two in‐home educational visits based on the principles of structured education. The primary outcomes of this analysis were hospitalization details and all‐cause mortality

**Results:** The study randomized 627 patients, to usual care (n=313) and HELP‐AF (n= 314), 277 (42%) females. Compared with males, females were older (71.9 ± 13.2 years vs. 66.3±13.2 years), were less educated (17% tertiary education vs. 28.6 %) and less likely to live alone (37.5% vs. 18.6%). Based on results of univariate Poisson regression, females contributed to a significant higher rate of total unplanned hospitalizations (Incidence Rate Ratio [IRR] 1.3, 95% CI: 1.0‐1.7; p=0.02), with no observed difference in unplanned AF‐related hospitalizations and cardiac‐related hospitalizations. No evidence of an interaction between the treatment group and sex in the multivariate Poisson regression model was observed (p=0.526). The proportion of patients with more than one hospitalization was higher in females compared to males (p = 0.03). Additionally, total unplanned hospitalization days were significantly higher in females than in males (p =0.001). Time to first hospital admission was significantly lower in females compared to males (Log‐rank p<0.001). All‐cause mortality occurred in 10 males and 4 females (p=0.23)

**Conclusions:** A home‐based structured education program reduced total unplanned hospitalizations in both females and males, with no modification for sex of the patient. However, females had a higher risk of hospitalization compared to males. There was no mortality difference observed between sexes.

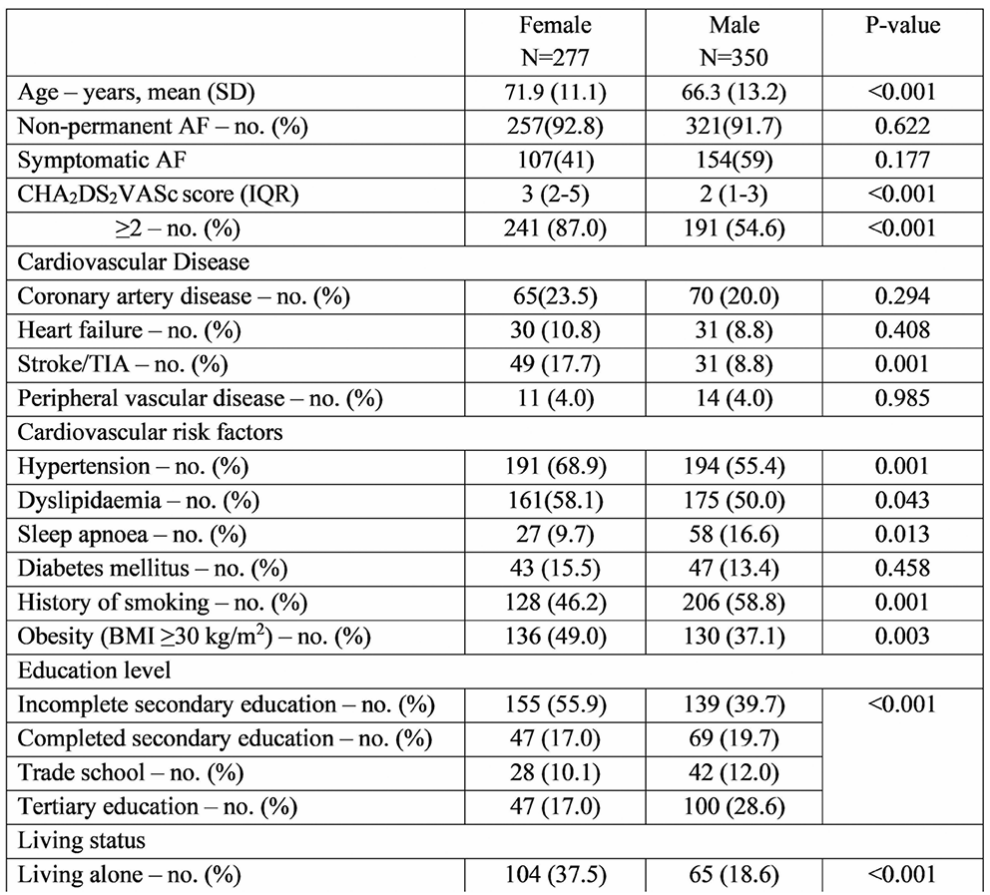

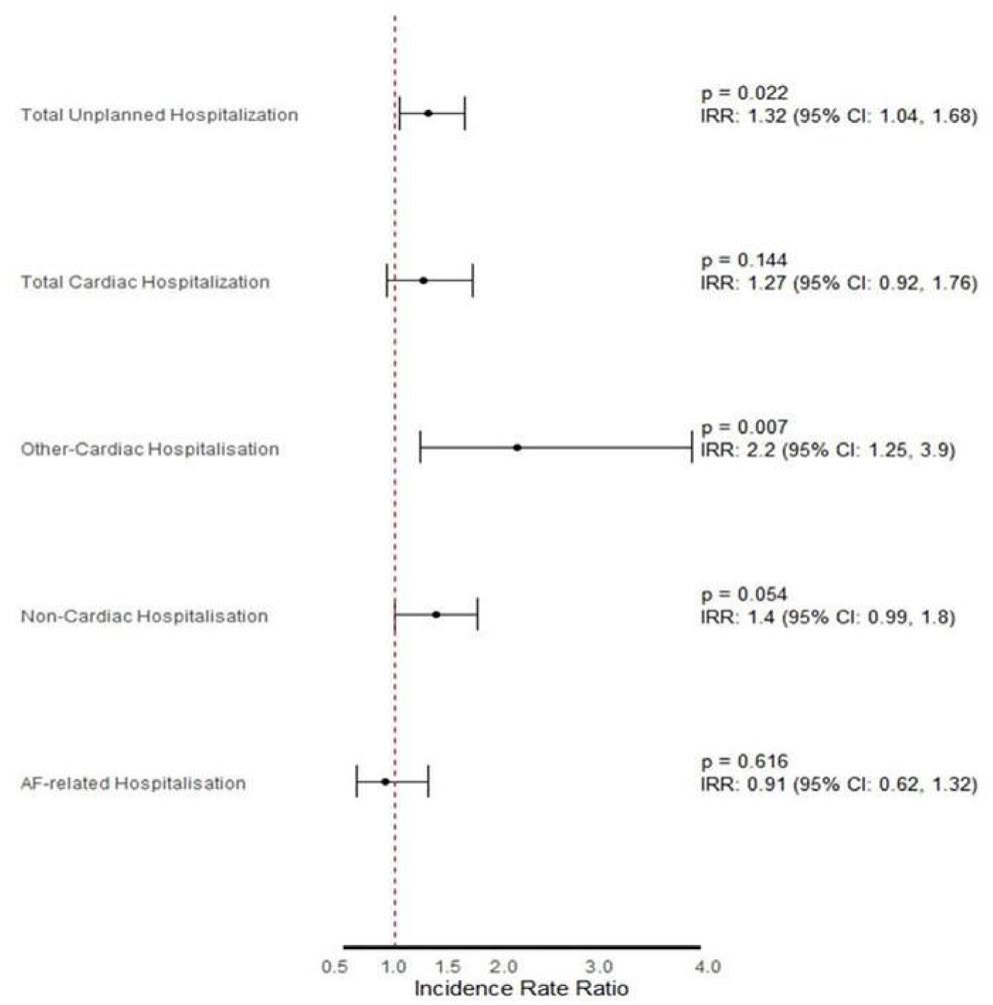

## 87 CHANGES IN EPICARDIAL ADIPOSE TISSUE VOLUME BEFORE AND AFTER CRYOBALLOON ABLATION PROCEDURE FOR PATIENTS WITH PAROXYSMAL AND PERSISTENT ATRIAL FIBRILLATION; SUPPORTING “AF BEGETS EAT” THEORY

### 87.1 **KAZUKI SHIMOJO**, ITSURO MORISHIMA, YASUHIRO MORITA, YASUNORI KANZAKI, NAOKI WATANABE, NAOKI YOSHIOKA, NAOKI SHIBATA, YOSHIHITO ARAO, TAKUMA OHI, HIROKI GOTO, HOSHITO KARASAWA, YUTA NAKAGAWA, YUKI KAWASAKI, TATSUKI YOSHIE

#### 87.1.1 Ogaki Municipal Hospital: Ogaki Shimin Byoin, Ogaki city, Japan

**Introduction:** Recently, it has been considered that epicardial adipose tissue (EAT) is associated with atrial fibrillation (AF). Several studies have shown that EAT volume surrounding the left atrium (LA‐EATV) may have a significant relationship with the increased risk of atrial fibrillation, as well as EATV surrounding entire heart (Total‐EATV). Fundamentally, the mechanism of “EAT begets AF” was demonstrated in multifactorial factors via various cytokines, oxidative stress, and fatty infiltration, which causes atrial remodeling. However, the mechanism of “AF begets EAT” is not elucidated completely. We tried to demonstrate one of atrial reverse remodeling pathways, revealing restoration to sinus rhythm causes atrial reverse remodeling via Total‐ and LA‐EATV reduction.

**Methods:** We analyzed 247 patients who underwent cryoballoon ablation (CBA) for AF with contrast‐enhanced CT (CECT) data in our institute, retrospectively [paroxysmal AF (PAF); 214pts, persistent AF (PEF); 33pts]. We characterized the EATV by CECT, using 3D analysis workstation; SYNAPSE VINCENT (Fuji Photo Film Co., Ltd., Japan), and analyzed both Total‐ and LA‐EATV before and 6 months after CBA.

**Results:** Total‐ and LA‐EATV were significantly larger in patients with PEF than those with PAF at baseline (Total‐EATV; 148.8±53.3 mL vs 123.7±49.8 mL, p=0.02, and LA‐EATV; 26.8±11.3 mL vs 21.7±10.8 mL, p=0.009, respectively). In PAF group, Total‐ and LA‐EATV did not change between before and after procedure, while both Total‐ and LA‐EATV significantly reduced 6 months after procedure in PEF group (Total‐EATV; 148.8±53.3 to 142.9±53.5 mL, p=0.01, and LA‐EATV; 26.8±11.3 to 25.2±10.7 mL, p=0.01, respectively). On multivariate logistic regression analysis, the presence of PEF remained as significant factor for Total‐ and LA‐EATV reduction [OR; 2.36, p=0.03, and OR; 2.43, p=0.05, respectively].

**Conclusions:** Total‐ and LA‐EATV could be reduced after CBA in patients with PEF, but not in patients with PAF. These findings might support “AF begets EAT” theory.

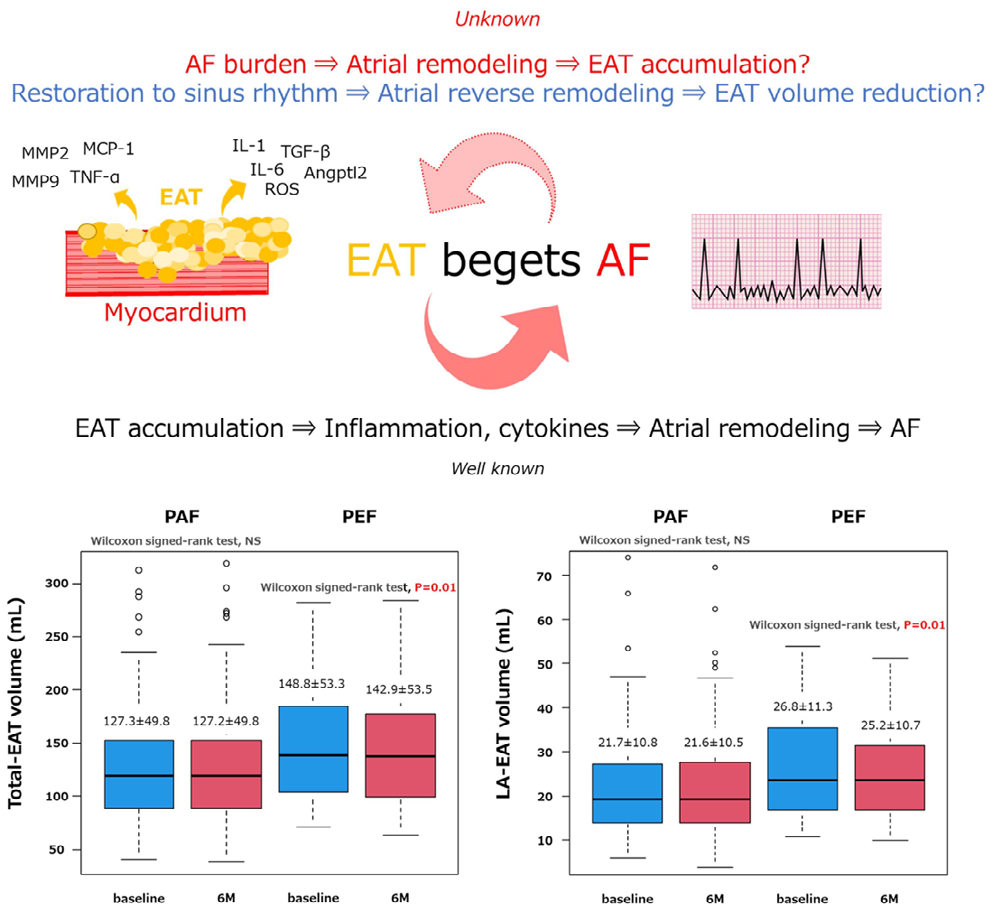

## 88 ONE YEAR SAFETY AND PERFORMANCE OF A DUAL‐CHAMBER LEADLESS PACEMAKER IN AN ASIA‐PACIFIC POPULATION

### 88.1 **MORIO SHODA** ^1^, CHIN PANG CHAN^2^, YAT SUN JOSEPH CHAN^2^, MARK TSZ‐KIN TAM^2^, KENJI ANDO^3^, MICHIO NAGASHIMA^3^, HIROSHI TASAKA^4^, MITSURU YOSHINO^4^, HUNG FAT TSE^5^, LIAN‐YU LIN^6^, DAIGO YAGISHITA^1^, KENGO KUSANO^7^, KOHEI ISHIBASHI^7^, DANIEL F. BOOTH^8^, ANU BULUSU^8^, REINOUD KNOPS^9^

#### 88.1.1 ^1^Tokyo Women's Medical University, Tokyo, Japan,^2^Prince of Wales Hospital, Hong Kong, Hong Kong,^3^Kokura Memorial Hospital, Kitakyushu, Japan,^4^Kurashiki Central Hospital, Kurashiki, Japan,^5^Queen Mary Hospital, Hong Kong, Hong Kong,^6^National Taiwan University Hospital, Taipei, Taiwan,^7^National Cerebral & Cardiovascular Center Hospital, Suita, Japan,^8^Abbott, Sylmar, CA,^9^Amsterdam Academic Medical Centre, Amsterdam, Netherlands

**Introduction:** A dual‐chamber leadless pacemaker (LP) clinical study previously reported the 3‐month safety and performance results for a cohort of 40 patients from an Asia‐Pacific (APAC) region with attempted implants. The study also reported the 1‐year results for the primary cohort (non‐APAC region) of 300 patients. Herein we report the longer term 1‐year outcomes of the APAC cohort compared to the non‐APAC cohort.

**Methods:** In both cohorts, patients with standard dual‐chamber pacing indications were implanted with the dual‐chamber LP system (AVEIR™ DR; Abbott), consisting of atrial and ventricular LPs. One‐year endpoints included proportion of patients with: (i) freedom from device‐ or procedure‐related serious adverse events (SADEs) and (ii) both atrial pacing capture threshold (aPCT) ≤3.0V at 0.4ms and P‐wave amplitude ≥1.0mV. Implant‐to‐implant (i2i™) wireless communication between the 2 LPs enabled true DDD(R) programming. The atrial‐to‐ventricular (A2V) and ventricular‐to‐atrial (V2A) i2i communication success was assessed, which may serve as a lower‐bound of atrioventricular synchrony delivered by the LP system.

**Results:** Forty APAC and 300 non‐APAC patients had attempted implants, with APAC patients having greater age and smaller body size (Table). Seven APAC patients had 11 different SADEs, all within 82 days post‐implant—none occurred after 3 months post‐implant. The Kaplan‐Meier SADE‐free estimate was 82.5% for APAC vs. 88.3% for non‐APAC patients (P=0.29; Figure). Adequate aPCT and P‐wave amplitude were achieved in 90.0% APAC vs. 92.8% non‐APAC patients (P=0.52). From the 6‐month to 1‐year follow‐up, A2V and V2A i2i communication was successful in APAC and non‐APAC patients (A2V: 90.3% vs. 90.3% of beats, P=0.78; and V2A: 88.9% vs. 87.5% of beats, P=0.76).

**Conclusions:** The first dual‐chamber LP system displayed continued safety and performance through 1 year post‐implant in an Asia‐Pacific population, with outcomes comparable to the non‐APAC primary cohort.

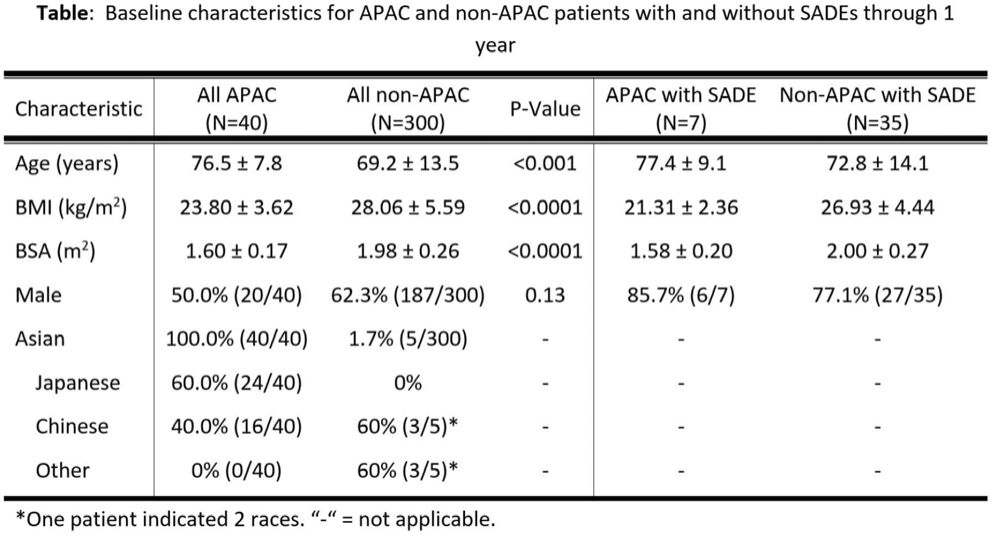

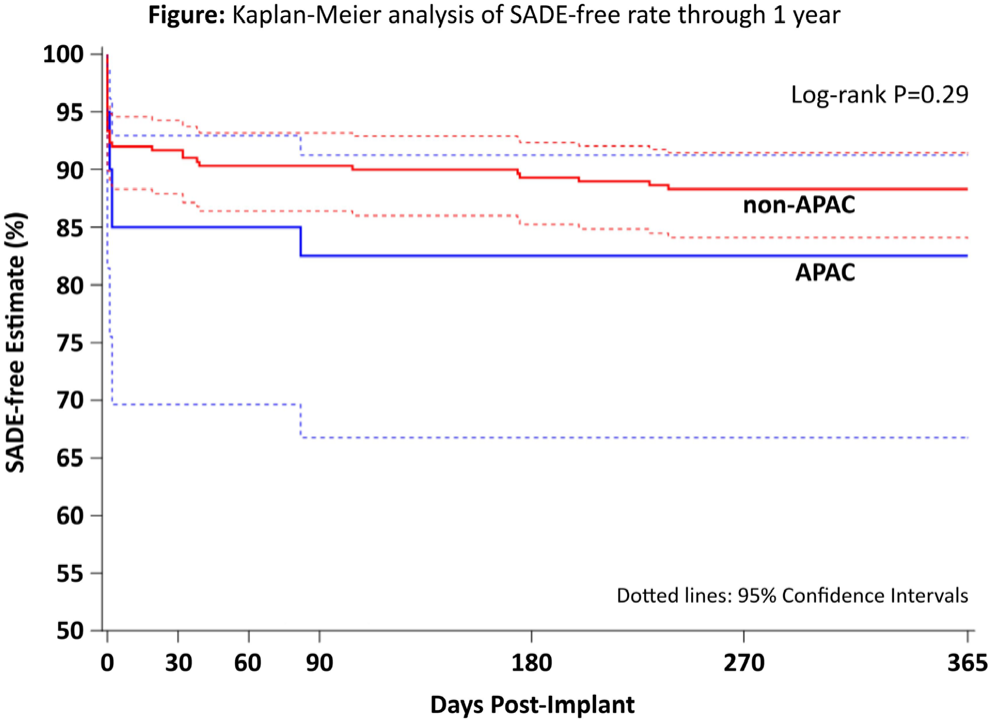

## 89 COMPARISON OF PROCEDURAL OUTCOMES OF LUMENLESS FIXED‐HELIX VERSUS STYLET DRIVEN RETRACTABLE‐HELIX LEAD SYSTEMS IN LEFT BUNDLE BRANCH PACING‐ COMPARE LBBP

### 89.1 **JENISH P SHROFF** ^1^, ANUGRAH NAIR^1^, LUKAH Q. TUAN^1^, WALTER P. ABHAYARATNA^2^, PRASHANTHAN SANDERS^3^, RAJEEV K. PATHAK^1^

#### 89.1.1 ^1^Canberra Heart Rhyhtm, Australian National University, Canberra, Australia,^2^Australian National University, Canberra, Australia,^3^Centre for Heart Rhythm Disorders, University of Adelaide and Royal Adelaide Hospital, Adelaide, Australia

**Introduction:** Left bundle branch pacing (LBBP) has emerged as a safe and effective alternative to right ventricular pacing.Traditionally, LBBP is performed with lumenless lead (LLL); however, the use of stylet driven lead (SDL) is on rise. We aimed to assess acute success and procedural outcomes of LLL vs SDL for LBBP.

**Methods:** 100 consecutive patients were randomized in 1:1 fashion to LLL and SDL arms. Patients with bradyarrhythmia and/or indication of cardiac resynchronization therapy were included. Abbott's Tendril lead with a CPS Locator 3D catheter and Medtronic's SelectSecure 3830 lead with a C315HIS catheter were used in SDL and LLL arms, respectively. LBBP was confirmed by paced right bundle branch block (RBBB), R wave peak time (RWPT) in V6, V6‐V1 interpeak interval and transition during threshold test. All electrocardiographic measurements were done on Labsystem Pro^TM^.

**Results:** Baseline characteristics were not different (P>0.05). Acute success in achieving LBBP was similar in LLL vs SDL arms (92% vs 88%, P=0.6) as implant attempts (2.1±1.3 vs 2.3±2.2, P=0.6), implant duration (9.9±7.1 vs 10±9.8 mins, P=0.3), median fluoroscopy dose (37 vs 29 mGy, P=0.6) and mean fluoroscopy time (7.1 ± 3.9 vs 7.9 ± 4.9 mins, P=0.4). Baseline (116.6 ± 24.6 vs 120.9 ± 35.7 ms, P=0.5) and post‐pacing QRS duration (141.1±14.7 vs 140.4±14 ms, P=0.8), RWPT (78.9±11 vs 79.6±10.4 ms, P=0.7), V6‐V1 interpeak interval (46.8±9.9 vs 47.8±8.7, P=0.6) were not different in LLL vs SDL arms, respectively. Sensed R wave (11.6±5.5 vs 8.2±3 V, P<0.001) and bipolar lead impedance (773±180 vs 640±85 Ω, P<0.001) were greater with LLL; however, pacing threshold was not different (0.65±0.2 vs 0.73±0.3 V, P=0.1). No major complications were recorded in either arm; however, RBBB and septal perforations were more common in SDL arm (P=0.007).

**Conclusions:** LBBP was feasible with both the lead systems with similar success rate, procedural duration and low pacing threshold. Whilst no major complications were recorded with either lead, septal perforations were more common with SDL. (Clinical trial registration: ACTRN12624000304538).

## 90 VENTRICULAR TACHYCARDIA ABLATION IN HEART FAILURE: PREVALENCE OF PAPILLARY MUSCLE VENTRICULAR TACHCARDIA, SUBSTRATE CHARACTERISTICS AND CLINICAL OUTCOMES

### 90.1 **TAI CHUNG SO**, SAURABH KUMAR

#### 90.1.1 Westmead Hospital, Sydney, Australia

**Introduction:** Ventricular tachycardia (VT) is common in heart failure, up to 20% of heart failure patients will experience VT. The interplay between heart failure and ventricular arrhythmias is complex. Despite VT ablation is effective treatment modality for VT, substrate characteristic and outcome of VT ablation in heart failure population have not been well studied. The aims of this study are: 1) to evaluate the substrate characteristics in heart failure patients with VT, in particular the role of papillary muscle; 2) to evaluate the outcomes of catheter ablation and its predictors for VT recurrence and mortality.

**Methods:** This is a retrospective single centre study. Patients with LVEF < 35% and sustained monomorphic VT undergoing VT ablation were included. Procedural characteristics including VT origin and its substrate characteristics were studied. Outcomes included procedural outcome and clinical outcomes. Procedural outcomes included acute procedural success and complication. Clinical outcome included VT recurrence and mortality in 30 days and 1 year. Chi‐square, Mann‐Whitney tests, Kaplan‐Meier method and Cox proportional hazard regression models were used for statistical analysis.

**Results:** There were 48 patients in the study. The main important findings are: (1) 33% of the patient has VT exit in LV papillary muscle, and mixed cardiomyopathy is more likely to have LV papillary muscle VT exit site; (2) Presence of hyerechogencity of LV papillary muscle on ICE is common and is found in 68% of patient, but its presence does not predict VT exit site at LV papillary muscle; (3) VT recurrence and mortality in 1 year in 41% and 4% respectively. Presence of VT exit in LV papillary muscle is associated with higher rate of VT recurrence (log rank p = 0.031), but not mortality in 1 year (log rank p = 0.071).

**Conclusions:** VT recurrence rate and mortality is high in heart failure population with VT. Understanding the VT substrate characteristics helps improving outcome in VT ablation in the heart failure population, in particular considering papillary muscle as VT exit and ablation target.

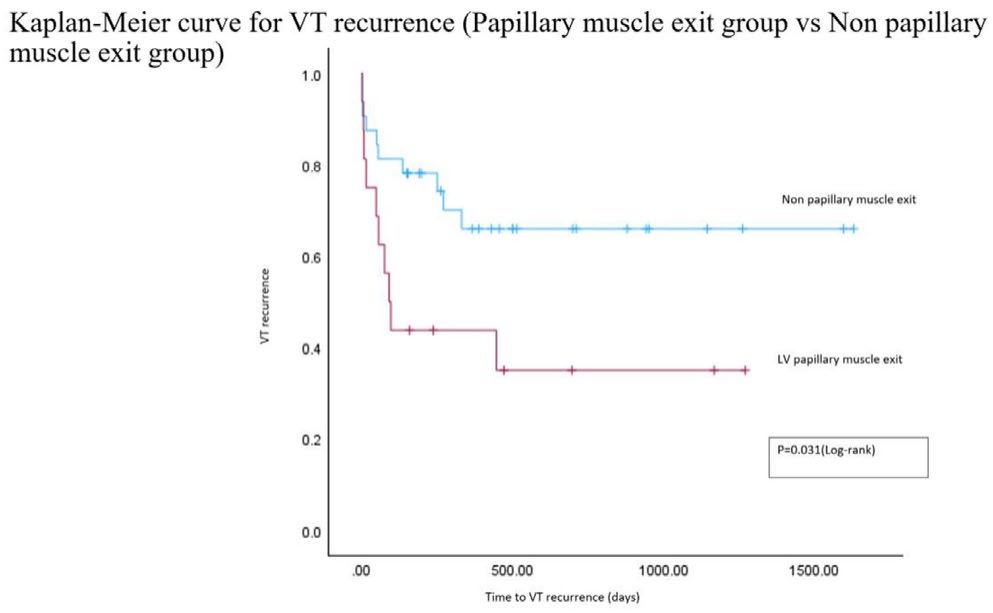

## 91 CLINICAL CHARACTERISTICS AND OUTCOMES OF PEDIATRIC PATIENTS WITH TYPE 1, 2, & 3 LONG QT SYNDROME: A KOREAN MULTICENTER COHORT STUDY

### 91.1 **MI KYOUNG SONG** ^1^, SO YUN JUN^1^, YOUNG HYE RYU^1^, SEUNG MIN BAEK^1^, HYE WON KWON^1^, JI EUN BAN^2^, AH YOUNG KIM^3^, MI JIN KIM^4^, JAE SUK BAEK^4^, JA KYOUNG YOON^3^, HEE JOUNG CHOI^5^, MIN JUNG CHO^6^, CHANG SIN KIM^3^, INSU CHOI^7^, JOUNG HEE BYUN^8^, JIHYE YOU^9^, HEIRIM LEE^10^, JAE YOON NA^11^, JUE SEOUNG LEE^12^, YOUNGKUK CHO^13^, JOOWON LEE^14^, LUCY YOUNGMIN EUN^15^, JUNGHYE KWON^16^, EUN‐JUNG BAE^1^, JUNE HUH^16^

#### 91.1.1 ^1^Seoul National University Hospital, Seoul, Korea, Republic of,^2^Sejong General Hospital, Bucheon, Gyeonggi‐do, Korea, Republic of,^3^Severance Cardiovascular Hospital, Yonsei University College of Medicine, Seoul, Korea, Republic of,^4^University of Ulsan College of Medicine, Seoul, Korea, Republic of,^5^Keimyung University School of Medicine, Daegu, Korea, Republic of,^6^Gyungsang National University Changwon Hospital, Changwon, Korea, Republic of,^7^Chonnam National University Medical School, Chonnam, Korea, Republic of,^8^Pusan National University Yangsan Hospital, Pusan National University School of Medicine, Pusan Yangsan, Korea, Republic of,^9^Jeonbuk National University Medical School, Jeonju, Korea, Republic of,^10^Pusan National University Hospital, Busan, Korea, Republic of,^11^Hanyang University College of Medicine, Seoul, Korea, Republic of,^12^Korea University College of Medicine and Korea University Medical Center, Seoul, Korea, Republic of,^13^College of Medicine Chosun University, Gwangju, Korea, Republic of,^14^Bundang Seoul National University Hospital, Bundang, Korea, Republic of,^15^Gangnam Severance Hospital, Yonsei University College of Medicine, Seoul, Korea, Republic of,^16^Heart vascular stroke institute, Samsung Medical Center, Sungkyunkwan University School of Medicine, Seoul, Korea, Republic of

**Introduction:** Congenital long QT syndrome (LQTS) is the most common inherited arrhythmic syndrome. Despite numerous studies, there has not been much data from Asia. This study aimed to elucidate the clinical characteristics and outcomes of patients with type 1, 2, and 3 LQTS from a nationwide Korean multicenter study.

**Methods:** We conducted a retrospective study for pediatric patients with inherited arrhythmia syndrome, including 17 pediatric cardiology centers in South Korea from 2022 to 2023. One hundred twenty nine patients with LQTS1, 2, and 3 were identified. Life‐threatening arrhythmic events (LAEs) were defined as ventricular tachycardia/fibrillation (VT/VF), appropriate implantable cardioverter‐defibrillator (ICD) shock, aborted cardiac arrest, and sudden cardiac death.

**Results: Results:** Fifty six percent of patients were male. The mean age of patients at diagnosis was 7.5±5.3 years with a mean follow‐up duration of 5.4±5.1 years. The most common variant was KCNQ1 (61%), followed by KCNH2 (24%), and SCN5A (15%). Symptoms at diagnosis included unexplained syncope (34%), and aborted cardiac arrest (11%).Beta‐blockers were used in 114 patients (88%). An ICD was implanted in 22 patients (17%) for primary prevention in 14%. Appropriate shocks were delivered in 12 patients (55%). Left sympathetic denervation was performed in 5 patients. LAE occurred in 14 patients (11%) during follow‐up and it was associated with VT/VF or cardiac arrest at diagnosis, female, fetal bradycardia, prolonged corrected QT interval at diagnosis, and LQTS2 and 3. Variants affecting transmembrane pore were found in 65 patients, unrelated to LAEs. On multivariate analysis, LAE at diagnosis (HR 7.791, 95% C.I. 2.562‐23.690) and corrected QT interval (HR 1.014, 95% C.I. 1.003‐1.025) were independent risk factors for LAEs.

**Conclusions:** LQTS patients had a significant risk for LAEs. This study showed that increased QTc and LAEs at diagnosis were risk factors for LAE during follow‐up in Korean patients. Longer‐term follow‐up and international collaborative data acquisition are required for predicting outcomes and managing LQTS patients.

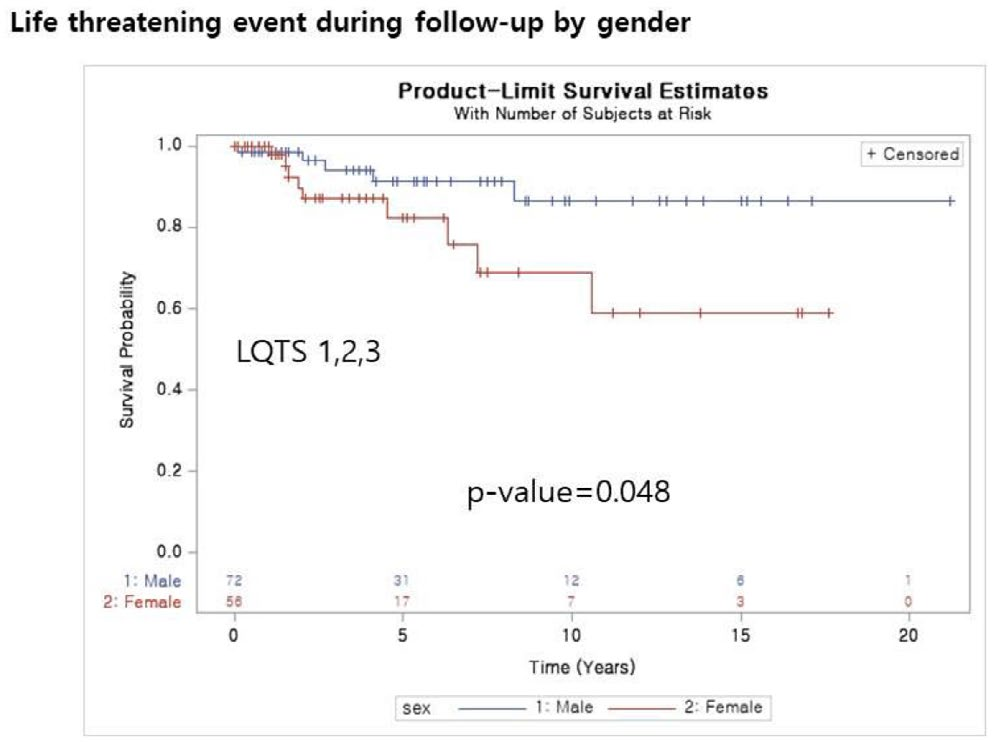

Chair

**M. Stiles**

Waikato Hospital, New Zealand

## 92 ASSOCIATION BETWEEN POST‐PROCEDURAL ECHOCARDIOGRAPHIC DATA, BNP LEVELS, AND CLINICAL OUTCOMES IN PATIENTS WITH PRESERVED EJECTION FRACTION FOLLOWING CATHETER ABLATION FOR ATRIAL FIBRILLATION

### 92.1 **HIRONOBU SUMIYOSHI**, HIROSHI TASAKA, KENTA YOSHIDA, RYUKI CHATANI, ATSUSHI SAKATA, MITSURU YOSHINO, KAZUSHIGE KADOTA

#### 92.1.1 Kurashiki central hospital, Kurashiki, Japan

**Introduction:** Atrial fibrillation (AF) is frequently associated with heart failure with preserved ejection fraction (HFpEF), but the diagnosis and prediction of the outcomes of HFpEF are difficult. Notably, the Heart Failure Association of the European Society of Cardiology proposed the use of the HFA‐PEFF score composed of echocardiographic data and BNP levels in the diagnosis of HFpEF. This study aimed to assess the prognostic value of the pre‐ and postprocedural HFA‐PEFF scores in patients with preserved ejection fraction (EF) after catheter ablation (CA) for AF. The primary endpoint was a composite of cardiac hospitalization for cardiovascular events and all‐cause mortality. The secondary endpoint was AF recurrence.

**Methods:** Overall, 354 patients with AF and preserved EF who underwent CA as well as blood tests and transthoracic echocardiography 2 weeks before and 6 months after CA from January 2018 to December 2019 were retrospectively enrolled in the study.

**Results:** In the 354 participants, univariate analysis showed that the postprocedural HFA‐PEFF score was associated with a 3‐year risk of the primary endpoint (hazard ratio [HR] = 3.73; 95% confidence interval [CI] = 2.07‐6.73; *P* < 0.001), whereas the preprocedural HFA‐PEFF score was not (HR = 1.24, 95% CI = 0.82‐1.86, *P* = 0.307). Further, the association between the postprocedural HFA‐PEFF score and primary endpoint was not modified even after including other relevant variables into the score. Similar to the primary endpoint, the postprocedural HFA‐PEFF score was associated with the 3‐year risk of AF recurrence (*P* < 0.001).

**Conclusions:** In patients with preserved EF undergoing AF ablation, the HFA‐PEFF score at 6 months after CA was associated with the primary endpoint and AF recurrence at the 3‐year follow‐up.

## 93 EFFECTS OF REVERSED C‐CURVE TECHNIQUE‐GUIDED ABLATION ON VENTRICULAR ARRHYTHMIAS ORIGINATING FROM THE PARA‐HISIAN REGION

### 93.1 **TOMOMICHI SUZUKI**, RYOTARO OKAMOTO, AKIO MONJI, SEIICHI HAYAKAWA, MASAYA KIMURA, MITSUTAKA MAKINO

#### 93.1.1 Nishichita General Hospital, Tokai, Japan

**Introduction:** Catheter ablation of ventricular arrhythmias (VAs) originating from the para‐Hisian region can be challenging because of adjacent conduction tissue. The perioperative success rate and long‐term success rate of catheter ablation above the septal leaflet of the tricuspid valve is not ideal. This study aimed to investigate the safety and efficacy of catheter ablation for para‐Hisian VAs under the septal valve with reversed C‐curve technique.

**Methods:** Fifteen consecutive patients with para‐Hisian VAs were included. Systematic mapping and radiofrequency catheter ablation (RFCA) were performed with two mapping methods: mapping above the tricuspid septal valve in 5 patients (Group A) and mapping under the tricuspid septal valve with reversed C‐curve technique in 10 patients (Group R).

**Results:** The earliest ventricular activation preceding surface QRS in Group R was significantly larger than that in Group A (26.2 ± 7.2 vs 20.2 ± 5.9 ms, P ≤ 0.01). His bundle potentials were recorded at the earliest ventricular activation site in 2 of 10 (20.0%) Group R patients, and none of 5 (0%) Group A patients. There was no significant difference in pace map correlation at the earliest ventricular activation site between the two groups (92.9 ± 4.0% in Group R and 94.6 ± 1.1% in Group A). RF ablation achieved acute success in 9 of 10 (90.0%) Group R patients, and 2 of 5 (40.0%) Group A patients. Of the Group A patients, 3 patients underwent ablation in the left ventricular outflow tract. The mean number of radiofrequency applications was 3.3 in Group R and 2.0 in Group A. The procedure time was significantly shorter in Group R patients than Group A patients (140.1 ± 25.9 vs 199.8 ± 46.8 min; P ≤ 0.05). In 1 patient (Group R) and 3 patients (Group A) with acute failure, VAs were eliminated the day after RFCA procedures. During a mean follow‐up of 21.3 ± 7.3 months, no patients presented with VAs recurrence. No procedure‐related complications occurred during ablation or follow‐up.

**Conclusions:** RFCA using reversed C‐curve technique is effective and safe for the acute elimination of para‐Hisian VAs.

Chair

**H. Tada**

University of Fukui, Japan

## 94 NT‐PROBNP AS A DISCRIMINATOR IN SCREENING FOR ATRIAL FIBRILLATION ‐ A CLINICAL TRIAL UPDATE FROM STROKESTOP II

### 94.1 **EMMA SVENNBERG** ^1,2^, KATRIN KEMP GUDMUNDSDOTTIR^3^, LEIF FRIBERG^4^, TOVE HYGRELL^4^, VIVEKA FRYKMAN^4^, FARIS AL‐KHALILI^4^, ZIAD HIJAZI^5^, MÅRTEN ROSENQVIST^4^, JOHAN ENGDAHL^6^

#### 94.1.1 ^1^Karolinska University Hospital, Stockholm, Sweden,^2^Karolinska Institutet, Department of Medicine Huddinge, Stockholm, Sweden,^3^Karolinska Institutet, Danderyds University hospital, Stockholm, Sweden,^4^Karolinska Institutet, Danderyd University Hospital, Stockholm, Sweden,^5^UCR‐Uppsala Clinical Research center, Associate professor at Department of Medical Sciences; Cardiology, Uppsala, Sweden,^6^Karolinska Institute, Dept of Clinical Sciences, Danderyd University hospital, Stockholm, Sweden

**Introduction:** The STROKESTOP II trial is a randomized controlled population‐based screening study focused on detecting atrial fibrillation (AF) in individuals aged 75/76 using NT‐proBNP levels as a discriminator to identify which participants should undergo more intensive screening. We hypothesized that elevated levels of NT‐proBNP are predictive of a future risk of AF, and morbidities thereof.

**Methods:** In total, 13,905 individuals were randomized to be invited to screening for AF and 49.2 % (6,843/13,905) participated. Participants free of AF with NT‐proBNP <125 ng/l had a single ECGs, participants with higher levels of NT‐proBNP ≥125 ng/l were screened more intensely. Here, the primary combined endpoint of stroke and systemic embolism, ischemic stroke, and incident AF after a median of 5.1 years in participants in the STROKESTOP II trial who had the biomarker NT‐proBNP collected and had no prior history of AF is reported.

**Results:** Of the 6,843 participants in the STROKESTOP II‐trial, n=6,161 participants had no prior AF, had NT‐proBNP collected and were included. Most included participants, 59 % (3,638/6,161) had NT‐proBNP levels ≥125 ng/l. After a median of five years more participants with NT‐proBNP ≥125 ng/l n=458 (12.6%) had incident AF compared to the group with lower NT‐proBNP levels, n=124 (4.9 %). For the primary combined endpoint participants with NT‐proBNP ≥125 ng/l sustained significantly more events compared to the group with NT‐proBNP <125 ng/l, 0.94 (95% CI 0.81‐1.08) events/100 risk‐years versus 0.6 (95% CI 0.48‐0.74) events/100 risk‐years, with an unadjusted hazard ratio (HR) of 1.57 (CI 1.21‐2.03), p=0.001 . After extensive adjustment, the adjusted (a) HR was 1.47 (CI 1.13‐1.92), p=0.004.For the secondary endpoint of ischemic stroke, participants with NT‐proBNP ≥125 ng/l sustained 0.79 (0.68‐0.93) events/100 risk‐years compared to 0.51 (0.41‐0.64) events/100 years in the low NT‐proBNP group with an unadjusted HR 1.56 (1.18‐2.05, p=0.002), aHR 1.46 (CI 1.10‐1.95), p=0.009.

**Conclusions:** NT‐proBNP is an effective discriminator for incident AF and future cardiovascular risk in population‐based screening programs for AF.

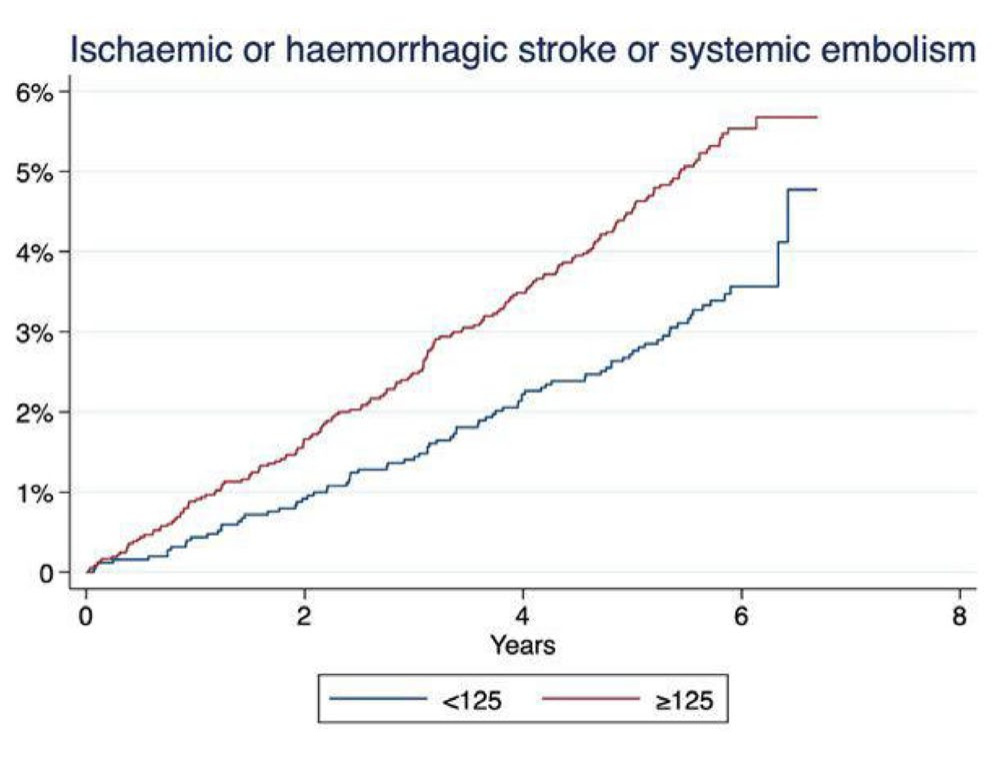

## 95 DUAL‐CHAMBER INTRAPROCEDURAL IMPEDANCE IN A NOVEL LEADLESS PACEMAKER CAN BE PREDICTIVE OF CHRONIC CAPTURE THRESHOLD

### 95.1 **MARK TK TAM** ^1^, CHIN‐PANG CHAN^2^, YAT SUN JOSEPH CHAN^2^, ERIC JOHNSON^3^, VIVEK REDDY^4^, JAMES IP^5^, RAHUL DOSHI^6^, DEREK EXNER^7^, PASCAL DEFAYE^8^, ROBERT CANBY^9^, MARIA GRAZIA BONGIORNI^10^, MORIO SHODA^11^, GERHARD HINDRICKS^12^, CHRISTIE HUFF^3^, DREW SAUCERMAN^3^, KENNETH BRUHNS^3^, REINOUD KNOPS^13^

#### 95.1.1 ^1^Chinese University of Hong Kong, Hong Kong, Hong Kong,^2^Prince of Wales Hospital, Hong Kong, Hong Kong,^3^Abbott, Sylmar, CA,^4^Icahn School of Medicine at Mount Sinai, New York, NY,^5^Weill Cornell Medicine/ New York Presbyterian Hospital, New York, NY,^6^HonorHealth Cardiac Arrhythmia Group, Scottsdale, AZ,^7^Foothills Medical Center, Calgary, AB, Canada,^8^CHU Grenoble Alpes, Grenoble, France,^9^Texas Cardiac Arrhythmia Institute, Austin, TX,^10^San Rossore Private Hospital and Medical Center, Pisa, Italy,^11^Tokyo Women's Medical University, Tokyo, Japan,^12^German Heart Center of the Charite, Berlin, Germany,^13^Amsterdam UMC, Amsterdam, Netherlands

**Introduction:** Traditional pacemaker implantations are guided by electrical measurements such as impedance and pacing capture thresholds (PCT). This analysis aimed to characterize leadless pacemaker (LP) impedance trends during implantation of the dual‐chamber Aveir DR pacemakers (Abbott Medical) and their associations with chronic PCT.

**Methods:** Retrospective analyses were performed on implant and follow‐up data from the Aveir DR i2i Study (NCT#:05252702). Patients with RV and RA impedance during mapping (LP contacting myocardium), tether (fixed LP), and post‐release (no catheter connection), and PCT obtained at 3 months were included. Impedance values were analyzed along with 3‐month PCT (0.4ms pulse width). Variables are reported as mean±SD. Student's t‐test or ANOVA was used to determine differences in means.

**Results:** 217 RV and 218 RA patients were included. RV and RA impedance values at mapping, tether, and release were 476±126Ω, 723±285Ω, and 757±324Ω, and 303±51Ω, 313±60Ω, and 330±59Ω, respectively. RV mapping impedance was significantly lower than tether or release (p<0.01). LP impedance in the RA was significantly higher at release vs. mapping or tether (p<0.01). RV PCT at 3‐months was significantly lower in patients with increase in impedance from mapping to tether (0.6±0.4V) vs. no change or decrease (1.4±1.4V, p<0.01). Change in impedance between mapping, tether, and release did not predict 3‐month PCT in RA implants. RV tether impedance >520Ω (0.5±0.2V) significantly predicted lower 3‐month PCT vs. tether impedance ≤520Ω (1.1±1.1V, p<0.01). Similarly, in the RA a tether impedance of >330Ω (0.6±0.5V) significantly predicted lower 3‐month PCT vs. tether impedance ≤330Ω (0.9 ±0.8V, p<0.01).

**Conclusions:** Monitoring LP impedance during implant may help identify optimal implant locations in the right ventricle and right atrium, resulting in lower chronic thresholds and potentially minimizing implant reattempts. While additional factors, such as electrogram characteristics, should be assessed during implant, this analysis provides initial interpretation of dual‐chamber leadless (Aveir DR) impedance trends.

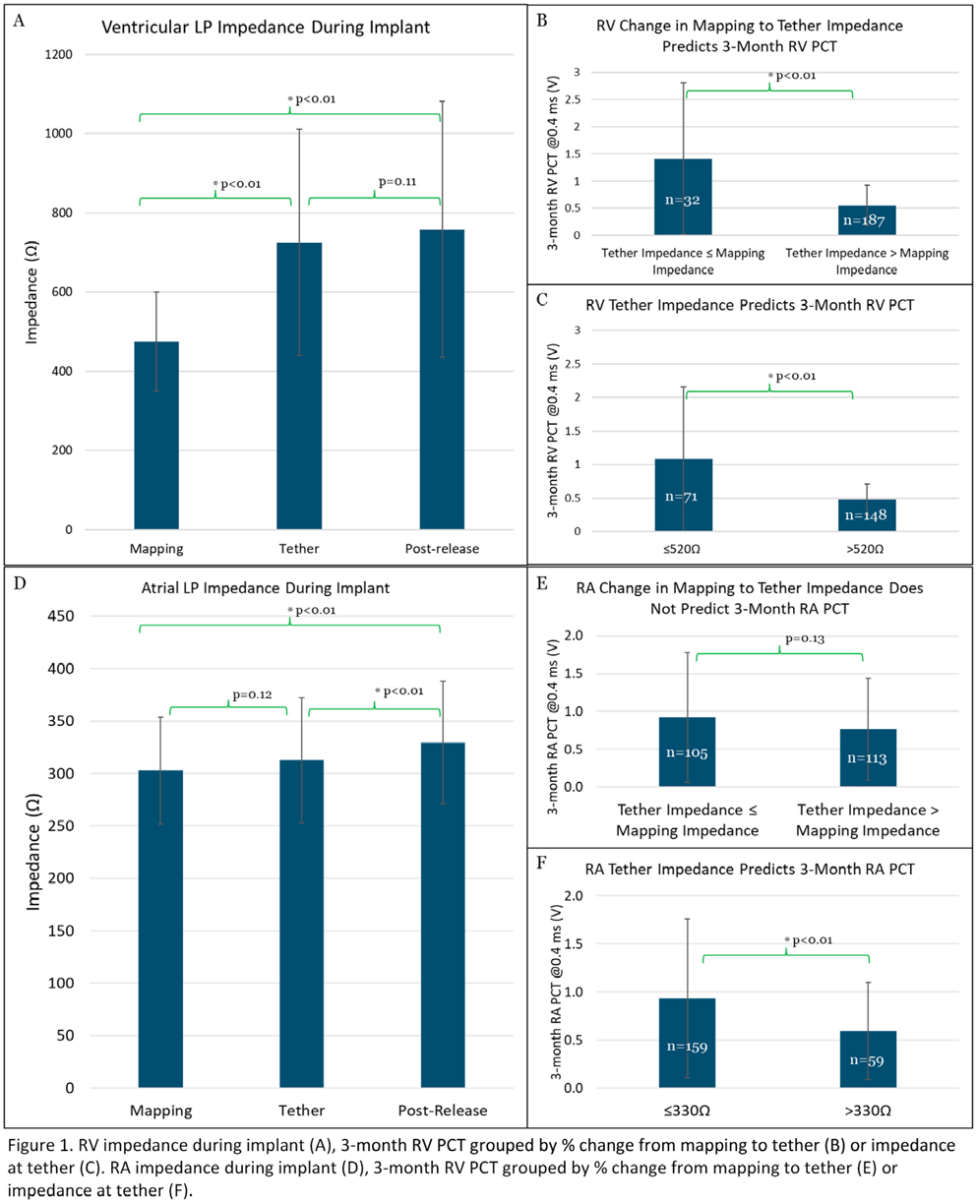

## 96 LONG TERM ADVERSE EFFECTS OF PULSED FIELD ABLATION TO HUMAN CORONARY ARTERIES, AN INTRA‐CORONARY IMAGING STUDY

### 96.1 **MARK TK TAM** ^1^, JOSEPH YS CHAN^2^, CHIN PANG CHAN^2^, EUGENE B WU^2^, ANGEL LAI^1^, ALEX CK AU^2^, GUANG MING TAN^1^, BRYAN P YAN^1^

#### 96.1.1 ^1^The Chinese University of Hong Kong, Hong Kong, Hong Kong,^2^Prince of Wales Hospital, Hong Kong, Hong Kong

**Introduction:** Pulsed field ablation (PFA) is known to cause acute coronary spasm. Whether this will translate into long term coronary stenosis is unknown.

**Methods:** Following pulmonary vein isolation with PFA (Farapulse, Boston Scientific, MA), consecutive patients undergoing ablation for mitral isthmus (MI) or cavo‐tricuspid isthmus (CTI) were included. They underwent coronary angiogram and OCT of the coronary vessel of interest before and immediately after ablation at peak spasm. Bolus intracoronary nitroglycerine was given before and throughout ablation. At 3 months, repeat OCTs were performed. The previous spasm site was located in follow‐up OCT by measuring distance from arterial bifurcation. Outcomes included change in vascular wall area (difference between vascular area bordered by external elastic laminar, and vascular lumen) and change in vascular luminal area. The investigator who assessed follow‐up OCT was blinded to the paired pre‐ablation imaging. The luminal area change in a predetermined reference site remote from ablation was also computed to correct for changes of vascular tone between OCT runs.

**Results:** 21 consecutive patients undergoing MI or CTI ablation were included. One did not undergo PFA due to coincidental finding of critical coronary lesion in proximity in the area to be ablated. One defaulted follow up imaging. 19 patients had paired imaging data in 20 coronary vessels (18 right coronary arteries and 2 left circumflex arteries). At the ablation site, vascular wall area increased significantly at 3 months by 0.47 +/‐0.69mm^2^ (p<0.01), or 18.7%. The gross luminal area reduced by 1.19 +/‐ 1.85mm^2, p=0.01. After correction of vascular tone, the mean reduction of LA was 1.08+/‐ 1.52mm^2 (p<0.01), or 12.0%. These changes was not observed in the pre‐determined reference site remote from ablation. A case of CTI ablation causing acute right coronary spasm and chronic coronary stenosis is shown in figure 1.

**Conclusions:** PFA for CTI and MI in proximity to coronary vessels is associated with increase in vascular wall area and reduction in luminal area at 3 months. We therefore caution on the routine use of PFA near coronary vessels.

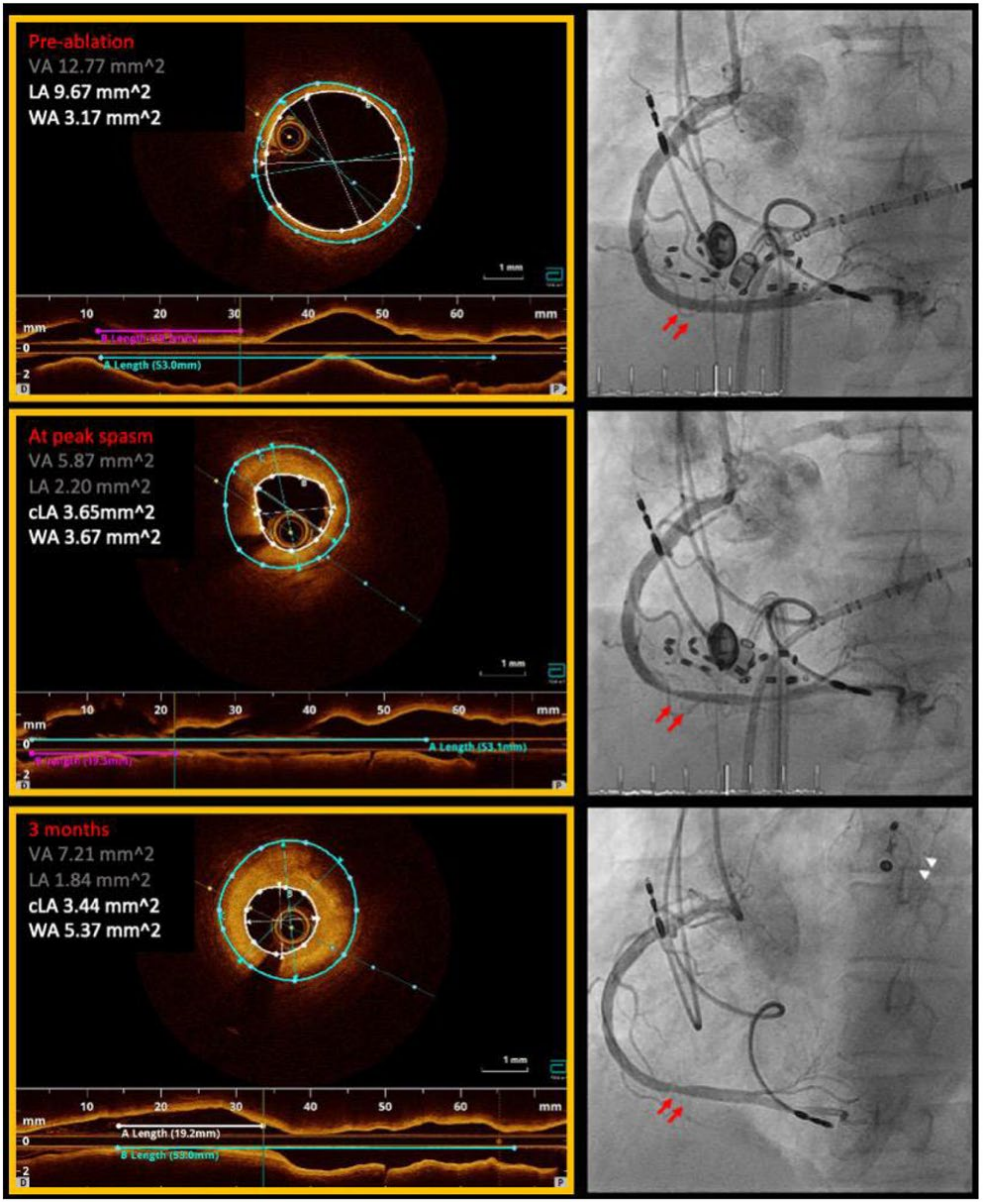

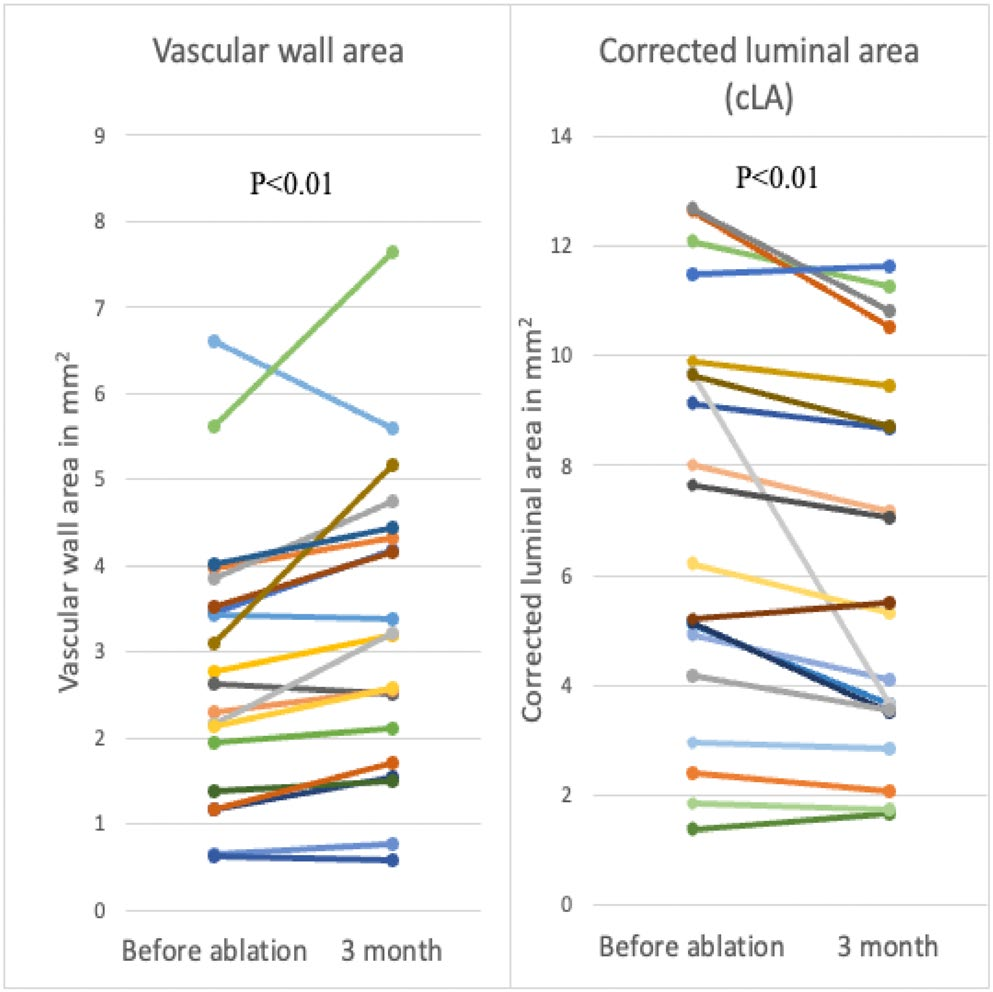

## 97 FEASIBILITY, SAFETY AND OUTCOMES OF CONDUCTION SYSTEM PACING FOR BRADYCARDIA AMONGST THE VERY ELDERLY

### 97.1 **EUGENE TAN** ^1^, RODNEY SOH^1^, JIE‐YING LEE^1^, ELAINE BOEY^2^, SIEW‐PANG CHAN^1^, SWEE‐CHONG SEOW^1^, LISA TEO^3^, COLIN YEO^3^, VERN HSEN TAN^3^, PIPIN KOJODJOJO^1^

#### 97.1.1 ^1^National University Heart Centre Singapore, Singapore, Singapore,^2^Ng Teng Fong General Hospital, Singapore, Singapore,^3^Changi General Hospital, Singapore, Singapore

**Introduction:** The impact of age (>85 vs ≤85 years) on clinical outcomes and pacemaker performance of conduction system pacing (CSP) compared to right ventricular pacing (RVP) were examined.

**Methods:** Consecutive patients from a prospective, multicenter study with pacemakers implanted for bradycardia were studied. The primary outcome was a composite of heart failure (HF)‐hospitalizations, pacing‐induced cardiomyopathy requiring cardiac resynchronization therapy or all‐cause mortality. Secondary outcomes were acutely successful CSP, absence of pacing‐complications, optimal pacemaker performance defined as pacing thresholds<2.5V, R‐wave amplitude≥5V and absence of complications, threshold stability (no increases of >1V) and persistence of His‐Purkinje capture on follow‐up.

**Results:** Among 984 patients (age 74.1±11.2years, 41% CSP, 16% ≥85years), CSP was independently associated with reduced hazard of the primary outcome compared to RVP, regardless of age‐group (<85years: adjusted hazard ratio[AHR] 0.63, 95% confidence interval[CI] 0.40‐0.98; ≥85years: AHR 0.40, 95%CI 0.17‐0.94). Among patients with CSP, age did not significantly impact the secondary outcomes of acute CSP success (86% vs 88%), pacing complications (19% vs 11%), optimal pacemaker performance (64% vs 69%), threshold stability (96% vs 96%) and persistent His‐Purkinje capture (86% vs 91%) on follow‐up (all p>0.05).

**Conclusions:** CSP improves clinical outcomes in all age‐groups, without compromising procedural safety or pacemaker performance in the very elderly.

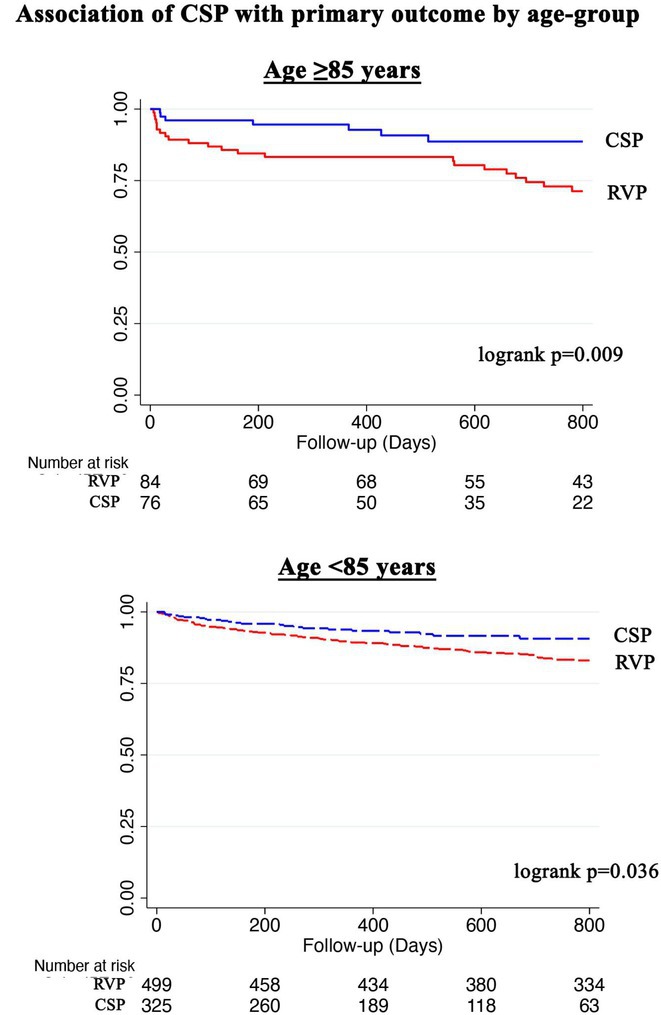

## 98 REPORTING OF COMPLICATIONS FOLLOWING AF ABLATION: COMPARISON OF THE MANUFACTURER AND USER FACILITY DEVICE EXPERIENCE FDA DATABASE AND A VOLUNTARY INVITATION‐BASED REGISTRY ‐ THE POTTER‐AF 3 STUDY

### 98.1 **ROLAND TILZ** ^1^, HELMUT PÜRERFELLNER^2^, KARL‐HEINZ KUCK^1^, JOSÉ LUIS MERINO^3^, VANESSA SCHMIDT^1^, JULIA VOGLER^1^, KUN XIANG^4^, EKIN UZUNOGLU^5^, CHRISTIAN‐HENDRIK HEEGER^6^, HARIKRISHNA TANDRI^7^, FABRIZIO ASSIS^8^, DANIEL STEVEN^9^, CHRISTIAN VELTMANN^10^, JOHN CATANZARO^8^, SORIN STEFAN POPESCU^1^

#### 98.1.1 ^1^University Hospital Schleswig‐Holstein, Lübeck, Germany,^2^Ordensklinikum Linz Elisabethinen, Linz, Austria,^3^La Paz University Hospital, Universidad Autónoma de Madrid, Madrid, Spain,^4^University of Florida Health, Gainesville, FL,^5^University of Florida Health Science Center, Jacksonville, FL,^6^Asklepios Klinik Hamburg Altona, Hamburg, Germany,^7^Vanderbilt University Medical Center, Nashville, TN,^8^East Carolina University Health, Greenville, NC,^9^Heart Center University Cologne, Cologne, Germany,^10^Heart Center Bremen, Bremen, Germany

**Introduction:** The Manufacturer and User Facility Device Experience (MAUDE) database houses medical device reports submitted to the U.S. Food and Drug Administration (FDA) by mandatory (e.g. manufacturers and user facilities) and voluntary reporters. Data about the efficacy and quality of this reporting system are sparse.

**Methods:** The esophageal fistula (EF) reports following AF ablation in the mandatory/voluntary MAUDE Database (MAUDE Group) were compared to those reported in the voluntary, invitation‐based POTTER‐AF registry (POTTER‐AF 1 Group) between 01/08/2009 and 31/08/2019.

**Results:** EF were reported in 47 patients in the MAUDE group and in 81 patients in the POTTER‐AF 1 group. In the MAUDE Group most EF were reported in the US (66.7%) and France (12.1%), while in the POTTER‐AF 1 Group most EF were reported in France (34.6%) and Germany (22.2%). Procedures were performed with radiofrequency, cryoenergy or laser energy in 66.0%, 31.9% and 2.1% in the MAUDE group and in 96.3%, 2.5% and 1.2% in the POTTER‐AF 1 Group. The diagnostic method was reported in 38.3% of patients in the MAUDE Group and in 98.8% in the POTTER‐AF 1 Group, the treatment in 57.4% and 100%, respectively and the outcome in all patients. The CT was the most common diagnostic method. In the MAUDE Group, treatment was surgical (51.9%), endoscopic (37.0%), combined (3.7%) or conservative (7.4%), compared to 43.2%, 19.8%, 7.4% and 29.6% in the POTTER‐AF 1 Group. Overall mortality was 76.6% in the MAUDE Group and 61.7% in the POTTER‐AF 1 Group (p=0.118). The mortality for patients undergoing surgery, endoscopic treatment only and conservative treatment were 80%, 80% and 100% in the former and 51.2%, 50.0% and 87.5% respectively in the latter group.

**Conclusions:** Significantly less cases of EF were reported in the mandatory/voluntary FDA database as compared to the invitation‐based POTTER‐AF registry. The data quality in the former was significantly poorer. The observed differences call for further study to identify their causes and enhance reporting on lethal complications linked to medical products.

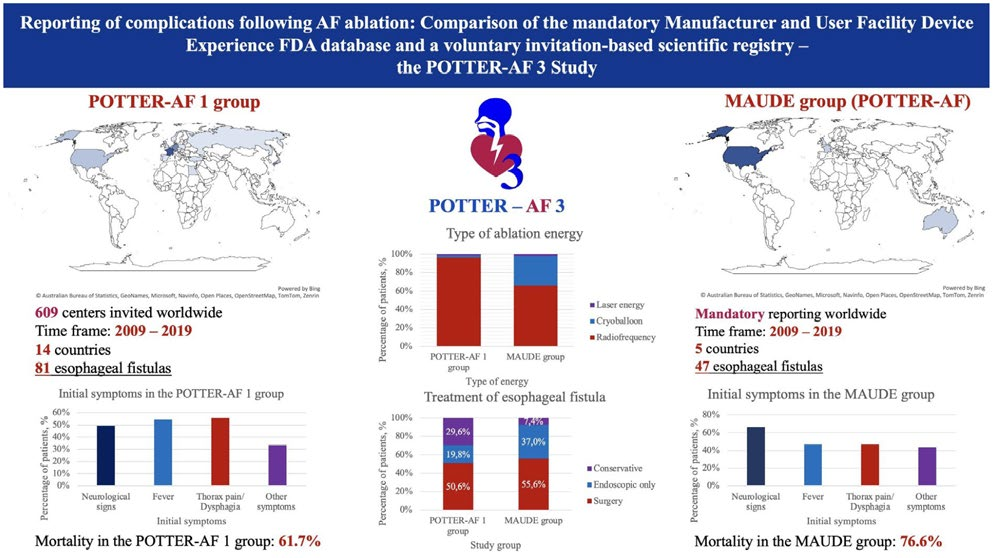

## 99 CONDUCTION SYSTEM PACING VALUES BEYOND A PHYSIOLOGICAL VENTRICULAR SYNCHRONY

### 99.1 **DAT TRAN CAO**, DUNG KIEU NGOC, PHUONG TRAN LE UYEN, SON NGUYEN KHAC LE, CHUONG NGUYEN KHAC THIEN, DUY VO THAI, THUC NGUYEN TRI

#### 99.1.1 Cho Ray Hospital, Ho Chi Minh, Viet Nam

**Introduction:** Conduction system pacing (CSP) has emerged as a novel treatment for patients with bradycardia. Besides the synchronous activation offered by these devices, CSP might also impact the occurrence and incidence of atrial high‐rate episodes, which are deemed to be precursors for atrial fibrillation (AF). We aimed to determine the risks of AHRE in patients implanted with His bundle pacing and left bundle pacing devices compared to traditional right ventricular septal pacing at Cho Ray Hospital.

**Methods:** Two groups of patients assigned with conduction system pacing or right septal pacing devices were followed up for 6 months. Assessment of AHRE was performed based on pacemaker monitoring function at 6 months post‐implantation. The lower cutoff duration to define AHRE in our study was 6 minutes

**Results:** A total of 301 patients were enrolled in our study (female: 53%, average age of 62), of which 156 patients were implanted with CSP pacemakers while the other 145 were treated with right septal pacing. QRS duration was significantly shorter in the CSP groups (121±21.3 vs 102.6 ± 23.5, p< 0.05). During the 6‐month follow‐up post‐device implantation, only 7 patients in the CSP group developed AHRE, while 44 patients in the traditional RV pacing group had AHRE documented in their pacemaker (6.4% vs 30.34%, p< 0.005). Within the CSP group, patients who received his bundle pacing would have shorter QRS duration (96.9 ± 12.2 vs 109.2 ± 10.54, p=0.02) but the higher non‐selective threshold difference was not significant (1.5 ± 0.87 vs 1.32 ± 0.93, p=0.198). 2 patients in the left bundle pacing groups were found to have AHRE while 5 patients with His bundle pacing experienced these events.

**Conclusions:** Conduction system pacing not only offers more physiological electrical activation of the ventricle but also simultaneously decreases the risk of AHRE or AF in bradycardia patients requiring pacemakers. This result has implications for the benefits of CSP in preventing the development of AF, which might have a role in pacing‐induced cardiomyopathy

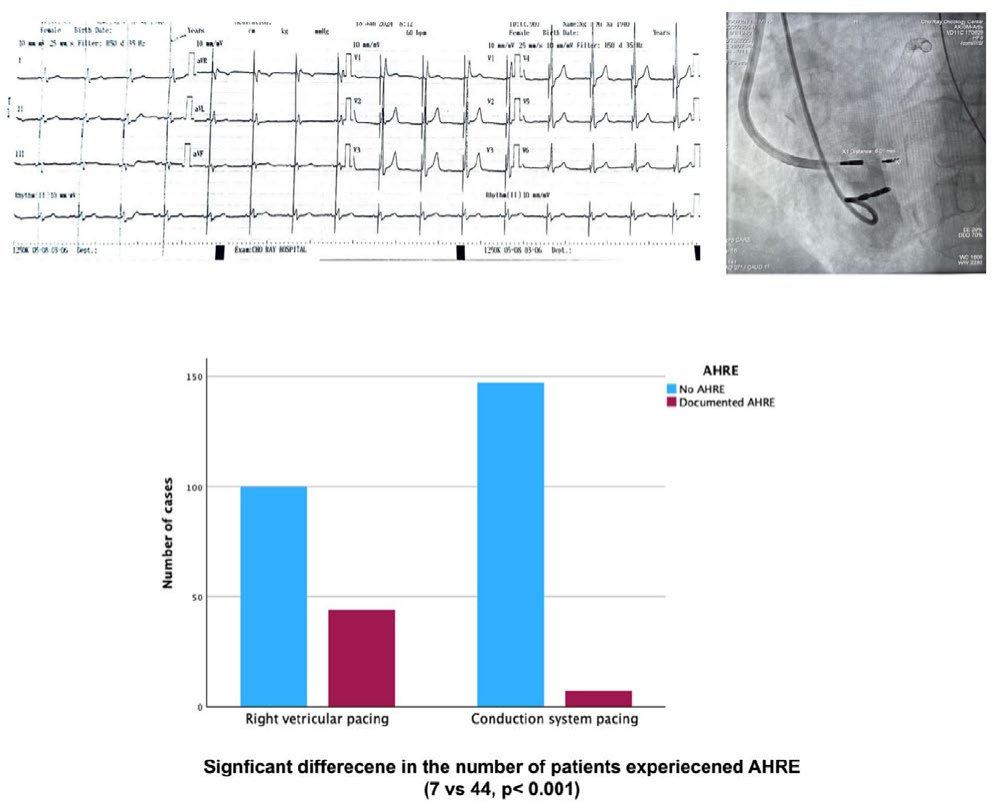

## 100 LEFT BUNDLE BRANCH AREA PACING IN TAVR PATIENTS. IMPLEMENTATION OF THE NOVEL PACING MODALITY IN FRAILTY PATIENTS

### 100.1 **YERLAN TURUBAYEV**, ABAY BAKYTZHANULY, SERIK BAGIBAYEV, ZHANDOS YESSILBAYEV, SARDOR YULDASHOV, ABDURASHID MUSSAYEV, OMIRBEK NURALINOV

#### 100.1.1 University Medical Center CF, Astana, Kazakhstan

**Introduction:** Transcatheter aortic valve replacement is a safe alternative to open‐heart surgery in high‐risk patients with symptomatic severe aortic stenosis due to multiple comorbidities, advanced age, and severe left ventricle dysfunction. Complete AV block and new onset LBBB ‐ most commonly reported conduction abnormalities after TAVR. Adverse clinical outcomes associated with chronic RV pacing after TAVR are widely discussed in multiply studies. LBBAP has emerged as an alternative for RV pacing with acceptable outcomes. The aim of our study to assess the feasibility and effectiveness of LBBAP in patients after TAVR.

**Methods:** Procedural data included capture threshold, R‐wave amplitude, impedance, Stim‐LVAT, pacing QRS duration, fluoroscopy time. 12‐lead ECG and TTE performed before, immediately after procedure and next day after procedure.

**Results:** Total of 302 patients underwent TAVR procedure in the period from Jan. 2022 to Nov. 2023. In this cohort 38 patients required ventricular pacing in postoperative period. 24 patients who underwent permanent pacing using LBBAP were included to this observational study. We collected baseline patient characteristics, type of conduction dysorders, type of prosthetic valve. LBBAP resulted in significant decreasing of QRS duration, from 134 ± 51 ms at baseline to 113,8 ± 8,9 ms. LBBAP threshold was 0.64 ± 0.3 V at 0.5 ms at implant. R‐wave amplitudes and pacing impedances at implantation (531 ± 117 ohms vs. 744 ± 196 ohms). No major complications were noted in all cases. 1 patient had peri‐procedural lead dislodgement, which was resolved by repositioning the lead. 3 cases of septal perforation during procedure. In 1 case of stylet‐driven lead we noticed helix damage. In 3 patients with pre‐existing LBBB we had mechanically induced 3rd degree AV block.

**Conclusions:** Complete AV block and new onset LBBB after TAVR is still an important issue. LBBB correction using conduction system pacing is effective strategy to correct conduction dysorder. However, long‐term clinical outcomes and safety profile of physiological pacing data is limited and requires further investigations.

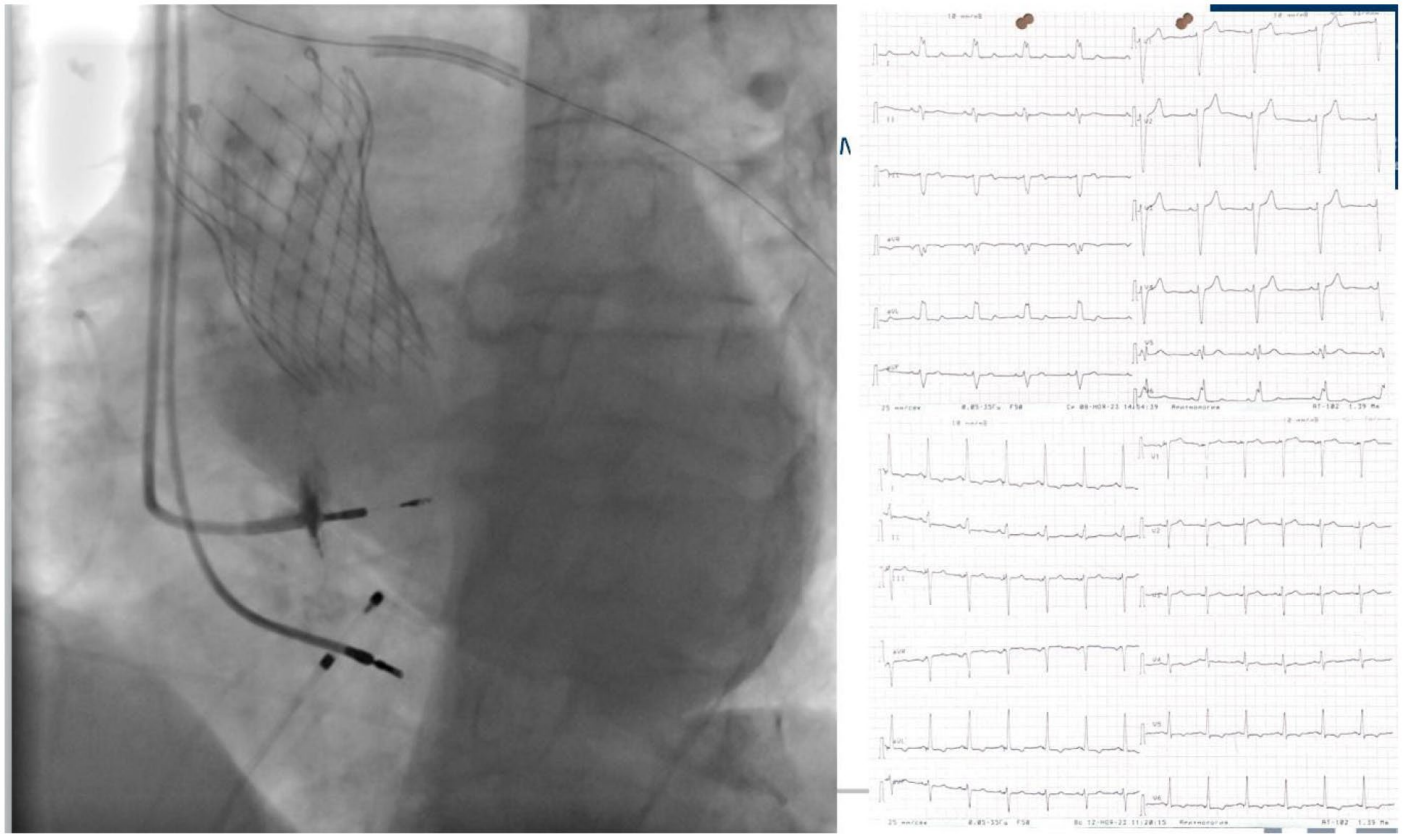

Chair

**D. J. Twomey**

James Cook University Hospital, Middlesbrough, United Kingdom

## 101 CONDUCTION SYSTEM PACING COMPARED TO BIVENTRICULAR PACING FOR CARDIAC RESYNCHRONIZATION THERAPY IN PATIENTS WITH MILDLY REDUCED LEFT VENTRICULAR EJECTION FRACTION (LVEF 36‐50%) : RESULTS FROM INTERNATIONAL COLLABORATIVE LBBAP STUDY (I‐CLAS) GROUP

### 101.1 **PUGAZHENDHI VIJAYARAMAN** ^1^, FRANCESCO ZANON^2^, SHUNMUGA SUNDARAM^3^, BENGT HERWEG^4^, PARIKSHIT SHARMA^5^, MANUEL MOLINA‐LERMA^6^, MAREK JASTRZęBSKI^7^, ZACHARY WHINNETT^8^, KEVIN VERNOOY^9^, RAJEEV K PATHAK^10^, RODERICK TUNG^11^, GAURAV UPADHYAY^12^, KAROL CURILA^13^, DIPEN ZALAVADIA^1^, NISCHAY SHAH^1^, LINA MARCANTONI^2^, MOHAMED GAD^14^, RAMEZ MORCOS^1^, PAWEL MOSKAL^7^, AKRITI NARAEN^8^, MISHAL MUMTAZ^4^, JAMARIO R SKEETE^5^, MIHAIL CHELU^14^, KENNETH A ELLENBOGEN^15^, OSCAR CANO^16^

#### 101.1.1 ^1^Geisinger Heart Institute, WilkeS Barre, PA,^2^Santa MariaDella Misericordia Hospital, Rovigo, Italy,^3^Velammal Medical College Hospital and Research Institute, Madurai, India,^4^USF Morsani College of Medicine, Tampa, FL,^5^Rush University, Chicago, IL,^6^Hospital Universitario Virgen de las Nieves, Granada, Spain,^7^First Department of Cardiology, Interventional Electrocardiology and Hypertension, Jagiellonian University, Krakow, Poland,^8^Imperial College, London, United Kingdom,^9^Maastricht University, Maastricht, Netherlands,^10^Australian National University, Canberra, Australia,^11^Banner Health, Phoenix, AZ,^12^University of Chicago, Chicago, IL,^13^Charles University, Prague, Czech Republic,^14^Baylor College of Medicine, Houston, TX,^15^VCU Health System, Richmond, VA,^16^Hospital Universitari i Politècnic La Fe and Centro de Investigaciones Biomédicas en RED en Enfermedades Cardiovasculares, Valencia, Spain

**Introduction:** Cardiac resynchronization therapy (CRT) with biventricular pacing (BVP) is a guideline‐recommended therapy in patients with mildly reduced left ventricular ejection fraction (mrLVEF 36‐50%), heart failure and wide QRS or indication for pacing. Conduction system pacing (CSP) utilizing left bundle branch area pacing (LBBAP) or His bundle pacing (HBP) has been shown to be a safe and physiologic alternative to BVP. The aim of this study was to compare the clinical outcomes between BVP and CSP among patients with mrLVEF undergoing CRT.

**Methods:** This registry included consecutive patients who underwent BVP or CSP in patients with mrLVEF between Jan 2018 to June 2023 at 16 international centers. Patient demographics, echocardiographic outcomes, heart failure hospitalization (HFH), mortality and lead complications were assessed. The primary outcome was the composite endpoint of time to death or HFH. Secondary endpoints included change in LVEF and individual endpoints of death and HFH.

**Results:** A total of 1004 patients met inclusion criteria: BVP 178, CSP 826 (HBP 154; LBBAP 672). Mean age was 73±13 yrs, female 34%, HTN 70%, DM 36%, CAD 38% and LVEF 42±5% (Table). Coronary sinus LV pacing thresholds and CSP thresholds were similar at implant and remained stable during follow‐up. Paced QRSd in CSP was significantly narrower than baseline (129±21 vs 142±33ms, p<0.001) and significantly narrower compared to BVP (144±19, p<0.001). LVEF improved during f/u in both groups (50±19 vs 48±10%, p=0.32). On multivariate analysis, CSP was associated with significant reduction in the primary endpoint of time to death or HFH compared to BVP (22% vs 34%; HR 0.62; 95%CI 0.46‐0.83; p=0.002) (Figure)

**Conclusions:** CSP improved clinical outcomes when compared to BVP in this large cohort of patients with mrLVEF indicated for CRT. Randomized controlled trials comparing CSP to BVP will be necessary in this population

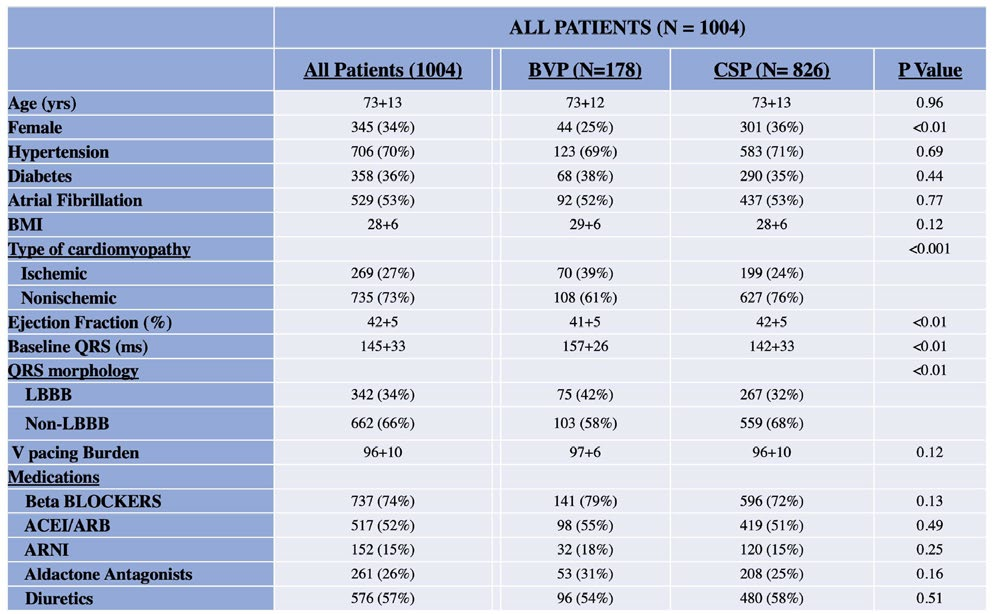

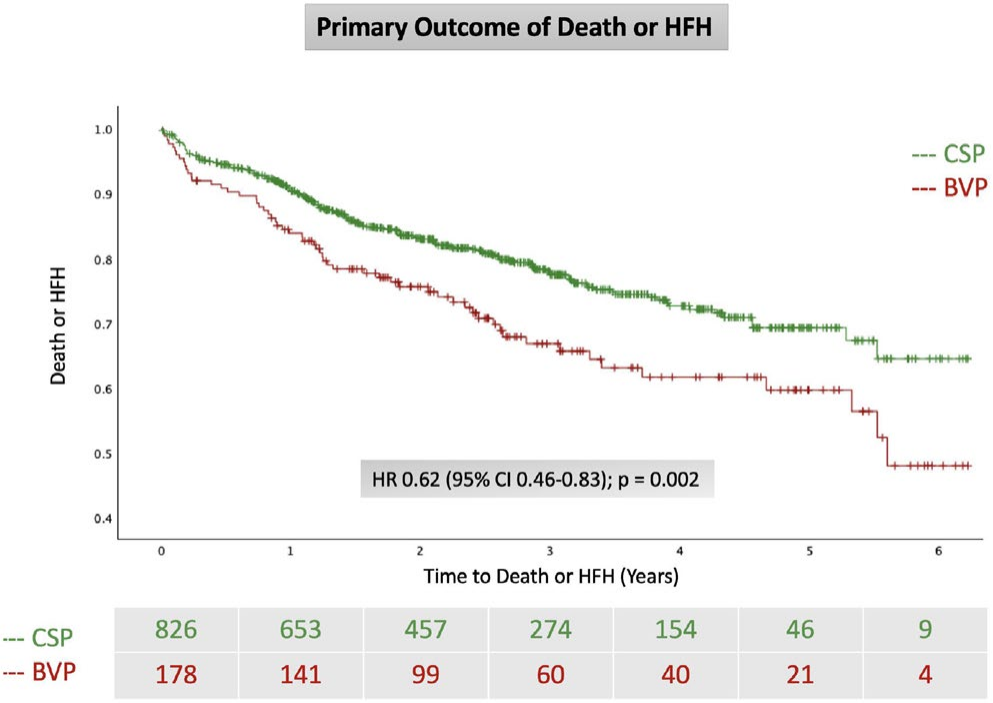

## 102 INTRAPROCEDURAL TRANSTHORACIC*EC*HOCARDIOGRAPHY TO ACHIEVE*L*EFT*B*UNDLE*B*RANCH*P*ACING (*EC‐LBBP)*: A PROSPECTIVE, CONTROLLED STUDY

### 102.1 **PUGAZHENDHI VIJAYARAMAN** ^1^, GRACE HUGHES^1^, MARILEE MANGANIELLO^1^, KAITLYN MROCZKA^1^, KAITLYN SACCO^1^, ALEXANDRA LAVER^1^, ELLIOT SCHMIDT^2^, VERNON MASCARENHAS^1^

#### 102.1.1 ^1^Geisinger Heart Institute, WilkeS Barre, PA,^2^Medtronic, Minneapolis, MN

**Introduction:** LBBP has gained rapid adoption. True LBB capture has varied from 30‐95% depending on the specificity of the criteria used. The aim of the study is to assess the feasibility and efficacy of intraprocedural transthoracic echo guidance to achieve LBB capture.

**Methods:** This was a prospective, case‐control study (NCT05646251). The pectoral region including echocardiographic windows(EW) were sterile‐draped using Ioban^R^. The lead was placed in the proximal RV septum and sheath orientation adjusted under echo. The lead was rotated while visualizing the lead advancement under echo until the tip reaches the LV subendocardium. LBB capture was strictly defined: Transition from nonselective to selective/LV septal capture; LBB potential with injury current; delta (HBP‐LBBP) V6RWPT of ≥10. The primary endpoint was successful LBB capture. Secondary endpoints were feasibility of echo‐guidance for LBBP lead implantation and confirmation of lead‐tip location.

**Results:** 63 patients were enrolled: 30 pts underwent EC‐LBBP (withdrew 2 due to poor EW) and compared with 30 pts (std approach). Mean age 74.4±10; female 45%; HTN 92%; cardiomyopathy 43%; AVB/AVN ablation 75%. Total and LBBP fluoroscopy duration were significantly shorter with EC‐LBBP. EC‐LBBP was 97% successful in achieving LBB capture (vs 70%, P<0.05) with LBB potentials (LB‐V 23±6ms) in 93% (vs 77%, 21±8ms). Morphology transition confirming LBB capture was seen in 85% vs 67% (P<0.05). Lead‐tip was visualized at LV subendocardium in 100% pts in EC‐LBBP. Feasibility of echo‐guidance for LBBP implantation was 91% (29/32). One patient in the control group had LV perforation at one day requiring repositioning.

**Conclusions:** EC‐LBBP was 97% successful in achieving LBB capture using strict criteria. Intraprocedural echo‐guidance was feasible in 91% of unselected patients. LBBP lead was LV subendocardial in all patients. EC‐LBBP is practical, feasible, safe and highly effective in achieving LBB capture.

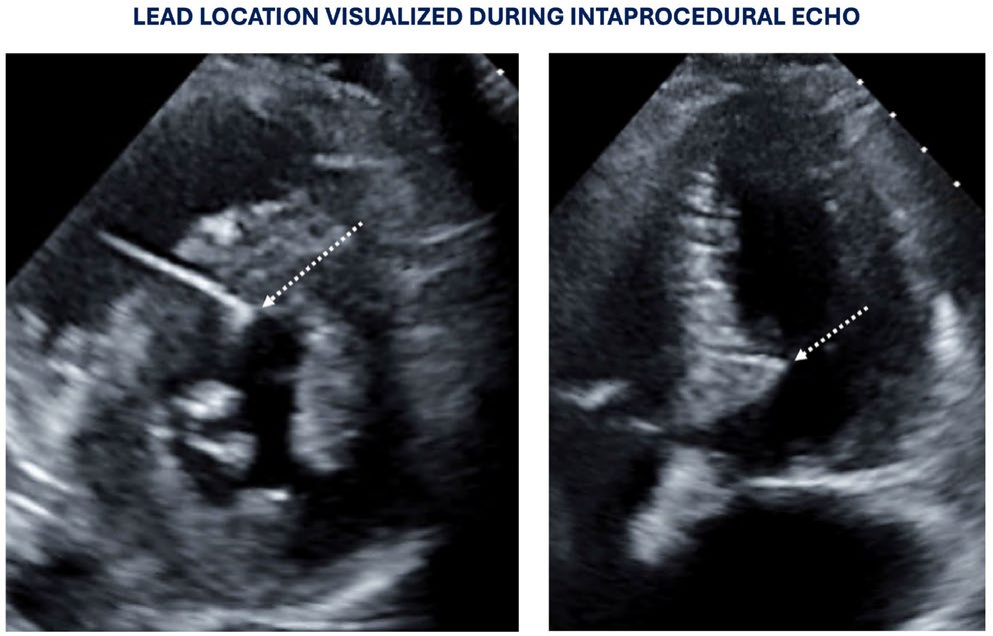

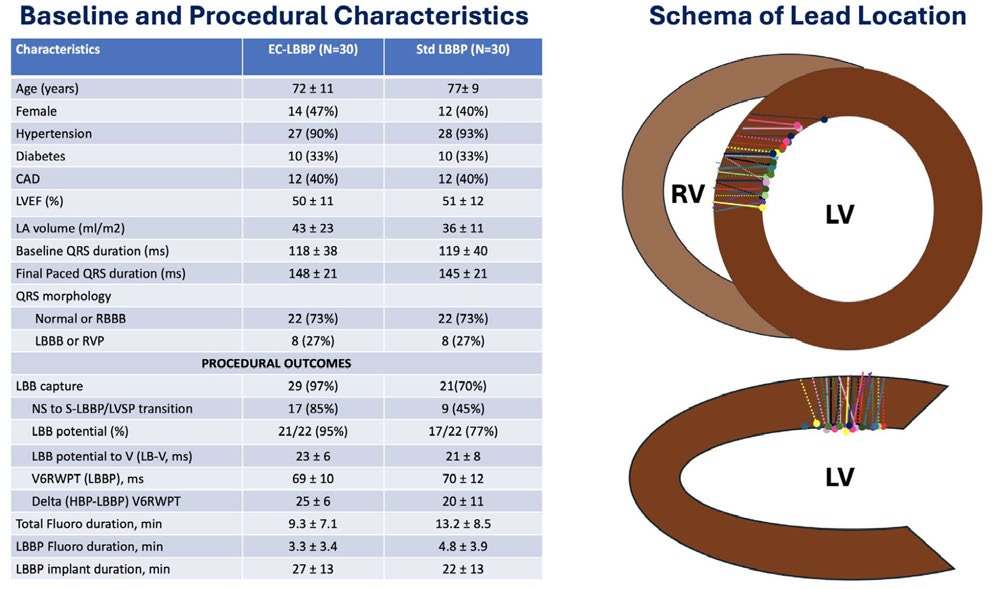

Chair

**J. Vohra**

The Royal Melbourne Hospital, Australia

## 103 LEFT VENTRICULAR SCAR CHARACTERISTICS ON CARDIAC MR IN MAORI AND PACIFIC PATIENTS WITH NON ISCHAEMIC CARDIOMYOPATHY. A SINGLE CENTRE COHORT

### 103.1 **JAMIE VOSS**, ROBERT MICHAEL, RYAN DAVIS, RUVIN GABRIEL, ANDREW KERR, JEN LI LOOI

#### 103.1.1 Middlemore Hospital, Auckland, New Zealand

**Introduction:** The phenotype in patients with non ischaemic cardiomyopathy (NICM) is heterogeneous. The presence of left ventricular (LV) scar as detected by delayed Gadolinium‐enhanced cardiac magnetic resonance (DE‐CMR) is associated with a higher risk of ventricular arrhythmia and scar location has implications for ventricular tachycardia ablation. The characteristics of LV scar detected by DE‐CMR has not be reported in Māori and Pacific patients with NICM.

**Methods:** Consecutive patients undergoing DE‐CMR to investigate NICM over a 5 year period at a single centre (Middlemore Hospital, Auckland, New Zealand) were included. Patients with other forms of heart disease were excluded. Clinical and CMR data were collected prospectively in the ANZACS‐QI CMR registry. Prioritised ethnicity data was obtained via linkage with the National Dataset. The characteristics of LV scar were analyzed by ethnicity.

**Results:** From 2019 to 2023 a total of 330 patients underwent DE‐CMR to investigate NICM. Ethnicity: European (E) 113 pts, Māori (M) 101 pts, Pacific (P) 85 pts, and Other 31 pts. Male 247/330 pts (75%). Age median 54y (range 17‐85y). Median LVEF 40% (IQR 33‐48%). LV scar was present in E 56/113 pts (49.6%), M 57/101 pts (56.4%) and P 35/85 pts (41.2%) (p 0.038 M v P, all others p≥ 0.05). Median LVEF E 41% (IQR 34‐46%), M 37% (IQR 30‐45%), P 35% (IQR 30‐43%). The scar pattern was predominantly septal/anteroseptal in E 30/56 pts (54%), M 31/57 pts (54%), and P 17/35 (49%) (all p≥ 0.05). The scar pattern was predominantly inferolateral/free wall in E 20/56 (36%), M 15/57 (26%) and P 10/35 (29%) (all p≥ 0.05). The median number of segments with scar when present were E 2 (IQR 1‐3), M 3 (IQR 2‐5) and P 3 (IQR 2‐5.5). Transmural scar was seen in at least 1 segment in 9/163 pts (5.5%) with the remainder mid‐myocardial or sub‐epicardial scar.

**Conclusions:** The LV scar pattern on DE‐CMR in pts with NICM was comparable for Māori and Pacific patients with European patients. Māori patients have a higher proportion of presence of any LGE when compared with Pacific patients. This may have implications for ventricular tachycardia ablation.

## 104 IMPACT OF SILENT CEREBRAL EMBOLISMSDURING AND AFTER LEFT ATRIAL APPENDAGE OCCLUSION ON LONG‐TERM COGNITION FUNCTION

### 104.1 **KEXIN WANG** ^1^, ZHE WANG^2^, CAIYI JIN^1^, WEIZHU JU^1^, MINGLONG CHEN^1^

#### 104.1.1 ^1^The First Affiliated Hospital of Nanjing Medical University, Nanjing, China,^2^Nantong First People's Hospital, Nantong, China

**Introduction:** Left atrial appendage occlusion (LAAO) was associated with a high incidence of procedure‐related silent cerebral embolisms (SCE). There is limited data regarding the long‐term cognitive trajectory of patients undergoing LAAO. The aim of our study was to comprehensively assess the acute and long‐term impact of SCE during and after LAAO by frequent follow‐up of cerebral magnetic resonance imaging (MRI) and cognitive assessments.

**Methods:** Consecutive AF patients referred for LAAO between February 2021 and February 2023 were included. All patients underwent MRI and cognitive assessments before and within 48 hours after the procedure. These evaluations were also repeated at 45‐day, 3‐month, 6‐month and 1‐year follow up.

**Results:** Out of 75 patients included in the final analysis, 29 (38.7%) patients suffered from new SCE during LAAO. Patients with SCE exhibited a significant decline in cognitive function (MMSE) immediately after the procedure (*p*<0.001), which was not reversible during 1 year follow‐up (*p*<0.001). Additionally, with time going on, the gap in cognitive function between patients with and without SCE became wider (SCE×1 year: B=‐4.81 [95% CI, ‐5.58 to ‐4.05]; *p*<0.001). New‐onset SCE was detected in 11 (14.7%) patients during the follow‐up MRIs, which also resulted in a decline in cognitive function (*p*=0.004). The results in MoCA scores were consistent with MMSE.

**Conclusions:** LAAO‐related SCE leads to a marked impairment in cognitive function immediately after the procedure and is irreversible over a 1‐year follow‐up. New MR‐detected SCE during the follow‐up after LAAO would also lead to a decline in cognitive function.

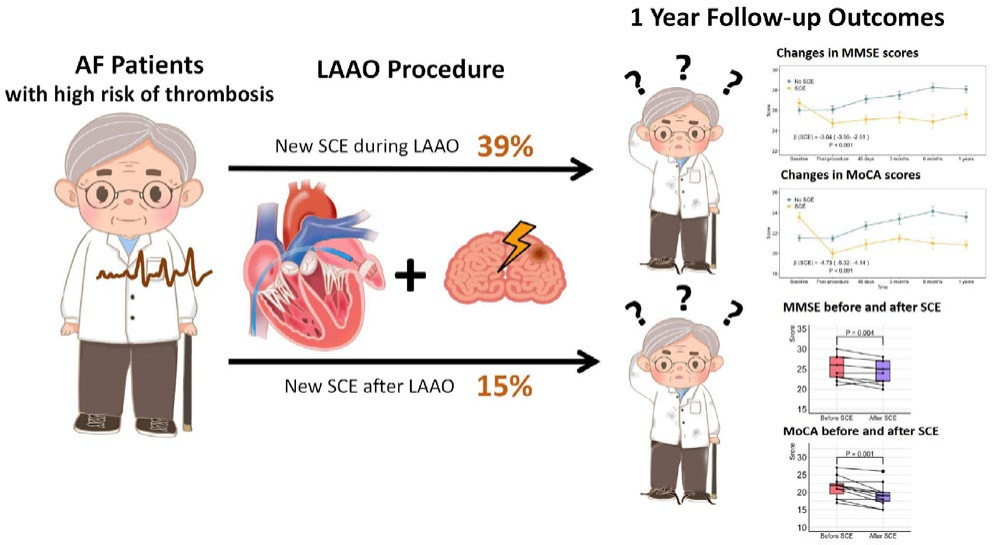

## 105 SUCCESSFUL SUPPRESSION OF PREMATURE VENTRICULAR CONTRACTIONS BY CATHETER ABLATION WITH AN INITIAL ATTEMPT‐‐‐‐IS ONESHOT ENOUGH?

### 105.1 **KEXIN WANG**, WEIZHU JU, MINGLONG CHEN

#### 105.1.1 The First Affiliated Hospital of Nanjing Medical University, Nanjing, China

**Introduction:** Recurrence remains a challenge after ablation of outflow tract premature ventricular complexes (OT‐PVCs). While adding additional lesions next to the index effective ablation site is sometimes employed to reinforced the ablation, it remained uncertain whether this approach truly works or merely serves as a “psychological reassurance” for the operators. This study aimed to test the hypothesis that additional ablation would reduce the recurrence rate compared with single‐point ablation at the index effective site for ablation of OT‐PVCs.

**Methods:** This study is a multi‐center, prospective randomized controlled trial. Patients receiving their first catheter ablation for OT‐PVCs were enrolled from 18 hospitals in China between October 2021 and February 2023. After identifying the target point and eliminating the PVC by a single‐point ablation, patients were randomized 1:1 into additional ablation group or control group. Scheduled follow‐up was conducted 3 months after the procedure. The primary endpoint of the study was freedom from PVC recurrence (≥ 80% reduction of PVC burden).

**Results:** Of 308 patients enrolled in the study, 286 were randomized into additional ablation and the control groups, while 22 were included in an observational group. After a median follow‐up of 3.2 ± 0.7 months, freedom from PVCs was significantly higher in the additional ablation group (139/142, 97.9%) compared with the control group (115/13, 82.7%) and observational group (18/22, 81.8%, *P* < 0.001). Patients in the additional ablation group also had a more significant reduction in PVC burden than the control group (23.0% *vs*. 19.0%, *P* < 0.001). In the observational group, 81.8% of the patients were free from PVCs and the decrease in PVC burden was 17.5%. There were no severe peri‐procedural complications.

**Conclusions:** This trial proved the superiority of additional ablation in reducing the recurrence of OT‐PVCs compared with the single‐point ablation strategy. Given the efficacy and safety of this approach, additional ablations surrounding the “bull's eye” should be routinely used in OT‐PVC ablation.

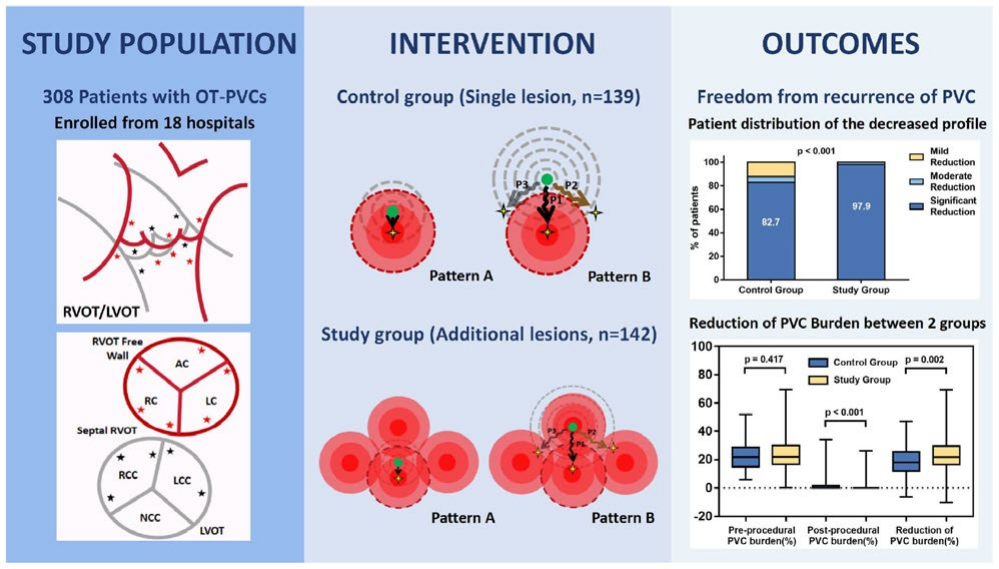

## 106 A SYSTEMATIC REVIEW AND META‐ANALYSIS ASSESSING BENEFITS OF CATHETER ABLATION FOR VENTRICULAR TACHYCARDIA IN PEOPLE WITH STRUCTURAL HEART DISEASE WHO ARE RECEIVING IMPLANTABLE CARDIOVERTER‐DEFIBRILLATOR

### 106.1 **YOGA WARANUGRAHA** ^1,2,3^, MUHAMMAD YUSUF^1^, KRISANTO TANJAYA^1^

#### 106.1.1 ^1^Universitas Brawijaya, Malang, Indonesia,^2^Universitas Brawijaya Hospital, Malang, Indonesia,^3^Persada Hospital, Malang, Indonesia

**Introduction:** Individuals diagnosed with structural heart disease (SHD) have an increased susceptibility to experiencing ventricular tachycardia (VT). Catheter ablation has the potential to decrease this risk. This systematic review and meta‐analysis study aimed to compare catheter ablation with medical therapy for managing VT in patients with SHD receiving implantable cardioverter‐defibrillator (ICD).

**Methods:** A comprehensive search for published papers that investigated the impact of catheter ablation on patients with SHD who were also receiving the ICD was conducted. This literature search process was conducted until March 2024 and included online scientific databases such as Cochrane, Open MD, ProQuest, PubMed, and ScienceDirect. The outcomes of interest assessed were recurrent VT or ventricular fibrillation, VT storm, and appropriate ICD shock. Risk ratio (RR) and 95% confidence intervals (CI) were estimated using a random‐effects model for each outcome. Furthermore, meta‐regression was performed to explore changes in outcome differences over time, with the year of publication serving as the moderator.

**Results:** A total of 11 studies were included. Of the total 1372 participants included, 591 participants received VT ablation, and 781 participants did not receive VT ablation. Catheter ablation provided more benefit in reducing the recurrent VT or VF (RR = 0.72; 95% CI = 0.55 to 0.96; p = 0.02), VT storm (RR = 0.72; 95% CI = 0.54 to 0.95; p = 0.02), and appropriate ICD shock (RR = 0.54; 95% CI = 0.35 to 0.84; p = <0.01). Meta‐regression showed a gradual decrease over time for the recurrent VT/VF rate after the catheter ablation procedure. A 1‐year increase in publication year was correlated with a ‐5.59% (95% CI = ‐9.66% to ‐1.52%; p <0.01) reduction or recurrent VT/VF.

**Conclusions:** Catheter ablation for VT in SHD patients was associated with a reduced risk of recurrent VT or VF, VT storm, and appropriate ICD shock. The efficacy of catheter ablation has improved with time, as evidenced by a gradual reduction in the recurrence rate.

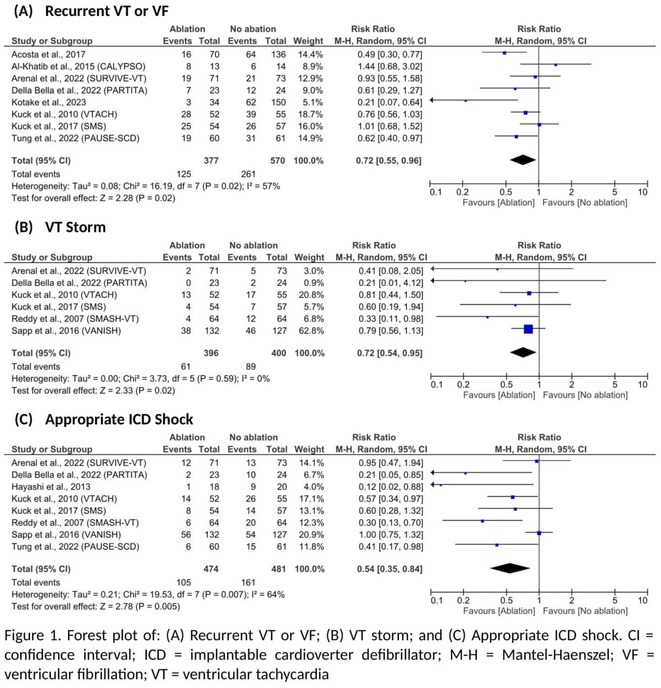

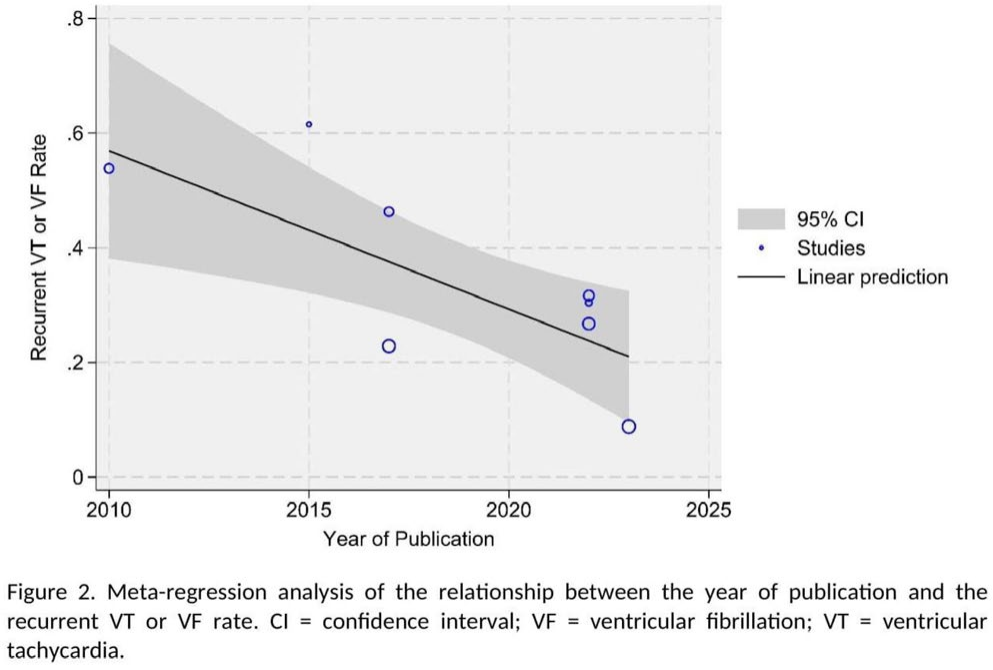

## 107 FOCAL MYOCARDIAL FIBROSIS ASSOCIATES WITH ADVERSE ELECTROPHYSIOLOGICAL PHENOTYPES: THE MYOFIT46 STUDY

### 107.1 SWAPNANIL DE^1^, ISABELLE WHITMORE^1^, NIKHIL KADAMBADI^1^, **MATTHEW WEBBER** ^1,2^, ALI ARDISSINO^1^, GEORGE JOY^1,2^, JONATHAN BENNETT^1,2^, FIONA CHAN^1,2^, ABHISHEK VIGNESH KUMAR^1^, DEBBIE FALCONER^3^, HUNAIN SHIWANI^1,2^, PABLO GONZALEZ MARTIN^1,4,5^, RHODRI H DAVIES^1,2^, EMMA MARTIN^6^, MARTIN UGANDER^7,8^, IAIN PIERCE^1,2,6^, REBECCA HARDY^1,6^, NISHI CHATURVEDI^6^, ALUN D HUGHES^1,6^, PETER KELLMAN^9^, JAMES C MOON^1,2^, PIER LAMBIASE^1,2^, TODD SCHLEGEL^7,10^, GABRIELLA CAPTUR^1^

#### 107.1.1 ^1^Institute of Cardiovascular Science, University College London, London, United Kingdom,^2^Barts Heart Centre, Barts Health NHS Trust, London, United Kingdom,^3^Centre for Inherited Heart Muscle Conditions, Department of Cardiology, Royal Free London NHS Foundation Trust, London, United Kingdom,^4^ELEM Biotech S.L., Barcelona, Spain,^5^Barcelona Supercomputing Center, Barcelona, Spain,^6^Medical Research Council Unit for Lifelong Health and Ageing, UCL, London, United Kingdom,^7^Department of Clinical Physiology, Karolinska University Hospital and Karolinska Institutet, Stockholm, Sweden,^8^Kolling Institute, Royal North Shore Hospital and University of Sydney, St Leonards, Sydney, Australia,^9^National Heart, Lung, and Blood Institute, Bethesda, MD,^10^Nicollier‐Schlegel SARL, Trélex, Switzerland

**Introduction:** Focal myocardial fibrosis has been associated with standard 12‐lead electrocardiographic (ECG) measures but little is known about its association with advanced ECG (A‐ECG) scores. We investigated the association between left ventricular (LV) scar and A‐ECG scores of LV systolic dysfunction (LVSD) and electrical remodelling (LVER) in a population‐based older age cohort.

**Methods:** Cardiovascular magnetic resonance with a single 3 Tesla magnet was performed on participants of the 1946 British birth cohort (the National Survey of Health and Development). A free‐breathing motion‐corrected balanced steady‐state free precession sequence with phase‐sensitive inversion‐recovery provided LV short‐axis stack late gadolinium enhancement (LGE) data that was quantified according to the American Heart Association 17‐segment model using ADAS 3D and the 5 standard deviation method (Figure 1). Univariate and multivariable linear regressions explored the association between LGE mass and the LVSD/LVER scores derived using various ECG measures (with spatial mean QRS‐T angle being key score determinants). Analyses were also conducted to study the association of LGE mass and parameters within the LVER score.

**Results:** 191 participants (49.8% male, 76.5±0.7 years, 1.55±3.28g LGE) with both LGE data and A‐ECG scores were studied. On univariate analyses, LGE mass was associated with worse (i.e. smaller) A‐ECG scores (both *p*<0.001), and most strongly with LVER (*p*<0.001, β = ‐1.07, 95% CI ‐1.65 to ‐0.59). On multivariable analysis, these associations persisted (both *p*<0.001) after adjusting for sex, socioeconomic position, body mass index, hypertension, diabetic status, and cigarette smoking. Of the measures within the LVER score, LGE mass associated with a higher spatial mean QRS‐T angle (*p*<0.001, β = 3.54, 95% CI 2.11 to 4.97) and lower QRS voltage (*p*<0.001, β = ‐0.82, 95% CI ‐1.30 to ‐0.34).

**Conclusions:** In an older‐age cohort, focal myocardial scar associates with adverse electrophysiological phenotypes, including a heterogeneity of the myocardial ventricular action potential duration and lower signal amplitudes.

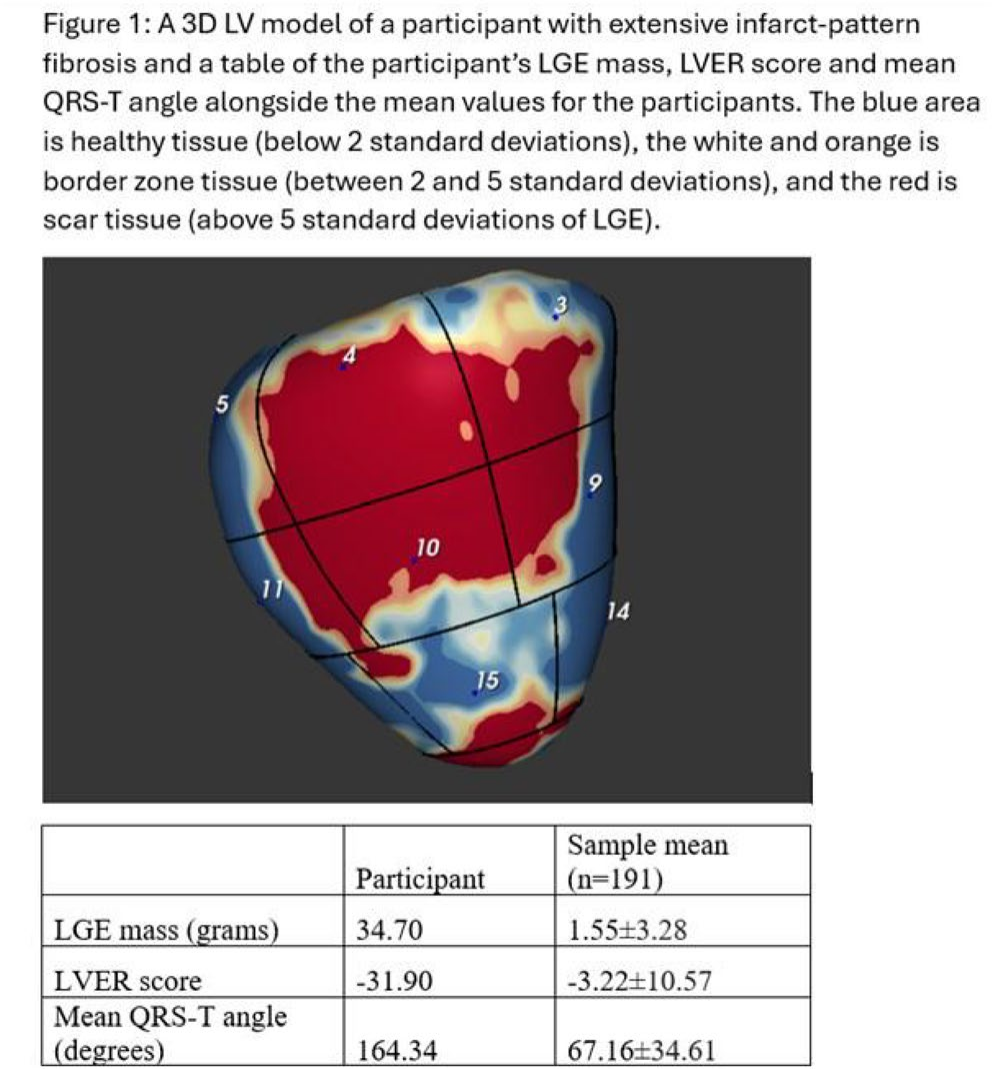

## 108 CATHETER ABLATION IN PERSISTENT ATRIAL FIBRILLATION: LONG TERM OUTCOMES OF THE CAPLA MULTICENTER RANDOMIZED TRIAL OF PULMONARY VEIN ISOLATION (PVI) VS PVI PLUS POSTERIOR LEFT ATRIAL WALL ISOLATION (PWI)

### 108.1 **JEREMY WILLIAM** ^1^, ANOUSHKA IYER^1^, DAVID CHIENG^1^, ANNIE CURTIN^1^, ROSE CROWLEY^1^, LOUISE SEGAN^1^, HARIHARAN SUGUMAR^1^, LIANG HAN LIGN^1^, SANDEEP PRABHU^1^, ALEKSANDR VOSKOBOINIK^1^, JOSEPH MORTON^2^, GEOFF LEE^2^, ALEX MCLELLAN^2^, RAJEEV PATHAK^3^, LAURENCE STERNS^4^, MATTHEW GINKS^5^, CHRISTOPHER REID^6^, PRASHANTHAN SANDERS^7^, JONATHAN KALMAN^2^, PETER KISTLER^1^

#### 108.1.1 ^1^Alfred Health, Melbourne, Australia,^2^Melbourne Health, Melbourne, Australia,^3^Canberra Heart Rhythm Centre, Canberra, Australia,^4^Royal Jubilee Hospital, Victoria, BC, Canada,^5^Oxford University Hospital, Oxford, United Kingdom,^6^Curtin University, Perth, Australia,^7^Royal Adelaide Hospital, Adelaide, Australia

**Introduction:** Posterior wall isolation (PWI) is commonly incorporated into catheter ablation (CA) strategies with persistent atrial fibrillation (AF) in an attempt to improve outcomes. In the CAPLA randomized study, adjunctive PWI did not improve freedom from atrial arrhythmia at 12 months compared with pulmonary vein isolation (PVI) alone. Whether additional PWI reduces arrhythmia recurrence over long‐term follow‐up remains unknown.

**Methods:** In this multicenter, international, randomized study patients with persistent AF undergoing index CA using RF were randomized to PVI+PWI versus PVI alone. Patients underwent regular follow‐up including rhythm monitoring for a minimum of 3 years post CA. The primary endpoint was freedom from any documented atrial arrhythmia recurrence after a single procedure. Secondary outcomes included AF burden, need for redo catheter ablation, rhythm at last clinical follow‐up, healthcare utilization metrics and AF‐related quality of life.

**Results:** 333 of 338 (98.5%) patients (mean age 64.3±9.4 years, 23% female) were included, with 169 patients randomized to PVI+PWI and 164 patients to PVI alone. At a median of 3.62 years post‐index ablation, freedom from recurrent atrial arrhythmia occurred in 59 patients (35.5%) randomized to PVI+PWI versus 68 patients (42.1%) randomized to PVI alone (HR 1.15, 95% CI 0.88‐1.51, p=0.55). Redo ablation was performed in 54 patients (32.0%) in the PVI+PWI group vs 49 patients (29.9%, p=0.68) in the PVI alone group. Median AF burden at 3 years was 0% in both groups (IQR 0‐0.85% PVI+PWI vs 0‐1.43% PVI alone, 0.49. Mean AF Effect On Quality‐Of‐Life (AFEQT) score at 3 years post‐ablation was 88.0±14.8 with PVI+PWI versus 88.9±15.4 with PVI alone (p=0.63).

**Conclusions:** In patients with persistent AF, the addition of PWI to PVI alone at index RF catheter ablation did not significantly improve freedom from atrial arrhythmia recurrence at long‐term follow‐up. Median AF burden remains low and AF quality of life high at 3 years with either ablation strategy.

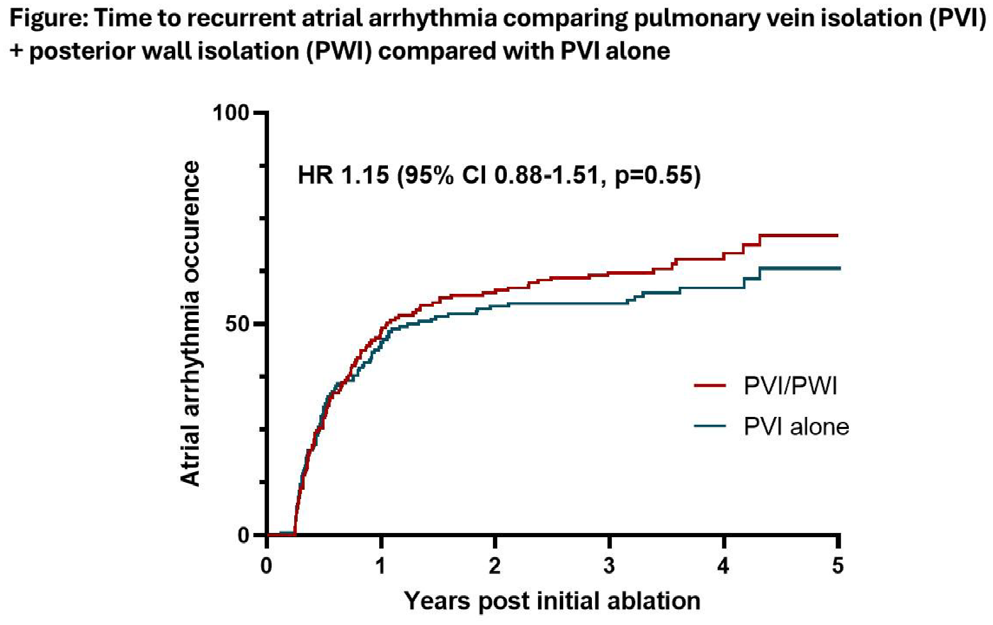

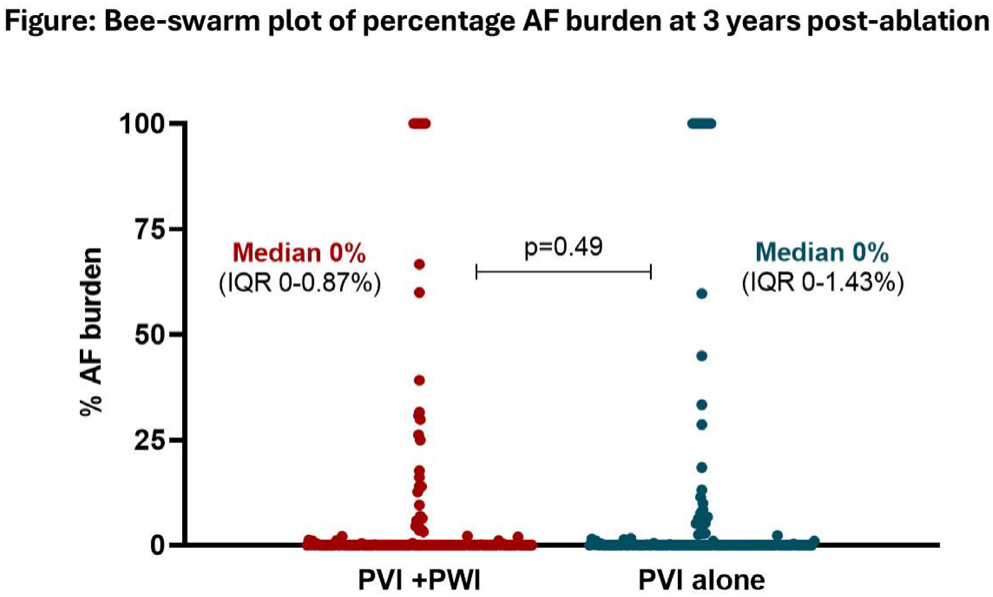

## 109 PREVALENCE OF CARDIAC FIBROSIS AND INFILTRATIVE CARDIOMYOPATHY IN PATIENTS WITH ADVANCED CONDUCTION SYSTEM DISEASE

### 109.1 **JEREMY WILLIAM**, HAIDER MUTHANNA, JOSEPH HOGARTY, JAMES HARE, HITESH PATEL, DAVID KAYE, ANDREW TAYLOR, SANDEEP PRABHU, PETER KISTLER, ALEKSANDR VOSKOBOINIK

#### 109.1.1 Alfred Health, Melbourne, Australia

**Introduction:** Conduction system disturbance may represent an early manifestation of underlying structural heart disease, including infiltrative disorders such as sarcoidosis. Timely diagnosis has significant implications for clinical management, allowing for disease‐modifying therapy or ICD implantation.

**Methods:** We evaluated all patients undergoing CMR between 2005‐2023 at The Alfred for investigation of advanced distal conduction system disease (complete heart block, Mobitz II 2nd degree AV block or bifascicular block). We excluded patients with known systolic heart failure (LVEF<50%) at time of CMR evaluation. We sought to determine the prevalence of CMR‐detected myocardial fibrosis and infiltrative cardiomyopathy in this cohort.

**Results:** 119 patients were identified (mean age 49±15 years, 52% male). Complete heart block was the most common subtype of conduction disease (50%), followed by bifascicular block (27%) and Mobitz II block (23%). Mean LVEF on TTE prior to CMR evaluation was 60.0±3.1%. Overall, CMR‐detected LGE was present in 32/119 patients (26.9%). LGE positive patients exhibited higher average LV mass index (64.1±19.6 vs 56.3±16.2g/m2, p=0.03) and a trend towards lower LVEF (56.1±8.5 vs 59.5±8.4%, p=0.05). Cardiac sarcoid was the most common final diagnosis (n=19, 16%), of whom only 6 (26%) had known extracardiac sarcoid prior to CMR. Cardiac sarcoidosis observed in a similar proportion of patients across the three subtypes of conduction disease studied (p=0.30)

**Conclusions:** Cardiac fibrosis is present in a substantial proportion of patients undergoing CMR for the investigation of conduction disease. Cardiac MRI may be an important adjunctive tool for investigation of conduction disease, particularly in younger patients.

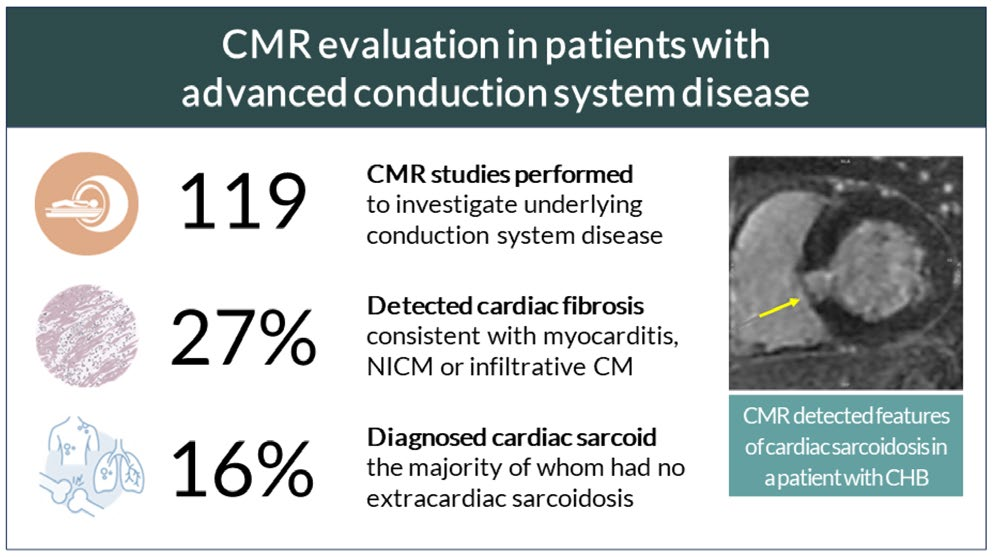

## 110 PREVENTING ATRIAL FIBRILLATION RECURRENCE WITH COMBINATION OF CATHETER ABLATION AND RENAL DENERVATION OR GANGLION PLEXUS ABLATION: A NETWORK META‐ANALYSIS

### 110.1 **SEBASTIAN WILLYANTO** ^1^, LILIANA DEWI^1^, RIZKI MULIA^1^, IMKE PULING^1^, NYOMAN GIRI^1^, ARDIAN RIZAL^2^, DERREN RAMPENGAN^3^, MELISSA ARIYANTO^4^

#### 110.1.1 ^1^Brawijaya University Faculty of Medicine, Malang, Indonesia,^2^Brawijaya Cardiovascular Research Center, Department of Cardiology and Vascular Medicine, Faculty of Medicine, Universitas Brawijaya, Malang, Indonesia,^3^Sam Ratulangi University Faculty of Medicine, Manado, Indonesia,^4^Airlangga University Faculty of Medicine, Surabaya, Indonesia

**Introduction:** Atrial fibrillation (AF), affects around 2% of the global population and is projected to rise over the next 50 years. Catheter ablation (CA) is the primary treatment for symptomatic AF resistant to drug therapy. Despite its widespread use, CA has a failure rate of 20% to 50%, often requiring repeat procedures, due to significant long‐term recurrence rates. Combining CA with renal denervation (RDN) or ganglion plexus ablation (GPA) may effectively reduce the recurrence rates of AF.

**Methods:** Quality assessment was done using the Cochrane ROB 2.0 tool, network meta‐analysis using RStudio, and comparative meta‐analysis using RevMan 5.4.

**Results:** A thorough search across seven databases resulted in 13 articles for analysis, with eight classified as low‐risk and five as moderate‐risk of bias. The network meta‐analysis found that RDN + CA had the highest freedom from AF episodes at 12 and 24 months (odds ratio [OR] = 2.28; 95% CI = 1.34, 3.86 and OR = 1.61; 95% CI = 0.89, 2.89), followed by GPA + CA (OR = 1.88; 95% CI = 0.91, 3.89 and OR = 1.36; 95% CI = 0.91, 2.03), compared to CA alone. RDN + CA also showed fewer procedure‐related complications (OR = 0.78; 95% CI = 0.30, 2.02), while GPA + CA was more prevalent (OR = 3.60; 95% CI = 1.72, 7.55), compared to CA alone. Additionally, RDN + CA significantly reduced systolic blood pressure (SBP) (MD = ‐5.22; 95% CI = ‐9.91, ‐0.53), diastolic blood pressure (DBP) (MD = ‐3.61; 95% CI = ‐7.98, ‐0.76), and creatinine levels (MD = ‐0.25; 95% CI = ‐0.34, ‐0.15), while increasing estimated glomerular filtration rate (eGFR) (MD = 7.98; 95% CI = ‐1.16, 17.11).

**Conclusions:** Remarkable success in preventing AF recurrence was observed when CA was combined with RDN or GPA. However, it is noteworthy that GPA + CA was associated with a higher incidence of procedural‐related complications, while RDN + CA demonstrated additional advantages by improving blood pressure regulation and renal function.

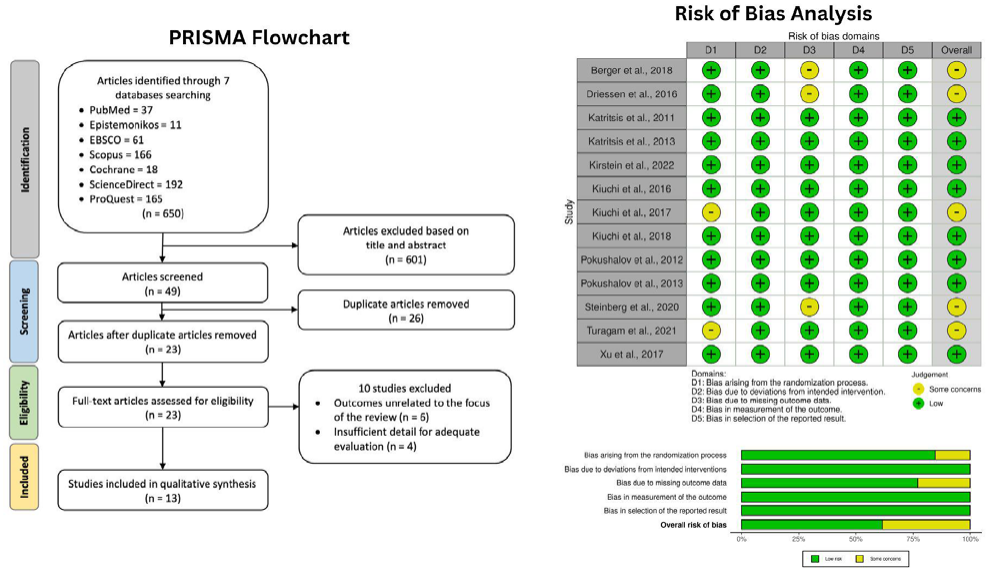

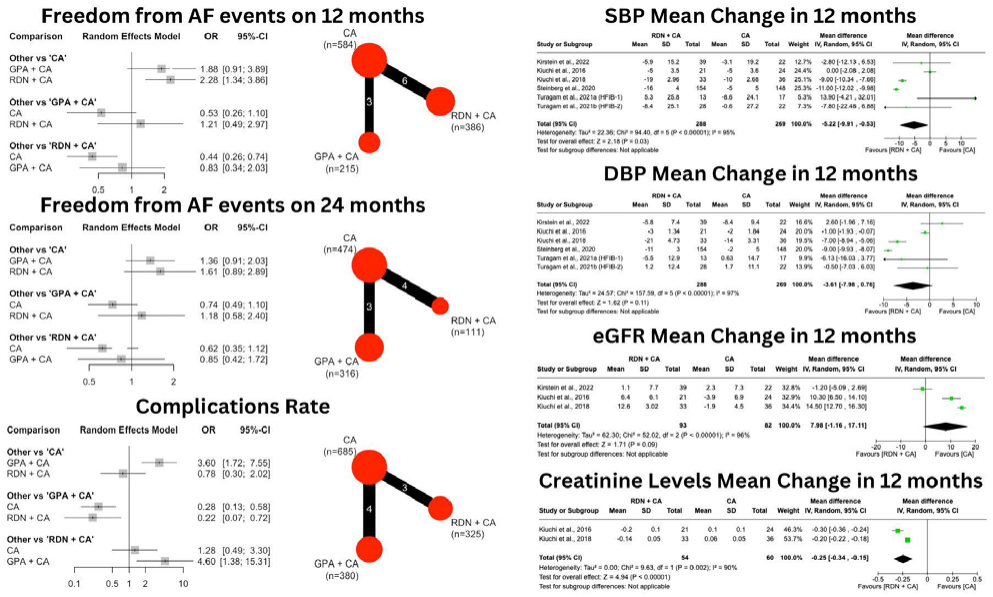

## 111 ANTI‐FACTOR XA ACTIVITY OF BRANDED GENERIC APIXABAN IN EXTREMELY LOW BODY WEIGHT

### 111.1 **WANWARANG WONGCHAROEN**, AMARASE PAMARAPA, ARINTAYA PHROMMINTIKUL

#### 111.1.1 Chiang Mai University, Chiang Mai, Thailand

**Introduction:** The efficacy and safety of direct oral anticoagulants (DOACs) are still concerns for patients with extremely low body weight (BW ≤ 50 kg). We aimed to compare the anti‐factor Xa activity (anti‐FXa) of apixaban between patients with normal BW (>50 kg) and those with low BW (≤50 kg).

**Methods:** We enrolled patients who were receiving branded generic apixaban, Apixan^TM^, therapy due to atrial fibrillation (AF), venous thromboembolism (VTE), and intracardiac thrombus. The anti‐factor Xa (anti‐FXa) activity of apixaban was measured at peak and trough concentrations.

**Results:** A total of 150 patients were included in the study, in whom 132 (88%) patients had AF. The mean age was 64.0±12.7 years, with males comprising 53.3%. The mean body weight (BW) was 61.3±15.3 kg. There were 43 patients (28.7%) who had a BW ≤ 50 kg. Overall, 90.7% and 87.3% of patients had trough and peak plasma levels within the expected range, respectively. Interestingly, mean trough and peak anti‐factor Xa (anti‐FXa) activity of apixaban were significantly higher in the low BW group compared to normal BW group (123.8±70.3 vs. 94.7±47.0 mcg/L and 232.0±123.3 vs. 177.0±75.8 mcg/L, respectively, P=0.007 for both). The low BW group had a higher proportion of patients with anti‐FXa activity at peak concentrations exceeding the expected range than the normal BW group (25.6% vs. 3.7%, p < 0.001). After multivariate analysis adjusting for potential covariates, low BW was the only independent predictor associated with the exceeding level of peak concentrations of apixaban (adjusted OR 4.87, 95% CI 1.31‐18.15, p = 0.018).

**Conclusions:** The patients with low BW were associated with a higher risk of having peak anti‐factor Xa (anti‐FXa) activity of apixaban exceed the expected range. Care should be taken when prescribing apixaban in patients with low BW.

## 112 DOUBLE CAPTURE TEST ‐ A TECHNIQUE TO DIFFERENTIATE NARROW COMPLEX SUPRAVENTRICULAR TACHYCARDIAS

### 112.1 **ELIZABETH WOOLLARD** ^1^, TIMOTHY RYAN^1^, DAVID YUN^1^, NIKOLA STOYANOV^1^, VINCENT PAUL^1^, RAFEEQ SAMIE^1^, ANNE POWELL^1^, TIMOTHY GATTORNA^1^, KRISHNAKUMAR NAIR^2^, BENJAMIN KING^1^

#### 112.1.1 ^1^Fiona Stanley Hospital, Perth, Australia,^2^Toronto General Hospital, Toronto, ON, Canada

**Introduction: Background**There are various diagnostic manoeuvres to distinguish between atrial tachycardia (AT), atrio‐ventricular nodal re‐entrant tachycardia (AVNRT) and orthodromic re‐entrant tachycardia (ORT) when assessing a narrow complex supraventricular tachycardia (SVT) in the electrophysiology (EP) laboratory. These manoeuvres are commonly used in combination to come to a diagnosis due to the inability of a single test to reliably differentiate between the arrhythmias.

**Objective**To determine whether a single captured His‐synchronous simultaneous extra‐stimulus in the atrium and ventricle (“Double Capture”) can reliably distinguish the mechanism of a narrow complex SVT.

**Methods:** At a single institution, we reviewed the data of patients with a narrow complex SVT in whom this maneuver was performed as part of their routine electrophysiology study, and analyzed the intracardiac recordings. If the simultaneous extra‐stimuli was delivered when the His was refractory and captured both the atrium (A) and ventricle (V), the earliest signal was assessed to attempt to differentiate the mechanism.

**Results:** Double Capture was attempted in 44 patients of which six were excluded due to incorrect timing or inadequate electrogram recordings. Of the 38 remaining patients who either had AVNRT or ORT (no ATs were included), “Double Capture” was achieved in 29 patients (76%). In patients in whom “Double Capture” occurred, reproducible termination of the tachyarrhythmia with “Double Capture” corresponded with ORT (n=7). In patients with “Double Capture” with a His signal as the first signal post, and ongoing tachycardia, this typically corresponded with a diagnosis of AVNRT (n=20), though there were two exceptions with ORT (n=2).

**Conclusions:** In this small study, “Double Capture” of the A and V during a sustained narrow complex SVT without change to the tachycardia or His interval timings was able to confirm AVNRT with a specificity of 78% and ORT with a specificity of 100%. This maneuver may be especially helpful to confirm bystander pathways or assess septal pathways.

## 113 MTORC1 SIGNALING IS CRUCIAL FOR THE MAINTENANCE OF SINOATRIAL NODE FUNCTION

### 113.1 **YUN XIE**, GUANHUA WU, YANGYANG BAO, QINGZHI LUO, CHANGJIAN LIN, YUE WEI, NING ZHANG, QI JIN, LIQUN WU

#### 113.1.1 Ruijin Hospital, Shanghai Jiaotong University School of Medicine, Shanghai, China

**Introduction:** Sinoatrial node (SAN) dysfunction is the common cause of bradycardia in the elderly, which may induce cardiac arrest, syncope and even sudden death. Despite its importance, the mechanism of pathogenesis remains largely unknown. mTORC1 signaling, which integrates growth factor and nutrients signals, plays a key role in cellular energy metabolism and function maintenance. However, its role in sinoatrial node pacemaker cells has not been well studied.

**Methods:** We generated cardiac conduction cell‐specific inducible Raptor knockout mice (cRapKO) by mating Raptor^lox/lox^ mice with HCN4‐CreERT2 mice. After 1 week of tamoxifen injection, the mice were tested for their sinus rhythm through resting electrocardiogram (ECG) and telemetry recordings. Mice were then sacrificed to obtain SAN tissue to confirm the inhibition of mTORC1 pathway and to process single‐cell RNA‐sequencing (sc‐RNAseq).

**Results:** Resting heart rate of cRapKO mice is significantly decreased compared to the control mice. ECG of freely moving mice by telemetry recording demonstrated that cRapKO mice gradually developed bradycardia in both light or dark phases after 36 hours of tamoxifen injection. PR interval and QRS duration were not affected in cRapKO mice, while its cardiac function remained normal. Through western blot and immunofluorescence study, we found that phospho‐S6 was down‐regulated in SAN tissue and HCN4‐positive cells, suggesting successful inhibition of mTORC1 signaling in target tissue. No significant difference of sinus nodal fibrosis was detected between cRapKO and control mice. To further investigate the mechanism of SAN dysfunction in cRapKO mice, we performed sc‐RNAseq of SAN tissue. Using specific marker of pacemaker cells, we determined the cluster of these cells and further compared its transcriptome in cRapKO and control mice. A series of genes involved in mitochondria structure and respiratory chain function was found to be dysregulated, as well as several genes encoding key ion channels related to pacemaking activity.

**Conclusions:** mTORC1 signaling is crucial for the regulation of sinoatrial node function, possibly through its impact on energy metabolism.

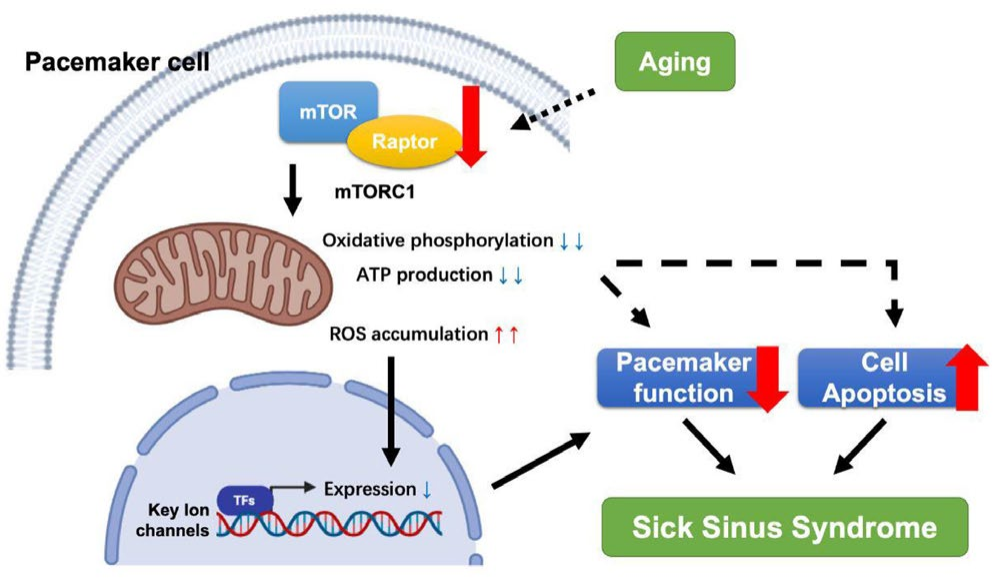

## 114 ASSOCIATION OF LEFT ATRIAL DIVERTICULUM WITH ADVERSE EVENTS OF CATHETER ABLATION FOR ATRIAL FIBRILLATION

### 114.1 **KOKI YAMAOKA**, SEIJI TAKATSUKI, SYUHEI YANO, YUKIHIRO HIMENO, SHYUHEI YAMASHITA, SUSUMU IBE, TAKAHIKO NISHIYAMA, YOSHINORI KATSUMATA, TAKEHIRO KIMURA, MASAKI IEDA

#### 114.1.1 Keio University School of Medicine, Tokyo, Japan

**Introduction:** Although there are several reports investigating left atrial diverticulum (LAD), its characteristics remain unclear. It has been suggested that LAD is associated with complications in catheter ablation for atrial fibrillation (AF), but there are few reports that have been examined.

**Methods:** Among patients who underwent catheter ablation for AF at a single center between December 4, 2008, and February 22, 2024, contrast extravasation was observed in 3 patients during pulmonary venography performed during the procedure. Since all three patients had LAD on preoperative CT, we initially investigated the frequency of contrast extravasation by pulmonary venography. Additionally, we retrospectively evaluated 595 patients with preoperative CT, aiming to clarify the prevalence and location of LAD, and examined the association between the presence of LAD and patient characteristics.

**Results:** Pulmonary venography was performed during catheter ablation procedures in 3442 cases/2867 patients during the study period, and contrast extravasation was observed in 3 cases/3 patients of them (incidence rate 0.09%). In all three cases, preoperative CT showed LAD in the anterior right superior region of the left atrium near the right superior pulmonary vein (RSPV). A retrospective analysis of preoperative CT from 595 cases (average age 65.1 ± 10.2 years, female 17.5%, paroxysmal AF 47.2%, CHA2DS2‐VASc score 0.93 ± 0.12) revealed a total of 266 LADs in 210 cases (prevalence 35.3%), with a predominant location in the anterior right superior region (53.4%) comparable to the extravasation cases. Comparison of patient backgrounds based on the presence of LAD showed no statistically significant differences in age, sex, BMI, AF type, prior stroke, CHA2DS2‐VASc score, and echocardiographic parameters including left atrial diameter.

**Conclusions:** The cases with LAD are more frequent than clinicians imagine, posing a potential risk of complications during catheter manipulation in the surrounding area. Recognizing the presence of LAD on preoperative CT can contribute to fewer complications.

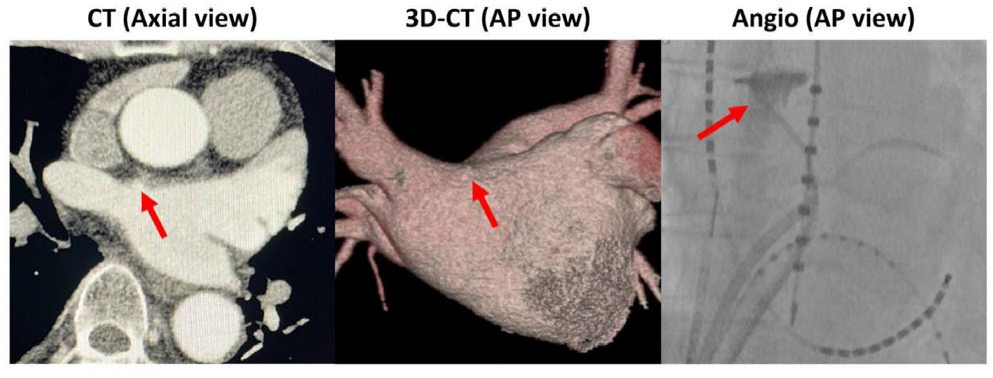

## 115 ANALYSIS OF TYPES AND PROGNOSIS OF INCIDENTALLY DISCOVERED LONG QT SYNDROME: INSIGHTS FROM A KOREAN MULTICENTER COHORT

### 115.1 **JIHYE YOU** ^1^, JUNE HUH^2^

#### 115.1.1 ^1^Jeonbuk National University Hospital, Jeonju, Korea, Republic of,^2^Samsung Medical Center, Sungkyunkwan University School of Medicine, Seoul, Korea, Republic of

**Introduction:** Long QT Syndrome (LQTS) is often discovered incidentally during routine electrocardiogram (ECG) screenings, presenting challenges in management and prognosis. Understanding the types and outcomes of incidentally discovered LQTS is crucial for improving patient care. This study provides insights into the clinical significance of incidentally discovered LQTS in a Korean multicenter cohort.

**Methods:** This study involved 223 patients diagnosed with LQTS through routine ECG screenings in a Korean multicenter cohort. Data on symptoms, genetic mutations, QTc intervals, underlying diseases, family history, and treatment were collected. Genetic testing identified variants of uncertain significance (VUS) in LQTS‐associated genes: KCNQ1, KCNH2, and SCN5A. QTc intervals were measured at diagnosis, and follow‐up assessed survival rates and medication impact.

**Results:** Among the 223 patients, 86 (38.6%) were asymptomatic at diagnosis. Among the asymptomatic, 68 (79.1%) had VUS in genetic mutations: 60.3% KCNQ1, 14.7% KCNH2, and 11.8% SCN5A. KCNQ1 mutations were more common in the asymptomatic group (47.7%) than in the symptomatic group (30.7%, p=0.002). The mean QTc interval for asymptomatic patients was approximately 508 ms. Of the 86 asymptomatic patients, 64 (74.4%) had no underlying disease, and 59 (68.6%) had a family history of cardiac conditions. The average QTc interval was 451.94 ms for patients on medication and 416.79 ms for those not on medication (p=0.015). The overall 5‐year event‐free survival rate was 92.3%, with a higher rate for those on medication (96.2%) compared to those not on medication (81.7%, p=0.002).

**Conclusions:** This study highlights the importance of genetic profiling and medication in improving prognosis and event‐free survival among asymptomatic LQTS patients. Personalized management strategies based on genetic and clinical data are essential for enhancing patient outcomes. Regular follow‐up and appropriate medication significantly improve prognosis, ensuring better long‐term health and reduced risk of adverse cardiac events.

Chair

**J. C. Zerpa**

HCor ‐ Hospital do Coracao, Sao Paulo, Brazil

## 116 CHARACTERIZATION OF A NOVEL*SLC4A3*VARIANT IDENTIFIED IN A SHORT QT SYNDROME FAMILY

### 116.1 **MING ZHU** ^1^, QI WANG^2^, MINORU HORIE^3^, MASAHITO OGAWA^1^, KAZU KIKUCHI^1^, SEIKO OHNO^1^

#### 116.1.1 ^1^National Cerebral and Cardiovascular Center, Osaka, Japan,^2^China Medical University, Shenyang, China,^3^Shiga University of Medical Science, Shiga, Japan

**Introduction:** Short QT syndrome (SQTS) is one of the lethal inherited arrhythmia syndromes, and it is mainly caused by pathogenic variants in *KCNH2*, which produces *I*_Kr_. Recently, *SLC4A3*, which encodes a membrane‐localized anion exchanger 3 (AE3) that regulates intracellular pH (pHi), has been reported as a causative gene for SQTS. However, the molecular mechanism of *SLC4A3* variants that cause SQTS remains unclear. Here, we aimed to elucidate the pathogenesis of a novel *SLC4A3* variant identified in a SQTS family.

**Methods:** We performed target gene and whole exome sequencing to detect pathogenic variants in a family with SQTS. Patch‐clamp analyses were performed to determine the electrophysiological properties using HEK293 cells expressing wild‐type (WT) or variant *SLC4A3*. We measured intracellular pH (pHi) by BCECF‐AM via time‐lapse microscopy. To clarify the pathogenesis of the *SLC4A3* variant *in vivo*, we generated *SLC4A3* knock‐in (KI) zebrafish model (hereafter referred to as *slc4a3* KI zebrafish) by genome editing, and the KI offspring were used for analysis. Action potential durations (APD) were recorded in zebrafish at 3 days post‐fertilization (dpf) and corrected by Bazett's formula for varying beating frequencies (cAPD).

**Results:** We identified two novel variants in *KCNH2* (c.280 c>t, p.H70Y) and *SLC4A3* (c.1059 c>a, p.N353K) in a SQTS patient. Both variants were co‐segregated in the patient's family with short QT intervals. *KCNH2*‐H70Y did not increase *I*_*K*r,_ indicating that *KCNH2*‐H70Y is not responsible for causing SQTS. Cells expressing *SLC4A3*‐N353K showed significantly slower pHi alternations than those with WT, suggesting the loss‐of‐function effect in regulating pHi. In the electrophysiological analysis, APD70 and cAPD70 were significantly shortened in the *slc4a3* KI zebrafish (homo) compared to that of WT, providing evidence for the functional contribution of the *SLC4A3* variant to the shortened QT interval *in vivo*.

**Conclusions:** We identified a novel *SLC4A3* variant, p.N353K, in a family with SQTS and revealed its contributions to the pHi control and the shortened APD. Further analysis will be necessary to clarify how *SLC4A3*‐N353K causes QT shortening.

